# Enantioselective synthesis of tertiary boronic esters through catalytic asymmetric reversed hydroboration

**DOI:** 10.1038/s41467-021-24012-z

**Published:** 2021-06-18

**Authors:** Tao-Tao Gao, Hou-Xiang Lu, Peng-Chao Gao, Bi-Jie Li

**Affiliations:** Center of Basic Molecular Science (CBMS), Department of Chemistry, Tsinghua University, Beijing, China

**Keywords:** Asymmetric catalysis, Asymmetric synthesis, Synthetic chemistry methodology

## Abstract

Chiral tertiary boronic esters are important precursors to bioactive compounds and versatile synthetic intermediates to molecules containing quaternary stereocenters. The development of conjugate boryl addition to α,β-unsaturated amide has been hampered by the intrinsic low electrophilicity of the amide group. Here we show the catalytic asymmetric synthesis of enantioenriched tertiary boronic esters through hydroboration of β,β-disubstituted α,β-unsaturated amides. The Rh-catalyzed hydroboration occurs with previously unattainable selectivity to provide tertiary boronic esters in high enantioselectivity. This strategy opens a door for the hydroboration of inert Michael acceptors with high stereocontrol and may provide future applications in the synthesis of biologically active molecules.

## Abstract (web summary)

The development of conjugate boryl addition to α,β-unsaturated amide has been hampered by the intrinsic low electrophilicity of the amide group. Here the authors show a catalytic asymmetric synthesis of enantioenriched tertiary boronic esters through hydroboration of β,β-disubstituted α,β-unsaturated amides.

## Introduction

The organoboron compounds have found widespread applications in the design of functional materials and chemical sensors^1,2^. In addition, organoborons exhibit important biological properties, including antibacterial, anticancer, and antiviral activities^3,4^. These properties have spurred the development of novel therapeutic agents for drug discovery. Moreover, chiral, non-racemic organoboronates are valuable compounds in organic synthesis. A growing list of methods have been developed that enable stereospecific conversion of these compounds to a broad range of functionalized molecules^5–11^. Tertiary boronic esters are particularly attractive because they provide rapid access to quaternary stereocenters through subsequent transformations^12–19^.

**Fig. 1:**
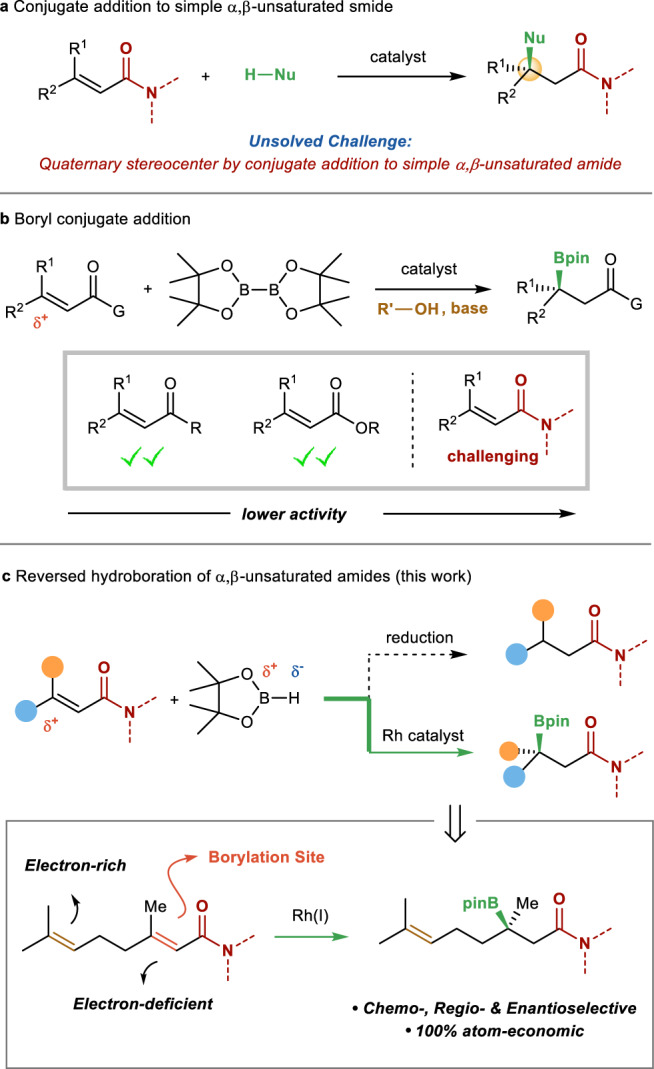
Catalytic hydroboration of alkenes. **a** Conjugate addition to α,β-unsaturated amides. **b** Boryl conjugate addition. **c** Reversed hydroboration of α,β-unsaturated amides.

Pioneering work from Yun^20,21^, Shibasaki^22,23^, Hoveyda^16,24,25^, and others^26–31^ have established metal-catalyzed boryl conjugate addition as a powerful method for the synthesis of chiral tertiary boronic esters. Starting from α,β-unsaturated compounds, such as ketones and esters, tertiary boronic esters were generated in high enantioselectivities^32–34^. As one of the most important functional groups in organic chemistry, the amide group is ubiquitous in proteins, drugs, and pharmaceutically active compounds^35,36^. However, due to the intrinsic low electrophilicity of the amide group, the activity of α,β-unsaturated amide is significantly lower as a Michael acceptor^37–39^. The delocalization of the nitrogen lone pair makes carboxamide the least electron-deficient carboxylic acid derivative. As a result, despite significant advances made in catalytic conjugate additions, currently there are only limited successful examples of catalytic asymmetric conjugate additions to simple α,β-unsaturated amides^40–46^, and very few of them formed a quaternary stereocenter (Fig. 1a)^47^. In particular, metal-catalyzed boryl conjugate to simple unsaturated amides has been limited to the synthesis of secondary boronic ester^30,48–51^. There is one example for a copper-catalyzed enantioselective addition to acyclic β,β-disubstituted α,β-unsaturated amide to form a tertiary boronic ester, in which a β-aryl group is necessary to activate the substrate (Fig. 1b)^52^. Thus, catalytic asymmetric conjugate addition to inert α,β-unsaturated amides to form quaternary stereocenters remains a significant challenge in asymmetric catalysis. A general protocol for catalytic asymmetric hydroboration of β,β-disubstituted α,β-unsaturated amide to form a tertiary boronic ester remains undeveloped.

We surmised that a metal-catalyzed reversed hydroboration of β,β-disubstituted α,β-unsaturated amide would provide an entry to address this challenge (Fig. 1c)^53–56^. In this strategy, a hydride is first incorporated at the α position followed by delivery of the boryl group at the β position, which is mechanistically distinct from a boryl conjugate addition (vide infra). In this type of mechanism, the migratory insertion into the metal hydride by an alkene with low polarity may occur, thus overcoming the inherent low electrophilicity of α,β-unsaturated amide. If the regioselectivity is effectively controlled and the enantioface of the alkene is successfully discriminated, this method would enable facile access to chiral tertiary boronic esters from inert α,β-unsaturated amides.

However, we are aware of substantial challenges associated with this design. First, hydroboration of electron-deficient alkenes has been notoriously problematic. Due to the inherent electronic requirement, metal-catalyzed hydroboration of an α,β-unsaturated compound affords a boron enolate^57,58^. This type of selectivity has long impeded the development of catalytic enantioselective hydroboration of widely existing electron-deficient alkenes. Second, in addition to the electronic requirement that favors the enolate formation, the steric hindrance at the β position also prefers the addition of a small hydride at this site. The formation of a sterically hindered tertiary boronic ester is disfavored. Third, to obtain high enantioselectivity, the catalyst must be able to differentiate between two similar alkyl groups at the β position in the acyclic system. Therefore, in order to achieve the hydroboration of β,β-disubstituted α,β-unsaturated amide, the catalyst must exert substantial control to override both the electronic and steric preferences, and to obtain high enantioselectivity.

We report here a catalytic asymmetric hydroboration of inert α,β-unsaturated amides to construct tertiary boronic esters. The catalyst system we developed is able to (1) reverse the inherent electronic requirement of a hydroboration process, (2) overcome the steric hindrance at the disubstituted β position, and (3) distinguish between the similar steric size of the two β substituents. Starting from easily available materials, this atom-economic process provides a facile method to generate tertiary boronic esters with high regio- and enantioselectivities. This strategy opens a door for the catalytic conjugate addition of inert disubstituted α,β-unsaturated amide to generate quaternary stereocenters.

## Results and discussion

### Reaction development

To take advantage of amide as a key designing element^59–63^, we began our study by testing the hydroboration of 1a and pinacolborane (HBpin; Table 1). In the presence of Rh(COD)_2_OTf, a series of chiral ligands were tested. However, only the reduction product (**2a**) was detected (entries 1–7). After substantial effort, we found that in the presence of (+)-DIOP ligand **L8** (entry 8)^64^, the reversed hydroboration product **3a** was obtained in 45% yield together with reduction product. In addition, the tertiary boronic ester was formed in good enantioselectivity. Subsequently, various solvents were tested (entries 9–12). When the reaction was conducted in 1,2-difluorobenzene, the selectivity was improved, favoring the formation of **3a**. Lowering the reaction temperature to 0 °C led to an increase ee of 93% (entry 13). Further lowering the temperature to −20 °C increased the enantioselectivity and suppressed the undesired reduction product (entry 14). Interestingly, the addition of catalytic amount of *tert*-butanol (*t*BuOH) further improved the yield (entry 15), although its role in the reaction is still unclear (see the Supplementary information). Lowering the catalyst loading to 2 mol% did not affect the yield (entry 16).

**Table 1:** Optimization of reaction conditions^a^.


Entry	Ligand	Solvent	Temp. (°C)	2a (yield%)	3a (yield%)	3a (ee%)
1	**L1**	THF	50	21	<5	—
2	**L2**	THF	50	27	<5	—
3	**L3**	THF	50	48	<5	—
4	**L4**	THF	50	40	<5	—
5	**L5**	THF	50	24	<5	—
6	**L6**	THF	50	38	<5	—
7	**L7**	THF	50	15	<5	—
8	**L8**	THF	50	29	45	85
9	**L8**	DCE	50	15	7	82
10	**L8**	Toluene	50	35	39	82
11	**L8**	1,4-Dioxane	50	29	51	86
12	**L8**	1,2-C_6_H_4_F_2_	50	6	76	85
13	**L8**	1,2-C_6_H_4_F_2_	0	7	89	93
14	**L8**	1,2-C_6_H_4_F_2_	−20	<5	76	96
15^b^	**L8**	1,2-C_6_H_4_F_2_	−20	<5	91	96
16^c^	**L8**	1,2-C_6_H_4_F_2_	−20	<5	90 (87)	96

*THF* tetrahydrofuran, *DCE* 1,2-dichloroethane.

^a^Reaction performed on 0.20 mmol scale. Yields were determined by GC using *n*-dodecane as an internal standard. Isolated yield in parenthesis.

^b^10 mol% *t*BuOH added.

^c^2% Rh catalyst, 4 mol% *t*BuOH.

### Reaction scope

**Fig. 2:**
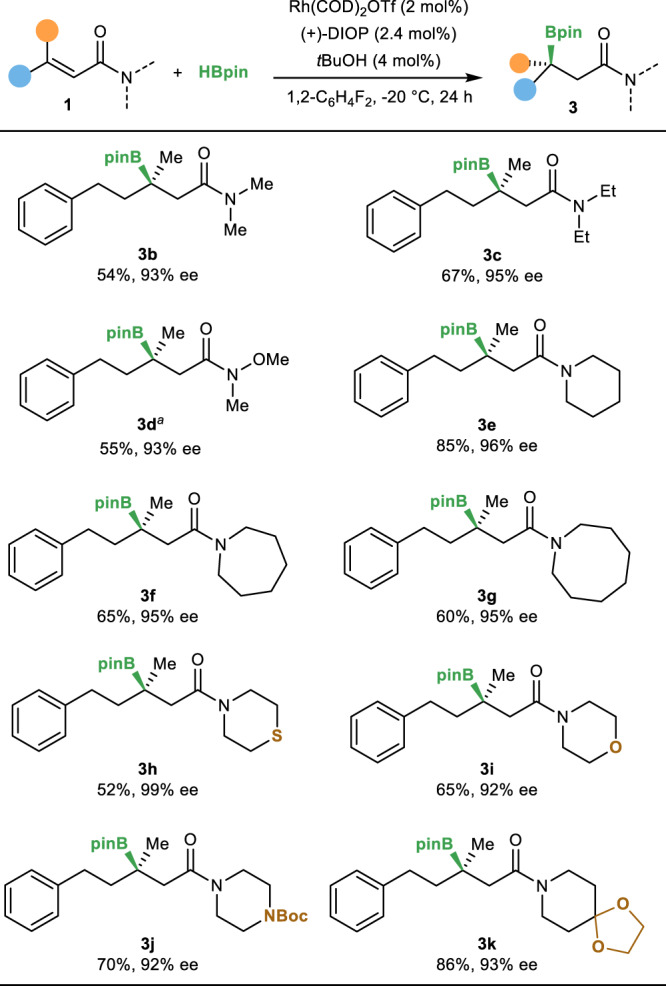
Amide scope. Reversed hydroboration of *N*-substituted α, β-unsaturated amide. ^a^The product was isolated after oxidation. Boc *t*-butyloxy carbonyl.

With this simple yet effective conditions in hand, we investigated the scope of various α,β-unsaturated amides. As summarized in Fig. 2, α,β-unsaturated amides derived from a variety of amines underwent the reversed hydroboration to give tertiary boronic esters in good yields and high enantioselectivities. The reaction is applicable to substrates derived from both acyclic amines (**3b**–**3d**) and cyclic amines (**3e**–**3k**). The successful hydroboration of Weinreb amide (**3d**) provides an opportunity to access ketone product. Functional groups, including sulfur, ether, carbamate, and acetal, were compatible with this system (**3h**–**3k**). However, hydroboration of α,β-unsaturated secondary amide with a free NH group did not provide any desired product.

**Fig. 3:**
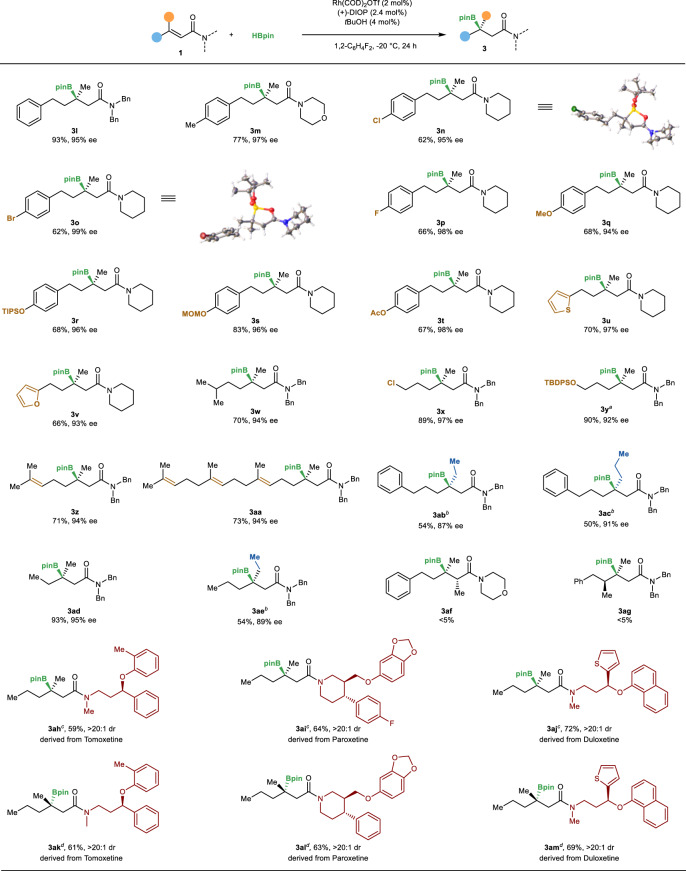
Substrate scope. Reversed hydroboration of β,β-disubstituted α,β-unsaturated amide. ^a^5 mol% Rh catalyst. ^b^7.5 mol% Rh catalyst, 3.5 equivalent of HBpin, 0 °C. The product was isolated after oxidation. ^c^5 mol% Rh catalyst. The product was isolated after oxidation. ^d^5 mol% Rh catalyst, 6.0 mol% (−)-DIOP. The product was isolated after oxidation. TIPS triisopropylsilyl, MOM methoxymethyl, TBDPS *t*-butyldiphenylsilyl.

Next, we sought to explore the reversed hydroborations of various β-disubstituted α,β-unsaturated amides (Fig. 3). A series of β-disubstituted α,β-unsaturated amides underwent regioselective hydroboration to afford the corresponding products in good yields and high enantioselectivities. Aryl and alkyl halide, ether, silyl ether, ester, and heteroarenes were well compatible with the catalytic system (**3l**–**3y**). The absolute configurations of compounds **3n** and **3o** were unambiguously confirmed by X-ray crystallography. In addition, the reaction is highly chemoselective, as demonstrated by the preferential reaction of electron-deficient alkene in the presence of electron-rich ones (**3z**, **3aa**). Furthermore, the catalyst system tolerated not only β-methyl substituent, but also larger ethyl and propyl groups (**3ab**, **3ac**). Importantly, the high enantioselectivities obtained by **3ad** and **3ae** highlighted the ability of the catalyst to differentiate quite similar groups. However, the attempts to form vicinal quaternary and tertiary stereocenters failed despite significant efforts, likely because of the increased steric hindrance (**3af**, **3ag**).

The synthetic utility of this reversed hydroboration was further probed by the reactions of amides derived from drug molecules. Hydroboration of α,β-unsaturated amides derived from tomoxetine, paroxetine, and duloxetine generated the products with high diastereoselectivities (**3ah**, **3ai**, **3aj**), no racemization occurred for the existing stereocenters. In addition, the stereoisomers of **3ah**, **3ai**, **3aj** were also obtained with high diastereoselectivities, respectively, when we use (−)-DIOP as the ligand.

### Transformation of products

**Fig. 4:**
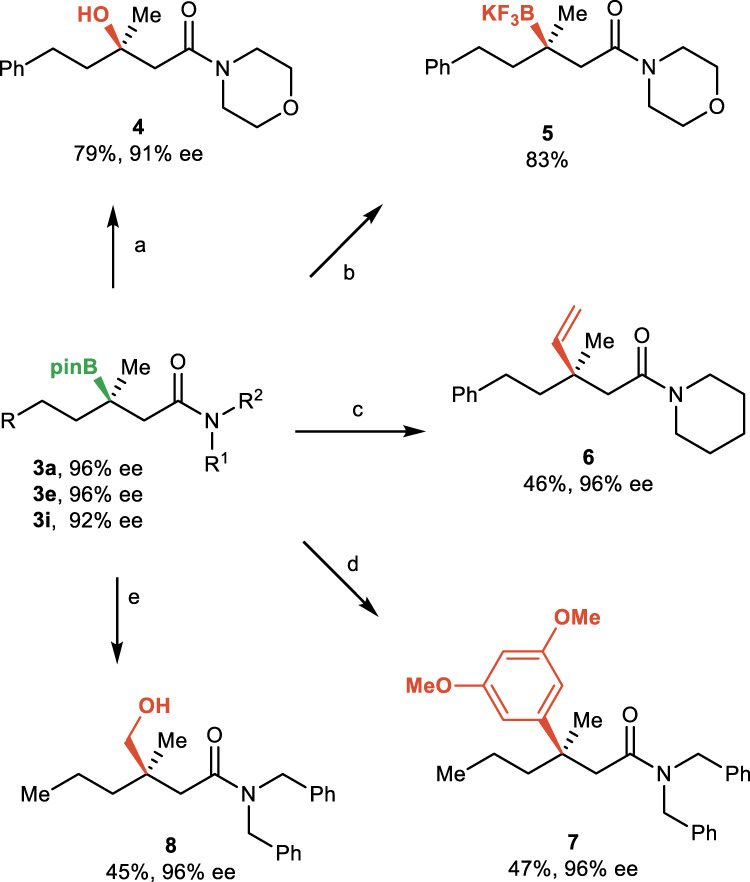
Synthetic utility. Further transformations of the hydroboration products. Reaction conditions: **a** NaBO_3_, H_2_O, THF; **b** KHF_2_, MeCN, H_2_O; **c** (1) vinylMgBr, THF, r.t; (2) I_2_, MeOH, −78 °C; (3) NaOMe, MeOH; **d** (1) 1-bromo-3,5-dimethoxybenzene, *t*BuLi, THF, −78 °C; (2) NBS, THF, −78 °C; **e** (1) ClCH_2_I, *n*BuLi, THF, −78 °C; (2) H_2_O_2_, NaOH, MeOH, H_2_O.

The resulting reversed hydroboration products could be further transformed to a series of functional groups (Fig. 4). For example, enantioenriched tertiary alcohol **4** could be obtained by stereospecific oxidation of the boron compound **3i**. Treatment of **3i** with KHF_2_ yields the potassium trifluoroborate salt **5** in 83% yield. In addition, tertiary boronic esters underwent C–C bond formation with vinyl Grignard or aryl lithium reagent to afford vinylation and arylation compounds **6** and **7** bearing an all-carbon quaternary stereocenter in high enantioselectivities, respectively. Finally, a homologation of the tertiary boronic ester occurred without erosion of the enantioselectivity (**8**).

### Mechanistic studies

**Fig. 5:**
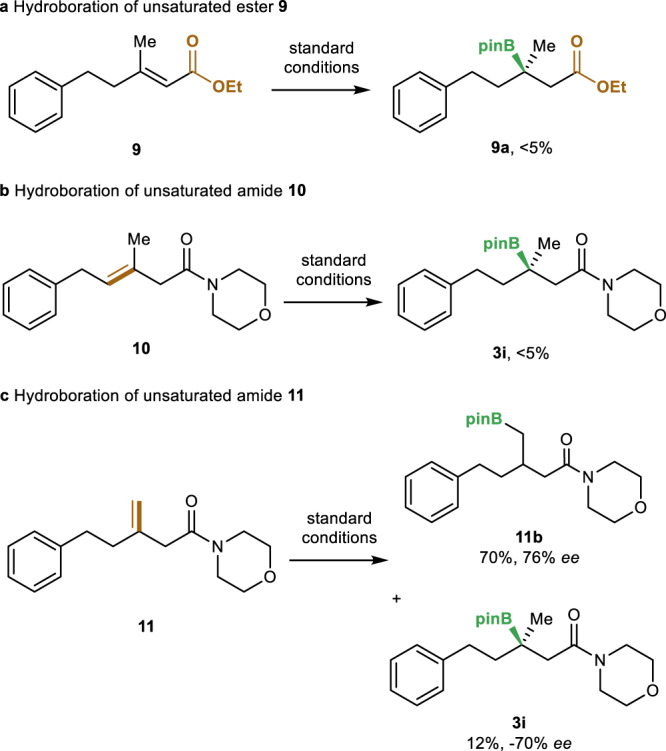
Control experiments. **a** Hydroboration of unsaturated ester **9**. **b** Hydroboration of unsaturated amide **10**. **c** Hydroboration of unsaturated amide **11**.

To gain insight into the reaction mechanism, a series of control experiments were performed. First, when α,β-unsaturated ester **9** was used, no reversed hydroboration product **9a** was observed under the standard reaction conditions (Fig. 5a). Thus, the coordinating ability of an amide group plays a crucial role in reaction system. To probe the possibility of a reaction sequence involving alkene isomerization followed by directed alkene hydroboration, catalytic hydroboration of β,γ-unsaturated amides **10** and **11** were conducted. No hydroboration product was observed when using β,γ-unsaturated amide **10** (Fig. 5b). In addition, the hydroboration of β,γ-unsaturated amide **11** occurred preferentially at the γ position (Fig. 5c). The β-boration product **3i** was obtained in low yield and, more importantly, opposite sense of enantioselectivity. These results provide evidence that the reverse hydroboration occurs directly with α,β-unsaturated amides without prior isomerization.

### Computational studies

**Fig. 6:**
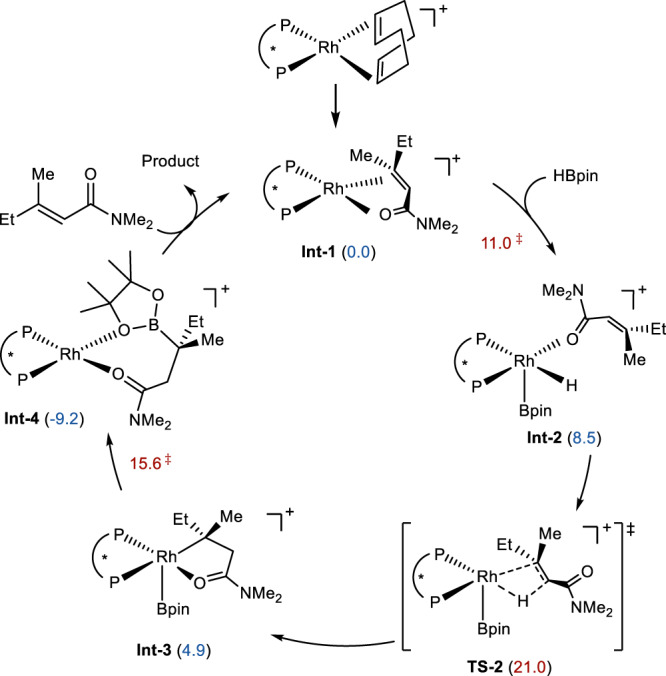
Proposed mechanism. Catalytic cycle with computed free energies of transition states (red) and intermediates (blue) in kcal/mol.

Computational studies provided information of the energy of each step in the catalytic cycle (Fig. 6). Oxidative addition of HBpin to the amide bound rhodium complex (**Int-1**) generates a five-coordinated rhodium hydride (**Int-2**). After migratory insertion of the alkene into the rhodium hydride, an alkyl rhodium complex (**Int-3**) is formed, in which the amide is coordinated to the metal center. Finally, C–B forming reductive elimination delivers the hydroboration product and regenerates the catalyst through ligand exchange. The calculated energies suggest that the migratory insertion is irreversible and determines the enantioselectivity.

**Fig. 7:**
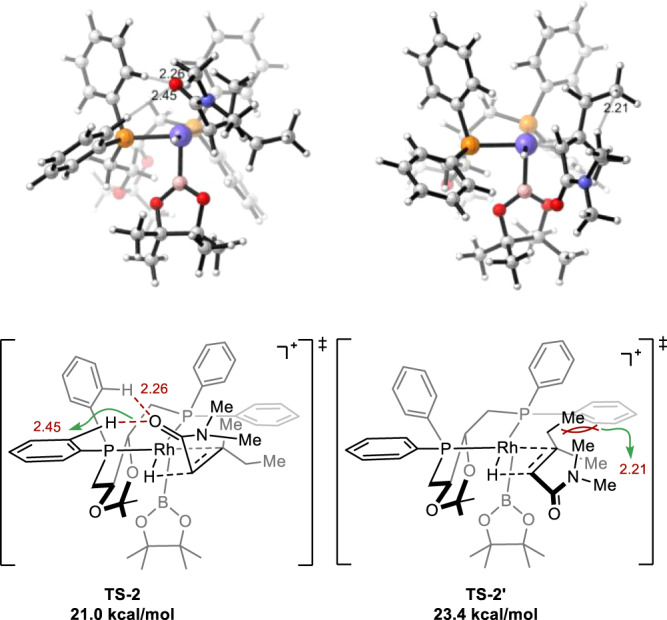
Structures of competing transition states. Transition state structures leading to major enantiomer (**TS-2**) and minor enantiomer (**TS-2′**).

To further understand the origin of enantioselectivity, the energies of different transition states for migration insertion were computed (Fig. 7). The lowest energy pathway leading to the ***S*** enantiomer has an activation barrier of 21.0 kcal/mol (**TS-2**), while the lowest energy pathway leading to the *R* enantiomer has an activation barrier of 23.4 kcal/mol (**TS-2′**). The energy difference between **TS-2** and **TS-2′** (2.4 kcal/mol) correlates with the major enantiomer observed in experiments. In **TS-2′**, the α,β-unsaturated amide coordinates to rhodium through the opposite enantioface to that in **TS-2**. In **TS-2**, the phenyl group on the phosphine atom of the ligand forms two attractive CH^…^O interaction with the carbonyl group of the substrate (CH^…^O distance 2.26 and 2.45 Å, respectively)^65,66^. In contrast, such interaction is not observed in **TS-2′** due to the orientation of the carbonyl group. In addition, the phenyl group on the ligand compels the substrate in a way that the ethyl group experiences significant repulsion with the *N*-methyl group (**TS-2′**). Therefore, the CH^…^O interaction with the phenyl group on the ligand in **TS-2** and the repulsive interaction in **TS-2′** contributes to the relative energies of these transition states, and leads to the high enantioselectivity observed.

In summary, we have developed a Rh-catalyzed asymmetric reversed hydroboration to construct chiral tertiary boronic esters. Using a catalyst formed from cationic rhodium and DIOP ligand, hydroboration of β,β-disubstituted α,β-unsaturated amides delivers chiral tertiary boronic esters with high regioselectivity and enantioselectivity. Computation studies revealed the origin of enantioselectivity. Further mechanistic studies and application of this methodology is ongoing in our laboratory.

## Methods

### General procedure for the catalytic hydroboration

In an Ar-filled glovebox, the α,β-unsaturated amide (0.25 mmol, 1.0 equiv.), Rh(COD)_2_OTf (2.3 mg, 2.0 mol%), and (+)-DIOP (3.0 mg, 2.4 mol%) were weighed into a one-dram screw-capped vial. Subsequently, 1,2-difluorobenzene (2.5 mL), *t*BuOH (4.0 mol%), and pinacolborane (80.0 mg, 2.5 equiv.) were added via syringes. The vial was capped with a Teflon-lined screw cap, and the reaction was then removed from the glovebox and stirred at −20 °C for 24 h. Then the solution was concentrated under reduced pressure. After removal of the solvent, the crude product was analyzed by ^1^H NMR and purified by column chromatography on silica gel with EtOAc/hexanes mixture as eluent.

### Reporting summary

Further information on research design is available in the Nature Research Reporting Summary linked to this article.

## Supplementary information

Supplementary Information

Reporting Summary

## Data Availability

The authors declare that all the data supporting the findings of this research are available within the article and its Supplementary Information. Crystallographic data of compounds **3n** and **3o** data have been deposited in the Cambridge Crystallographic Data Center under accession number CCDC: 2031005 and 2031762.
