# Supplementary material for: Enantioselective synthesis of tertiary boronic esters through catalytic asymmetric reversed hydroboration
Source: Nat Commun. 2021 Jun 18;12:3776. doi: 10.1038/s41467-021-24012-z (PMC8213697; doi:10.1038/s41467-021-24012-z)
Supplement: Supplementary file 1 — Supplementary Information [file 41467_2021_24012_MOESM1_ESM.pdf]

Supplementary Information

Enantioselective Synthesis of Tertiary Boronic Esters Through Catalytic Asymmetric Reversed Hydroboration

Tao-Tao Gao, Hou-Xiang Lu, Peng-Chao Gao and Bi-Jie Li*

Center of Basic Molecular Science (CBMS), Department of Chemistry, Tsinghua University

Table of Contents

Supplementary Methods S2

General Procedure for the Synthesis of α,β-Unsaturated Carboxylic Acid S2

General Procedure for the Synthesis of α,β-Unsaturated Amide S3

Characterization of Substrates S6

Characterization of Products..S21

Synthetic Transformation of Boration Products S54

Substrates and Products for Control Experiments S59

General Information for Computation S62

Copies of NMR Spectra...S84

Supplementary References...S173

Supplementary Methods

Unless otherwise noted, all reactions were assembled on a Schlenk vacuum line or in a glovebox using oven-dried glassware and were stirred with Teflon-coated magnetic stirring bars. All the ligands were purchased from Strem Chemicals and were used as received. [Rh(cod)$_2$OTf] was prepared according to literature methods.[1] Pinacolborane was purchased from Energy Chemical and was used as received. 1,2-Difluorobenzene was purchased from Energy Chemical and was distilled over calcium hydride. Tetrahydrofuran (THF) and acetonitrile (CH_3CN) were degassed by purging with nitrogen and then dried with a solvent purification system containing activated alumina. All other solvents and reagents were used as received. All work-up and purification procedures were carried out with reagent grade solvents in air. Reaction temperatures above 23 ℃ refer to temperatures of an aluminum heating block or a silicon oil bath, which were controlled by an electronic temperature modulator from IKA. NMR spectra were acquired on NMR spectrometer with 400 MHz for **^{1}H NMR** and 101 MHz for **^{13}C NMR** at the NMR facility at Center of Basic Molecular Science (CBMS). Chemical shifts (δ) are reported in ppm relative to the residual solvent signal. Data for ^{1}H NMR spectra are reported as follows: chemical shift (multiplicity, coupling constants, number of hydrogens). Abbreviations are as follows: s (singlet), d (doublet), t (triplet), q (quartet), m (multiplet), br (broad). Infrared (IR) spectra were recorded on a Bruker FT–IR alpha (ATR mode) spectrophotometer. High-resolution mass spectral data was performed on a Thermo Scientific Q Exactive (positive mode) at the Mass Spectrometry Facility, CBMS. Enantiomer excess (ee) values were determined by analytical liquid chromatography (HPLC) analysis on a Shimadzu chromatograph (Daicel chiral columns Chiralpak IA, IC, ID, IE, AD, OD, AS, OJ (4.6 x 250 mm)). Specific rotations were measured on a Jasco P-2000 Polarimeter.

General procedure for the synthesis of (*E*)-α, β-unsaturated carboxylic acid

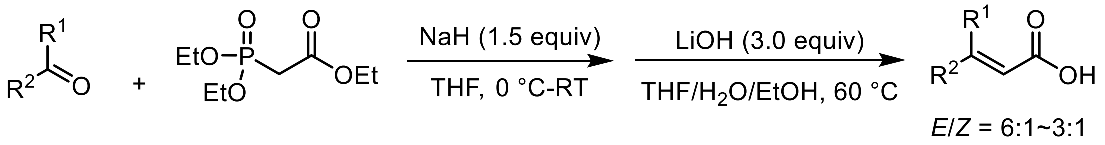

To a 250 mL flask with a Teflon-coated magnetic stir bar was added NaH (24.0 mmol, 1.2 equiv.) and THF (60 mL). Then ethyldiethylphosphonoacetate (24.0 mmol, 1.2 equiv.) was added dropwise at 0 °C. After stirring at 0 °C for 1 hour, the ketone (20.0 mmol, 1.0 equiv.) was added dropwise. After stirring 5 hours at room temperature, the reaction was cooled down and quenched with saturated NH_4Cl solution and extracted with EtOAc (3 x 50 mL). The combined organic layer was washed with water (20 mL), brine (20 mL) and dried over Na_2SO_4. After removal of the solvent, the residue was purified by column chromatography on silica gel with EtOAc/hexanes as eluent, affording the (*E*)-*α,β*-unsaturated ester.

To the solution of (*E*)-*α, β*-unsaturated ester (10.0 mmol, 1.0 equiv.) in THF (20 mL), H_2O (20 mL), EtOH (20 mL), and LiOH (30 mmol, 3.0 equiv.) were added at room temperature. The reaction mixture was stirred at 60 °C for 5 hours and monitored by TLC. After completion of the reaction, the reaction mixture was cooled to 0 °C and was acidified until pH=3 using 1 M HCl solution. EtOAc (50 mL) was added, and the mixture was washed with water (20 mL), brine (20 mL) and dried over Na_2SO_4. The concentrated reaction mixture was used directly in next step without further purification.

General procedure for the synthesis of *α, β*-unsaturated amide

To a 100 mL round-bottom flask with a Teflon-coated magnetic stir bar were added *α,β*-unsaturated carboxylic acid (5.0 mmol, 1.0 equiv.), 1-ethyl-3-(3-(dimethylamino)propyl)-carbodiimide hydrochloride (EDCI) (7.5 mmol, 1.5 equiv.), *N*,*N*-dimethylaminopyridine (0.50 mmol, 10 mol%) , the amine (6.0 mmol, 1.2 equiv.) and dichloromethane (30.0 mL). The reaction mixture was stirred at room temperature for 12 h. Dichloromethane (50 mL) was added, and the mixture was washed with water (20 mL), brine (20 mL) and dried over Na_2SO_4. After removal of the solvent, the residue was purified by column chromatography on silica gel with EtOAc/hexanes mixture as eluent.

Synthesis of *α, β*-unsaturated amide **1ab**, **1ac**,**1ae, 10** and **11:** To a 100 mL round-bottom flask with a Teflon-coated magnetic stir bar were added *α,β*-unsaturated carboxylic acid (5.0 mmol, 1.0 equiv.), 2-(7-azabenzotriazol-1-yl)-*N*,*N*,*N'*,*N'*-

tetramethyluronium hexafluorophosphate (HATU) (8.0 mmol, 1.6 equiv.), 2,4,6-trimethylpyridine (10.0 mmol, 2.0 equiv.), the amine (6.0 mmol, 1.2 equiv.) and dichloromethane (30.0 mL). The reaction mixture was stirred at room temperature for 12 h. Dichloromethane (50 mL) was added, and the mixture was washed with water (20 mL), brine (20 mL) and dried over Na_2SO_4. After removal of the solvent, the residue was purified by column chromatography on silica gel with EtOAc/hexanes mixture as eluent.

According to the general procedure, the hydroboration using various additives was analyzed. It seems only weakly protic solvent additives have the beneficial effect. Our proposal is that the alcohol coordinates to the catalytic intermediates through hydrogen bonding to facilitate the catalytic cycle, but further evidence is necessary.

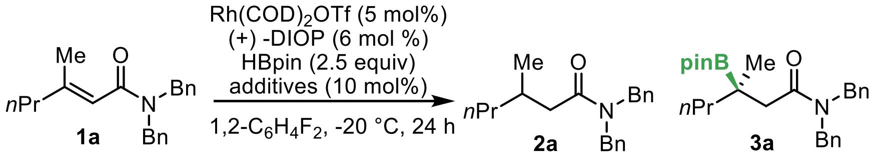

Entry	additives	**2a** (yield%)	**3a** (yield%)	**3a** (ee%)
1	**-**	<5	76	96
2	**THF**	16	57	93
3	**Toluene**	13	18	92
4	**DCM**	7	9	91
5	**$CHCl_3$**	12	<5	-
6	**MeOH**	<5	90	96
7	**TFEA**	<5	92	95
8	**Phenol**	<5	70	96

Reaction performed on 0.10 mmol scale. Yields were determined by GC using *n*-dodecane as an internal standard.

Supplementary Figure 1. Additives in the reversed hydroboration

Catalytic hydroboration of Z-amide was also tested. However, isomerization of the *Z*-isomer to *E*-isomer occurred at a rate comparable to the hydroboration. Thus, only moderate ee was obtained for *Z*-isomer. The reaction of a mixture of *E*/*Z* alkenes also provided low ee because of the isomerization.

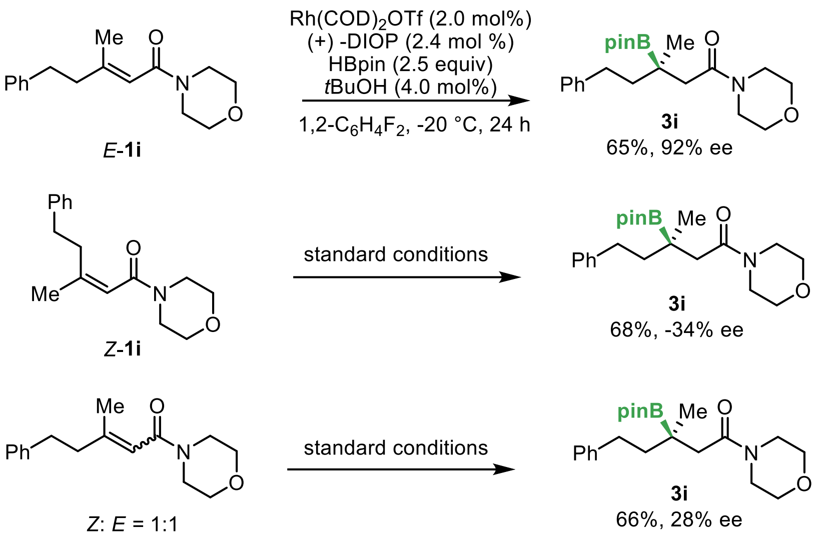

Supplementary Figure 2. Hydroboration of (*Z*)-alkene and (*Z*), (*E*)-alkene mixture

Characterization of Substrates

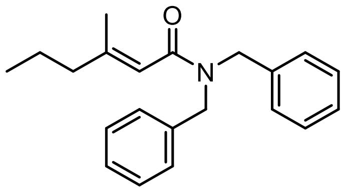

amide 1a: colorless oil. TLC analysis (hexanes/EtOAc 11:1) R_f= 0.21; **^{1}H NMR** (400 MHz, $CDCl_3$) δ 7.42 – 7.09 (m, 10H), 5.92 (q, *J* = 1.3 Hz, 1H), 4.59 (s, 2H), 4.45 (s, 2H), 2.05 (td, *J* = 7.4, 1.2 Hz, 2H), 2.01 (s, 3H), 1.45 (h, *J* = 7.4 Hz, 2H), 0.85 (t, *J* = 7.4 Hz, 3H). **^{13}C NMR** (101 MHz, $CDCl_3$) δ 169.1, 151.6, 137.7, 137.0, 129.0, 128.7, 128.5, 127.7, 127.4, 127.0, 117.4, 50.5, 47.3, 42.2, 20.8, 18.8, 13.8. **ESI-HR** calcd for $C_{21}H_{26}NO^+$ ($[M+H]^+$) 308.2009, found 308.2003. **IR** ν (cm^{-1}) 2958, 2926, 2863, 1625, 697.

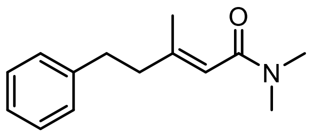

amide 1b: colorless oil. TLC analysis (hexanes/EtOAc 1:2) R_f= 0.42; **^{1}H NMR** (400 MHz, $CDCl_3$) δ 7.34 – 7.13 (m, 5H), 5.71 (t, *J* = 1.3 Hz, 1H), 2.94 (s, 3H), 2.79 (d, *J* = 7.4 Hz, 5H), 2.42 (td, *J* = 7.7, 1.2 Hz, 2H), 1.93 (d, *J* = 1.3 Hz, 3H). **^{13}C NMR** (101 MHz, $CDCl_3$) δ 168.7, 147.5, 141.5, 128.5, 126.1, 118.9, 41.4, 37.6, 34.7, 33.9, 18.5. **ESI-HR** calcd for $C_{14}H_{20}NO^+$ ($[M+H]^+$) 218.1539, found 218.1537. **IR** ν (cm^{-1}) 2923, 1651, 1621, 744.

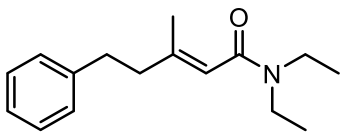

amide 1c: colorless oil. TLC analysis (hexanes/EtOAc 2:1) R_f= 0.34; **^{1}H NMR** (400 MHz, $CDCl_3$) δ 7.34 – 7.13 (m, 5H), 5.71 (q, *J* = 1.3 Hz, 1H), 3.38 (q, *J* = 7.1 Hz, 2H), 3.09 (q, *J* = 7.2 Hz, 2H), 2.81 (dd, *J* = 8.7, 6.8 Hz, 2H), 2.48 – 2.37 (m, 2H), 1.94 (d, *J* = 1.3 Hz, 3H), 1.12 (t, *J* = 7.1 Hz, 3H), 1.00 (t, *J* = 7.1 Hz, 3H). **^{13}C NMR** (101 MHz, $CDCl_3$) δ 167.9, 147.1, 141.5, 128.5, 128.5, 126.1, 119.1, 42.4, 41.2, 39.4, 33.8, 18.5, 14.3, 13.3.

ESI-HR calcd for $C_{16}H_{24}NO^+$ ($[M+H]^+$) 246.1852, found 246.1849. **IR** ν (cm^{-1}) 2975, 2934, 1650, 1611, 726.

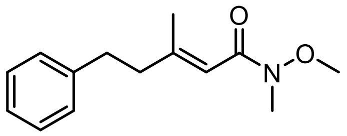

amide 1d: colorless oil. TLC analysis (hexanes/EtOAc 2:1) R_f= 0.46; **^{1}H NMR** (400 MHz, $CDCl_3$) δ 7.30 – 7.22 (m, 2H), 7.16 (d, J = 2.0 Hz, 3H), 6.05 (s, 1H), 3.52 (s, 3H), 3.15 (s, 3H), 2.80 (dd, J = 9.0, 6.6 Hz, 2H), 2.46 (dd, J = 9.0, 6.7 Hz, 2H), 2.25 – 2.13 (m, 3H). **^{13}C NMR** (101 MHz, $CDCl_3$) δ 168.0, 155.2, 141.3, 128.5, 128.5, 126.1, 114.7, 61.4, 42.8, 34.0, 32.3, 18.8. **ESI-HR** calcd for $C_{14}H_{20}NO_2^+$ ($[M+H]^+$) 234.1489, found 234.1485. **IR** ν (cm^{-1}) 2935, 1653, 1637, 1495, 1368, 698.

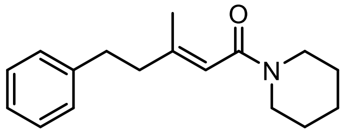

amide 1e: colorless oil. TLC analysis (hexanes/EtOAc 1:1) R_f= 0.45; **^{1}H NMR** (400 MHz, $CDCl_3$) δ 7.27 (dd, *J* = 8.6, 6.5 Hz, 2H), 7.22 – 7.11 (m, 3H), 5.65 (q, *J* = 1.3 Hz, 1H), 3.53 (t, *J* = 5.5 Hz, 2H), 3.11 (dd, *J* = 6.6, 4.5 Hz, 2H), 2.80 (dd, *J* = 8.5, 6.8 Hz, 2H), 2.42 (td, *J* = 7.5, 1.1 Hz, 2H), 1.86 (d, *J* = 1.2 Hz, 3H), 1.63 – 1.48 (m, 4H), 1.39 (dq, *J* = 11.3, 5.6 Hz, 2H). **^{13}C NMR** (101 MHz, $CDCl_3$) δ 167.3, 145.4, 141.4, 128.5, 128.4, 126.0, 119.6, 47.2, 42.2, 40.9, 33.7, 26.7, 25.7, 24.7, 18.4. **ESI-HR** calcd for $C_{17}H_{24}NO^+$ ($[M+H]^+$) 258.1852, found 258.1849. **IR** ν (cm^{-1}) 2935, 1650, 1616, 731.

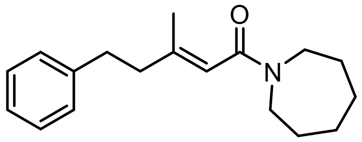

amide 1f: colorless oil. TLC analysis (hexanes/EtOAc 1:1) R_f= 0.50; **^{1}H NMR** (400 MHz, $CDCl_3$) δ 7.31 – 7.14 (m, 5H), 5.71 (s, 1H), 3.50 (td, *J* = 6.1, 1.5 Hz, 2H), 3.17 (dt, *J* = 6.0, 2.5 Hz, 2H), 2.80 (t, *J* = 7.7 Hz, 2H), 2.43 (t, *J* = 7.7 Hz, 2H), 1.94 (d, *J* = 1.4 Hz, 3H), 1.74 – 1.67 (m, 2H), 1.57 – 1.46 (m, 6H). **^{13}C NMR** (101 MHz, $CDCl_3$) δ 168.4,

147.0, 141.5, 128.5, 126.0, 119.4, 48.2, 45.4, 41.2, 33.8, 29.3, 27.7, 27.4, 27.0, 18.6. **ESI-HR** calcd for $C_{18}H_{26}NO^+$ ($[M+H]^+$) 272.2009, found 272.2005.**IR** ν (cm^{-1}) 2927, 1650, 1607, 725, 698.

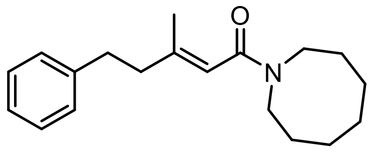

amide 1g: colorless oil. TLC analysis (hexanes/EtOAc 1:1) R_f= 0.44; **^{1}H NMR** (400 MHz, $CDCl_3$) δ 7.31 – 7.15 (m, 5H), 5.72 (q, *J* = 1.3 Hz, 1H), 3.48 – 3.42 (m, 2H), 3.19 – 3.08 (m, 2H), 2.81 (dd, *J* = 8.5, 6.8 Hz, 2H), 2.43 (ddd, *J* = 8.6, 6.8, 1.1 Hz, 2H), 1.96 (d, *J* = 1.2 Hz, 3H), 1.73 (td, *J* = 8.5, 7.3, 4.6 Hz, 2H), 1.50 (d, *J* = 4.2 Hz, 8H). **^{13}C NMR** (101 MHz, $CDCl_3$) δ 168.4, 147.4, 141.5, 128.5, 126.0, 119.5, 49.5, 46.8, 41.4, 33.8, 27.5, 27.1, 26.2, 25.5, 25.3, 18.6. **ESI-HR** calcd for $C_{19}H_{28}NO^+$ ($[M+H]^+$) 286.2165, found 286.2160. **IR** ν (cm^{-1}) 2928, 1650, 1605, 907, 726.

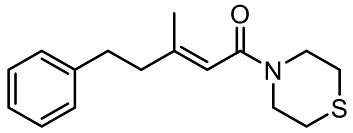

amide 1h: colorless oil. TLC analysis (hexanes/EtOAc 3:1) R_f= 0.38; **^{1}H NMR** (400 MHz, $CDCl_3$) δ 7.27 (dd, *J* = 8.5, 6.6 Hz, 2H), 7.20 – 7.14 (m, 3H), 5.57 (q, *J* = 1.2 Hz, 1H), 3.84 (q, *J* = 5.0 Hz, 2H), 3.38 – 3.25 (m, 2H), 2.81 (t, *J* = 7.5 Hz, 2H), 2.60 – 2.54 (m, 2H), 2.44 (td, *J* = 7.4, 1.1 Hz, 2H), 2.41 – 2.31 (m, 2H), 1.87 (s, 3H). **^{13}C NMR** (101 MHz, $CDCl_3$) δ 167.5, 146.8, 141.2, 128.5, 128.5, 126.0, 119.2, 48.7, 43.7, 40.7, 33.5, 28.2, 27.5, 18.4. **ESI-HR** calcd for $C_{16}H_{22}NOS^+$ ($[M+H]^+$) 276.1417, found 276.1413. **IR** ν (cm^{-1}) 3024, 2910, 1622, 743, 699.

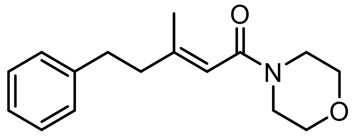

amide 1i: colorless oil. TLC analysis (hexanes/EtOAc 1:1) R_f= 0.37; **^{1}H NMR** (400 MHz, $CDCl_3$) δ 7.27 (dd, *J* = 8.5, 6.5 Hz, 2H), 7.21 – 7.12 (m, 3H), 5.58 (q, *J* = 1.2 Hz, 1H), 3.66 – 3.56 (m, 4H), 3.46 (t, *J* = 4.8 Hz, 2H), 3.06 (t, *J* = 4.7 Hz, 2H), 2.81 (t, *J* = 7.5

Hz, 2H), 2.44 (td, J = 7.4, 1.1 Hz, 2H), 1.90 (d, J = 1.3 Hz, 3H). **^{13}C NMR** (101 MHz, $CDCl_3$) δ 167.4, 147.4, 141.3, 128.6, 128.5, 126.1, 118.7, 67.0, 46.4, 41.6, 41.0, 33.6, 18.4. **ESI-HR** calcd for $C_{16}H_{22}NO_2^+$ ($[M+H]^+$) 260.1645, found 260.1640. **IR** ν (cm^{-1}) 3025, 2916, 1622, 744.

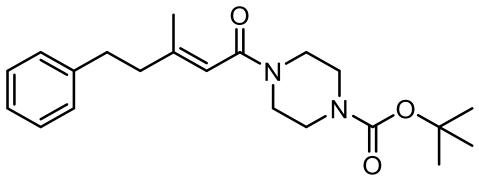

amide 1j: white solid. TLC analysis (hexanes/EtOAc 2:1) R_f= 0.32; **^{1}H NMR** (400 MHz, $CDCl_3$) δ 7.32 – 7.27 (m, 2H), 7.21 – 7.14 (m, 3H), 5.59 (s, 1H), 3.59 – 3.51 (m, 2H), 3.37 (q, J = 5.4, 4.9 Hz, 2H), 3.22 (t, J = 5.1 Hz, 2H), 3.12 – 2.95 (m, 2H), 2.82 (t, J = 7.5 Hz, 2H), 2.45 (td, J = 7.5, 1.1 Hz, 2H), 1.88 (d, J = 1.2 Hz, 3H), 1.47 (s, 9H). **^{13}C NMR** (101 MHz, $CDCl_3$) δ 167.5, 154.7, 147.4, 141.2, 128.6, 128.6, 126.2, 119.0, 80.4, 45.8, 43.8, 41.1, 41.0, 33.6, 28.5, 18.4. **ESI-HR** calcd for $C_{21}H_{30}N_2NaO_3^+$ ($[M+Na]^+$) 381.2149, found 381.2159. **IR** ν (cm^{-1}) 2926, 2859, 1693, 1627, 737. **M. P.** 105-108 °C.

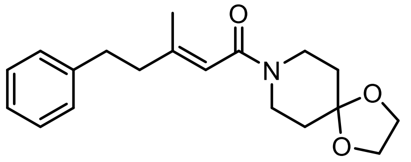

amide 1k: colorless oil. TLC analysis (hexanes/EtOAc 3:1) R_f= 0.15; **^{1}H NMR** (400 MHz, $CDCl_3$) δ 7.28 (dd, J = 8.4, 6.5 Hz, 2H), 7.18 (dd, J = 8.3, 6.5 Hz, 3H), 5.64 (d, J = 1.9 Hz, 1H), 3.97 (s, 4H), 3.67 (t, J = 5.9 Hz, 2H), 3.19 (t, J = 5.8 Hz, 2H), 2.81 (t, J = 7.6 Hz, 2H), 2.44 (t, J = 7.6 Hz, 2H), 1.86 (s, 3H), 1.65 (t, J = 5.9 Hz, 2H), 1.51 (t, J = 5.9 Hz, 2H). **^{13}C NMR** (101 MHz, $CDCl_3$) δ 167.3, 146.3, 141.3, 128.5, 128.5, 126.1, 119.3, 107.1, 64.5, 44.1, 40.9, 39.3, 35.9, 34.8, 33.6, 18.4. **ESI-HR** calcd for $C_{19}H_{26}NO_3^+$ ($[M+H]^+$) 316.1907, found 316.1903. **IR** ν (cm^{-1}) 2931, 1651, 1613, 726.

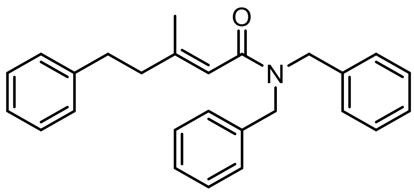

amide 1l: white solid. TLC analysis (hexanes/EtOAc 9:1) R_f = 0.21; **^{1}H NMR** (400 MHz, $CDCl_3$) δ 7.39 – 7.02 (m, 15H), 5.83 (d, *J* = 1.5 Hz, 1H), 4.55 (s, 2H), 4.21 (s, 2H), 2.74 (dd, *J* = 8.7, 6.7 Hz, 2H), 2.41 (t, *J* = 7.7 Hz, 2H), 2.05 (s, 3H). **^{13}C NMR** (101 MHz, $CDCl_3$) δ 169.0, 149.7, 141.3, 137.6, 136.9, 128.9, 128.7, 128.5, 128.4, 128.4, 127.6, 127.4, 127.0, 126.1, 118.5, 50.4, 47.2, 41.5, 33.8, 18.9. **ESI-HR** calcd for $C_{26}H_{28}NO^+$ ($[M+H]^+$) 370.2165, found 370.2159. **IR** ν (cm^{-1}) 3026, 2917,1625,1494, 696. **M. P.** 68-71 ^{o}C.

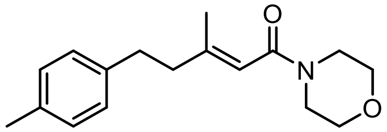

amide 1m: colorless oil. TLC analysis (hexanes/EtOAc 1:1) R_f = 0.28; **^{1}H NMR** (400 MHz, $CDCl_3$) δ 7.06 (q, *J* = 8.0 Hz, 4H), 5.58 (q, *J* = 1.4 Hz, 1H), 3.62 (dq, *J* = 9.9, 5.3, 4.7 Hz, 4H), 3.46 (t, *J* = 4.8 Hz, 2H), 3.12 – 3.03 (m, 2H), 2.77 (t, *J* = 7.5 Hz, 2H), 2.42 (t, *J* = 7.5 Hz, 2H), 2.30 (s, 3H), 1.88 (d, *J* = 1.3 Hz, 3H). **^{13}C NMR** (101 MHz, $CDCl_3$)δ 167.4, 147.5, 138.2, 135.5, 129.2, 128.5, 118.7, 67.0, 46.5, 41.6, 41.2, 33.2, 21.1, 18.4. **ESI-HR** calcd for $C_{17}H_{24}NO_2^+$ ($[M+H]^+$) 274.1802, found 274.1797. **IR** ν (cm^{-1}) 2920, 2855, 1618, 727.

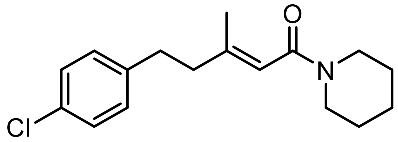

amide 1n: white solid. TLC analysis (hexanes/EtOAc 2:1) R_f = 0.23; **^{1}H NMR** (400 MHz, $CDCl_3$) δ 7.24 (d, *J* = 8.2 Hz, 2H), 7.10 (d, *J* = 8.2 Hz, 2H), 5.62 (d, *J* = 1.9 Hz, 1H), 3.54 (t, *J* = 5.5 Hz, 2H), 3.10 (t, *J* = 5.6 Hz, 2H), 2.78 (t, *J* = 7.6 Hz, 2H), 2.39 (t, *J* = 7.6 Hz, 2H), 1.85 (s, 3H), 1.57 (dq, *J* = 28.7, 6.2, 5.5 Hz, 4H), 1.41 (t, *J* = 5.8 Hz, 2H). **^{13}C NMR** (101 MHz, $CDCl_3$) δ 167.2, 145.1, 139.9, 131.8, 129.9, 128.6, 119.9, 47.3,

42.3, 40.8, 33.0, 26.7, 25.7, 24.7, 18.4. **ESI-HR** calcd for $C_{17}H_{23}ClNO^+$([M+H]$^+$) 292.1463, found 292.1459. **IR** ν (cm^{-1}) 2939, 1651, 1606, 724. **M. P.** 55-57 °C.

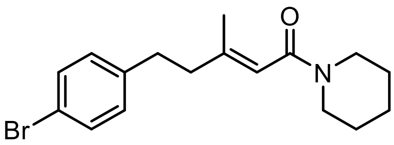

amide 1o: white solid. TLC analysis (hexanes/EtOAc 2:1) R_f= 0.19; ^{1}**H NMR** (400 MHz, $CDCl_3$) δ 7.44 – 7.36 (m, 2H), 7.09 – 7.01 (m, 2H), 5.62 (q, *J* = 1.3 Hz, 1H), 3.54 (t, *J* = 5.5 Hz, 2H), 3.14 – 3.05 (m, 2H), 2.76 (t, *J* = 7.6 Hz, 2H), 2.39 (t, *J* = 7.6 Hz, 2H), 1.85 (d, *J* = 1.2 Hz, 3H), 1.66 – 1.49 (m, 4H), 1.41 (p, *J* = 5.9 Hz, 2H). ^{13}**C NMR** (101 MHz, $CDCl_3$) δ 167.1, 145.0, 140.4, 131.5, 130.3, 120.0, 119.8, 47.2, 42.2, 40.7, 33.1, 26.7, 25.7, 24.7, 18.3. **ESI-HR** calcd for $C_{17}H_{23}BrNO^+$ ([M+H]$^+$) 336.0958, found 336.0952. **IR** ν (cm^{-1}) 2935, 1650, 1614, 731. **M. P.** 58-60 °C.

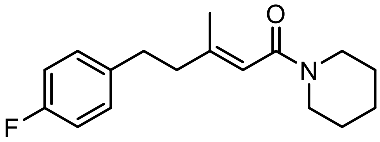

amide 1p: colorless oil. TLC analysis (hexanes/EtOAc 2:1) R_f= 0.30; ^{1}**H NMR** (400 MHz, $CDCl_3$) δ 7.18 – 7.07 (m, 2H), 6.96 (t, *J* = 8.7 Hz, 2H), 5.64 (s, 1H), 3.54 (t, *J* = 5.5 Hz, 2H), 3.23 – 3.04 (m, 2H), 2.78 (t, *J* = 7.6 Hz, 2H), 2.39 (t, *J* = 7.6 Hz, 2H), 1.85 (s, 3H), 1.65 – 1.48 (m, 4H), 1.41 (p, *J* = 5.8 Hz, 2H). ^{13}**C NMR** (101 MHz, $CDCl_3$) δ 167.2, 161.4 (J_1 = 243.6 Hz), 145.2, 137.1 (J_4= 3.3 Hz), 129.9 (J_3= 7.8 Hz), 119.8, 115.2 (J_2= 21.8 Hz), 47.3, 42.2, 41.0, 32.9, 26.7, 25.7, 24.7, 18.4. ^{19}**F NMR** (376 MHz, CDCl3) δ -117.5. **ESI-HR** calcd for $C_{17}H_{23}FNO^+$ ([M+H]$^+$) 276.1758, found 276.1754. **IR** ν (cm^{-1}) 2935, 2850, 1650, 1616, 822.

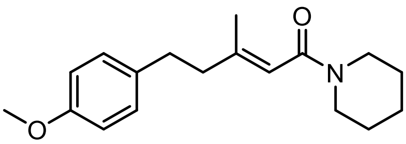

amide 1q: colorless oil. TLC analysis (hexanes/EtOAc 3:2) R_f= 0.29; ^{1}**H NMR** (400 MHz, $CDCl_3$) δ 7.12 – 7.01 (m, 2H), 6.86 – 6.79 (m, 2H), 5.65 (q, *J* = 1.3 Hz, 1H), 3.78 (s,

3H), 3.54 (t, *J* = 5.5 Hz, 2H), 3.14 (q, *J* = 4.9, 4.4 Hz, 2H), 2.74 (dd, *J* = 8.5, 6.8 Hz, 2H), 2.38 (ddd, *J* = 8.6, 6.8, 1.2 Hz, 2H), 1.85 (d, *J* = 1.2 Hz, 3H), 1.65 – 1.49 (m, 4H), 1.47 – 1.31 (m, 2H). **^{13}C NMR** (101 MHz, $CDCl_3$) δ 167.4, 157.9, 145.6, 133.5, 129.4, 119.6, 113.9, 55.3, 47.3, 42.2, 41.2, 32.8, 26.7, 25.7, 24.7, 18.4. **ESI-HR** calcd for $C_{18}H_{26}NO_2^+$ ([M+H]$^+$) 288.1959, found 288.1953. **IR** ν (cm^{-1}) 2935, 1649, 1611, 728.

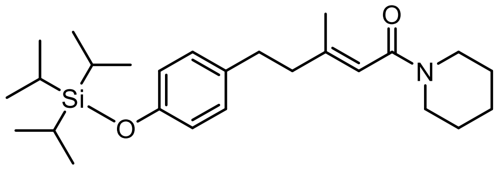

amide 1r: pale-yellow oil. TLC analysis (hexanes/EtOAc 1:1) R_f= 0.53; **^{1}H NMR** (400 MHz, $CDCl_3$) δ 7.03 – 6.96 (m, 2H), 6.81 – 6.75 (m, 2H), 5.66 (q, *J* = 1.3 Hz, 1H), 3.55 (t, *J* = 5.4 Hz, 2H), 3.19 (t, *J* = 5.5 Hz, 2H), 2.72 (dd, *J* = 8.6, 6.7 Hz, 2H), 2.42 – 2.33 (m, 2H), 1.85 (d, *J* = 1.2 Hz, 3H), 1.64 – 1.50 (m, 4H), 1.48 – 1.40 (m, 2H), 1.28 – 1.19 (m, 3H), 1.09 (d, *J* = 7.3 Hz, 18H). **^{13}C NMR** (101 MHz, $CDCl_3$) δ 167.4, 154.3, 145.9, 133.8, 129.3, 119.8, 119.4, 47.4, 42.3, 41.2, 33.0, 26.7, 25.8, 24.8, 18.6, 18.0, 12.8.**ESI-HR** calcd for $C_{26}H_{44}NO_2Si^+$ ([M+H]$^+$) 430.3136, found 430.3131. **IR** ν (cm^{-1}) 2940, 2865, 1649, 1611, 733.

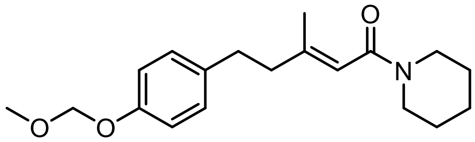

amide 1s: colorless oil. TLC analysis (hexanes/EtOAc 3:1) R_f= 0.25; **^{1}H NMR** (400 MHz, $CDCl_3$) δ 7.09 (d, *J* = 8.4 Hz, 2H), 6.99 – 6.91 (m, 2H), 5.65 (q, *J* = 1.3 Hz, 1H), 5.15 (s, 2H), 3.54 (t, *J* = 5.5 Hz, 2H), 3.47 (s, 3H), 3.15 (t, *J* = 5.6 Hz, 2H), 2.75 (dd, *J* = 8.6, 6.8 Hz, 2H), 2.39 (t, *J* = 7.7 Hz, 2H), 1.85 (d, *J* = 1.2 Hz, 3H), 1.64 – 1.50 (m, 4H), 1.42 (dq, *J* = 11.3, 5.3 Hz, 2H). **^{13}C NMR** (101 MHz, $CDCl_3$) δ 167.4, 155.6, 145.5, 134.9, 129.4, 119.6, 116.3, 94.6, 56.0, 47.3, 42.2, 41.1, 32.9, 26.7, 25.7, 24.7, 18.4. **ESI-HR** calcd for $C_{19}H_{28}NO_3^+$ ([M+H]$^+$) 318.2064, found 318.2059. **IR** ν (cm^{-1}) 2932, 1649, 1616, 998, 828.

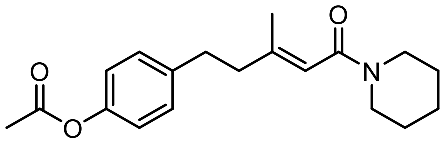

amide 1t: colorless oil. TLC analysis (hexanes/EtOAc 1:1) R_f= 0.31; **^{1}H NMR** (400 MHz, $CDCl_3$) δ 7.22 – 7.13 (m, 2H), 7.06 – 6.95 (m, 2H), 5.66 (q, *J* = 1.3 Hz, 1H), 3.54 (t, *J* = 5.5 Hz, 2H), 3.15 (dd, *J* = 6.6, 4.4 Hz, 2H), 2.80 (dd, *J* = 8.6, 6.8 Hz, 2H), 2.41 (td, *J* = 7.6, 1.2 Hz, 2H), 2.29 (s, 3H), 1.86 (d, *J* = 1.2 Hz, 3H), 1.64 – 1.50 (m, 4H), 1.42 (tq, *J* = 7.6, 5.3, 4.3 Hz, 2H). **^{13}C NMR** (101 MHz, $CDCl_3$) δ 169.7, 167.2, 149.0, 145.3, 139.0, 129.4, 121.5, 119.7, 47.3, 42.2, 40.9, 33.1, 26.7, 25.7, 24.7, 21.2, 18.4. **ESI-HR** calcd for $C_{19}H_{26}NO_3^+$ ([M+H]$^+$) 316.1907, found 316.1901. **IR** ν (cm^{-1}) 2934, 1758, 1648, 1617, 732.

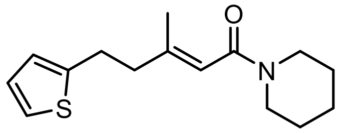

amide 1u: brown oil**.** TLC analysis (hexanes/EtOAc 2:1) R_f= 0.24; **^{1}H NMR** (400 MHz, $CDCl_3$) δ 7.11 (dd, *J* = 5.2, 1.2 Hz, 1H), 6.96 – 6.86 (m, 1H), 6.85 – 6.72 (m, 1H), 5.72 (q, *J* = 1.3 Hz, 1H), 3.55 (t, *J* = 5.5 Hz, 2H), 3.27 – 3.13 (m, 2H), 3.07 – 2.94 (m, 2H), 2.52 – 2.41 (m, 2H), 1.86 (d, *J* = 1.2 Hz, 3H), 1.65 – 1.48 (m, 4H), 1.43 (dp, *J* = 11.3, 5.6, 4.5 Hz, 2H). **^{13}C NMR** (101 MHz, $CDCl_3$) δ 167.2, 144.8, 144.2, 126.8, 124.6, 123.3, 120.0, 47.3, 42.3, 41.2, 27.9, 26.8, 25.8, 24.7, 18.3.. **ESI-HR** calcd for $C_{15}H_{22}NOS^+$ ([M+H]$^+$) 264.1417, found 264.1412. **IR** ν (cm^{-1}) 2938, 1651, 1615, 729.

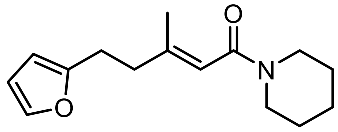

amide 1v: brown oil. TLC analysis (hexanes/EtOAc 1:1) R_f= 0.47; **^{1}H NMR** (400 MHz, $CDCl_3$) δ 7.29 (d, *J* = 2.1 Hz, 1H), 6.26 (q, *J* = 2.6 Hz, 1H), 5.99 (d, *J* = 3.0 Hz, 1H), 5.72 (q, *J* = 1.5 Hz, 1H), 3.55 (dd, *J* = 6.4, 4.0 Hz, 2H), 3.26 (dq, *J* = 5.6, 2.5 Hz, 2H), 2.82 (td, *J* = 7.5, 2.2 Hz, 2H), 2.48 – 2.38 (m, 2H), 1.84 (t, *J* = 1.7 Hz, 3H), 1.68 – 1.39 (m, 6H). **^{13}C NMR** (101 MHz, $CDCl_3$) δ 167.3, 155.1, 145.1, 141.0, 119.6, 110.2,

105.4, 47.3, 42.3, 37.6, 26.7, 26.1, 25.7, 24.7, 18.3. **ESI-HR** calcd for $C_{15}H_{22}NO_2^+$ ($[M+H]^+$) 248.1645, found 248.1641. **IR** ν (cm^{-1}) 2939, 1651, 1606, 724.

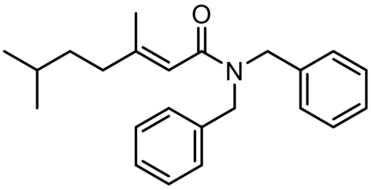

amide 1w: colorless oil. TLC analysis (hexanes/EtOAc 9:1) R_f= 0.32; **^{1}H NMR** (400 MHz, $CDCl_3$) δ 7.39 – 7.13 (m, 10H), 5.92 (q, J = 1.3 Hz, 1H), 4.60 (s, 2H), 4.45 (s, 2H), 2.07 (ddd, J = 9.4, 6.0, 1.2 Hz, 2H), 2.01 (d, J = 1.3 Hz, 3H), 1.46 (dt, J = 13.2, 6.6 Hz, 1H), 1.36 – 1.23 (m, 2H), 0.84 (d, J = 6.6 Hz, 6H). **^{13}C NMR** (101 MHz, $CDCl_3$) δ 169.1, 152.0, 137.7, 137.0, 128.9, 128.6, 128.5, 127.6, 127.4, 126.9, 117.1, 50.5, 47.3, 37.9, 36.7, 27.8, 22.5, 18.9. **ESI-HR** calcd for $C_{23}H_{30}NO^+$ ($[M+H]^+$) 336.2322, found 336.2315. **IR** ν (cm^{-1}) 2955, 1646, 1620, 728.

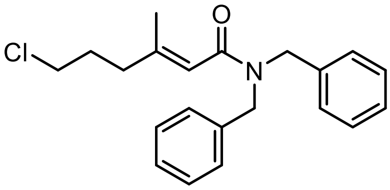

amide 1x: colorless oil. TLC analysis (hexanes/EtOAc 9:1) R_f= 0.20; **^{1}H NMR** (400 MHz, $CDCl_3$) δ 7.45 – 7.04 (m, 10H), 5.98 (t, J = 1.5 Hz, 1H), 4.60 (s, 2H), 4.45 (s, 2H), 3.45 (t, J = 6.4 Hz, 2H), 2.24 (t, J = 7.5 Hz, 2H), 2.02 (d, J = 1.4 Hz, 3H), 1.89 (dq, J = 9.0, 6.5 Hz, 2H). **^{13}C NMR** (101 MHz, $CDCl_3$) δ 168.7, 149.5, 137.5, 136.8, 129.0, 128.7, 128.5, 127.7, 127.5, 126.8, 118.6, 50.5, 47.4, 44.3, 36.9, 30.1, 18.7.**ESI-HR** calcd for $C_{21}H_{25}ClNO^+$ ($[M+H]^+$) 342.1619, found 342.1613. **IR** ν (cm^{-1}) 2915, 1648, 1624, 697.

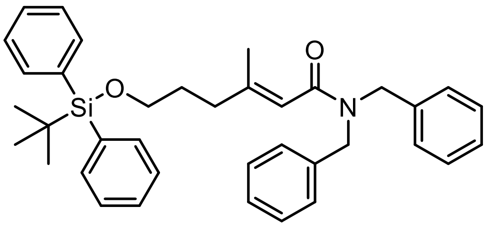

amide 1y: pale-yellow oil. TLC analysis (hexanes/EtOAc 7:1) R_f= 0.35; **^{1}H NMR** (400 MHz, $CDCl_3$) δ 7.66 – 7.57 (m, 4H), 7.43 – 7.21 (m, 14H), 7.17 – 7.07 (m, 2H), 5.90

(q, *J* = 1.3 Hz, 1H), 4.58 (s, 2H), 4.39 (s, 2H), 3.62 (t, *J* = 6.3 Hz, 2H), 2.24 – 2.10 (m, 2H), 1.99 (S, *J* = 1.1 Hz, 3H), 1.71 – 1.58 (m, 2H), 1.01 (s, 9H).**^{13}C NMR** (101 MHz, $CDCl_3$) δ 169.0, 151.2, 137.7, 136.9, 135.7, 134.0, 129.7, 129.0, 128.7, 128.5, 127.8, 127.7, 127.5, 127.0, 117.5, 63.4, 50.5, 47.4, 36.3, 30.6, 27.0, 19.3, 19.0.**ESI-HR** calcd for $C_{37}H_{44}NO_2Si^+$ ($[M+H]^+$) 562.3130, found 562.3131. **IR** ν (cm^{-1}) 2923, 2856, 1626, 1428, 699.

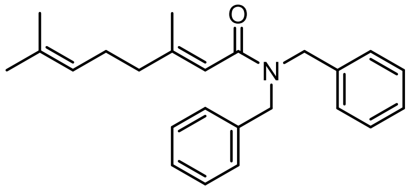

amide 1z: colorless oil. TLC analysis (hexanes/EtOAc 9:1) R_f= 0.39; **^{1}H NMR** (400 MHz, $CDCl_3$) δ 7.38 – 7.12 (m, 10H), 5.91 (q, *J* = 1.3 Hz, 1H), 5.01 (dqd, *J* = 6.1, 3.8, 1.6 Hz, 1H), 4.59 (s, 2H), 4.45 (s, 2H), 2.14 (dd, *J* = 21.8, 2.3 Hz, 4H), 2.01 (d, *J* = 1.2 Hz, 3H), 1.57 (d, *J* = 1.3 Hz, 3H), 1.52 (d, *J* = 1.3 Hz, 3H). **^{13}C NMR** (101 MHz, $CDCl_3$) δ 169.2, 150.7, 137.6, 136.9, 132.3, 128.9, 128.7, 128.5, 127.6, 127.4, 126.9, 123.5, 117.6, 50.4, 47.2, 39.9, 26.0, 25.7, 18.9, 17.8.**ESI-HR** calcd for $C_{24}H_{30}NO^+$ ($[M+H]^+$) 348.2322, found 348.2317. **IR** ν (cm^{-1}) 2914, 1627, 732, 697.

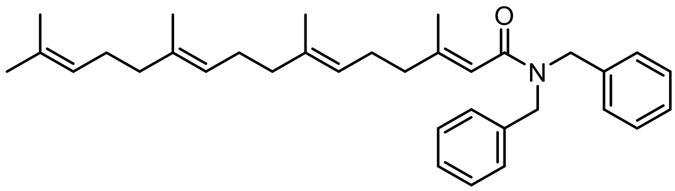

amide 1aa: colorless oil. TLC analysis (hexanes/EtOAc 9:1) R_f= 0.31; **^{1}H NMR** (400 MHz, $CDCl_3$) δ 7.43 – 7.09 (m, 10H), 5.92 (d, *J* = 1.7 Hz, 1H), 5.13 – 4.95 (m, 3H), 4.59 (s, 2H), 4.44 (s, 2H), 2.12 (d, *J* = 3.3 Hz, 4H), 2.10 – 1.84 (m, 11H), 1.67 (d, *J* = 1.5 Hz, 3H), 1.59 (d, *J* = 1.3 Hz, 3H), 1.54 (dd, *J* = 8.7, 1.3 Hz, 6H). **^{13}C NMR** (101 MHz, $CDCl_3$) δ 169.1, 151.0, 137.7, 136.9, 136.1, 135.1, 131.4, 129.0, 128.7, 128.5, 127.7, 127.4, 126.9, 124.5, 124.2, 123.3, 117.5, 50.5, 47.2, 40.0, 39.8, 39.7, 26.9, 26.7, 26.0, 25.8, 19.0, 17.8, 16.2, 16.1. **ESI-HR** calcd for $C_{34}H_{46}NO^+$ ($[M+H]^+$) 484.3574, found 484.3572. **IR** ν (cm^{-1}) 2916, 1648, 1618, 906, 727.

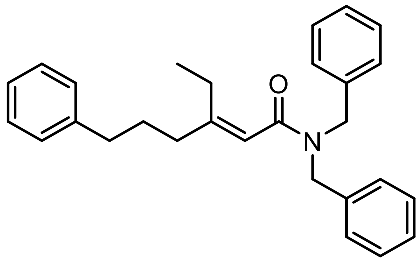

amide 1ab: colorless oil. TLC analysis (hexanes/EtOAc 7:1) R_f = 0.35; **^{1}H NMR** (400 MHz, $CDCl_3$) δ 7.40 – 7.21 (m, 10H), 7.18 – 7.06 (m, 5H), 5.88 (s, 1H), 4.60 (s, 2H), 4.45 (s, 2H), 2.56 (t, J = 7.8 Hz, 2H), 2.44 (q, J = 7.5 Hz, 2H), 2.13 (t, J = 7.7 Hz, 2H), 1.79 – 1.69 (m, 2H), 1.09 (t, J = 7.5 Hz, 3H). **^{13}C NMR** (101 MHz, $CDCl_3$) δ 169.0, 156.2, 142.0, 137.6, 136.9, 129.0, 128.7, 128.6, 128.5, 128.4, 127.7, 127.4, 126.9, 125.9, 117.1, 50.6, 47.3, 36.1, 35.6, 29.4, 25.6, 13.1. **ESI-HR** calcd for $C_{28}H_{32}NO^+$ ($[M+H]^+$) 398.2478, found 398.2472. **IR** ν (cm^{-1}) 3027, 2934, 1626, 1494, 1216, 696.

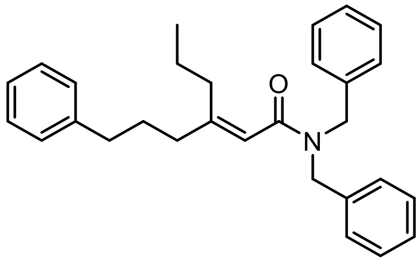

amide 1ac: colorless oil. TLC analysis (hexanes/EtOAc 7:1) R_f = 0.37; **^{1}H NMR** (400 MHz, $CDCl_3$) δ 7.38 – 7.28 (m, 5H), 7.23 (d, *J* = 7.4 Hz, 2H), 7.19 – 7.06 (m, 5H), 5.90 (s, 1H), 4.59 (s, 2H), 4.45 (s, 2H), 2.56 (t, *J* = 7.8 Hz, 2H), 2.46 – 2.35 (m, 2H), 2.12 (t, *J* = 7.7 Hz, 2H), 1.73 (p, *J* = 7.8 Hz, 2H), 1.49 (h, *J* = 7.4 Hz, 2H), 0.94 (t, *J* = 7.3 Hz, 3H). **^{13}C NMR** (101 MHz, $CDCl_3$) δ 169.1, 154.4, 142.1, 137.6, 136.9, 129.0, 128.7, 128.5, 128.4, 127.7, 127.5, 126.9, 125.9, 117.8, 50.6, 47.3, 36.5, 35.6, 34.4, 29.4, 21.8, 14.4. **ESI-HR** calcd for $C_{29}H_{34}NO^+$ ($[M+H]^+$) 412.2635, found 412.2627. **IR** ν (cm^{-1}) 2945, 1676, 1641, 1144, 710.

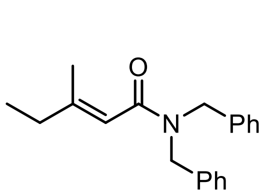

amide 1ad: colorless oil. TLC analysis (hexanes/EtOAc 11:1) R_f = 0.23; **^{1}H NMR** (400 MHz, $CDCl_3$) δ 7.40 – 7.12 (m, 10H), 5.92 (p, J = 1.3 Hz, 1H), 4.60 (s, 2H), 4.45 (s, 2H), 2.10 (q, J = 7.4 Hz, 2H), 2.02 (d, J = 1.1 Hz, 3H), 1.01 (t, J = 7.4 Hz, 3H). **^{13}C NMR** (101 MHz, $CDCl_3$) δ 169.2, 152.8, 137.6, 137.0, 128.9, 128.6, 128.4, 127.6, 127.4, 126.9, 116.3, 50.5, 47.3, 32.7, 18.9, 12.1. **ESI-HR** calcd for $C_{20}H_{24}NO^+$ ($[M+H]^+$) 294.1852, found 294.1847. **IR** ν (cm^{-1}) 2966, 1625, 1494, 1224, 697.

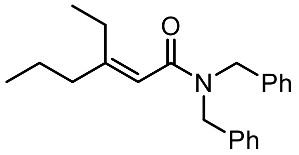

amide 1ae: colorless oil. TLC analysis (hexanes/EtOAc 9:1) R_f = 0.53; **^{1}H NMR** (400 MHz, $CDCl_3$) δ 7.55 – 6.95 (m, 10H), 5.87 (s, 1H), 4.59 (s, 2H), 4.45 (s, 2H), 2.43 (q, J = 7.5 Hz, 2H), 2.15 – 2.03 (m, 2H), 1.42 (dt, J = 14.8, 7.4 Hz, 2H), 1.10 (t, J = 7.5 Hz, 3H), 0.86 (t, J = 7.3 Hz, 3H). **^{13}C NMR** (101 MHz, $CDCl_3$) δ 169.0, 156.6, 137.6, 136.9, 128.9, 128.6, 128.4, 127.6, 127.4, 126.9, 116.9, 50.5, 47.2, 38.7, 25.6, 20.9, 13.9, 13.1. **ESI-HR** calcd for $C_{22}H_{28}NO^+$ ($[M+H]^+$) 322.2165, found 322.2158. **IR** ν (cm^{-1}) 2959, 1626, 1494, 1359, 1215, 697.

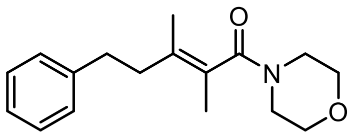

amide 1af: pale yellow oil. TLC analysis (hexanes/EtOAc 2:1) R_f = 0.30; **^{1}H NMR** (400 MHz, $CDCl_3$) δ 7.48 – 6.92 (m, 5H), 3.62 (t, J = 7.4 Hz, 4H), 3.50 – 3.40 (m, 2H), 3.02 – 2.95 (m, 2H), 2.88 – 2.49 (m, 3H), 2.28 (s, 1H), 1.67 (d, J = 2.3 Hz, 6H). **^{13}C NMR** (101 MHz, $CDCl_3$) δ 172.2, 141.5, 132.7, 128.6, 128.4, 126.5, 126.1, 67.2, 66.9, 46.3, 41.4, 34.2, 33.5, 19.3, 15.8. **ESI-HR** calcd for $C_{17}H_{24}NO_2^+$ ($[M+H]^+$) 274.1802, found 274.1795. **IR** ν (cm^{-1}) 2854, 1626, 1496, 1426, 1229, 1121, 700.

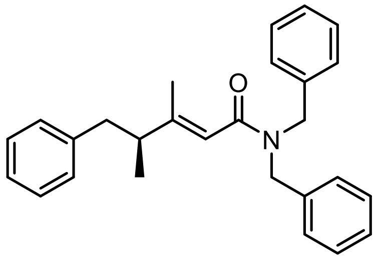

amide 1ag: colorless oil. TLC analysis (hexanes/EtOAc 11:1) R_f= 0.26; **^{1}H NMR** (400 MHz, $CDCl_3$) δ 7.37 – 6.98 (m, 15H), 5.74 (s, 1H), 4.63 (d, J = 14.8 Hz, 1H), 4.42 (d, J = 14.8 Hz, 1H), 4.15 – 3.96 (m, 2H), 2.69 (dd, J = 13.4, 8.0 Hz, 1H), 2.58 (dd, J = 13.5, 6.8 Hz, 1H), 2.50 (p, J = 6.9 Hz, 1H), 1.97 (d, J = 1.3 Hz, 3H), 1.05 (d, J = 6.8 Hz, 3H).**^{13}C NMR** (101 MHz, $CDCl_3$) δ 169.0, 153.0, 140.4, 137.5, 136.9, 128.9, 128.8, 128.6, 128.3, 128.3, 127.5, 127.3, 126.9, 126.0, 118.1, 50.2, 47.1, 45.1, 41.4, 18.8, 15.6. **ESI-HR** calcd for $C_{27}H_{30}NO^+$ ($[M+H]^+$) 384.2322, found 384.2313. **IR** ν (cm^{-1}) 3019, 1618, 1265, 1215, 748, 703. $[\alpha]^{20}_D$ = + 48.9 (*c* = 0.70, $CHCl_3$)

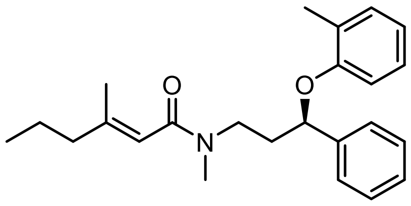

amide 1ah: colorless oil. Two sets of peaks were observed in **^{1}H** and **^{13}C NMR** spectra due to carbamate rotamers, which are assigned to major and minor rotamers, respectively. TLC analysis (hexanes/EtOAc 2:1) R_f= 0.38; **^{1}H NMR** (400 MHz, $CDCl_3$) δ 7.46 – 7.19 (m, 5H, *overlapping*), 7.15 – 7.07 (m, 1H, *overlapping*), 6.93 (t, J = 7.8 Hz, 1H, *overlapping*), 6.76 (td, J = 7.3, 4.5 Hz, 1H, *overlapping*), 6.58 (d, J = 8.2 Hz, 1H, *minor*), 6.53 (d, J = 8.2 Hz, 1H, *major*), 5.78 – 5.74 (m, 1H, *minor*), 5.71 – 5.66 (m, 1H, *major*), 5.19 (dd, J = 8.4, 4.6 Hz, 1H, *minor*), 5.13 (dd, J = 8.1, 4.2 Hz, 1H, *major*), 3.72 – 3.58 (m, 2H, *minor*), 3.46 (m, 2H, *major*), 2.95 (s, 3H, *overlapping*), 2.34 (s, 3H, *overlapping*), 2.15 (m, 3H, *overlapping*), 1.90 – 1.82 (m, 4H, *overlapping*), 1.48 (p, J = 7.4 Hz, 2H, *minor*), 1.34 (h, J = 7.3 Hz, 2H, *major*), 0.91 (t, J = 7.3 Hz, 3H, *minor*), 0.84 (t, J = 7.3 Hz, 3H, *major*). **^{13}C NMR** (101 MHz, $CDCl_3$) δ 168.7 (*major*), 168.5 (*minor*), 155.9 (*minor*), 155.5 (*major*), 150.8 (*major*), 149.4 (*minor*), 141.8 (*minor*),

141.3 (*major*), 130.7 (*major*), 130.7 (*minor*), 128.8 (*major*), 128.7 (*minor*), 127.8 (*major*), 127.6 (*minor*), 127.0 (*minor*), 126.7 (*major*), 126.7 (*major*), 126.6 (*minor*), 125.8, (*minor*) 125.6 (*major*), 120.5 (*major*), 120.3 (*minor*), 117.9 (*minor*), 116.9 (*major*), 112.8 (*major*), 112.5 (*minor*), 77.7 (*minor*), 76.3 (*major*), 46.9 (*major*), 44.8 (*minor*), 41.9 (*overlapping*), 37.5 (*major*), 36.6 (*minor*), 36.3 (*minor*), 32.8 (*major*), 20.7 (*overlapping*), 18.5 (*major*), 18.4 (*minor*), 16.6 (*major*), 16.6 (*minor*), 13.8 (*major*), 13.8 (*minor*). **ESI-HR** calcd for $C_{24}H_{32}NO_2^+$ ($[M+H]^+$) 366.2428, found 366.2420. **IR** ν (cm^{-1}) 2957, 2931, 1650, 1625, 1492, 1238, 750, 701. $[\alpha]^{20}_D$ = - 22.3 (*c* = 6.00, $CHCl_3$)

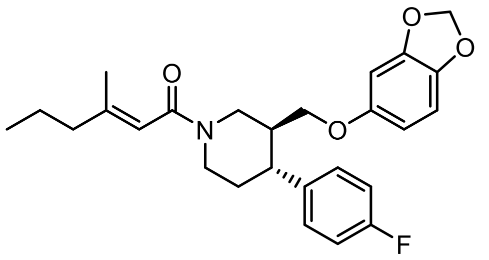

amide 1ai: colorless oil. Two sets of peaks were observed in **^{1}H**, **^{13}C** and **^{19}F NMR** spectra due to carbamate rotamers, which are assigned to major and minor rotamers, respectively. TLC analysis (hexanes/EtOAc 2:1) R_f= 0.32; **^{1}H NMR** (400 MHz, $CDCl_3$) δ 7.20 – 7.06 (m, 2H, *overlapping*), 6.98 (q, J = 7.7, 7.3 Hz, 2H, *overlapping*), 6.63 (dd, J = 8.4, 2.1 Hz, 1H, *overlapping*), 6.34 (dd, J = 17.7, 2.5 Hz, 1H, *overlapping*), 6.13 (ddd, J = 14.0, 8.4, 2.6 Hz, 1H, *overlapping*), 5.88 (d, J = 2.4 Hz, 2H, *overlapping*), 5.83 (s, 1H, *overlapping*), 4.95 (m, 1H, *minor*), 4.89 – 4.73 (m, 1H, *major*), 4.32 (d, J = 13.3 Hz, 1H, *major*), 4.08 (d, J = 13.3 Hz, 1H, *minor*), 3.62 (ddd, J = 21.0, 9.6, 2.9 Hz, 1H, *overlapping*), 3.53 – 3.38 (m, 1H, *overlapping*), 3.17 – 2.99 (m, 1H, *overlapping*), 2.89 – 2.63 (m, 2H, *overlapping*), 2.17 – 1.98 (m, 3H, *overlapping*), 1.95 – 1.83 (m, 4H, *overlapping*), 1.71 (qd, J = 13.8, 13.1, 4.3 Hz, 1H, *overlapping*), 1.52 (p, J = 7.4 Hz, 2H, *overlapping*), 0.94 (q, J = 7.2 Hz, 3H, *overlapping*). **^{13}C NMR** (101 MHz, $CDCl_3$) δ 168.7 (*major*), 168.5 (*minor*), 155.9 (*minor*), 155.5 (*major*), 150.8 (*major*), 149.4 (*minor*), 141.8 (*minor*), 141.3 (*major*), 130.7 (*major*), 130.7 (*minor*),

128.8 (*major*), 128.7 (*minor*), 127.8 (*major*), 127.6 (*minor*), 127.0 (*minor*), 126.7 (*major*), 126.7 (*major*), 126.6 (*minor*), 125.8, (*minor*) 125.6 (*major*), 120.5 (*major*), 120.3 (*minor*), 117.9 (*minor*), 116.9 (*major*), 112.8 (*major*), 112.5 (*minor*), 77.7 (*minor*), 76.3 (*major*), 46.9 (*major*), 44.8 (*minor*), 41.9 (*overlapping*), 37.5 (*major*), 36.6 (*minor*), 36.3 (*minor*), 32.8 (*major*), 20.7 (*overlapping*), 18.5 (*major*), 18.4 (*minor*), 16.6 (*major*), 16.6 (*minor*), 13.8 (*major*), 13.8 (*minor*). 13C NMR (101 MHz, CDCl3) δ 167.6(*overlapping*), 161.8 (J_1 = 246.9 Hz) (*overlapping*), 154.4 (*minor*), 154.0 (*major*), 148.9 (*minor*), 148.6 (*major*), 148.3 (*overlapping*), 141.9 (*major*), 141.8 (*minor*), 138.8 (*overlapping*), 128.8 (J_3 = 7.4 Hz) (*overlapping*), 117.9 (*overlapping*), 115.7 (J_3 =22.9 Hz) (*overlapping*), 107.9 (*overlapping*), 105.7 (*minor*), 105.5 (*major*), 101.3 (*overlapping*), 98.1 (*minor*), 97.9 (*major*), 68.8 (*overlapping*), 49.9 (*major*), 46.9 (*minor*), 44.7 (*minor*), 44.6 (*major*), 44.0 (*minor*), 42.9 (*major*), 42.0 (*minor*), 41.9 (*major*), 41.8 (*minor*), 41.7 (*major*), 34.8 (*minor*), 33.9 (*major*), 20.7 (*overlapping*), 18.6 (*overlapping*), 13.9 (*overlapping*),. **^{19}F NMR** (376 MHz, CDCl3) δ -115.8 (*major*), -116.0 (*minor*). **ESI-HR** calcd for $C_{26}H_{31}FNO_4^+$ ($[M+H]^+$) 440.2232, found 440.2226. **IR** ν (cm^{-1}) 3015, 2961, 1607, 1510, 1183, 741. $[\alpha]^{20}_D$ = + 2.7 (*c* = 2.94, $CHCl_3$)

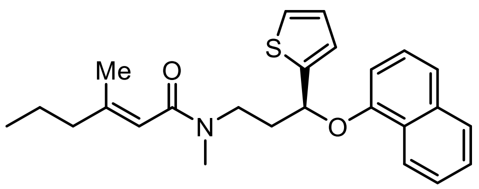

amide 1aj: white solid. Two sets of peaks were observed in **^{1}H** and **^{13}C NMR** spectra due to carbamate rotamers, which are assigned to major and minor rotamers, respectively. TLC analysis (hexanes/EtOAc 2:1) R_f= 0.33; **^{1}H NMR** (400 MHz, $CDCl_3$) δ 8.46 – 8.23 (m, 1H, *overlapping*), 7.77 (td, J = 5.5, 5.0, 2.6 Hz, 1H, *overlapping*), 7.49 (dq, J = 7.4, 3.8 Hz, 2H, *overlapping*), 7.38 (dd, J = 8.3, 4.4 Hz, 1H, *overlapping*), 7.31 – 7.15 (m, 2H, *overlapping*), 7.10 (d, J = 3.5 Hz, 1H, *minor*), 7.04 (d, J = 3.5 Hz, 1H, *major*), 6.92 (td, J = 5.2, 3.5 Hz, 1H, *overlapping*), 6.84 (d, J = 7.7 Hz, 1H, *minor*), 6.77

(d, J = 7.7 Hz, 1H, *major*), 5.79 – 5.53 (m, 2H, *overlapping*), 3.76 (ddd, J = 12.8, 10.4, 5.8 Hz, 1H, *overlapping*), 3.60 – 3.45 (m, 1H, *overlapping*), 2.97 (s, 3H, *overlapping*), 2.61 – 2.29 (m, 2H, *overlapping*), 2.05 (t, J = 7.6 Hz, 1H, *overlapping*), 1.88 (s, 3H, *minor*), 1.80 (s, 3H, *major*), 1.64 (td, J = 7.3, 4.4 Hz, 1H, *overlapping*), 1.47 (h, J = 7.3 Hz, 1H, *overlapping*), 1.18 (h, J = 7.5 Hz, 1H, *overlapping*), 0.90 (t, J = 7.4 Hz, 3H, *minor*), 0.68 (t, J = 7.3 Hz, 3H, *major*). **^{13}C NMR** (101 MHz, $CDCl_3$) δ 168.9 (*major*), 168.7 (*minor*), 153.2 (*minor*), 152.8 (*major*), 151.0 (*major*), 149.8 (*minor*), 144.9 (*minor*), 144.4 (*major*), 134.7 (*major*), 134.7 (*minor*), 127.7 (*major*), 127.6 (*minor*), 126.8 (*major*), 126.7 (*minor*), 126.5 (*major*), 126.4 (*minor*), 126.2 (*minor*), 126.0 (*major*), 125.8 (*minor*), 125.8 (*major*), 125.5 (*major*), 125.4 (*minor*), 125.0 (*major*), 125.0 (*minor*), 124.9 (*minor*), 124.8 (*major*), 122.2 (*minor*), 121.9 (*major*), 121.0 (*major*), 120.8 (*minor*), 117.8 (*minor*), 116.8 (*major*), 107.1 (*minor*), 106.8 (*major*), 74.7 (*minor*), 73.0 (*major*), 46.7 (*major*), 44.7 (*minor*), 42.0 (*minor*), 41.8 (*major*), 37.8 (*major*), 36.8 (*minor*), 36.5 (*minor*), 32.9 (*major*), 20.7 (*minor*), 20.6 (*major*), 18.6 (*major*), 18.5 (*minor*), 13.8 (*minor*), 13.8 (*major*). **ESI-HR** calcd for $C_{25}H_{30}NO_2S^+$ ($[M+H]^+$) 408.1992, found 408.1987. **IR** ν (cm^{-1}) 3018, 2962, 1610, 1470, 1214, 745. **M. P.** 100-102 °C. $[\alpha]^{20}_D$ = + 142.2 (*c* = 0.59, $CHCl_3$)

Characterization of Products

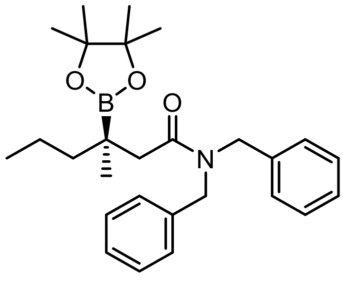

product 3a: Following the general procedure, amide **1a** (76.8 mg, 0.25 mmol) was converted to the boration product. Purification by silica gel chromatography gave 94.8 mg (87% yield) of **3a** as a white solid. TLC analysis (hexanes/EtOAc 11:1) R_f= 0.34; **^{1}H NMR**(400 MHz, $CDCl_3$) δ 7.50 – 7.06 (m, 10H), 4.78 (d, *J* = 14.7 Hz, 1H), 4.45 (d, *J* = 16.9 Hz, 1H), 4.31 (d, *J* = 15.7 Hz, 2H), 2.64 (d, *J* = 16.6 Hz, 1H), 2.30 (d, *J* = 16.6

Hz, 1H), 1.29 (m, J = 6.6 Hz, 16H), 1.02 (s, 3H), 0.87 (t, J = 6.9 Hz, 3H). **^{13}C NMR** (101 MHz, $CDCl_3$) δ 174.1, 137.4, 136.4, 129.0, 128.6, 128.5, 127.7, 127.4, 126.5, 82.5, 49.6, 48.2, 44.2, 41.2, 25.2, 25.1, 21.7, 18.4, 15.2. **ESI-HR** calcd for $C_{27}H_{39}BNO_3^+$ ($[M+H]^+$) 436.3018, found 436.3007. **IR** ν (cm^{-1}) 2957, 1644, 1142, 751. **M. P.** 71-73 ºC. $[\alpha]^{20}_D$ = + 56.3 (c = 0.36, $CHCl_3$) for a 96% *ee* sample. The enantiomeric purity of this compound was determined by HPLC analysis in comparison with authentic racemic material (Chiralpak AD, 99.5:0.5 hexanes:*i*-PrOH, 1.0 mL/min, 208 nm): t_{major} =6.579 min, t_{minor} =7.906 min.

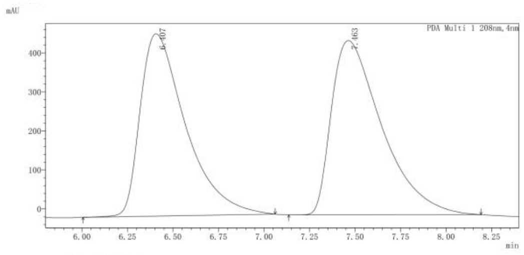

PDA Ch1 208nm

峰号	保留时间	峰宽(高度 50%)	高度	面积	面积%
1	6.407	0.258	467284	8110946	48.516
2	7.463	0.292	446396	8607168	51.484

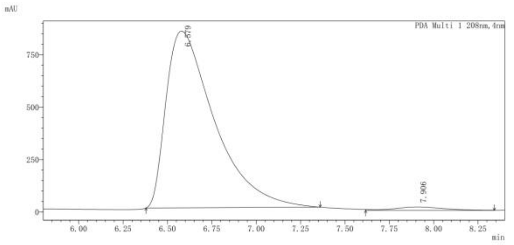

PDA Ch1 208nm

峰号	保留时间	峰宽(高度 50%)	高度	面积	面积%
1	6.579	0.283	843179	16024935	98.093
2	7.906	0.289	15974	311557	1.907

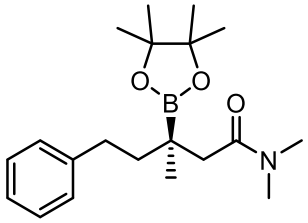

product 3b: Following the general procedure, amide **1b** (54.3 mg, 0.25 mmol) was converted to the boration product. Purification by silica gel chromatography gave 46.5 mg (54% yield) of **3b** as a white solid. TLC analysis (hexanes/EtOAc 1:2) R_f= 0.36; **^{1}H NMR**(400 MHz, $CDCl_3$) δ 7.41 – 7.06 (m, 5H), 2.95 (d, J = 1.6 Hz, 6H), 2.73 – 2.55 (m, 2H), 2.50 (d, J = 16.6 Hz, 1H), 2.25 (d, J = 16.6 Hz, 1H), 1.77 – 1.69 (m, 1H), 1.65 – 1.54 (m, 1H), 1.25 (d, J = 8.3 Hz, 12H), 1.05 (s, 3H).**^{13}C NMR** (101 MHz, $CDCl_3$) 13C NMR (101 MHz, CDCl3) δ 174.7, 143.9, 128.5, 128.3, 125.5, 81.9, 44.4, 41.1, 37.3, 35.9, 32.0, 25.3, 25.2, 22.2. **ESI-HR** calcd for $C_{20}H_{33}BNO_3^+$ ($[M+H]^+$) 346.2548, found 346.2539. **IR** ν (cm^{-1}) 2982, 2927, 1637, 1140, 699. **M. P.** 88-90 ºC. $[\alpha]^{20}_D$ = + 32.5 (c = 0.43, $CHCl_3$) for a 93% *ee* sample. The enantiomeric purity of this compound was determined by HPLC analysis in comparison with authentic racemic material

(Chiralpak OD, 98.0:2.0 hexanes:*i*-PrOH, 1.0 mL/min, 208 nm): t_{major} =7.034 min, t_{minor} =6.005 min.

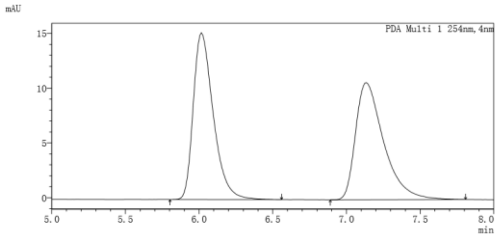

<Peak Results>
PDA Ch1 254nm

Index	Time/min	Height/mAU	Quantity/Area	Area %/%
1	6.016	15227	143565	50.311
2	7.133	10691	141793	49.689

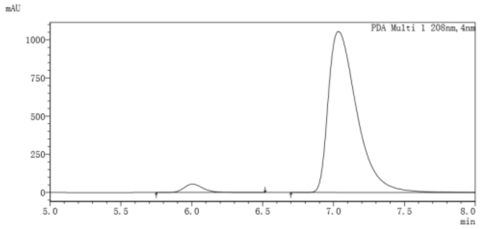

<Peak Results>
PDA Ch1 208nm

Index	Time/min	Height/mAU	Quantity/Area	Area %/%
1	6.005	54994	521139	3.406
2	7.034	1055245	14781060	96.594

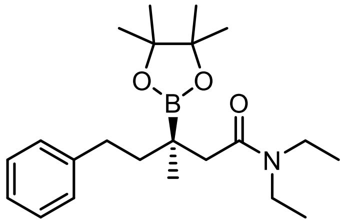

product 3c: Following the general procedure, amide **1c** (61.3 mg, 0.25 mmol) was converted to the boration product. Purification by silica gel chromatography gave 62.1 mg (67% yield) of **3c** as a colorless oil. TLC analysis (hexanes/EtOAc 2:1) R_f = 0.38; **^{1}H NMR**(400 MHz, $CDCl_3$) δ 7.36 – 7.10 (m, 5H), 3.46 (dq, *J* = 14.2, 7.2 Hz, 1H), 3.38 – 3.21 (m, 3H), 2.69 (td, *J* = 12.8, 5.0 Hz, 1H), 2.63 – 2.43 (m, 2H), 2.25 (d, *J* = 16.6 Hz, 1H), 1.78 – 1.56 (m, 2H), 1.23 (d, *J* = 6.7 Hz, 12H), 1.14 (dt, *J* = 14.2, 7.2 Hz, 6H), 1.03 (s, 3H). **^{13}C NMR** (101 MHz, $CDCl_3$) δ 175.0, 144.1, 128.6, 128.3, 125.5, 81.3, 44.2, 42.4, 41.3, 41.0, 32.0, 25.4, 25.3, 22.2, 14.1, 13.0. **ESI-HR** calcd for $C_{22}H_{37}BNO_3^+$ ($[M+H]^+$) 374.2861, found 374.2854. **IR** ν (cm^{-1}) 2972, 2933, 1632, 1140, 671. $[\alpha]^{20}_{D}$ = + 16.3 (*c* = 0.52, $CHCl_3$) for a 95% *ee* sample. The enantiomeric purity of this compound was determined by HPLC analysis in comparison with authentic racemic material (Chiralpak OD, 99.7:0.3 hexanes:*i*-PrOH, 1.0 mL/min, 208 nm): t_{major} =12.201 min, t_{minor} =9.825 min.

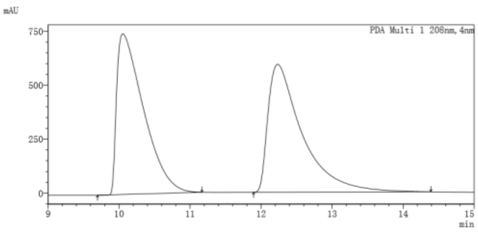

<Peak Results>
PDA Ch1 208nm

Index	Time/min	Height/mAU	Quantity/Area	Area %/%
1	10.054	744214	19533746	49.619
2	12.233	592745	19833937	50.381

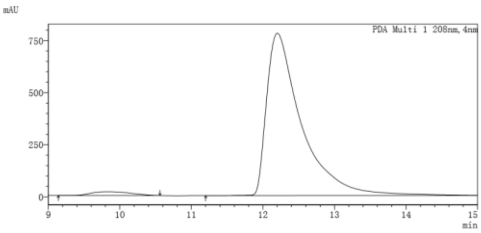

<Peak Results>
PDA Ch1 208nm

Index	Time/min	Height/mAU	Quantity/Area	Area %/%
1	9.825	17967	689781	2.555
2	12.201	778404	26310846	97.445

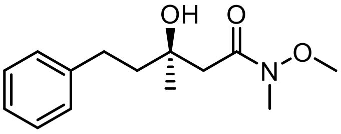

product 3d-ol: Following the general procedure with amide **1d** (58.3mg, 0.25 mmol). After the reaction, the residue was dissolved in THF (2.0 mL), H_2O (2.0 mL), then sodium perborate trihydrate (4.0 equiv) was added. The reaction was stirred at room temperature for 12 h. Ethyl acetate (20 mL) was added, and the mixture was washed with water (10 mL), brine (10 mL) and dried over Na_2SO_4. After removal of the solvent, the residue was purified by column chromatography on silica gel with EtOAc/hexanes mixture as eluent to give 34.3 mg (55% yield) of **3d-ol** as a colorless oil. TLC analysis (hexanes/EtOAc 2:1) R_f= 0.35; **^{1}H NMR** (400 MHz, $CDCl_3$) δ 7.37 – 7.12 (m, 5H), 4.83 (s, 1H), 3.67 (s, 3H), 3.20 (s, 3H), 2.83 – 2.55 (m, 4H), 1.95 – 1.75 (m, 2H), 1.32 (s, 3H). **^{13}C NMR** (101 MHz, $CDCl_3$) δ 174.0, 142.7, 128.5, 128.5, 125.8, 71.2, 61.5, 44.2, 40.7, 31.8, 30.4, 27.1. **ESI-HR** calcd for $C_{14}H_{22}NO_3^+$ ([M+H]$^+$) 252.1594, found 252.1591. **IR** ν (cm^{-1}) 3432, 2936, 1634, 1455, 746. $[\alpha]^{20}_D$ = + 78.7 (*c* = 0.71, $CHCl_3$) for a 93% *ee* sample. The enantiomeric purity of this compound was determined by HPLC analysis in comparison with authentic racemic material (Chiralpak AD, 90.0:10.0 hexanes:*i*-PrOH, 1.0 mL/min, 208 nm): t_{major} = 8.772 min, t_{minor} =8.172 min.

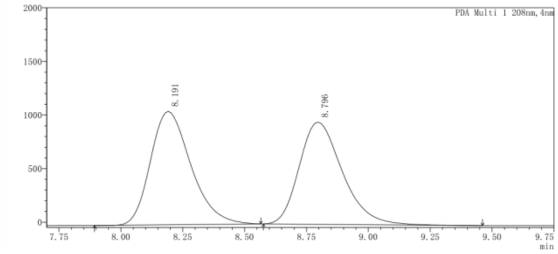

PDA Ch1 208nm

峰号	保留时间	峰宽(高度 50%)	高度	面积	面积%
1	8.191	0.174	1057472	12036305	51.042
2	8.796	0.185	952030	11544880	48.958

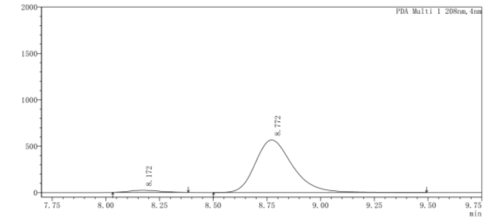

PDA Ch1 208nm

峰号	保留时间	峰宽(高度 50%)	高度	面积	面积%
1	8.172	0.158	23414	231984	3.275
2	8.772	0.182	566565	6850694	96.725

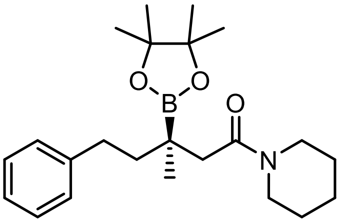

product 3e: Following the general procedure, amide **1e** (64.3 mg, 0.25 mmol) was converted to the boration product. Purification by silica gel chromatography gave 81.7 mg (85% yield) of **3e** as a white solid. TLC analysis (hexanes/EtOAc 1:1) R_f = 0.44; **^{1}H NMR** (400 MHz, $CDCl_3$) δ 7.26 – 7.11 (m, 5H), 3.57 (qt, *J* = 13.1, 5.4 Hz, 2H), 3.35 (q, *J* = 5.9 Hz, 2H), 2.70 (td, *J* = 12.8, 4.9 Hz, 1H), 2.63 – 2.42 (m, 2H), 2.27 (d, *J* = 16.6 Hz, 1H), 1.77 – 1.45 (m, 8H), 1.23 (d, *J* = 6.8 Hz, 12H), 1.04 (s, 3H). **^{13}C NMR** (101 MHz, $CDCl_3$) δ 173.9, 144.1, 128.6, 128.3, 125.5, 81.3, 46.8, 44.1, 41.1, 32.1, 26.3, 25.6, 25.4, 25.3, 24.3, 22.4. **ESI-HR** calcd for $C_{23}H_{37}BNO_3^+$ ($[M+H]^+$) 386.2861, found 386.2849. **IR** ν (cm^{-1}) 2939, 2858, 1620, 1445, 1139, 730. **M. P.** 109-111 ^{o}C. $[\alpha]^{20}_{D}$ = + 12.3 (*c* = 0.96, $CHCl_3$) for a 96% *ee* sample. The enantiomeric purity of this compound was determined by HPLC analysis in comparison with authentic racemic material (Chiralpak OD, 99.2:0.8 hexanes:*i*-PrOH, 1.0 mL/min, 208 nm): t_{major} = 9.930 min, t_{minor} =7.229 min.

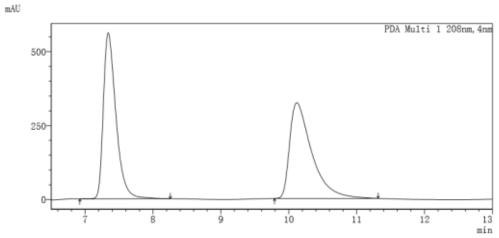

<Peak Results>
PDA Ch1 208nm

Index	Time/min	Height/mAU	Quantity/Area	Area %/%
1	7.341	561146	7234399	49.668
2	10.118	323311	7331102	50.332

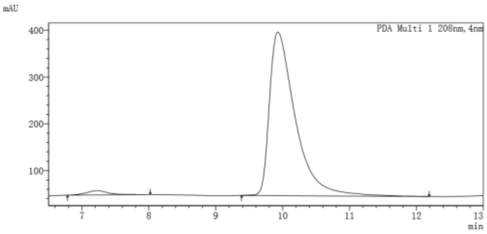

<Peak Results>
PDA Ch1 208nm

Index	Time/min	Height/mAU	Quantity/Area	Area %/%
1	7.229	9020	210211	2.188
2	9.930	349728	9395551	97.812

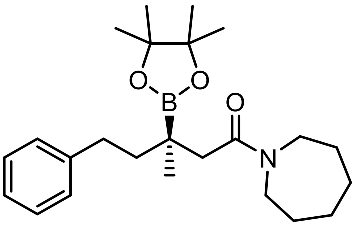

product 3f: Following the general procedure, amide **1f** (67.8 mg, 0.25 mmol) was converted to the boration product. Purification by silica gel chromatography gave 72.1

mg (65% yield) of **3f** as a white solid. TLC analysis (hexanes/EtOAc 1:1) R_f = 0.35; **^{1}H NMR** (400 MHz, $CDCl_3$) δ 7.32 – 7.06 (m, 5H), 3.60 – 3.50 (m, 2H), 3.42 – 3.32 (m, 2H), 2.69 (td, *J* = 12.8, 5.0 Hz, 1H), 2.62 – 2.47 (m, 2H), 2.27 (d, *J* = 16.7 Hz, 1H), 1.79 – 1.47 (m, 10H), 1.23 (d, *J* = 7.1 Hz, 12H), 1.04 (s, 3H). **^{13}C NMR** (101 MHz, $CDCl_3$) δ 175.5, 144.1, 128.6, 128.3, 125.4, 81.3, 48.2, 46.9, 44.4, 41.0, 32.0, 28.8, 27.3, 27.1, 27.0, 25.4, 25.3, 22.2. **ESI-HR** calcd for $C_{24}H_{39}BNO_3^+$ ($[M+H]^+$) 400.3018, found 400.3009. **IR** ν (cm^{-1}) 2927, 2863, 1632, 1145, 699. **M. P.** 75-77 °C. $[\alpha]^{20}_D$ = + 13.0 (*c* = 0.67, $CHCl_3$) for a 95% *ee* sample. The enantiomeric purity of this compound was determined by HPLC analysis in comparison with authentic racemic material (Chiralpak OD, 99.5:0.5 hexanes:*i*-PrOH, 1.0 mL/min, 208 nm): t_{major} = 13.732 min, t_{minor} =9.480 min.

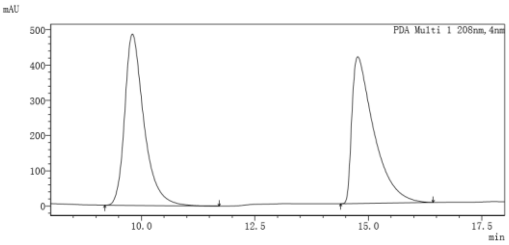

<Peak Results>
PDA Ch1 208nm

Index	Time/min	Height/mAU	Quantity/Area	Area %/%
1	9.802	486162	14303968	50.463
2	14.765	416235	14041285	49.537

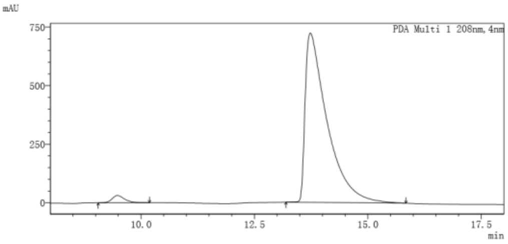

<Peak Results>
PDA Ch1 208nm

Index	Time/min	Height/mAU	Quantity/Area	Area %/%
1	9.480	31172	610269	2.449
2	13.732	723011	24304110	97.551

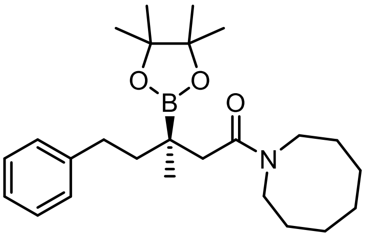

product 3g: Following the general procedure, amide **1g** (71.3 mg, 0.25 mmol) was converted to the boration product. Purification by silica gel chromatography gave 62.0 mg (60% yield) of **3g** as a white solid. TLC analysis (hexanes/EtOAc 11:1) R_f = 0.38; **^{1}H NMR** (400 MHz, $CDCl_3$) δ 7.31 – 7.07 (m, 5), 3.48 (t, *J* = 6.0 Hz, 2H), 3.34 (t, *J* = 5.8 Hz, 2H), 2.69 (td, *J* = 12.8, 5.0 Hz, 1H), 2.63 – 2.47 (m, 2H), 2.28 (d, *J* = 16.6 Hz, 1H), 1.82 – 1.45 (m, 12H), 1.23 (d, *J* = 7.8 Hz, 12H), 1.05 (s, 3H). **^{13}C NMR** (101 MHz, $CDCl_3$) δ 175.6, 144.1, 128.6, 128.3, 125.4, 81.2, 49.4, 48.0, 44.6, 40.8, 32.0, 26.9, 26.8, 25.8, 25.7, 25.4, 25.3, 24.9, 22.2. **ESI-HR** calcd for $C_{25}H_{41}BNO_3^+$ ($[M+H]^+$) 414.3174,

found 414.3163. **IR** ν (cm^{-1}) 2925, 1632, 1145, 699. **M. P.** 103-105 °C. $[\alpha]^{20}_D$ = + 9.5 (c = 0.42, $CHCl_3$) for a 95% *ee* sample. The enantiomeric purity of this compound was determined by HPLC analysis in comparison with authentic racemic material (Chiralpak IC, 99.0:1.0 hexanes:*i*-PrOH, 0.5 mL/min, 208 nm): t_{major} = 22.021 min, t_{minor} =20.975 min.

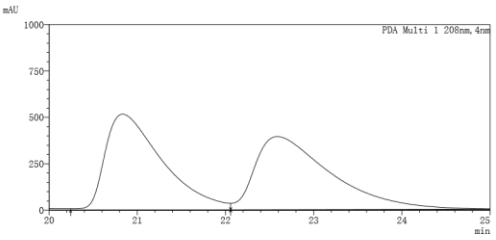

<Peak Results>
PDA Ch1 208nm

Index	Time/min	Height/mAU	Quantity/Area	Area %/%
1	20.835	516522	23360896	49.858
2	22.590	394188	23493999	50.142

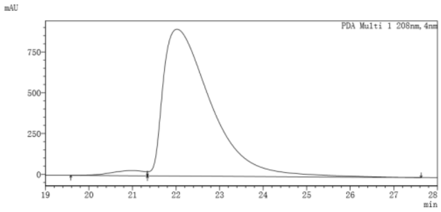

<Peak Results>
PDA Ch1 208nm

Index	Time/min	Height/mAU	Quantity/Area	Area %/%
1	20.975	31194	1641912	2.335
2	22.021	899699	68668125	97.665

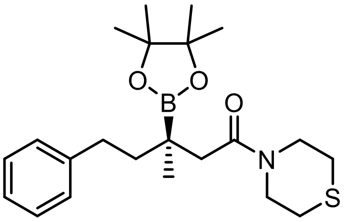

product 3h: Following the general procedure, amide **1h** (68.8 mg, 0.25 mmol) was converted to the boration product. Purification by silica gel chromatography gave 52.1 mg (52% yield) of **3h** as a white solid. TLC analysis (hexanes/EtOAc 3:1) R_f= 0.37; **^{1}H NMR** (400 MHz, $CDCl_3$) δ 7.30 – 7.12 (m, 5H), 3.85 (dddd, J = 24.0, 13.5, 10.6, 5.1 Hz, 2H), 3.68 (q, J = 4.9 Hz, 2H), 2.73 – 2.55 (m, 6H), 2.48 (d, J = 16.4 Hz, 1H), 2.21 (d, J = 16.4 Hz, 1H), 1.74 (ddd, J = 13.3, 10.7, 6.4 Hz, 1H), 1.58 (ddd, J = 13.4, 11.1, 6.7 Hz, 1H), 1.26 (d, J = 10.2 Hz, 12H), 1.06 (s, 3H). **^{13}C NMR** (101 MHz, $CDCl_3$) δ 172.1, 143.7, 128.5, 128.4, 125.6, 82.5, 48.2, 44.9, 44.2, 41.2, 32.0, 27.8, 27.6, 25.2, 25.1, 22.1. **ESI-HR** calcd for $C_{22}H_{35}BNO_3S^+$ ($[M+H]^+$) 404.2425, found 404.2416. **IR** ν (cm^{-1}) 2922, 1639, 1146, 700. **M. P.** 120-122 °C. $[\alpha]^{20}_D$ = + 10.0 (c =1.33, $CHCl_3$) for a 99% *ee* sample. The enantiomeric purity of this compound was determined by HPLC analysis in comparison with authentic racemic material (Chiralpak AD, 99.6:0.4 hexanes:*i*-PrOH, 1.0 mL/min, 208 nm): t_{major} =15.877 min, t_{minor} =20.994 min.

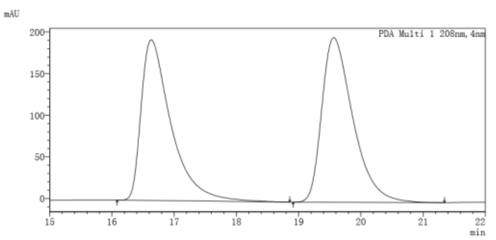

<Peak Results>
PDA Ch1 208nm

Index	Time/min	Height/mAU	Quantity/Area	Area %/%
1	16.632	193035	6444118	48.359
2	19.565	197821	6881401	51.641

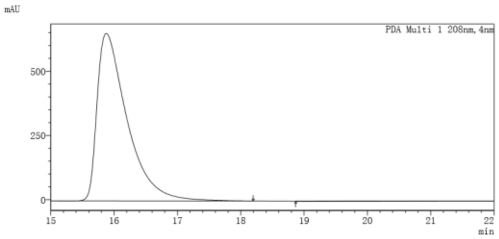

<Peak Results>
PDA Ch1 208nm

Index	Time/min	Height/mAU	Quantity/Area	Area %/%
1	15.877	651537	21404696	99.613
2	20.994	587	83119	0.387

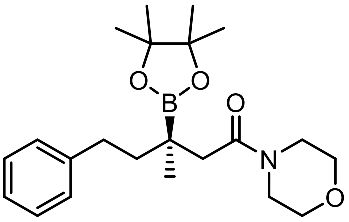

product (*S*)-3i: Following the general procedure, amide **1i** (64.8 mg, 0.25 mmol) was converted to the boration product. Purification by silica gel chromatography gave 62.7 mg (65% yield) of **(*S*)-3i** as a white solid. TLC analysis (hexanes/EtOAc 2:1) $R_f = 0.23$; **^{1}H NMR** (400 MHz, $CDCl_3$) δ 7.29 – 7.12 (m, 5H), 3.71 – 3.54 (m, 6H), 3.41 (q, *J* = 4.8 Hz, 2H), 2.63 (tt, *J* = 10.4, 7.3 Hz, 2H), 2.49 (d, *J* = 16.4 Hz, 1H), 2.23 (d, *J* = 16.4 Hz, 1H), 1.74 (ddd, *J* = 13.3, 10.6, 6.5 Hz, 1H), 1.62 – 1.54 (m, 1H), 1.27 (d, *J* = 9.8 Hz, 12H), 1.06 (s, 3H). **^{13}C NMR** (101 MHz, $CDCl_3$) δ 172.2, 143.7, 128.5, 128.4, 125.6, 82.6, 67.0, 66.6, 45.9, 43.8, 42.4, 41.3, 32.0, 25.2, 25.1, 22.1. **ESI-HR** calcd for $C_{22}H_{35}BNO_4^+$ ($[M+H]^+$) 388.2654, found 388.2642. **IR** ν (cm^{-1}) 2970, 2856, 1638, 1114, 700. **M. P.** 130-132 °C. $[\alpha]^{20}_D$ = + 11.7 (*c* = 0.42, $CHCl_3$) for a 92% *ee* sample. The enantiomeric purity of this compound was determined by HPLC analysis in comparison with authentic racemic material (Chiralpak AD, 99.0:1.0 hexanes:*i*-PrOH, 1.0 mL/min, 208 nm): t_{major} = 11.969 min, t_{minor} =9.958 min.

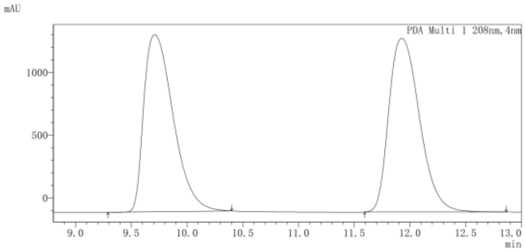

<Peak Results>
PDA Ch1 208nm

Index	Time/min	Height/mAU	Quantity/Area	Area %/%
1	9.710	1409752	25786618	49.334
2	11.926	1383030	26483345	50.666

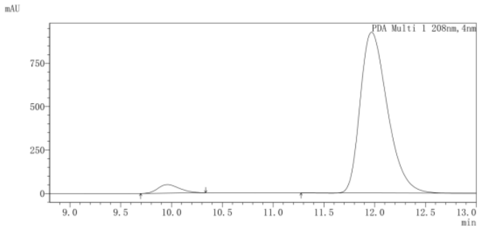

<Peak Results>
PDA Ch1 208nm

Index	Time/min	Height/mAU	Quantity/Area	Area %/%
1	9.958	49722	741617	4.123
2	11.969	927042	17243549	95.877

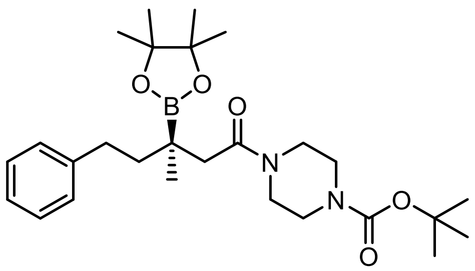

product 3j: Following the general procedure, amide **1j** (89.5 mg, 0.25 mmol) was converted to the boration product. Purification by silica gel chromatography gave 85.5 mg (70% yield) of **3j** as a white solid. TLC analysis (hexanes/EtOAc 2:1) R_f = 0.32; **^{1}H NMR** (400 MHz, $CDCl_3$) δ 7.29 – 7.22 (m, 2H), 7.21 – 7.11 (m, 3H), 3.57 (q, *J* = 5.8 Hz, 2H), 3.39 (dt, *J* = 11.0, 6.0 Hz, 6H), 2.63 (tt, *J* = 10.3, 7.4 Hz, 2H), 2.51 (d, *J* = 16.4 Hz, 1H), 2.24 (d, *J* = 16.4 Hz, 1H), 1.74 (ddd, *J* = 13.4, 10.6, 6.5 Hz, 1H), 1.58 (ddd, *J* = 13.3, 10.9, 6.8 Hz, 1H), 1.47 (s, 9H), 1.26 (d, *J* = 10.2 Hz, 12H), 1.06 (s, 3H). **^{13}C NMR** (101 MHz, $CDCl_3$) δ 172.2, 154.7, 143.6, 128.5, 128.4, 125.6, 82.6, 80.4, 45.3, 44.0, 41.9, 41.2, 32.0, 28.5, 25.2, 25.1, 22.1. **ESI-HR** calcd for $C_{27}H_{44}BN_2O_5^+$ ($[M+H]^+$) 487.3338, found 487.3338. **IR** ν (cm^{-1}) 2975, 2929, 1644, 1147, 707. **M. P.** 136-138 ^{o}C. $[\alpha]^{20}_{D}$ = + 13.2 (*c* = 0.65, $CHCl_3$) for a 92% *ee* sample. The enantiomeric purity of this compound was determined by HPLC analysis in comparison with authentic racemic material (Chiralpak AD, 98.0:2.0 hexanes:*i*-PrOH, 1.0 mL/min, 208 nm): t_{major} = 10.235 min, t_{minor} =11.583 min.

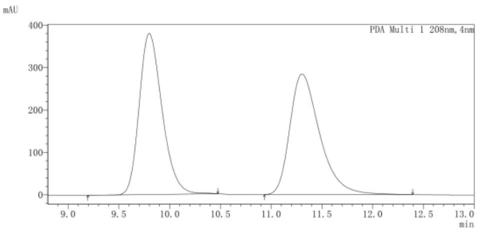

<Peak Results>
PDA Ch1 208nm

Index	Time/min	Height/mAU	Quantity/Area	Area %/%
1	9.799	379738	5945787	50.554
2	11.304	284338	5815386	49.446

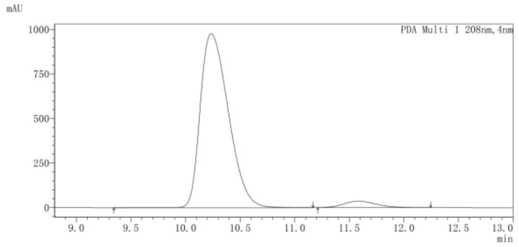

<Peak Results>
PDA Ch1 208nm

Index	Time/min	Height/mAU	Quantity/Area	Area %/%
1	10.235	976246	17477196	95.990
2	11.583	35681	730152	4.010

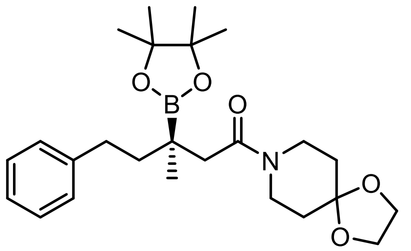

product 3k: Following the general procedure, amide **1k** (78.8 mg, 0.25 mmol) was converted to the boration product. Purification by silica gel chromatography gave 95.2 mg (86% yield) of **3k** as a white solid. TLC analysis (hexanes/EtOAc 3:1) R_f = 0.23; **^{1}H NMR** (400 MHz, $CDCl_3$) δ 7.30 – 7.11 (m, 5H), 3.97 (s, 4H), 3.70 (ddt, *J* = 25.3, 13.4, 6.4 Hz, 2H), 3.49 (q, *J* = 5.8 Hz, 2H), 2.69 – 2.46 (m, 3H), 2.27 (d, *J* = 16.4 Hz, 1H), 1.76 – 1.54 (m, 6H), 1.25 (d, *J* = 9.2 Hz, 12H), 1.05 (s, 3H). **^{13}C NMR** (101 MHz, $CDCl_3$) δ 172.7, 143.8, 128.5, 128.3, 125.6, 106.8, 82.0, 64.6, 44.0, 43.6, 41.2, 40.7, 35.5, 34.9, 32.0, 25.3, 25.2, 22.2. **ESI-HR** calcd for $C_{25}H_{39}BNO_5^+$ ($[M+H]^+$) 444.2916, found 444.2903. **IR** ν (cm^{-1}) 3445, 3025, 2971, 2929, 1632, 1032, 699. **M. P.** 105-108 °C. $[\alpha]^{20}_D$ = + 16.7 (*c* = 0.42, $CHCl_3$) for a 93% *ee* sample. The enantiomeric purity of this compound was determined by HPLC analysis in comparison with authentic racemic material (Chiralpak OD, 99.0:1.0 hexanes:*i*-PrOH, 1.0 mL/min, 208 nm): t_{major} = 19.564 min, t_{minor} =17.486 min.

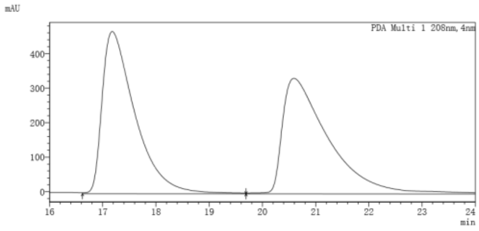

<Peak Results>
PDA Ch1 208nm

Index	Time/min	Height/mAU	Quantity/Area	Area %/%
1	17.178	469559	20194312	49.963
2	20.592	334608	20224077	50.037

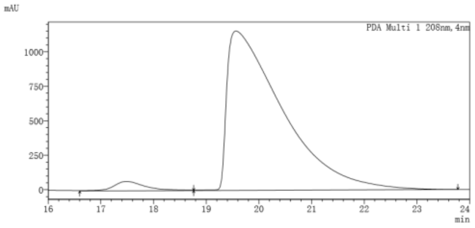

<Peak Results>
PDA Ch1 208nm

Index	Time/min	Height/mAU	Quantity/Area	Area %/%
1	17.486	68232	2868485	3.269
2	19.564	1155503	84875711	96.731

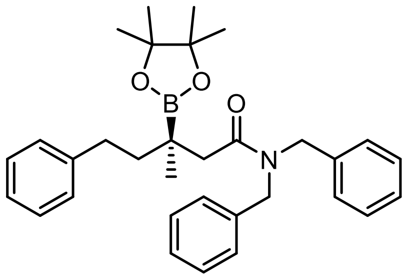

product 3l: Following the general procedure, amide **1l** (92.3 mg, 0.25 mmol) was converted to the boration product. Purification by silica gel chromatography gave 115.9 mg (93% yield) of **3l** as a white solid. TLC analysis (hexanes/EtOAc 4:1) R_f = 0.52; **^{1}H NMR** (400 MHz, $CDCl_3$) δ 7.38 – 7.18 (m, 10H), 7.17 – 7.10 (m, 5H), 4.81 (d, *J* = 14.7 Hz, 1H), 4.44 (d, *J* = 17.1 Hz, 1H), 4.35 – 4.28 (m, 2H), 2.68 (d, *J* = 16.6 Hz, 1H), 2.59 (dq, *J* = 12.7, 7.5, 6.3 Hz, 2H), 2.36 (d, *J* = 16.5 Hz, 1H), 1.82 – 1.70 (m, 1H), 1.59

(ddd, J = 13.4, 11.3, 6.5 Hz, 1H), 1.31 (d, J = 8.2 Hz, 12H), 1.12 (s, 3H). **^{13}C NMR** (101 MHz, $CDCl_3$) δ 174.1, 143.7, 137.3, 136.3, 129.1, 128.6, 128.5, 128.5, 128.3, 127.8, 127.5, 126.6, 125.6, 82.6, 49.7, 48.4, 44.3, 41.1, 31.9, 25.3, 25.2, 21.8. **ESI-HR** calcd for $C_{32}H_{41}BNO_3^+$ ($[M+H]^+$) 498.3174, found 498.3166. **IR** ν (cm^{-1}) 2973, 2927, 1642, 1145, 698. **M. P.** 119-121 °C. $[\alpha]^{20}_D$ = + 44.7 (c = 0.54, $CHCl_3$) for a 95% *ee* sample. The enantiomeric purity of this compound was determined by HPLC analysis in comparison with authentic racemic material (Chiralpak AD, 99.0:1.0 hexanes:*i*-PrOH, 1.0 mL/min, 208 nm): t_{major} = 13.156 min, t_{minor} =11.542 min.

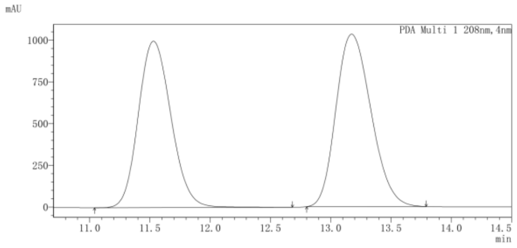

<Peak Results>
PDA Ch1 208nm

Index	Time/min	Height/mAU	Quantity/Area	Area %/%
1	11.529	997451	18836650	47.381
2	13.173	1033959	20919007	52.619

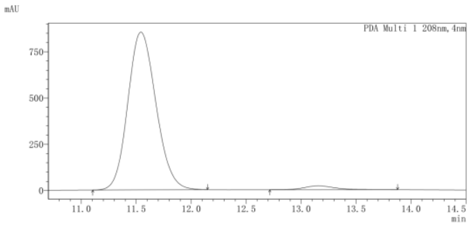

<Peak Results>
PDA Ch1 208nm

Index	Time/min	Height/mAU	Quantity/Area	Area %/%
1	11.542	853045	15200857	97.467
2	13.156	20571	395058	2.533

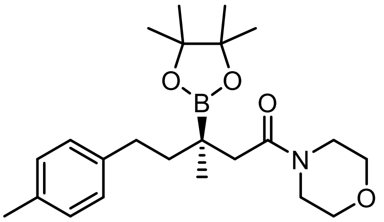

product 3m: Following the general procedure, amide **1m** (68.3 mg, 0.25 mmol) was converted to the boration product. Purification by silica gel chromatography gave 77.9 mg (77% yield) of **3m** as a white solid. TLC analysis (hexanes/EtOAc 1:1) R_f= 0.28; **^{1}H NMR** (400 MHz, $CDCl_3$) δ 7.07 (s, 4H), 3.71 – 3.56 (m, 6H), 3.42 (m, J = 5.1 Hz, 2H), 2.59 (ddd, J = 9.9, 6.1, 2.4 Hz, 2H), 2.49 (d, J = 16.4 Hz, 1H), 2.31 (s, 3H), 2.22 (d, J = 16.4 Hz, 1H), 1.71 (ddd, J = 13.3, 10.3, 7.0 Hz, 1H), 1.55 (ddd, J = 13.3, 10.8, 7.0 Hz, 1H), 1.26 (d, J = 10.6 Hz, 12H), 1.06 (s, 3H). **^{13}C NMR** (101 MHz, $CDCl_3$) δ 172.2, 140.5, 135.1, 129.1, 128.4, 82.5, 67.0, 66.6, 45.9, 43.8, 42.4, 41.5, 31.5, 25.2, 25.1, 22.1, 21.1. **ESI-HR** calcd for $C_{23}H_{37}BNO_4^+$ ($[M+H]^+$) 402.2810, found 402.2797. **IR** ν (cm^{-1}) 2972, 2924, 2858, 1643, 1147, 858. **M. P.** 131-133 °C. $[\alpha]^{20}_D$ = + 10.0 (c = 0.45, $CHCl_3$) for a 97% *ee* sample. The enantiomeric purity of this compound was determined

by HPLC analysis in comparison with authentic racemic material (Chiralpak OD, 99.0:1.0 hexanes:*i*-PrOH, 1.0 mL/min, 208 nm): t_{major} = 13.718 min, t_{minor} =11.815 min.

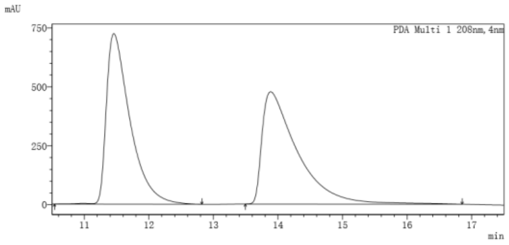

<Peak Results>
PDA Ch1 208nm

Index	Time/min	Height/mAU	Quantity/Area	Area %/%
1	11.457	723176	18374699	50.365
2	13.886	476050	18108022	49.635

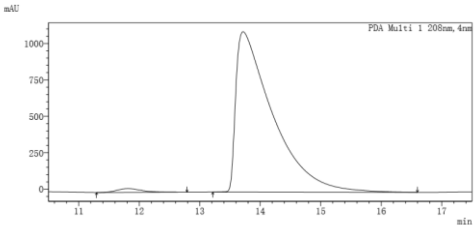

<Peak Results>
PDA Ch1 208nm

Index	Time/min	Height/mAU	Quantity/Area	Area %/%
1	11.815	25383	732991	1.545
2	13.718	1100407	46722259	98.455

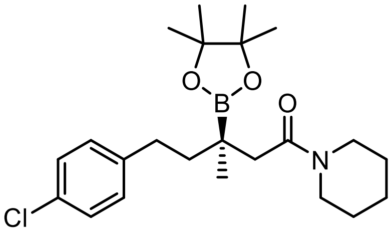

product 3n: Following the general procedure, amide **1n** (72.9 mg, 0.25 mmol) was converted to the boration product. Purification by silica gel chromatography gave 65.5 mg (62% yield) of **3n** as a white solid. TLC analysis (hexanes/EtOAc 2:1) R_f = 0.25; **^{1}H NMR** (400 MHz, $CDCl_3$) δ 7.23 (d, *J* = 8.0 Hz, 2H), 7.13 (d, *J* = 8.0 Hz, 2H), 3.59 (q, *J* = 5.8 Hz, 2H), 3.38 (q, *J* = 4.9 Hz, 2H), 2.69 (td, *J* = 12.9, 4.9 Hz, 1H), 2.63 – 2.43 (m, 2H), 2.30 (d, *J* = 16.6 Hz, 1H), 1.63 (ddt, *J* = 41.2, 12.9, 5.9 Hz, 8H), 1.25 (d, *J* = 5.3 Hz, 12H), 1.04 (s, 3H). **^{13}C NMR** (101 MHz, $CDCl_3$) δ 173.9, 142.6, 131.1, 129.9, 128.4, 81.3, 46.8, 44.2, 41.1, 31.5, 26.3, 25.6, 25.4, 25.3, 24.3, 22.4. **ESI-HR** calcd for $C_{23}H_{36}BClNO_3^+$ ($[M+H]^+$) 420.2471, found 420.2464. **IR** ν (cm^{-1}) 3417, 2976, 2939, 1617, 1138, 884. **M. P.** 96-98 ^{o}C. $[\alpha]^{20}_{D}$ = + 11.8 (*c* = 0.47, $CHCl_3$) for a 95% *ee* sample. The enantiomeric purity of this compound was determined by HPLC analysis in comparison with authentic racemic material (Chiralpak AD, 99.6:0.4 hexanes:*i*-PrOH, 1.0 mL/min, 208 nm): t_{major} = 10.490 min, t_{minor} =13.102 min.

Single crystal suitable for X-ray analysis was obtained through slow diffusion of hexane into a solution of **3n** in dichloromethane (CCDC number 2031005).

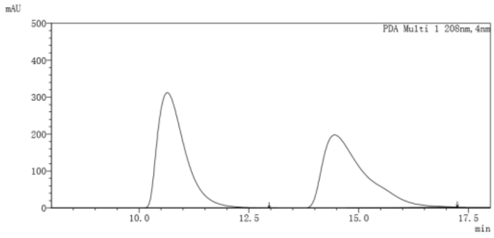

<Peak Results>
PDA Ch1 208nm

Index	Time/min	Height/mAU	Quantity/Area	Area %/%
1	10.643	312936	13619863	50.944
2	14.454	198118	13114981	49.056

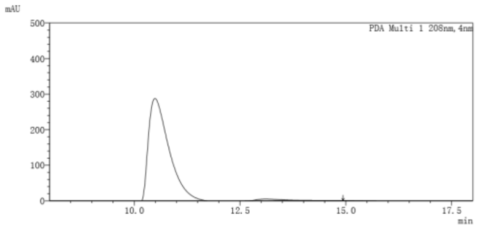

<Peak Results>
PDA Ch1 208nm

Index	Time/min	Height/mAU	Quantity/Area	Area %/%
1	10.490	293437	10090971	97.333
2	13.102	5615	276470	2.667

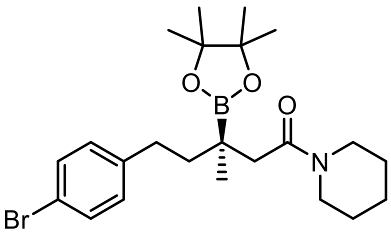

product 3o: Following the general procedure, amide **1o** (83.8 mg, 0.25 mmol) was converted to the boration product. Purification by silica gel chromatography gave 72.1 mg (62% yield) of **3o** as a white solid. TLC analysis (hexanes/EtOAc 2:1) R_f = 0.27; **^{1}H NMR** (400 MHz, $CDCl_3$) δ 7.43 – 7.28 (m, 2H), 7.15 – 6.99 (m, 2H), 3.57 (q, *J* = 5.7 Hz, 2H), 3.35 (q, *J* = 5.0 Hz, 2H), 2.66 (td, *J* = 12.9, 4.8 Hz, 1H), 2.53 (dt, *J* = 13.0, 6.5 Hz, 1H), 2.47 (d, *J* = 16.7 Hz, 1H), 2.28 (d, *J* = 16.7 Hz, 1H), 1.76 – 1.47 (m, 8H), 1.22 (d, *J* = 5.1 Hz, 12H), 1.02 (s, 3H). **^{13}C NMR** (101 MHz, $CDCl_3$) δ 173.9, 143.1, 131.3, 130.4, 119.1, 81.3, 46.8, 44.2, 41.1, 31.5, 26.3, 25.6, 25.4, 25.3, 24.3, 22.4. **ESI-HR** calcd for $C_{23}H_{36}BBrNO_3^+$ ($[M+H]^+$) 464.1966, found 464.1954. **IR** ν (cm^{-1}) 3416, 2975, 2937, 1616, 1139, 855. **M. P.** 93-95 °C. $[\alpha]^{20}_D$ = + 10.9 (*c* = 0.46, $CHCl_3$) for a 99% *ee* sample. The enantiomeric purity of this compound was determined by HPLC analysis in comparison with authentic racemic material (Chiralpak AD, 99.5:0.5 hexanes:*i*-PrOH, 1.0 mL/min, 208 nm): t_{major} =9.068 min, t_{minor} =11.668 min.

Single crystal suitable for X-ray analysis was obtained through slow diffusion of hexane into a solution of **3o** in dichloromethane (CCDC number 2031762).

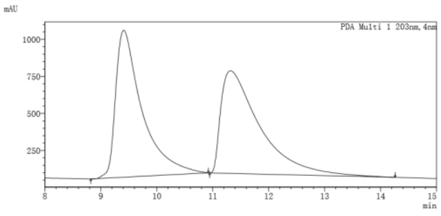

<Peak Results>
PDA Ch1 203nm

Index	Time/min	Height/mAU	Quantity/Area	Area %/%
1	9.409	991764	32557884	49.248
2	11.319	692875	33551575	50.752

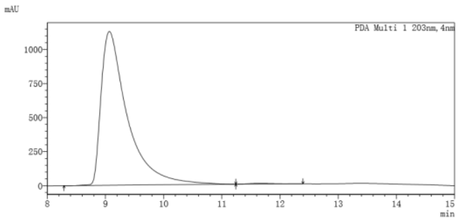

<Peak Results>
PDA Ch1 203nm

Index	Time/min	Height/mAU	Quantity/Area	Area %/%
1	9.068	1130349	35502821	99.569
2	11.668	4546	153571	0.431

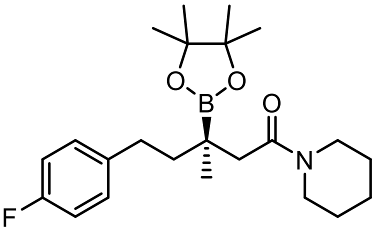

product 3p: Following the general procedure, amide **1p** (68.8 mg, 0.25 mmol) was converted to the boration product. Purification by silica gel chromatography gave 66.5 mg (66% yield) of **3p** as a white solid. TLC analysis (hexanes/EtOAc 2:1) R_f = 0.36; **^{1}H NMR** (400 MHz, $CDCl_3$) δ 7.16 – 7.09 (m, 2H), 6.97 – 6.88 (m, 2H), 3.58 (ddt, *J* = 15.9, 13.2, 6.6 Hz, 2H), 3.36 (q, *J* = 5.1 Hz, 2H), 2.67 (td, *J* = 12.9, 4.9 Hz, 1H), 2.55 (dt, *J* = 13.1, 6.6 Hz, 1H), 2.48 (d, *J* = 16.6 Hz, 1H), 2.27 (d, *J* = 16.7 Hz, 1H), 1.74 – 1.48 (m, 8H), 1.23 (d, *J* = 5.7 Hz, 12H), 1.02 (s, 3H). **^{13}C NMR** δ 173.9, 161.2(J_1 = 242.1 Hz), 139.7(J_4= 3.0 Hz), 129.8(J_3= 7.8 Hz), 115.0(J_2 = 21.2 Hz), 81.3, 46.8, 44.2, 41.3, 31.3, 26.3, 25.6, 25.4, 25.3, 24.3, 22.4. **^{19}F NMR** (376 MHz, CDCl3) δ -118.6. **ESI-HR** calcd for $C_{23}H_{36}BFNO_3^+$ ($[M+H]^+$) 404.2767, found 404.2764. **IR** ν (cm^{-1}) 3425, 2974, 2937, 1618, 1140, 856. **M. P.** 55-58 °C. $[\alpha]^{20}_D$ = + 11.4 (*c* = 0.47, $CHCl_3$) for a 98% *ee* sample. The enantiomeric purity of this compound was determined by HPLC analysis in comparison with authentic racemic material (Chiralpak AD, 99.6:0.4 hexanes:*i*-PrOH, 1.0 mL/min, 208 nm): t_{major} =10.063 min, t_{minor} =13.392 min.

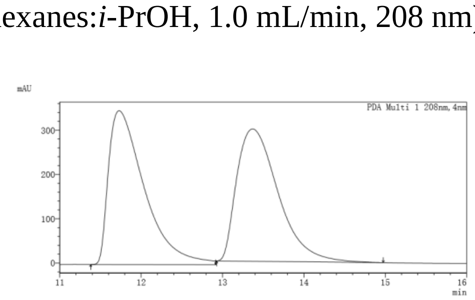

<Peak Results>
PDA Ch1 208nm

Index	Time/min	Height/mAU	Quantity/Area	Area %/%
1	11.730	347462	10676911	50.184
2	13.370	298783	10598417	49.816

<Chromatogram>

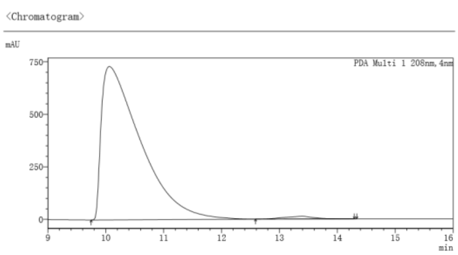

<Peak Results>
PDA Ch1 208nm

Index	Time/min	Height/mAU	Quantity/Area	Area %/%
1	10.063	729800	34579124	98.757
2	13.392	12298	435330	1.243

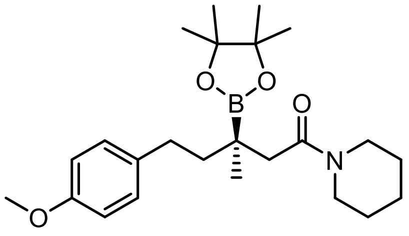

product 3q: Following the general procedure, amide **1q** (71.8 mg, 0.25 mmol) was converted to the boration product. Purification by silica gel chromatography gave 70.7 mg (68% yield) of **3q** as a white solid. TLC analysis (hexanes/EtOAc 3:2) R_f = 0.31; **^{1}H NMR** (400 MHz, $CDCl_3$) δ 7.14 – 7.06 (m, 2H), 6.84 – 6.78 (m, 2H), 3.77 (s, 3H), 3.61 – 3.50 (m, 2H), 3.35 (q, *J* = 5.6 Hz, 2H), 2.63 (td, *J* = 12.8, 4.9 Hz, 1H), 2.57 – 2.39 (m, 2H), 2.26 (d, *J* = 16.6 Hz, 1H), 1.73 – 1.47 (m, 8H), 1.23 (d, *J* = 6.9 Hz, 12H), 1.02 (s, 3H). **^{13}C NMR** (101 MHz, $CDCl_3$) δ 173.8, 157.6, 136.2, 129.3, 129.3, 113.8, 113.7, 81.3, 55.4, 46.8, 44.1, 41.3, 31.0, 26.2, 25.6, 25.4, 25.3, 24.3, 22.4. **ESI-HR** calcd for $C_{24}H_{39}BNO_4^+$ ($[M+H]^+$) 416.2967, found 416.2957. **IR** ν (cm^{-1}) 3418, 2935, 1613, 1139. 855, 667. **M. P.** 82-85 °C. $[\alpha]^{20}_D$ = + 16.4 (*c* = 0.53, $CHCl_3$) for a 94% *ee* sample. The enantiomeric purity of this compound was determined by HPLC analysis in comparison with authentic racemic material (Chiralpak AD, 99.0:1.0 hexanes:*i*-PrOH, 1.0 mL/min, 208 nm): t_{major} = 9.745 min, t_{minor} =10.816 min.

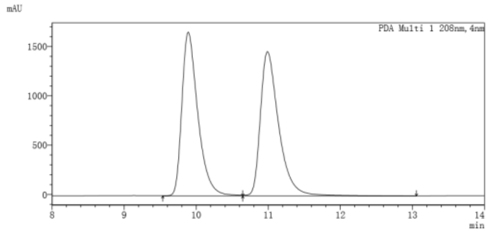

<Peak Results>
PDA Ch1 208nm

Index	Time/min	Height/mAU	Quantity/Area	Area %/%
1	9.889	1661052	25639704	49.690
2	10.988	1463830	25959604	50.310

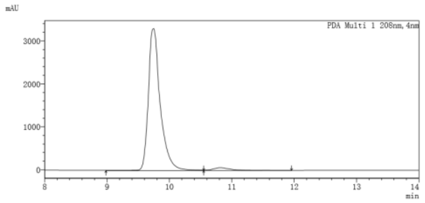

<Peak Results>
PDA Ch1 208nm

Index	Time/min	Height/mAU	Quantity/Area	Area %/%
1	9.745	3305832	44089447	96.844
2	10.816	65961	1436608	3.156

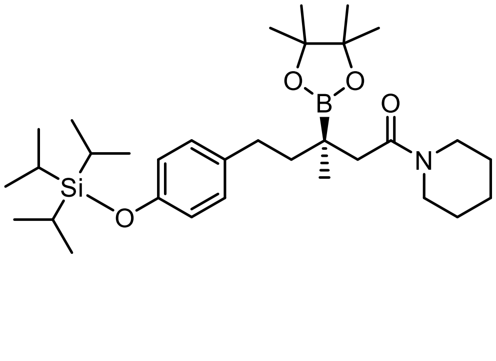

product 3r: Following the general procedure, amide **1r** (107.3 mg, 0.25 mmol) was converted to the boration product. Purification by silica gel chromatography gave 95.0 mg (68% yield) of **3r** as a colorless oil. TLC analysis (hexanes/EtOAc 4:1) R_f= 0.24; **^{1}H NMR** (400 MHz, $CDCl_3$) δ 7.01 (d, J = 8.3 Hz, 2H), 6.81 – 6.71 (m, 2H), 3.57 (ddq, J = 19.5, 13.2, 7.5, 6.5 Hz, 2H), 3.35 (q, J = 5.9 Hz, 2H), 2.69 – 2.44 (m, 3H), 2.25 (d, J = 16.6 Hz, 1H), 1.73 – 1.50 (m, 8H), 1.23 (m, J = 6.8 Hz, 15H), 1.09 (d, J = 7.2 Hz, 18H), 1.02 (s, 3H). **^{13}C NMR** (101 MHz, $CDCl_3$) δ 173.9, 153.8, 136.4, 129.2, 119.6, 81.3, 46.8, 44.1, 41.1, 31.1, 26.2, 25.6, 25.4, 25.3, 24.3, 22.3, 18.1, 12.8. **IR** ν (cm^{-1}) 2945, 2865, 1633, 1254, 1140, 882, 678. **ESI-HR** calcd for $C_{32}H_{57}BNO_4Si^+$ ($[M+H]^+$) 558.4144, found 558.4139. $[\alpha]^{20}_D$ = + 12.8 (*c* = 0.56, $CHCl_3$) for a 96% *ee* sample. The enantiomeric purity of this compound was determined by HPLC analysis in comparison with authentic racemic material (Chiralpak AD, 99.7:0.3 hexanes:*i*-PrOH, 1.0 mL/min, 208 nm): t_{major} = 7.397 min, t_{minor} =9.269 min.

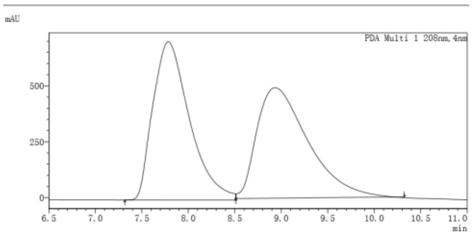

<Peak Results>
PDA Ch1 208nm

Index	Time/min	Height/mAU	Quantity/Area	Area %/%
1	7.785	708116	19433764	50.623
2	8.934	494646	18955318	49.377

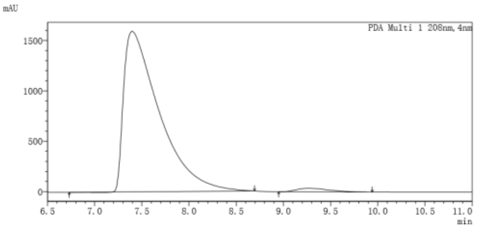

<Peak Results>
PDA Ch1 208nm

Index	Time/min	Height/mAU	Quantity/Area	Area %/%
1	7.397	1594297	41991223	97.967
2	9.269	36705	871258	2.033

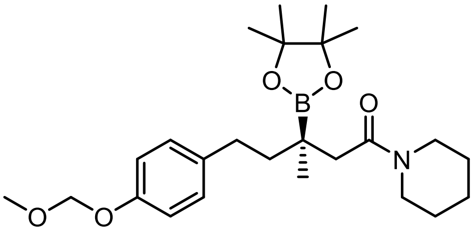

product 3s: Following the general procedure, amide **1s** (79.3 mg, 0.25 mmol) was converted to the boration product. Purification by silica gel chromatography gave 93.5 mg (83% yield) of **3s** as a colorless oil. TLC analysis (hexanes/EtOAc 3:1) R_f= 0.18; **^{1}H NMR** (400 MHz, $CDCl_3$) δ 7.15 – 7.08 (m, 2H), 6.99 – 6.89 (m, 2H), 5.14 (s, 2H), 3.58 (ddt, *J* = 17.1, 11.5, 5.6 Hz, 2H), 3.47 (s, 3H), 3.36 (td, *J* = 13.6, 7.8 Hz, 2H), 2.64 (td, *J* = 12.8, 4.9 Hz, 1H), 2.58 – 2.44 (m, 2H), 2.26 (d, *J* = 16.6 Hz, 1H), 1.72 – 1.52 (m, 8H), 1.23 (d, *J* = 6.8 Hz, 12H), 1.02 (s, 3H). **^{13}C NMR** (101 MHz, $CDCl_3$) δ 174.0,

155.2, 137.6, 129.4, 116.3, 94.8, 81.3, 56.0, 46.8, 44.1, 41.3, 31.2, 26.2, 25.6, 25.4, 25.3, 24.3, 22.4. **ESI-HR** calcd for $C_{25}H_{41}BNO_5^+$ ($[M+H]^+$) 446.3072, found 446.3058. **IR** ν (cm^{-1}) 3418, 2935, 1614, 1150, 885, 669. $[\alpha]^{20}_D$ = + 12.7 (*c* = 0.66, $CHCl_3$) for a 96% *ee* sample. The enantiomeric purity of this compound was determined by HPLC analysis in comparison with authentic racemic material (Chiralpak AD, 99.0:1.0 hexanes:*i*-PrOH, 1.0 mL/min, 208 nm): t_{major} =11.945 min, t_{minor} =13.450 min.

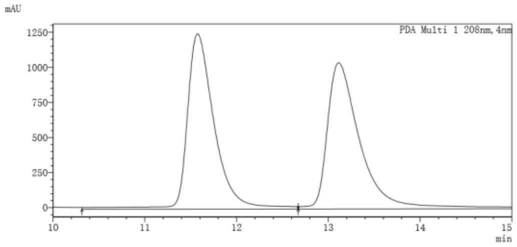

<Peak Results>
PDA Ch1 208nm

Index	Time/min	Height/mAU	Quantity/Area	Area %/%
1	11.575	1248824	25762400	49.705
2	13.113	1040842	26068061	50.295

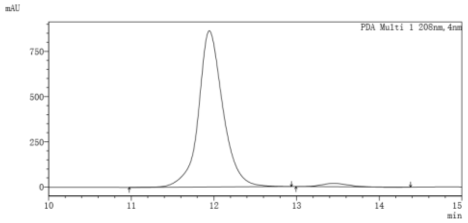

<Peak Results>
PDA Ch1 208nm

Index	Time/min	Height/mAU	Quantity/Area	Area %/%
1	11.945	863258	17701572	97.850
2	13.450	18696	388957	2.150

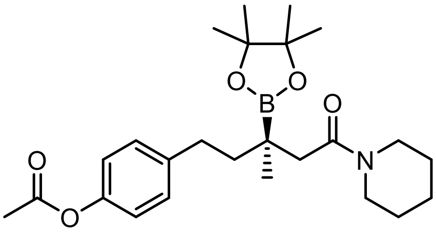

Product 3t: Following the general procedure, amide **1t** (78.8 mg, 0.25 mmol) was converted to the boration product. Purification by silica gel chromatography gave 74.0 mg (67% yield) of **3t** as a colorless oil. TLC analysis (hexanes/EtOAc 1:1) R_f = 0.35; **^{1}H NMR** (400 MHz, $CDCl_3$) δ 7.19 (d, *J* = 8.2 Hz, 2H), 7.03 – 6.90 (m, 2H), 3.58 (tq, *J* = 13.2, 7.5, 6.5 Hz, 2H), 3.35 (q, *J* = 5.3 Hz, 2H), 2.70 (td, *J* = 12.9, 4.8 Hz, 1H), 2.57 (td, *J* = 12.8, 5.4 Hz, 1H), 2.47 (d, *J* = 16.7 Hz, 1H), 2.28 (s, 4H), 1.76 – 1.47 (m, 8H), 1.23 (d, *J* = 6.2 Hz, 12H), 1.02 (s, 3H). **^{13}C NMR** (101 MHz, $CDCl_3$) δ 174.0, 169.9, 148.5, 141.7, 129.4, 121.3, 81.2, 46.8, 44.2, 44.1, 41.0, 31.4, 26.2, 25.6, 25.4, 25.3, 24.3, 22.4, 21.3.**ESI-HR** calcd for $C_{25}H_{39}BNO_5^+$ ($[M+H]^+$) 444.2916, found 444.2907. **IR** ν (cm^{-1}) 2937, 2858, 1758, 1629, 1215, 1133, 732. **M. P.** 112-114 °C. $[\alpha]^{20}_D$ = + 8.6 (*c* = 0.63, $CHCl_3$) for a 98% *ee* sample. The enantiomeric purity of this compound was determined by HPLC analysis in comparison with authentic racemic material

(Chiralpak OD, 99.2:0.8 hexanes:*i*-PrOH, 1.0 mL/min, 208 nm): t_{major} = 19.192 min, t_{minor} =16.994 min.

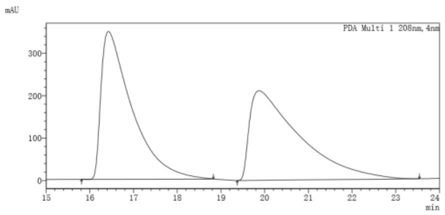

<Peak Results>
PDA Ch1 208nm

Index	Time/min	Height/mAU	Quantity/Area	Area %/%
1	16.423	348585	17094390	50.408
2	19.872	211283	16817545	49.592

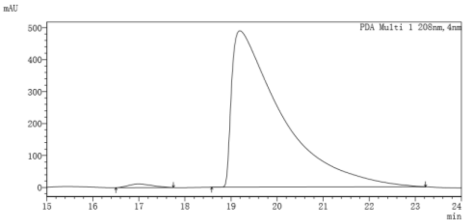

<Peak Results>
PDA Ch1 208nm

Index	Time/min	Height/mAU	Quantity/Area	Area %/%
1	16.994	11109	406294	1.092
2	19.192	489279	36804494	98.908

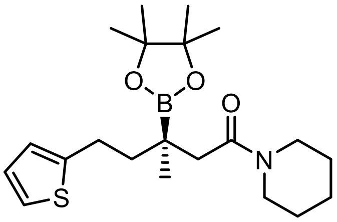

product 3u: Following the general procedure, amide **1u** (65.8 mg, 0.25 mmol) was converted to the boration product. Purification by silica gel chromatography gave 68.4 mg (70% yield) of **3u** as a white solid. TLC analysis (hexanes/EtOAc 2:1) R_f = 0.30; **^{1}H NMR** (400 MHz,$CDCl_3$) δ 7.07 (dd, *J* = 5.0, 1.1 Hz, 1H), 6.96 – 6.84 (m, 1H), 6.78 (d, *J* = 3.4 Hz, 1H), 3.57 (qt, *J* = 12.7, 5.4 Hz, 2H), 3.35 (q, *J* = 5.4 Hz, 2H), 2.86 (dttd, *J* = 24.5, 18.3, 12.7, 12.3, 5.2 Hz, 2H), 2.49 (d, *J* = 16.7 Hz, 1H), 2.27 (d, *J* = 16.7 Hz, 1H), 1.85 – 1.49 (m, 8H), 1.23 (d, *J* = 5.5 Hz, 12H), 1.01 (s, 3H). **^{13}C NMR** (101 MHz, $CDCl_3$) δ 174.1, 147.2, 126.7, 123.7, 122.6, 81.2, 46.8, 44.2, 44.0, 41.1, 26.2, 26.1, 25.6, 25.3, 25.3, 24.3, 22.4. **ESI-HR** calcd for $C_{21}H_{35}BNO_3S^+$ ($[M+H]^+$) 392.2425, found 392.2416. **IR** ν (cm^{-1}) 3406, 2975, 1620, 1142, 852. **M. P.** 89-91 ºC. $[\alpha]^{20}_D$ = + 17.1 (*c* = 0.42, $CHCl_3$) for a 97% *ee* sample. The enantiomeric purity of this compound was determined by HPLC analysis in comparison with authentic racemic material (Chiralpak OD, 99.2:0.8 hexanes:*i*-PrOH, 1.0 mL/min, 208 nm): t_{major} =11.188 min, t_{minor} =7.905 min.

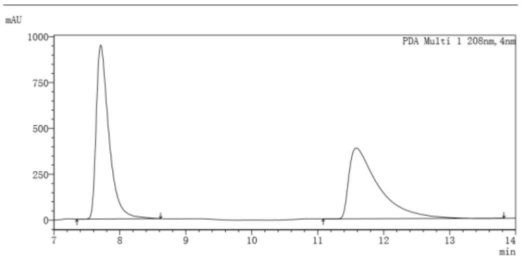

<Peak Results>
PDA Ch1 208nm

Index	Time/min	Height/mAU	Quantity/Area	Area %/%
1	7.710	948862	12720611	51.414
2	11.585	386445	12020862	48.586

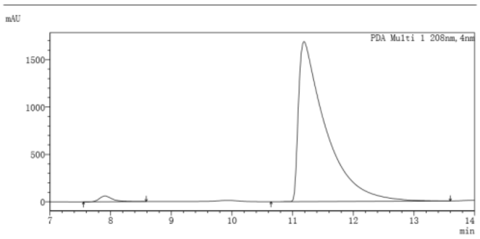

<Peak Results>
PDA Ch1 208nm

Index	Time/min	Height/mAU	Quantity/Area	Area %/%
1	7.905	60532	929959	1.739
2	11.188	1687888	52557168	98.261

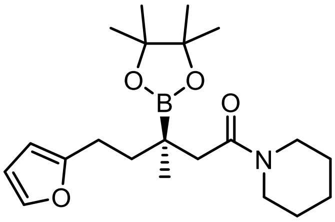

product 3v: Following the general procedure, amide **1v** (61.8 mg, 0.25 mmol) was converted to the boration product. Purification by silica gel chromatography gave 61.6 mg (66% yield) of **3v** as a white solid. TLC analysis (hexanes/EtOAc 1:1) R_f = 0.50; **^{1}H NMR** (400 MHz, $CDCl_3$) δ 7.27 (s, 1H), 6.26 (dd, *J* = 3.1, 1.9 Hz, 1H), 5.96 (d, *J* = 3.1 Hz, 1H), 3.65 – 3.49 (m, 2H), 3.35 (q, *J* = 5.6 Hz, 2H), 2.67 (dddd, *J* = 44.8, 15.6, 11.5, 5.4 Hz, 2H), 2.45 (d, *J* = 16.7 Hz, 1H), 2.25 (d, *J* = 16.7 Hz, 1H), 1.80 – 1.51 (m, 8H), 1.22 (d, *J* = 4.2 Hz, 12H), 0.99 (s, 3H). **^{13}C NMR** (101 MHz, $CDCl_3$) δ 174.3, 157.8, 140.5, 110.2, 104.2, 81.1, 46.8, 44.3, 43.7, 36.6, 26.2, 25.5, 25.3, 25.3, 24.3, 24.1, 22.1. **ESI-HR** calcd for $C_{21}H_{35}BNO_4^+$ ($[M+H]^+$) 376.2654, found 376.2643. **IR** ν (cm^{-1}) 3417, 2975, 2938, 1617, 1140, 884. **M. P.** 64-66 ^{o}C. $[\alpha]^{20}_D$ = + 27.1 (*c* = 0.38, $CHCl_3$) for a 93% *ee* sample. The enantiomeric purity of this compound was determined by HPLC analysis in comparison with authentic racemic material (Chiralpak OD, 99.2:0.8 hexanes:*i*-PrOH, 1.0 mL/min, 208 nm): t_{major} =7.301 min, t_{minor} =8.722 min.

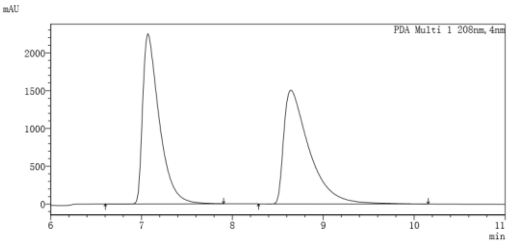

<Peak Results>
PDA Ch1 208nm

Index	Time/min	Height/mAU	Quantity/Area	Area %/%
1	7.072	2249797	28501092	49.423
2	8.644	1502419	29166718	50.577

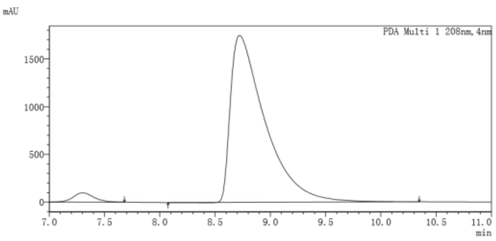

<Peak Results>
PDA Ch1 208nm

Index	Time/min	Height/mAU	Quantity/Area	Area %/%
1	7.301	98437	1290695	3.272
2	8.722	1749624	38155208	96.728

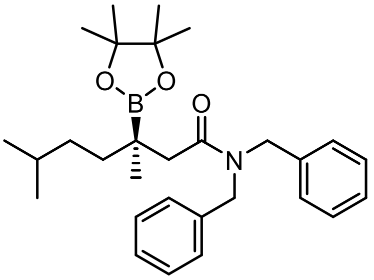

product 3w: Following the general procedure, amide **1w** (83.8 mg, 0.25 mmol) was converted to the boration product. Purification by silica gel chromatography gave 81.2 mg (70% yield) of **3w** as a white solid. TLC analysis (hexanes/EtOAc 9:1) R_f = 0.33; **^{1}H NMR** (400 MHz, $CDCl_3$) δ 7.42 – 7.09 (m, 10H), 4.84 (d, *J* = 14.7 Hz, 1H), 4.47 (d, *J* = 16.9 Hz, 1H), 4.28 (dd, *J* = 18.6, 15.9 Hz, 2H), 2.66 (d, *J* = 16.6 Hz, 1H), 2.28 (d, *J* = 16.6 Hz, 1H), 1.43 (td, *J* = 12.9, 7.1 Hz, 2H), 1.29 (d, *J* = 6.5 Hz, 13H), 1.18 – 1.11 (m, 2H), 1.02 (s, 3H), 0.85 (dd, *J* = 6.6, 3.0 Hz, 6H). **^{13}C NMR** (101 MHz, $CDCl_3$) δ 174.1, 137.4, 136.4, 129.0, 128.6, 128.5, 127.7, 127.5, 126.5, 82.5, 49.5, 48.1, 44.4, 36.4, 34.3, 29.0, 25.2, 25.2, 22.9, 22.8, 21.7. **ESI-HR** calcd for $C_{29}H_{43}BNO_3^+$ ($[M+H]^+$) 464.3331, found 464.3330. **IR** ν (cm^{-1}) 2923, 1644, 1146, 858. **M. P.** 88-90 °C. $[\alpha]^{20}_D$ = + 49.3 (*c* = 0.54, $CHCl_3$) for a 94% *ee* sample. The enantiomeric purity of this compound was determined by HPLC analysis in comparison with authentic racemic material (Chiralpak IA, 99.6:0.4 hexanes:*i*-PrOH, 1.0 mL/min, 208 nm): t_{major} =7.806 min, t_{minor} =9.780 min.

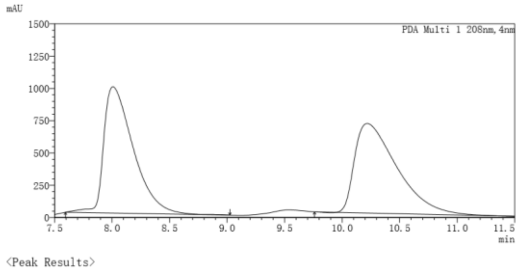

<Peak Results>
PDA Ch1 208nm

Index	Time/min	Height/mAU	Quantity/Area	Area %/%
1	8.007	978358	17551389	49.776
2	10.218	693649	17709410	50.224

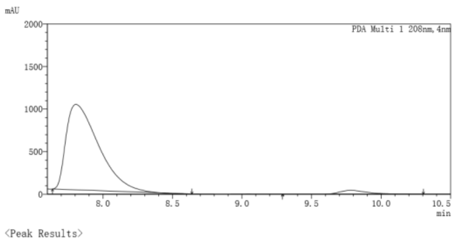

<Peak Results>
PDA Ch1 208nm

Index	Time/min	Height/mAU	Quantity/Area	Area %/%
1	7.806	1004542	16375524	96.388
2	9.780	45185	613569	3.612

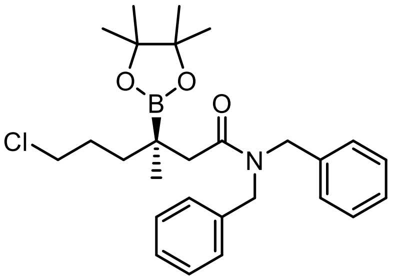

product 3x: Following the general procedure, amide **1x** (85.3 mg, 0.25 mmol) was converted to the boration product. Purification by silica gel chromatography gave 104.6 mg (89% yield) of **3x** as a white solid. TLC analysis (hexanes/EtOAc 10:1) R_f= 0.25; **^{1}H NMR** (400 MHz, $CDCl_3$) δ 7.39 – 6.99 (m, 10H), 4.64 (d, *J* = 14.7 Hz, 1H), 4.40 – 4.21 (m, 3H), 3.42 (tt, *J* = 6.8, 3.5 Hz, 2H), 2.53 (d, *J* = 16.6 Hz, 1H), 2.29 (d, *J* = 16.6 Hz, 1H), 1.81 – 1.58 (m, 2H), 1.48 (td, *J* = 12.5, 4.6 Hz, 1H), 1.33 (td, *J* = 12.7, 4.7 Hz, 1H), 1.21 (d, *J* = 3.9 Hz, 12H), 0.94 (s, 3H). **^{13}C NMR** (101 MHz, $CDCl_3$) δ 174.3, 137.1, 136.0, 129.1, 128.7, 128.5, 127.8, 127.6, 126.5, 82.5, 49.7, 48.4, 46.1, 44.2, 35.7, 28.8, 25.2, 25.1, 21.7. **ESI-HR** calcd for $C_{27}H_{38}BClNO_3^+$ ($[M+H]^+$) 470.2628, found 470.2622. **IR** ν (cm^{-1}) 2977, 1638, 1264, 733. **M. P.** 90-92 ºC. $[\alpha]^{20}_D$ = + 8.6 (*c* = 1.88, $CHCl_3$) for a 97% ee sample. The enantiomeric purity of this compound was determined by HPLC analysis in comparison with authentic racemic material (Chiralpak AD, 99.5:0.5 hexanes:*i*-PrOH, 1.0 mL/min, 208 nm): t_{major} = 13.804 min, t_{minor} =17.646 min.

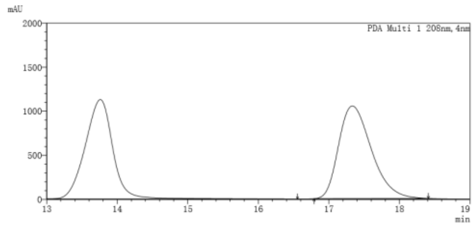

<Peak Results>
PDA Ch1 208nm

Index	Time/min	Height/mAU	Quantity/Area	Area %/%
1	13.760	1127766	30702736	47.476
2	17.333	1051781	33967587	52.524

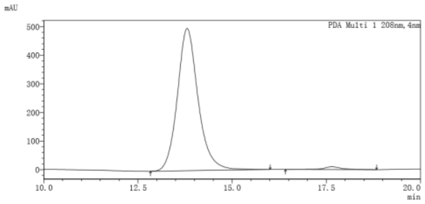

<Peak Results>
PDA Ch1 208nm

Index	Time/min	Height/mAU	Quantity/Area	Area %/%
1	13.804	497003	19089396	98.308
2	17.646	9816	328512	1.692

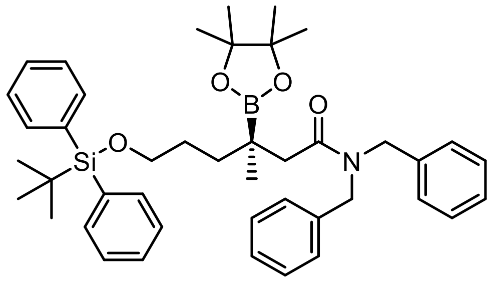

product 3y: Following the general procedure with 5 mol% catalyst, amide **1y** (140.3 mg, 0.25 mmol) was converted to the boration product. Purification by silica gel chromatography gave 154.6 mg (90% yield) of **3y** as a colorless oil. TLC analysis (hexanes/EtOAc 7:1) R_f= 0.31; **^{1}H NMR** (400 MHz,$CDCl_3$) δ 7.67 – 7.61 (m, 4H), 7.42 – 7.20 (m, 14H), 7.14 (d, *J* = 6.9 Hz, 2H), 4.81 (d, *J* = 14.7 Hz, 1H), 4.46 (d, *J* = 17.0 Hz, 1H), 4.28 (dd, *J* = 15.8, 13.1 Hz, 2H), 3.61 (t, *J* = 6.6 Hz, 2H), 2.62 (d, *J* = 16.5 Hz,

1H), 2.28 (d, *J* = 16.6 Hz, 1H), 1.68 – 1.40 (m, 4H), 1.27 (d, *J* = 5.7 Hz, 12H), 1.02 (d, *J* = 5.7 Hz, 12H). **^{13}C NMR** (101 MHz, $CDCl_3$) δ 174.1, 137.4, 136.4, 135.7, 134.3, 129.6, 129.1, 128.6, 128.6, 127.8, 127.7, 127.5, 126.6, 82.5, 65.0, 49.7, 48.2, 44.4, 34.8, 28.6, 27.0, 25.3, 25.1, 21.8, 19.3. **ESI-HR** calcd for $C_{43}H_{57}BNO_4Si^+$ ($[M+H]^+$) 690.4144, found 690.4128. **IR** ν (cm^{-1}) 2930, 2837, 1642, 1146, 737. $[\alpha]^{20}_D$ = + 22.8 (*c* = 0.50, $CHCl_3$) for a 92% *ee* sample. The enantiomeric purity of this compound was determined by HPLC analysis in comparison with authentic racemic material (Chiralpak AD, 99.2:0.8 hexanes:*i*-PrOH, 1.0 mL/min, 208 nm): t_{major} = 9.492 min, t_{minor} =6,342 min.

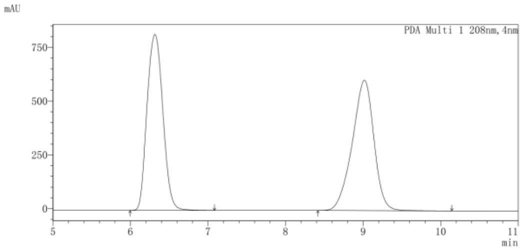

<Peak Results>
PDA Ch1 208nm

Index	Time/min	Height/mAU	Quantity/Area	Area %/%
1	6.315	819074	11621222	49.648
2	9.016	606712	11786185	50.352

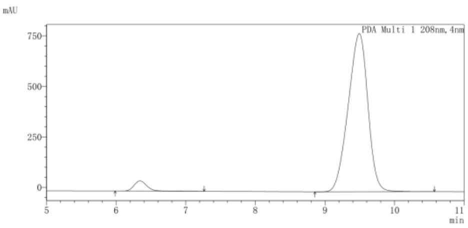

<Peak Results>
PDA Ch1 208nm

Index	Time/min	Height/mAU	Quantity/Area	Area %/%
1	6.342	50380	654871	4.076
2	9.492	784354	15411798	95.924

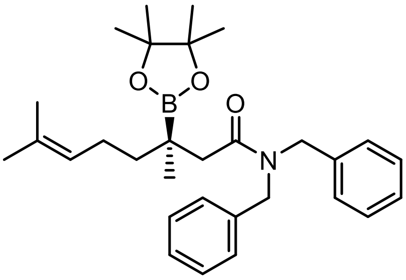

product 3z: Following the general procedure, amide **1z** (86.8 mg, 0.25 mmol) was converted to the boration product. Purification by silica gel chromatography gave 83.9 mg (71% yield) of **3z** as a white solid. TLC analysis (hexanes/EtOAc 9:1) R_f= 0.43; **^{1}H NMR** (400 MHz, $CDCl_3$) δ 7.33 – 7.03 (m, 10H), 5.01 (td, *J* = 7.0, 3.7 Hz, 1H), 4.76 (d, *J* = 14.7 Hz, 1H), 4.39 (d, *J* = 16.9 Hz, 1H), 4.21 (m, *J* = 15.4 Hz, 2H), 2.59 (d, *J* = 16.6 Hz, 1H), 2.24 (d, *J* = 16.6 Hz, 1H), 1.88 (q, *J* = 8.0 Hz, 2H), 1.49 (s, 3H), 1.42 – 1.33 (m, 1H), 1.22 (m, *J* = 7.1 Hz, 13H), 0.97 (s, 3H). **^{13}C NMR** (101 MHz, $CDCl_3$) δ 174.2, 130.9, 129.1, 128.6, 128.5, 127.7, 127.5, 126.6, 125.4, 82.5, 49.6, 48.2, 44.3, 38.9, 25.8, 25.3, 25.1, 24.0, 21.7, 17.7. **IR** ν (cm^{-1}) 2974, 1638, 1110, 733, 699. **ESI-**

HR calcd for $C_{30}H_{43}BNO_3^+$ ($[M+H]^+$) 476.3331, found 476.3321. **M. P.** 80-82 ^oC. $[\alpha]^{20}_D$ = + 61.6 (*c* = 0.45, $CHCl_3$) for a 94% *ee* sample. The enantiomeric purity of this compound was determined by HPLC analysis in comparison with authentic racemic material (Chiralpak AD, 99.5:0.5 hexanes:*i*-PrOH, 1.0 mL/min, 208 nm): t_{major} = 9.298 min, t_{minor} =14.821 min.

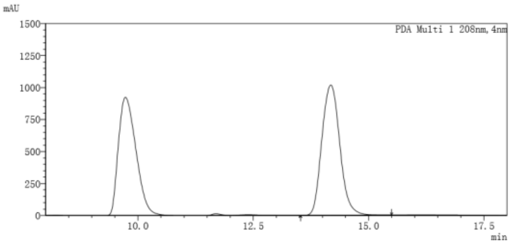

<Peak Results>
PDA Ch1 208nm

Index	Time/min	Height/mAU	Quantity/Area	Area %/%
1	9.730	929493	25417072	47.930
2	14.181	1017685	27612435	52.070

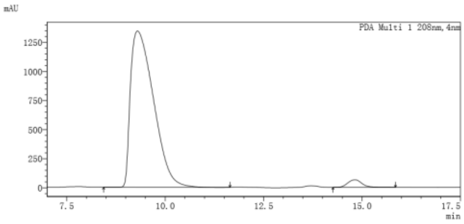

<Peak Results>
PDA Ch1 208nm

Index	Time/min	Height/mAU	Quantity/Area	Area %/%
1	9.298	1342600	54553484	97.205
2	14.821	64111	1568889	2.795

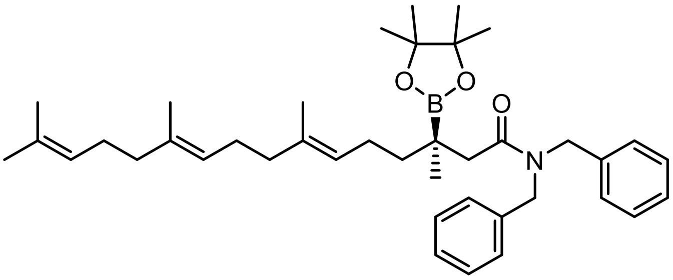

product 3aa: Following the general procedure, amide **1aa** (120.8 mg, 0.25 mmol) was converted to the boration product. Purification by silica gel chromatography gave 133.4 mg (73% yield) of **3aa** as a colorless oil. TLC analysis (hexanes/EtOAc 9:1) R_f= 0.44; **^{1}H NMR** (400 MHz, $CDCl_3$) δ 7.43 – 7.10 (m, 10H), 5.21 – 4.99 (m, 3H), 4.82 (d, *J* = 14.8 Hz, 1H), 4.46 (d, *J* = 16.9 Hz, 1H), 4.29 (dd, *J* = 15.8, 12.8 Hz, 2H), 2.66 (d, *J* = 16.6 Hz, 1H), 2.31 (d, *J* = 16.5 Hz, 1H), 2.10 – 1.89 (m, 10H), 1.67 (s, 3H), 1.58 (d, *J* = 7.6 Hz, 9H), 1.50 – 1.42 (m, 1H), 1.29 (d, *J* = 7.0 Hz, 13H), 1.05 (s, 3H). **^{13}C NMR** (101 MHz, $CDCl_3$) δ 174.1, 137.4, 136.4, 135.0, 134.5, 131.3, 129.1, 128.6, 128.5, 127.7, 127.5, 126.6, 125.3, 124.5, 124.4, 82.5, 49.6, 48.2, 44.3, 39.8, 38.9, 26.9, 26.8, 25.8, 25.3, 25.1, 23.9, 23.6, 21.8, 17.8, 16.1, 16.1.**ESI-HR** calcd for $C_{40}H_{59}BNO_3^+$ ($[M+H]^+$) 612.4583, found 612.4577. **IR** ν (cm^{-1}) 2957, 2924, 2859, 1646, 1466. 749. $[\alpha]^{20}_D$ = + 58.9 (*c* = 0.65, $CHCl_3$) for a 94% *ee* sample. The enantiomeric purity of this compound was determined by HPLC analysis in comparison with authentic racemic material

(Chiralpak AD, 99.7:0.3 hexanes:*i*-PrOH, 1.0 mL/min, 208 nm): t_{major} =13.280 min, t_{minor} =9.324 min.

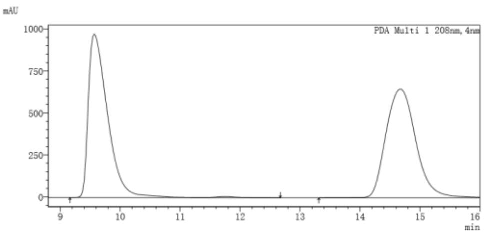

<Peak Results>
PDA Ch1 208nm

Index	Time/min	Height/mAU	Quantity/Area	Area %/%
1	9.569	972385	21772794	49.252
2	14.673	647313	22434281	50.748

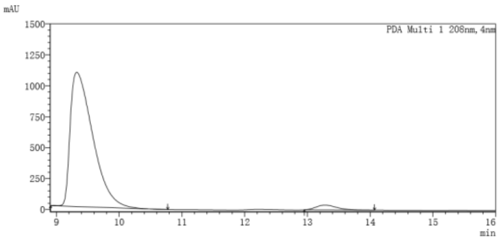

<Peak Results>
PDA Ch1 208nm

Index	Time/min	Height/mAU	Quantity/Area	Area %/%
1	9.324	1085920	27082598	96.770
2	13.280	39369	904075	3.230

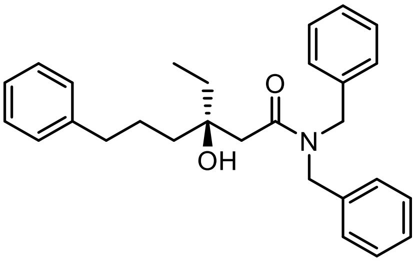

product 3ab-ol: Following the general procedure with amide **1ab** (99.3mg, 0.25 mmol), 7.5 mol% catalyst and 3.5 equivalent of HBpin, and the temperature was increased to 0 °C. After the reaction, the residue was dissolved in THF (2.0 mL), 3 M NaOH (2.0 mL) and methanol (2.0 mL) and was cooled to 0 °C. Then 30% aqueous H_2O_2 (0.5 mL) was added dropwise. The reaction was stirred at room temperature for 3 h. Ethyl acetate (20 mL) was added, and the mixture was washed with water (10 mL), brine (10 mL) and dried over Na_2SO_4. After removal of the solvent, the crude product was purified by column chromatography on silica gel with EtOAc/hexanes mixture as eluent to provide 55.6 mg (54% yield) of **3ab-ol** as a colorless oil. TLC analysis (hexanes/EtOAc 7:1) R_f= 0.27; **^{1}H NMR** (400 MHz, $CDCl_3$) δ 7.41 – 7.26 (m, 7H), 7.26 – 7.09 (m, 8H), 5.38 (s, 1H), 4.63 (s, 2H), 4.42 (s, 2H), 2.63 – 2.54 (m, 2H), 2.48 (s, 2H), 1.63 (dt, *J* = 20.8, 6.8 Hz, 2H), 1.54 – 1.41 (m, 3H), 1.37 – 1.13 (m, 3H), 0.87 (t, *J* = 7.2 Hz, 3H). **^{13}C NMR** (101 MHz, $CDCl_3$) δ 174.1, 142.6, 137.0, 136.1, 129.2, 128.9, 128.5, 128.4, 128.3, 128.0, 127.8, 126.4, 125.8, 73.6, 50.2, 48.4, 39.3, 38.5, 36.5, 31.8, 25.8, 8.3.**ESI-HR** calcd for $C_{28}H_{34}NO_2^+$ ([M+H]$^+$) 416.2584, found 416.2578. **IR** ν (cm^{-1}) 3404, 3062, 3027, 937, 1620, 1212, 698. $[\alpha]^{20}_D$ = + 22.4 (*c* = 1.26, $CHCl_3$) for an 87% *ee* sample.

The enantiomeric purity of this compound was determined by HPLC analysis in comparison with authentic racemic material (Chiralpak OD, 90.0:10.0 hexanes:*i*-PrOH, 1.0 mL/min, 208 nm): t_{major} =10.240 min, t_{minor} =11.763 min.

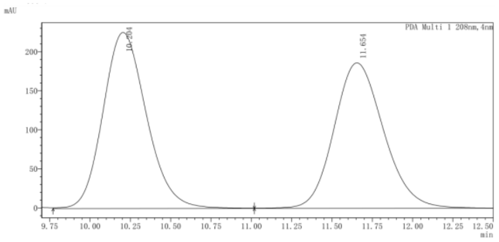

PDA Ch1 208nm

峰号	保留时间	峰宽(高度 50%)	高度	面积	面积%
1	10.204	0.287	225607	4225446	51.433
2	11.654	0.328	186364	3989944	48.567

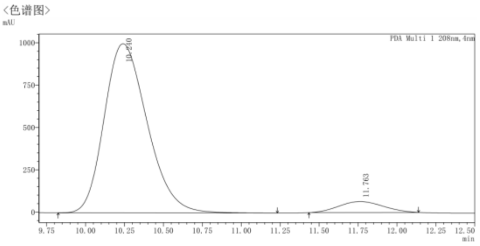

PDA Ch1 208nm

峰号	保留时间	峰宽(高度 50%)	高度	面积	面积%
1	10.240	0.296	999649	19174069	93.701
2	11.763	0.318	65081	1288957	6.299

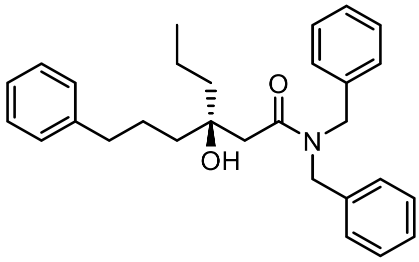

product 3ac-ol: Following the procedure described above. Amide **1ac** (102.8mg, 0.25 mmol) was converted to the alcohol product. Purification by silica gel chromatography gave 53.9 mg (50% yield) of **3ac-ol** as a colorless oil. TLC analysis (hexanes/EtOAc 7:1) R_f= 0.30; **^{1}H NMR**(400 MHz, $CDCl_3$) δ 7.41 – 7.26 (m, 7H), 7.26 – 7.09 (m, 8H), 5.38 (s, 1H), 4.63 (s, 2H), 4.42 (s, 2H), 2.63 – 2.54 (m, 2H), 2.48 (s, 2H), 1.63 (dt, *J* = 20.8, 6.8 Hz, 2H), 1.54 – 1.41 (m, 3H), 1.37 – 1.13 (m, 3H), 0.87 (t, *J* = 7.2 Hz, 3H). **^{13}C NMR** (101 MHz, $CDCl_3$) δ 174.2, 142.6, 137.1, 136.2, 129.2, 128.9, 128.5, 128.4, 128.4, 128.0, 127.8, 126.4, 125.9, 73.4, 50.3, 48.4, 41.9, 39.6, 39.2, 36.5, 25.8, 17.2, 14.8. **ESI-HR** calcd for $C_{29}H_{36}NO_2^+$ ($[M+H]^+$) 430.2741, found 430.2726. **IR** ν (cm^{-1}) 3397, 3027, 2931, 2870, 1618, 1328, 1126, 698. $[\alpha]^{20}_D$ = + 16.4 (*c* = 0.37, $CHCl_3$) for a 91% *ee* sample. The enantiomeric purity of this compound was determined by HPLC analysis in comparison with authentic racemic material (Chiralpak OD, 90.0:10.0 hexanes:*i*-PrOH, 1.0 mL/min, 208 nm): t_{major} =9.785 min, t_{minor} =12.031 min.

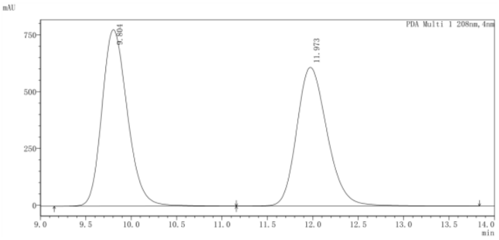

PDA Ch1 208nm

峰号	保留时间	峰宽(高度 50%)	高度	面积	面积%
1	9.804	0.298	777207	15184671	51.544
2	11.973	0.357	610179	14274840	48.456

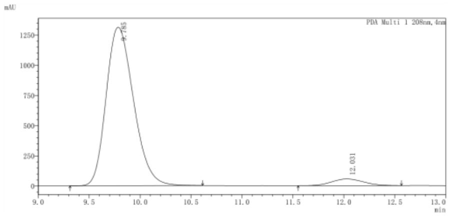

PDA Ch1 208nm

峰号	保留时间	峰宽(高度 50%)	高度	面积	面积%
1	9.785	0.290	1312842	24581429	95.327
2	12.031	0.342	55256	1204922	4.673

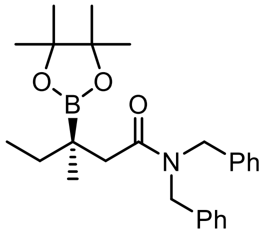

product 3ad: Following the general procedure, amide **1ad** (73.3 mg, 0.25 mmol) was converted to the boration product. Purification by silica gel chromatography gave 98.3 mg (93% yield) of **3ad** as a colorless oil. TLC analysis (hexanes/EtOAc 11:1) R_f = 0.36; **^{1}H NMR** (400 MHz, $CDCl_3$) δ 7.45 – 7.02 (m, 10H), 4.78 (d, J = 14.7 Hz, 1H), 4.46 (d, J = 17.0 Hz, 1H), 4.31 (dd, J = 15.8, 5.0 Hz, 2H), 2.62 (d, J = 16.6 Hz, 1H), 2.29 (d, J = 16.5 Hz, 1H), 1.56 – 1.44 (m, 1H), 1.29 (d, J = 6.4 Hz, 13H), 1.01 (s, 3H), 0.85 (t, J = 7.5 Hz, 3H). **^{13}C NMR** (101 MHz, $CDCl_3$) δ 174.1, 137.5, 136.4, 129.0, 128.6, 128.5, 127.7, 127.5, 126.6, 82.5, 49.6, 48.2, 44.1, 31.1, 25.2, 25.1, 21.2, 9.6. **ESI-HR** calcd for $C_{26}H_{37}BNO_3^+$ ($[M+H]^+$) 422.2861, found 422.2859. **IR** ν (cm^{-1}) 2972, 1644, 1495, 1207, 750. $[\alpha]^{20}_{D}$ = + 58.3 (*c* = 1.44, $CHCl_3$) for a 95% *ee* sample The enantiomeric purity of this compound was determined by HPLC analysis in comparison with authentic racemic material (Chiralpak AD, 99.2:0.8 hexanes:*i*-PrOH, 1.0 mL/min, 208 nm): t_{major} =5.830 min, t_{minor} =6.469 min.

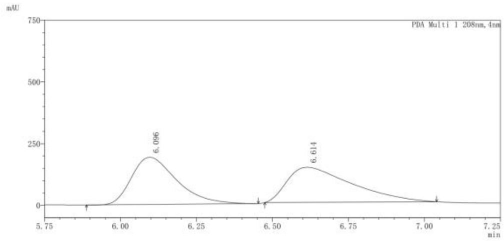

PDA Ch1 208nm

峰号	保留时间	峰宽(高度 50%)	高度	面积	面积%
1	6.096	0.157	190564	1971372	48.380
2	6.614	0.230	142644	2103406	51.620

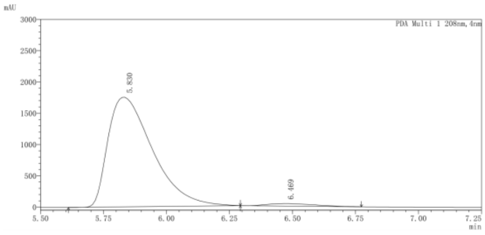

PDA Ch1 208nm

峰号	保留时间	峰宽(高度 50%)	高度	面积	面积%
1	5.830	0.190	1751129	21558364	97.685
2	6.469	0.215	39128	510898	2.315

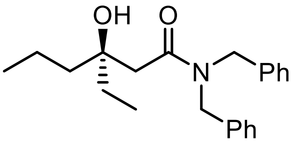

product 3ae-ol: Following the procedure described above. Amide **1ae** (80.3 mg, 0.25 mmol), 7.5 mol% catalyst and 3.5 equivalent of HBpin, and the temperature was increased to 0 °C. After the reaction, the residue was dissolved in THF (2.0 mL), 3 M NaOH (2.0 mL) and methanol (2.0 mL) and was cooled to 0 °C. Then 30% aqueous H_2O_2 (0.5 mL) was added dropwise. The reaction was stirred at room temperature for 3 h. Ethyl acetate (20 mL) was added, and the mixture was washed with water (10 mL), brine (10 mL) and dried over Na_2SO_4. After removal of the solvent, the crude product was purified by column chromatography on silica gel with EtOAc/hexanes mixture as eluent to provide 46.1 mg (54% yield) of **3ae-ol** as a colorless oil. TLC analysis (hexanes/EtOAc 9:1) R_f= 0.48; **^{1}H NMR** (400 MHz, $CDCl_3$) δ 7.53 – 7.07 (m, 10H), 5.32 (s, 1H), 4.70 – 4.58 (m, 2H), 4.46 (s, 2H), 2.50 (d, J = 2.0 Hz, 2H), 1.62 – 1.42 (m, 4H), 1.36 – 1.17 (m, 2H), 0.87 (dt, J = 21.6, 7.4 Hz, 6H). **^{13}C NMR** (101 MHz, $CDCl_3$) δ 174.2, 137.1, 136.2, 129.2, 128.8, 128.3, 127.9, 127.7, 126.4, 73.6, 50.3, 48.4, 41.2, 39.3, 31.8, 17.1, 14.8, 8.2. **ESI-HR** calcd for $C_{22}H_{30}NO_2^+$ ([M+H]$^+$) 340.2271, found 340.2256. **IR** ν (cm^{-1}) 3405, 2962, 1620, 1214, 1080, 700. $[\alpha]^{20}_D$ = + 20.8 (*c* = 1.49, $CHCl_3$) for a 89% *ee* sample The enantiomeric purity of this compound was determined by HPLC analysis in comparison with authentic racemic material (Chiralpak AD, 90.0:10.0 hexanes:*i*-PrOH, 1.0 mL/min, 208 nm): t_{major} =9.756 min, t_{minor} =8.924 min.

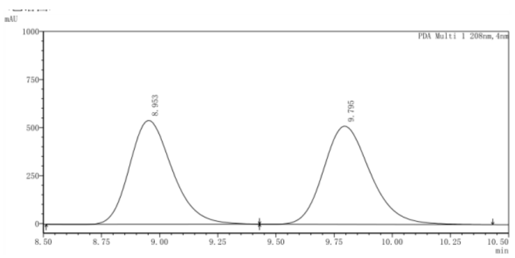

PDA Ch1 208nm

峰号	保留时间	峰宽(高度 50%)	高度	面积	面积%
1	8.953	0.195	539960	6907680	49.074
2	9.795	0.214	510780	7168490	50.926

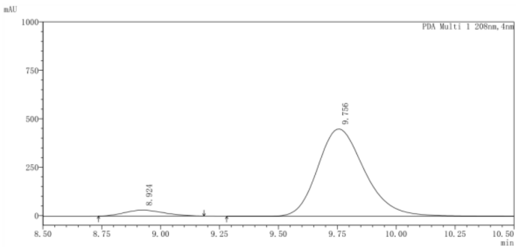

PDA Ch1 208nm

峰号	保留时间	峰宽(高度 50%)	高度	面积	面积%
1	8.924	0.187	30600	362387	5.465
2	9.756	0.211	450738	6269214	94.535

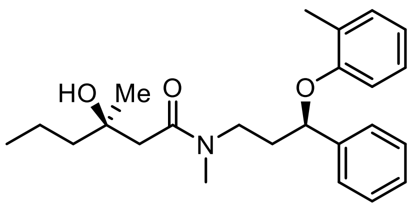

product 3ah-ol: Following the general procedure with 5 mol% catalyst, amide **1ah** (91.3 mg, 0.25 mmol). After the reaction, the residue was dissolved in THF (2.0 mL), 3 M NaOH (2.0 mL) and methanol (2.0 mL) and was cooled to 0 °C. Then 30% aqueous H_2O_2 (0.5 mL) was added dropwise. The reaction was stirred at room temperature for 3 h. Ethyl acetate (20 mL) was added, and the mixture was washed with water (10 mL), brine (10 mL) and dried over Na_2SO_4. After removal of the solvent, Analysis of the crude **^{1}H** and **^{13}C NMR** spectra indicates >20:1 dr. the crude product was purified by column chromatography on silica gel with EtOAc/hexanes mixture as eluent to provide 57.0 mg (59% yield) of **3ah-ol** as a colorless oil. Two sets of peaks were observed in **^{1}H** and **^{13}C NMR** spectra due to carbamate rotamers, which are assigned to major and minor rotamers, respectively. TLC analysis (hexanes/EtOAc 2:1) R_f= 0.31; **^{1}H NMR** (400 MHz, $CDCl_3$) δ 7.40 – 7.20 (m, 5H, *overlapping*), 7.12 (t, J = 8.3 Hz, 1H, *overlapping*), 7.02 – 6.90 (m, 1H, *overlapping*), 6.78 (dt, J = 10.3, 7.4 Hz, 1H, *overlapping*), 6.55 (dd, J = 13.1, 8.2 Hz, 1H, *overlapping*), 5.30 (m, J = 7.3 Hz, 1H, *overlapping*), 5.15 (qm, J = 7.0, 6.3 Hz, 1H, *overlapping*), 3.62 (m, 2H, *major*), 3.51 – 3.34 (m, 2H, *minor*), 2.94 (d, J = 2.1 Hz, 3H, *overlapping*), 2.47 – 2.07 (m, 7H, *overlapping*), 1.57 – 1.09 (m, 7H, *overlapping*), 0.88 (m, 3H, *overlapping*). **^{13}C NMR** (101 MHz, $CDCl_3$) δ 173.3 (*major*), 173.1 (*minor*), 155.8 (*minor*), 155.3 (*major*), 141.5 (*minor*), 140.9 (*major*), 130.9 (*major*), 130.7 (*minor*), 128.9 (*major*), 128.7 (*minor*), 128.0 (*major*), 127.7 (*minor*), 126.9 (*minor*), 126.9 (*major*), 126.7 (*major*), 126.5 (*minor*), 125.7 (*minor*), 125.5 (*major*), 120.8 (*major*), 120.5 (*minor*), 112.7 (*minor*), 112.5 (*major*), 77.5 (*major*), 76.0 (*minor*), 71.2 (*minor*), 71.1 (*major*), 46.4 (*major*), 45.4 (*minor*), 44.7 (*minor*), 44.4 (*major*), 41.7 (*minor*), 41.1 (*major*), 37.6 (*major*), 36.5 (*minor*), 36.2 (*minor*), 33.2 (*major*), 27.0 (*minor*), 26.9 (*major*), 17.4 (*minor*), 17.1 (*major*), 16.6 (*minor*), 16.6 (*major*), 14.7 (*minor*), 14.7 (*major*). **ESI-HR** calcd for $C_{24}H_{34}NO_3^+$ ($[M+H]^+$) 384.2533,

found 384.2528. **IR** ν (cm^{-1}) 3398, 3019, 2958, 1620, 1493, 1214, 743. $[\alpha]^{20}_{D}$ = + 48.7 (*c* = 0.79, $CHCl_3$).

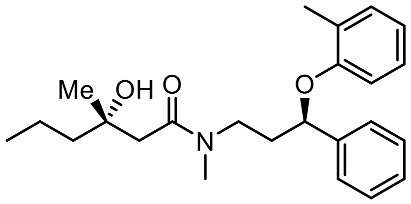

product 3ak-ol: Following the general procedure with 5 mol% catalyst and 6mol% (−)-DIOP, amide **1ah** (91.3 mg, 0.25 mmol). After the reaction, the residue was dissolved in THF (2.0 mL), 3 M NaOH (2.0 mL) and methanol (2.0 mL) and was cooled to 0 °C. Then 30% aqueous H_2O_2 (0.5 mL) was added dropwise. The reaction was stirred at room temperature for 3 h. Ethyl acetate (20 mL) was added, and the mixture was washed with water (10 mL), brine (10 mL) and dried over Na_2SO_4. After removal of the solvent, Analysis of the crude **^{1}H** and **^{13}C NMR** spectra indicates >20:1 dr. the crude product was purified by column chromatography on silica gel with EtOAc/hexanes mixture as eluent to provide 58.5 mg (61% yield) of **3ak-ol** as a colorless oil. Two sets of peaks were observed in **^{1}H** and **^{13}C NMR** spectra due to carbamate rotamers, which are assigned to major and minor rotamers, respectively. TLC analysis (hexanes/EtOAc 2:1) R_f= 0.31; **^{1}H NMR** (400 MHz, $CDCl_3$) δ 7.39 – 7.22 (m, 5H, *overlapping*), 7.19 – 7.07 (m, 1H, *overlapping*), 6.95 (t, J = 7.8 Hz, 1H, *overlapping*), 6.78 (dt, J = 10.5, 7.4 Hz, 1H, *overlapping*), 6.56 (dd, J = 14.3, 8.2 Hz, 1H, *overlapping*), 5.30 (s, 1H, *overlapping*), 5.22 – 5.09 (m, 1H, *overlapping*), 3.63 (m, 2H, *major*), 3.42 (m, 2H, *minor*), 2.95 (d, J = 3.3 Hz, 3H *overlapping*), 2.48 – 2.12 (m, 7H *overlapping*), 1.54 – 1.41 (m, 1H *overlapping*), 1.38 – 1.24 (m, 3H *overlapping*), 1.12 (m, 3H *overlapping*), 0.91 (m, 3H, *overlapping*). **^{13}C NMR** (101 MHz, $CDCl_3$) δ 173.3 (*major*), 173.2 (*minor*), 155.8 (*minor*), 155.4 (*major*), 141.6 (*minor*), 140.9 (*major*), 131.0 (*major*), 130.8 (*minor*), 129.0 (*major*), 128.8 (*minor*), 128.0 (*major*), 127.8 (*minor*), 126.9 (*overlapping*), 126.8 (*major*), 126.6 (*minor*), 125.8 (*minor*), 125.6 (*major*), 120.9 (*major*), 120.5 (*minor*), 112.8 (*minor*), 112.6 (*major*), 77.6 (*minor*), 76.1 (*major*), 71.3 (*minor*), 71.2 (*major*), 46.4 (*major*), 45.5 (*minor*), 44.8 (*minor*), 44.7

(*major*), 41.8 (*minor*), 41.1 (*major*), 37.7 (*major*), 36.6 (*minor*), 36.3 (*minor*), 33.3 (*major*), 27.0 (*minor*), 26.8 (*major*), 17.4 (*minor*), 17.2 (*major*), 16.7 (*overlapping*), 14.8 (*overlapping*). **ESI-HR** calcd for $C_{24}H_{34}NO_3^+$ ($[M+H]^+$) 384.2533, found 384.2524. **IR** ν (cm^{-1}) 3400, 3028, 2958, 1619, 1492, 1212, 737. $[\alpha]^{20}_D$ = -17.9 (*c* = 0.80, $CHCl_3$).

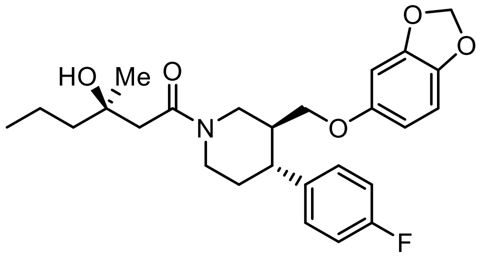

product 3ai-ol: Following the procedure described above. Amide **1aj** (109.8mg, 0.25 mmol) was converted to the alcohol product. Analysis of the crude **^{1}H** and **^{13}C NMR** spectra indicates >20:1 dr. Purification by silica gel chromatography gave 73.7 mg (64% yield) of **3ai-ol** as a colorless oil. Two sets of peaks were observed in **^{1}H, ^{13}C** and **^{19}F NMR** spectra due to carbamate rotamers, which are assigned to major and minor rotamers, respectively. TLC analysis (hexanes/EtOAc 2:1) R_f = 0.27; **^{1}H NMR** (400 MHz, $CDCl_3$) δ 7.13 (dd, J = 8.2, 5.3 Hz, 2H, *overlapping*), 6.99 (td, J = 8.5, 6.7 Hz, 2H, *overlapping*), 6.63 (t, J = 8.4 Hz, 1H, *overlapping*), 6.36 (dd, J = 5.6, 2.5 Hz, 1H, *overlapping*), 6.15 (dd, J = 8.4, 2.5 Hz, 1H, *overlapping*), 5.89 (s, 2H, *overlapping*), 5.21 (s, 1H, *overlapping*), 5.04 – 4.95 (m, 1H, *major*), 4.87 – 4.76 (m, 1H, *minor*), 4.19 (m, 1H, *major*), 4.05 – 3.93 (m, 1H, *minor*), 3.64 (dd, J = 10.0, 2.9 Hz, 1H, , *overlapping*), 3.58 – 3.44 (m, 1H, , *overlapping*), 3.23 – 3.05 (m, 1H, *overlapping*), 2.93 – 2.62 (m, 2H, *overlapping*), 2.58 – 2.39 (m, 2H, *overlapping*), 2.08 – 1.85 (m, 2H, *overlapping*), 1.69 (m, 1H, *overlapping*), 1.61 – 1.32 (m, 4H, *overlapping*), 1.26 (s, 3H, *overlapping*), 0.95 (m, 3H, *overlapping*). **^{13}C NMR** (101 MHz, $CDCl_3$) δ 171.5 (*overlapping*), 161.9 (J_1 = 245.5 Hz) (*overlapping*), 154.3 (*minor*), 153.8 (*major*), 148.4 (*major*), 148.3 (*minor*), 142.1 (*major*), 141.9 (*minor*), 138.4 (J_4 = 3.7 Hz) (*overlapping*),

128.9(J_3 = 8.1 Hz) (*minor*), 128.8 (J_3 = 8.1 Hz) (*major*), 115.9 (J_2 = 22.5 Hz) (*major*), 115.7 (J_2 = 22.5 Hz) (*minor*),108.1 (*major*), 108.0 (*minor*), 105.8 (*minor*), 105.7 (*major*), 101.4 (*major*), 101.2 (*minor*), 98.2 (*minor*), 98.1 (*major*), 71.3 (*overlapping*), 68.7 (*overlapping*), 49.2 (*major*), 46.3 (*minor*), 45.0 (*major*), 44.9 (*minor*), 44.8 (*minor*), 44.4 (*major*), 43.9 (*major*), 42.9 (*minor*), 42.2 (*major*), 42.1 (*minor*), 41.4 (*minor*), 41.3 (*major*), 34.6 (*minor*), 33.7 (*major*), 27.3 (*minor*), 27.1 (*major*), 17.5 (*minor*), 17.4 (*major*), 14.8 (*major*), 14.8 (*minor*). **^{19}F NMR** (376 MHz, CDCl3) δ -115.6 (*major*), -115.7 (*minor*). **ESI-HR** calcd for $C_{26}H_{33}FNO_5^+$ ($[M+H]^+$) 458.2337, found 458.2331. **IR** ν (cm^{-1}) 3398, 3019, 1612, 1488, 1265, 746. $[\alpha]^{20}_D$ = + 39.6 (*c* = 0.51, $CHCl_3$)

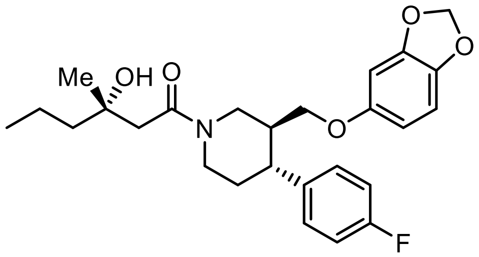

product 3al-ol: Following the procedure described above. Amide **1aj** (109.8mg, 0.25 mmol) was converted to the alcohol product. Analysis of the crude **^{1}H** and **^{13}C NMR** spectra indicates >20:1 dr. Purification by silica gel chromatography gave 72.3 mg (63% yield) of **3al-ol** as a colorless oil. Two sets of peaks were observed in **^{1}H, ^{13}C** and **^{19}F NMR** spectra due to carbamate rotamers, which are assigned to major and minor rotamers, respectively. TLC analysis (hexanes/EtOAc 2:1) R_f= 0.27; **^{1}H NMR** (400 MHz, $CDCl_3$) δ 7.17 – 7.09 (m, 2H, *overlapping*), 7.00 (m, 2H, *overlapping*), 6.63 (t, J = 8.2 Hz, 1H, , *overlapping*), 6.36 (dd, J = 4.7, 2.5 Hz, 1H, *overlapping*), 6.15 (m, 1H, , *overlapping*), 5.89 (d, J = 6.6 Hz, 2H, *overlapping*), 5.21 (s, 1H, *overlapping*), 4.81 (m, 1H, *overlapping*), 4.19 (m, 1H, *minor*), 4.00 (m, 1H, *major*), 3.64 (t, J = 2.1 Hz, 1H, *overlapping*), 3.49 (m, 1H, *overlapping*), 3.22 – 3.04 (m, 1H, *overlapping*), 2.85 (td, J = 11.9, 4.0 Hz, 1H, *overlapping*), 2.77 – 2.62 (m, 2H, *overlapping*), 2.57 – 2.42 (m, 2H, *overlapping*), 2.05 – 1.88 (m, 2H, *overlapping*), 1.76 – 1.66 (m, 1H, *overlapping*), 1.60

– 1.32 (m, 4H, *overlapping*), 1.25 (d, J = 8.1 Hz, 3H, *overlapping*), 0.95 (t, J = 7.1 Hz, 3H, *overlapping*). **^{13}C NMR** (101 MHz, $CDCl_3$) δ 171.5 (*overlapping*), 161.8 (J_1 = 245.5 Hz) (*overlapping*), 154.3 (*minor*), 153.9 (*major*), 148.4 (*major*), 148.3 (*minor*), 142.1 (*major*), 141.9 (*minor*), 138.4 (J_4 = 6.0 Hz) (*overlapping*), 128.9(J_3 = 9.6 Hz) (*minor*), 128.8(J_3 = 9.6 Hz) (*major*), 115.9 (J_2 = 21.2 Hz) (*major*), 115.8 (J_2 = 21.2 Hz) (*minor*), 108.1 (*major*), 108.0 (*minor*), 105.8 (*overlapping*), 101.4 (*major*), 101.2 (*minor*), 98.2 (*minor*), 98.1 (*major*), 71.3 (*overlapping*), 68.7 (*major*), 68.7 (*minor*), 49.2 (*major*), 46.4 (*minor*), 45.1 (*major*), 44.9 (*minor*), 44.8 (*minor*), 44.4 (*major*), 43.9 (*minor*), 42.9 (*major*), 42.2 (*minor*), 42.1 (*major*), 41.5 (*minor*), 41.3 (*major*), 34.6 (*major*), 33.8 (*minor*), 27.2 (*major*), 27.0 (*minor*), 17.5 (*major*), 17.4 (*minor*), 14.8 (*minor*), 14.8 (*major*). **^{19}F NMR** (376 MHz, CDCl3) δ -115.6 (*major*), -115.7 (*minor*). **ESI-HR** calcd for $C_{26}H_{33}FNO_5^+$ ($[M+H]^+$) 458.2337, found 458.2323. **IR** ν (cm^{-1}) 3400, 2979, 1619, 1495, 1267, 732. $[\alpha]^{20}_D$ = - 21.3 (*c* = 1.25, $CHCl_3$).

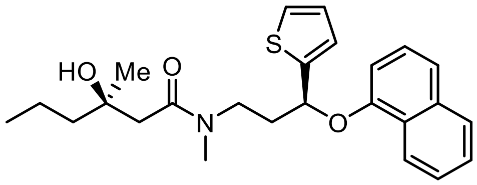

product 3aj-ol: Following the procedure described above. Amide **1aj** (101.8mg, 0.25 mmol) was converted to the alcohol product. Analysis of the crude **^{1}H** and **^{13}C NMR** spectra indicates >20:1 dr. Purification by silica gel chromatography gave 76.1 mg (72% yield) of **3aj-ol** as a colorless oil. Two sets of peaks were observed in **^{1}H** and **^{13}C NMR** spectra due to carbamate rotamers, which are assigned to major and minor rotamers, respectively. TLC analysis (hexanes/EtOAc 2:1) R_f= 0.31; **^{1}H NMR** (400 MHz, $CDCl_3$) δ 8.33 (td, J = 8.2, 6.6, 4.5 Hz, 1H, *overlapping*), 7.88 – 7.65 (m, 1H, *overlapping*), 7.55 – 7.45 (m, 2H, *overlapping*), 7.40 (dd, J = 10.6, 8.2 Hz, 1H, *overlapping*), 7.29 – 7.16 (m, 2H, *overlapping*), 7.11 – 7.02 (m, 1H, *overlapping*), 6.93 (ddd, J = 15.3, 5.0, 3.5 Hz, 1H, *overlapping*), 6.80 (dd, J = 13.1, 7.7 Hz, 1H, *overlapping*), 5.69 (dd, J = 8.0, 4.8 Hz, 1H, *major*), 5.61 (dd, J = 8.4, 4.0 Hz, 1H, *minor*), 5.26 (s, 1H, *major*), 5.21 (s,

1H, *minor*), 3.83 – 3.69 (m, 1H *overlapping*), 3.50 (dddd, J = 40.4, 14.7, 8.3, 5.3 Hz, 1H, *overlapping*), 2.95 (d, J = 2.6 Hz, 3H, *overlapping*), 2.55 – 2.10 (m, 4H, *overlapping*), 1.57 – 1.23 (m, 2H, *overlapping*), 1.17 (s, 3H, *major*), 1.12 (td, J = 6.9, 3.1 Hz, 2H, *overlapping*), 0.91 (m, 3H, *major*), 0.86 (s, 3H, *minor*), 0.82 – 0.71 (m, 3H, *minor*). **^{13}C NMR** (101 MHz, $CDCl_3$) δ 173.4 (*major*), 173.3 (*minor*), 153.1 (*minor*), 152.7 (*major*), 144.6 (*minor*), 144.0 (*major*), 134.8 (*major*), 134.7 (*minor*), 127.9 (*major*), 127.7 (*minor*), 127.0 (*major*), 126.8 (*minor*), 126.7 (*major*), 126.4 (*minor*), 126.1 (*minor*), 125.9 (*major*), 125.8 (*minor*), 125.8 (*major*), 125.7 (*major*), 125.4 (*minor*), 125.2 (*minor*), 125.0 (*major*), 125.0 (*major*), 124.8 (*minor*), 122.0 (*minor*), 121.6 (*major*), 121.3 (*major*), 120.9 (*minor*), 107.1 (*minor*), 106.9 (*major*), 74.5 (*minor*), 72.9 (*major*), 71.2 (*minor*), 71.0 (*major*), 46.1 (*major*), 45.3 (*minor*), 44.8 (*minor*), 44.6 (*major*), 41.8 (*minor*), 41.0 (*major*), 37.7 (*major*), 36.6 (*minor*), 36.3 (*minor*), 33.2 (*major*), 27.0 (*minor*), 26.5 (*major*), 17.3 (*minor*), 17.0 (*major*), 14.8 (*minor*), 14.7 (*major*). **ESI-HR** calcd for $C_{25}H_{32}NO_3S^+$ ($[M+H]^+$) 426.2097, found 426.2091. **IR** ν (cm^{-1}) 3401, 3052, 2959, 1620, 1264, 737. $[\alpha]^{20}_D$ = + 112.3 (*c* = 1.07, $CHCl_3$).

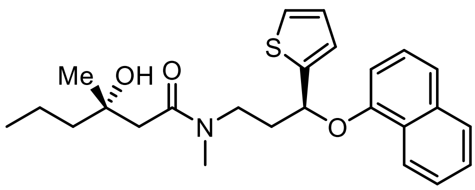

product 3am-ol: Following the procedure described above. Amide **1aj** (101.8mg, 0.25 mmol) was converted to the alcohol product. Analysis of the crude **^{1}H** and **^{13}C NMR** spectra indicates >20:1 dr. Purification by silica gel chromatography gave 73.5 mg (69% yield) of **3am-ol** as a colorless oil. Two sets of peaks were observed in **^{1}H** and **^{13}C NMR** spectra due to carbamate rotamers, which are assigned to major and minor rotamers, respectively. TLC analysis (hexanes/EtOAc 2:1) R_f= 0.31; **^{1}H NMR** (400 MHz, $CDCl_3$) δ 8.41 – 8.25 (m, 1H, *overlapping*), 7.78 (t, J = 7.8, Hz, 1H, *overlapping*), 7.50

(m, 2H, *overlapping*), 7.41 (t, J = 9.1 Hz, 1H, *overlapping*), 7.33 – 7.23 (m, 2H, *major*), 7.23 – 7.17 (m, 2H, *minor*), 7.08 (t, J = 3.2 Hz, 1H, *overlapping*), 6.94 (m, 1H, *overlapping*), 6.81 (m, 1H, *overlapping*), 5.69 (dd, J = 8.2, 4.1 Hz, 1H, *minor*), 5.63 (dd, J = 8.2, 4.1 Hz, 1H, *major*), 5.27 (s, 1H, *minor*), 5.17 (s, 1H, *major*), 3.81 – 3.65 (m, 1H, *overlapping*), 3.54 (m, 1H, *overlapping*), 2.96 (s, 3H), 2.53 – 2.15 (m, 4H, *overlapping*), 1.55 – 1.29 (m, 2H, *overlapping*), 1.25 – 0.96 (m, 5H, *overlapping*), 0.91 (t, J = 7.5 Hz, 3H, , *major*), 0.75 (t, J = 6.9 Hz, 3H, *minor*). **^{13}C NMR** (101 MHz, $CDCl_3$) δ 173.4 (*minor*), 173.3 (*major*), 153.1 (*major*), 152.7 (*minor*), 144.7 (*minor*), 144.1 (*major*), 134.8 (*major*), 134.7 (*minor*), 127.9 (*major*), 127.7 (*minor*), 127.0 (*major*), 126.8 (*minor*), 126.7 (*major*), 126.5 (*minor*), 126.2 (*minor*), 125.9 (*major*), 125.8 (*minor*), 125.8 (*minor*), 125.7 (*major*), 125.4 (*minor*), 125.2 (*major*), 125.0 (*major*), 125.0 (*major*), 124.9 (*minor*), 122.1 (*minor*), 121.6 (*major*), 121.3 (*major*), 121.0 (*minor*), 107.1 (*minor*), 106.9 (*major*), 74.5 (*minor*), 73.0 (*major*), 71.3 (*major*), 71.1 (*minor*), 46.3 (*major*), 45.3 (*minor*), 44.8 (*minor*), 44.4 (*major*), 41.8 (*major*), 41.2 (*minor*), 37.8 (*major*), 36.7 (*minor*), 36.4 (*minor*), 33.3 (*major*), 27.1 (*minor*), 26.8 (*major*), 17.4 (*minor*), 17.0 (*major*), 14.8 (*minor*), 14.7 (*major*). **ESI-HR** calcd for $C_{25}H_{32}NO_3S^+$ ($[M+H]^+$) 426.2097, found 426.2092. **IR** ν (cm^{-1}) 3400, 3054, 2960, 1619, 1264, 732. $[\alpha]^{20}_D$ = + 49.0 (*c* = 1.0, $CHCl_3$).

Synthetic Transformation of Boration Products

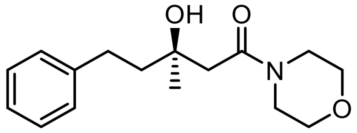

product 4[2]: To a solution of hydroboration product **3i** (77.4 mg, 0.20 mmol) in THF (2.0 mL) and water (2.0 mL) was added sodium perborate trihydrate (4.0 equiv). The reaction was stirred at room temperature for 12 h. Ethyl acetate (20 mL) was added, and the mixture was washed with water (10 mL), brine (10 mL) and dried over Na_2SO_4. After removal of the solvent, the residue was purified by column chromatography on silica gel with EtOAc/hexanes mixture as eluent to give 43.6 mg (79% yield) of **4** as a

colorless oil. TLC analysis (hexanes/EtOAc 1:1) R_f = 0.16; **^{1}H NMR** (400 MHz, $CDCl_3$) δ 7.35 – 7.13 (m, 5H), 5.16 (s, 1H), 3.65 (tt, *J* = 8.3, 4.5 Hz, 6H), 3.42 (td, *J* = 4.5, 2.0 Hz, 2H), 2.74 (dddd, *J* = 39.6, 13.5, 11.5, 5.4 Hz, 2H), 2.54 – 2.34 (m, 2H), 1.86 (dddd, *J* = 37.0, 13.6, 11.5, 5.6 Hz, 2H), 1.32 (s, 3H). **^{13}C NMR** (101 MHz, $CDCl_3$) δ 171.5, 142.6, 128.5, 128.5, 125.9, 71.1, 66.9, 66.6, 46.1, 44.1, 41.8, 41.5, 30.4, 27.1. **ESI-HR** calcd for $C_{16}H_{24}NO_3^+$ ($[M+H]^+$) 278.1751, found 278.1743. **IR** ν (cm^{-1}) 3406, 2917, 2857, 1613, 1113, 699. $[\alpha]^{20}_D$ = + 27.0 (*c* = 0.47, $CHCl_3$) for a 91% *ee* sample. The enantiomeric purity of this compound was determined by HPLC analysis in comparison with authentic racemic material (Chiralpak ID, 90.0:10.0 hexanes:*i*-PrOH, 1.0 mL/min, 208 nm): t_{major} = 26.532 min, t_{minor} =27.789 min.

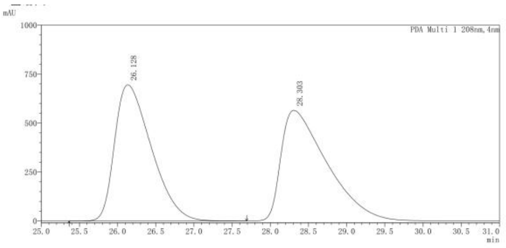

PDA Ch1 208nm

峰号	保留时间	峰宽(高度 50%)	高度	面积	面积%
1	26.128	0.521	695257	23242731	49.543
2	28.303	0.643	565112	23671722	50.457

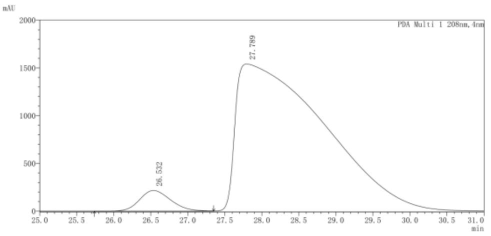

PDA Ch1 208nm

峰号	保留时间	峰宽(高度 50%)	高度	面积	面积%
1	26.532	0.451	216404	6318704	4.690
2	27.789	1.368	1541401	128394386	95.310

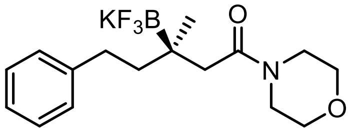

product 5[3]: In a 25 mL reaction tube with a magnetic stir bar, the hydroboration product **3i** (77.4 mg, 0.20 mmol) was dissolved in MeCN (2.0 mL). Water (2.0 mL) and KHF_2 (0.80 mmol, 4.0 equiv) were added. The reaction mixture was stirred at room temperature for 12 h, concentrated, and azeotroped with MeOH (5 mL ×3). The resulting material was then placed on high vacuum overnight. The crude product was extracted with hot acetone, filtered and then concentrated. Et_2O (10 mL) was added to the crude material, and the mixture was sonicated for 30 min. After filtration, the trifluoroborate salt was obtained as a white solid (60.8 mg, 83% yield). **^{1}H NMR** (400 MHz, $(CD_3)_2CO$) δ 7.19 (d, *J* = 4.4 Hz, 4H), 7.07 (m, 1H), 3.52 (s, 8H), 2.74 – 2.64 (m, 2H), 2.34 (d, *J* = 13.0 Hz, 1H), 2.24 (d, *J* = 13.0 Hz, 1H), 1.63 – 1.55 (m, 2H), 0.90 (s, 3H).**^{13}C NMR** (101 MHz, $(CD_3)_2CO$) δ174.0, 146.8, 129.3, 128.7, 125.5, 67.6, 67.5, 48.1, 43.4, 42.4,

39.6, 32.8, 23.4. **^{19}F NMR** (376 MHz, $(CD_3)_2CO$) δ -148.57. **IR** ν (cm^{-1}) 2937, 2853, 1619, 1114, 696. $[\alpha]^{20}_D$ = - 24.0 (*c* = 0.38, Acetone) **ESI-HR** calcd for $C_{16}H_{22}BF_3NO_2^-$ ($[M-K^+]^-$) 328.1704, found 328.1701.

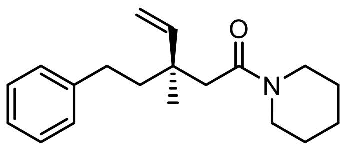

product 6[4]: To a solution of hydroboration product **3e** (77.0 mg, 0.20 mmol) in THF (2.0 mL) was added vinylmagnesium bromide (4.0 equiv). The resultant mixture was stirred as room temperature for 1 h. To the above solution was cooled to -78 °C and iodine (4.0 equiv) in methanol (1.0 mL) was added. The reaction mixture was stirred at the -78 °C for 1 h followed by addition of a solution of NaOMe (8.0 equiv) in methanol (2.0 mL). After warming to room temp, the resultant mixture was stirred for another 1.5 h. It was then diluted with pentane (20 mL) and washed with a solution of $Na_2S_2O_3$ (3.0 mL). Ethyl acetate (20 mL) was added, and the mixture was washed with water (10 mL), brine (10 mL) and dried over Na_2SO_4. After removal of the solvent, the reaction mixture was concentrated. Purification by silica gel chromatography gave 26.1 mg (46% yield) of **6** as a white solid. TLC analysis (hexanes/EtOAc 4:1) R_f= 0.23; **^{1}H NMR** (400 MHz, $CDCl_3$) δ 7.30 – 7.12 (m, 5H), 5.94 (dd, *J* = 17.5, 10.8 Hz, 1H), 5.13 – 4.92 (m, 2H), 3.55 (t, *J* = 5.5 Hz, 2H), 3.40 (t, *J* = 5.4 Hz, 2H), 2.56 (t, *J* = 8.6 Hz, 2H), 2.47 – 2.36 (m, 2H), 1.81 (dp, *J* = 9.9, 6.3 Hz, 2H), 1.57 (dq, *J* = 35.5, 6.4, 5.8 Hz, 6H), 1.22 (s, 3H). **^{13}C NMR** (101 MHz, $CDCl_3$) δ 169.4, 146.3, 143.1, 128.5, 128.4, 125.7, 112.2, 47.8, 43.0, 42.7, 42.7, 40.0, 30.9, 26.7, 25.8, 24.7, 23.3. **ESI-HR** calcd for $C_{19}H_{27}KNO^+$ ($[M+K]^+$) 324.1724, found 324.1712. **IR** ν (cm^{-1}) 2930, 2855, 1632, 1444, 1140, 700. **M. P.** 40-42 °C. $[\alpha]^{20}_D$ = + 21.1 (*c* = 0.27, $CHCl_3$) for a 96% *ee* sample. The enantiomeric purity of this compound was determined by HPLC analysis in comparison with authentic racemic material (Chiralpak IC, 92.0:8.0 hexanes:*i*-PrOH, 1.0 mL/min, 208 nm): t_{major} = 16.846 min, t_{minor} =18.563 min.

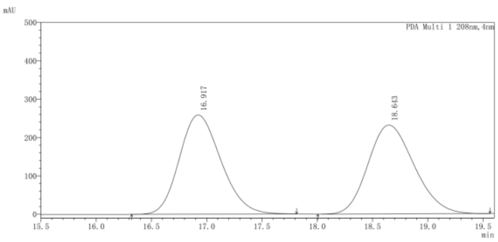

PDA Ch1 208nm

峰号	保留时间	峰宽(高度 50%)	高度	面积	面积%
1	16.917	0.406	258618	6774781	50.221
2	18.643	0.452	231609	6715240	49.779

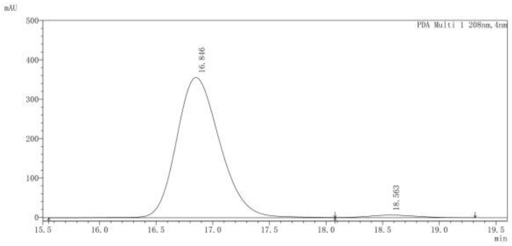

PDA Ch1 208nm

峰号	保留时间	峰宽(高度 50%)	高度	面积	面积%
1	16.846	0.409	356453	9515774	97.882
2	18.563	0.461	6797	205924	2.118

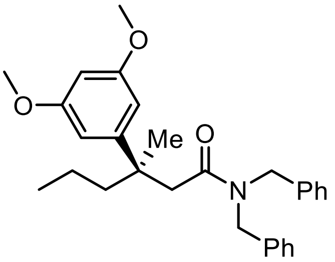

Product 7[5]: In a 25 mL flame-dried Schlenk tube with a magnetic stir bar, the 1-bromo-3,5-dimethoxybenzene (102 mg, 0.470 mmol, 1.4 equiv) and THF (2.0 mL) were added under Ar atmosphere. Then *t*-BuLi (0.94 mmol, 2.8 equiv) was added dropwise at -78 °C. The reaction mixture was stirred for 1 hour at the same temperature, and then **3a** (146 mg, 0.330 mmol, 1.0 equiv) was added dropwise as a solution in THF (1.0 mL). After stirring at the -78 °C for another 1 hour, a solution of NBS (238 mg, 1.33 mmol, 4.0 equiv) in THF (2.0 mL) was added dropwise. The reaction mixture was stirred at -78 °C for 1 hour. Then the reaction mixture was quenched with saturated $Na_2S_2O_3$ solution (2.0 mL). Ethyl acetate (20 mL) was added, and the mixture was washed with water (10 mL), brine (10 mL) and dried over Na_2SO_4. After removal of the solvent, the reaction mixture was concentrated. Purification by silica gel chromatography gave 69.1 mg (47% yield) of **7** as a colorless oil. TLC analysis (hexanes/EtOAc 4:1) R_f = 0.48; **^{1}H NMR** (400 MHz, $CDCl_3$) δ 7.42 – 7.16 (m, 6H), 7.12 – 6.96 (m, 4H), 6.49 (d, J = 2.2 Hz, 2H), 6.32 (t, J = 2.2 Hz, 1H), 4.73 (d, J = 14.9 Hz, 1H), 4.31 (dd, J = 16.1, 7.0 Hz, 2H), 4.14 (d, J = 17.3 Hz, 1H), 3.75 (s, 6H), 2.79 (d, J = 14.6 Hz, 1H), 2.58 (d, J = 14.6 Hz, 1H), 1.87 – 1.67 (m, 2H), 1.55 (s, 3H), 1.26 – 1.16 (m, 1H), 1.05 – 0.93 (m, 1H), 0.83 (t, J = 7.2 Hz, 3H).**^{13}C NMR** (101 MHz, $CDCl_3$)δ 171.8, 160.8, 150.1, 137.6, 136.9, 129.0, 128.6, 128.1, 127.6, 127.2, 126.4, 105.1, 97.3, 55.3, 50.3, 48.2, 45.4, 45.4, 41.5, 24.5, 17.5, 14.8.**ESI-HR** calcd for $C_{29}H_{36}NO_3^+$ ($[M+H]^+$) 446.2690, found 446.2687.

IR ν (cm^{-1}) 3010, 2935, 1615, 1216, 750. $[\alpha]^{20}_{D}$ = + 72.2 (*c* = 0.65, $CHCl_3$) for a 96% *ee* sample. The enantiomeric purity of this compound was determined by HPLC analysis in comparison with authentic racemic material (Chiralpak OD, 94.0:6.0 hexanes:*i*-PrOH, 1.0 mL/min, 208 nm): t_{major} = 7.718 min, t_{minor} =6.656 min.

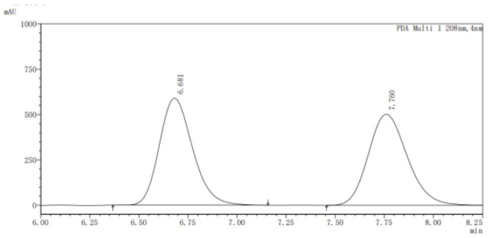

PDA Ch1 208nm

峰号	保留时间	峰宽(高度 50%)	高度	面积	面积%
1	6.681	0.182	588810	6957639	50.055
2	7.760	0.213	501481	6942323	49.945

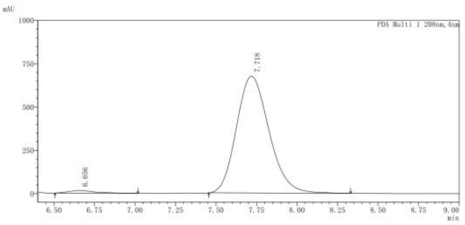

PDA Ch1 208nm

峰号	保留时间	峰宽(高度 50%)	高度	面积	面积%
1	6.656	0.168	14726	171134	1.785
2	7.718	0.215	673774	9415976	98.215

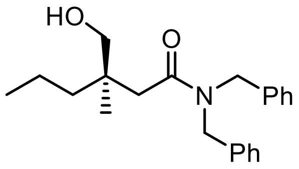

product 8[6]: In a 10 mL flame-dried Schlenk tube with a magnetic stir bar, the $ClCH_2I$ (176 mg, 1.00 mmol, 5.0 equiv), **3a** (87.0 mg, 0.20 mmol, 1.0 equiv) and THF (2.0 mL) was added under Ar atmosphere. Then *n*-BuLi (1.0 mmol, 5.0 equiv) was added dropwise at -78 ℃. The reaction mixture was stirred for 10 min at the same temperature, and then the reaction was stirred at the room temperature for another 1 hour. To the reaction mixture was added 3 M NaOH (2.0 mL) and methanol (2.0 mL), and it was cooled to 0 ℃. Then 30% aqueous H_2O_2 (0.5 mL) was added dropwise. The reaction was stirred at room temperature for 3 h. Ethyl acetate (20 mL) was added, and the mixture was washed with water (10 mL), brine (10 mL) and dried over Na_2SO_4. After removal of the solvent, the reaction mixture was concentrated. Purification by silica gel chromatography gave 30.4 mg (45% yield) of **8** as a colorless oil. TLC analysis (hexanes/EtOAc 2:1) R_f = 0.51; **^{1}H NMR** (400 MHz, $CDCl_3$) δ 7.51 – 7.08 (m, 10H), 4.72 (t, J = 13.9 Hz, 2H), 4.59 – 4.39 (m, 3H), 3.59 – 3.37 (m, 2H), 2.59 – 2.37 (m, 2H), 1.39 – 1.17 (m, 4H), 0.98 – 0.80 (m, 6H). **^{13}C NMR** (101 MHz, $CDCl_3$) δ 174.1, 137.2, 136.3, 129.2, 128.8, 128.4, 128.0, 127.7, 126.5, 70.5, 51.0, 48.6, 41.4, 41.0, 39.4, 22.8,

17.0, 15.0. **ESI-HR** calcd for $C_{22}H_{30}NO_2^+$ ($[M+H]^+$) 340.2271, found 340.2269. **IR** ν (cm^{-1}) 3396, 2956, 1615, 1417, 1265, 733. $[\alpha]^{20}_D$ = + 137.3 (*c* = 0.33, $CHCl_3$). for a 96% *ee* sample. The enantiomeric purity of this compound was determined by HPLC analysis in comparison with authentic racemic material (Chiralpak AD, 84.0:16.0 hexanes:*i*-PrOH, 1.0 mL/min, 208 nm): t_{major} = 7.342 min, t_{minor} =6.908 min.

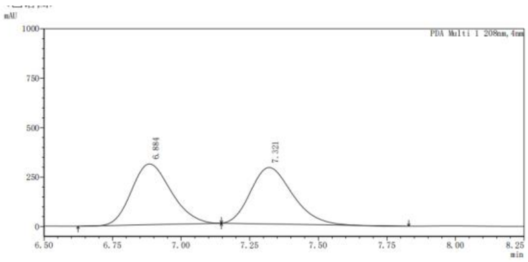

PDA Ch1 208nm

峰号	保留时间	峰宽(高度 50%)	高度	面积	面积%
1	6.884	0.156	307223	3088938	50.449
2	7.321	0.165	285376	3033945	49.551

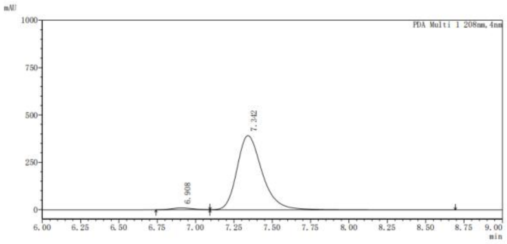

PDA Ch1 208nm

峰号	保留时间	峰宽(高度 50%)	高度	面积	面积%
1	6.908	0.149	9097	85048	1.849
2	7.342	0.170	392917	4515727	98.151

Substrates and products for control experiments

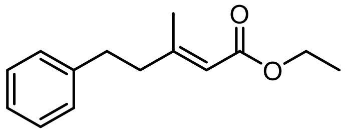

Ester 9: colorless oil. TLC analysis (hexanes/EtOAc 25:1) R_f = 0.42; **^{1}H NMR** (400 MHz, $CDCl_3$) δ 7.33 – 7.07 (m, 5H), 5.68 (q, *J* = 1.3 Hz, 1H), 4.14 (q, *J* = 7.2 Hz, 2H), 2.81 – 2.73 (m, 2H), 2.43 (ddd, *J* = 9.1, 6.0, 1.2 Hz, 2H), 2.20 (d, *J* = 1.3 Hz, 3H), 1.26 (t, *J* = 7.1 Hz, 3H). **^{13}C NMR** (101 MHz, $CDCl_3$) δ 166.8, 159.0, 141.2, 128.5, 128.3, 126.2, 116.1, 59.6, 42.8, 34.0, 19.0, 14.4. **ESI-HR** calcd for $C_{14}H_{18}NaO_2^+$ ($[M+Na]^+$) 241.1199, found 241.1191. **IR** ν (cm^{-1}) 2982, 2932, 1711, 1647, 1495, 697.

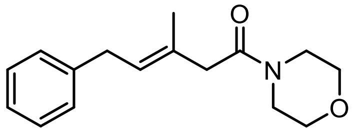

amide 10: pale yellow oil. TLC analysis (hexanes/EtOAc 1:1) R_f = 0.27; **^{1}H NMR** (400 MHz, $CDCl_3$) δ 7.28 (t, *J* = 7.4 Hz, 2H), 7.23 – 7.13 (m, 3H), 5.52 – 5.33 (m, 1H), 3.61 (q, *J* = 3.1, 2.7 Hz, 4H), 3.51 (t, *J* = 4.7 Hz, 2H), 3.44 – 3.37 (m, 4H), 3.11 (s, 2H), 1.81 (s, 3H). **^{13}C NMR** (101 MHz, $CDCl_3$) δ 169.7, 141.0, 130.3, 128.6, 128.4, 127.0, 126.1,

67.0, 66.7, 46.4, 44.7, 42.1, 34.5, 16.6. **ESI-HR** calcd for $C_{16}H_{22}NO_2^+$ ($[M+H]^+$) 260.1645, found 260.1639. **IR** ν (cm^{-1}) 2919, 2864, 1634, 1494, 1113, 699.

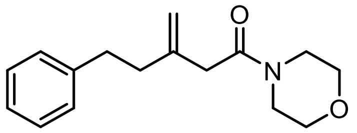

amide 11: pale yellow oil. TLC analysis (hexanes/EtOAc 1:1) R_f = 0.29; **^{1}H NMR** (400 MHz, $CDCl_3$) δ 7.32 – 7.16 (m, 5H), 4.98 (d, *J* = 1.7 Hz, 1H), 4.85 (s, 1H), 3.71 – 3.54 (m, 6H), 3.37 – 3.28 (m, 2H), 3.11 (s, 2H), 2.81 (dd, *J* = 9.2, 6.7 Hz, 2H), 2.42 (t, *J* = 8.0 Hz, 2H). **^{13}C NMR** (101 MHz, $CDCl_3$) δ 169.3, 142.9, 141.6, 128.5, 128.5, 126.1, 112.9, 67.0, 66.8, 46.5, 42.0, 42.0, 37.7, 34.1. **ESI-HR** calcd for $C_{16}H_{22}NO_2^+$ ($[M+H]^+$) 260.1645, found 260.1637. **IR** ν (cm^{-1}) 2919, 2856, 1632, 1425, 1433, 1114, 698.

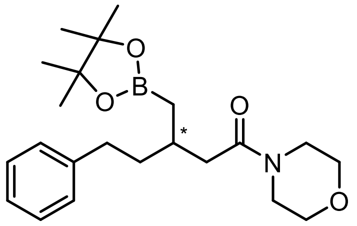

Boration product 11b: Following the general procedure, amide **9** (64.8 mg, 0.25 mmol) was converted to the boration product. Purification by silica gel chromatography gave 67.9 mg (70% yield) of **11b** as a colorless oil. TLC analysis (hexanes/EtOAc 1:1) R_f = 0.28; **^{1}H NMR** (400 MHz, $CDCl_3$) δ 7.27 – 7.02 (m, 5H), 3.68 – 3.23 (m, 8H), 2.64 (ddd, *J* = 13.6, 10.1, 5.5 Hz, 1H), 2.51 (ddd, *J* = 13.6, 10.0, 6.4 Hz, 1H), 2.36 (dd, *J* = 14.2, 6.6 Hz, 1H), 2.20 (dd, *J* = 14.2, 7.6 Hz, 1H), 2.03 (hept, *J* = 6.7 Hz, 1H), 1.73 – 1.62 (m, 1H), 1.58 – 1.47 (m, 1H), 1.17 (s, 12H), 0.91 (dd, *J* = 15.9, 5.5 Hz, 1H), 0.80 (dd, *J* = 15.9, 7.4 Hz, 1H). **^{13}C NMR** (101 MHz, $CDCl_3$) δ 171.5, 142.7, 128.5, 128.4, 125.8, 83.2, 67.0, 46.4, 42.1, 40.1, 38.3, 33.3, 31.5, 25.0, 25.0. **ESI-HR** calcd for $C_{22}H_{35}BNO_4^+$ ($[M+H]^+$) 388.2654, found 388.2646. $[\alpha]^{20}_D$ = - 8.4 (*c* = 0.81, $CHCl_3$) for a 76% *ee* sample. **IR** ν (cm^{-1}) 2976, 1642, 1144, 755. The enantiomeric purity of this compound was determined by HPLC analysis in comparison with authentic racemic material (Chiralpak AD, 98.0:2.0 hexanes:*i*-PrOH, 1.0 mL/min, 208 nm): t_{major} =31.069 min, t_{minor} =28.930 min.

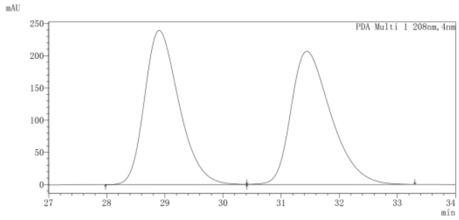

<Peak Results>
PDA Ch1 208nm

Index	Time/min	Height/mAU	Quantity/Area	Area %/%
1	28.902	238966	9864726	49.946
2	31.442	206430	9886070	50.054

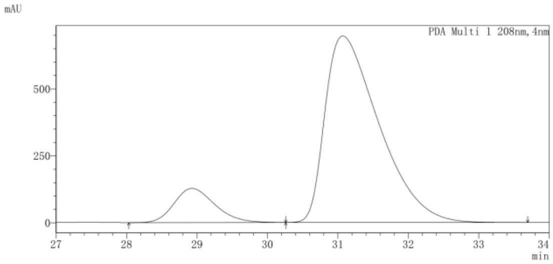

<Peak Results>
PDA Ch1 208nm

Index	Time/min	Height/mAU	Quantity/Area	Area %/%
1	28.930	127799	5172159	12.206
2	31.069	697061	37200149	87.794

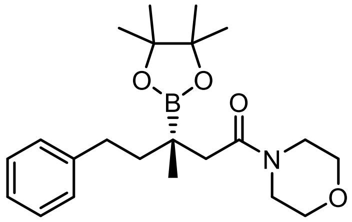

Boration product (*R*)-3i: Following the general procedure, amide **11** (64.8 mg, 0.25 mmol) was converted to the boration product. Purification by silica gel chromatography gave 11.6 mg (12% yield) of **(*R*)-3i** as a colorless oil. For a 70% *ee* sample. The enantiomeric purity of this compound was determined by HPLC analysis in comparison with authentic racemic material (Chiralpak AD, 99.0:1.0 hexanes:*i*-PrOH, 1.0 mL/min, 208 nm): t_{major} =9.6069 min, t_{minor} =11.761 min.

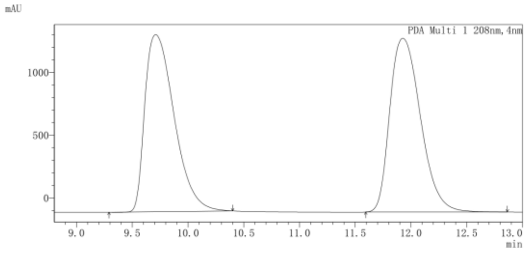

<Peak Results>
PDA Ch1 208nm

Index	Time/min	Height/mAU	Quantity/Area	Area %/%
1	9.710	1409752	25786618	49.334
2	11.926	1383030	26483345	50.666

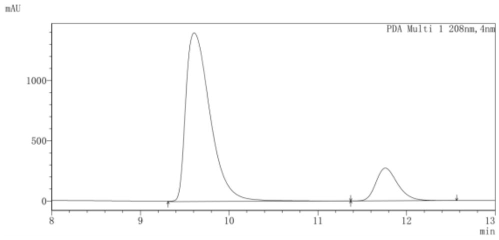

<Peak Results>
PDA Ch1 208nm

Index	Time/min	Height/mAU	Quantity/Area	Area %/%
1	9.606	1399338	26697618	85.224
2	11.761	271748	4628954	14.776

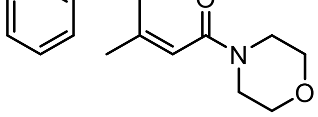

Amide *Z*-1i: Pale yellow oil**.** TLC analysis (hexanes/EtOAc 1:1) R_f = 0.28 **^{1}H NMR** (400 MHz, $CDCl_3$) δ 7.32 – 7.10 (m, 5H), 5.77 (d, *J* = 1.7 Hz, 1H), 3.67 – 3.50 (m, 6H), 3.38 – 3.29 (m, 2H), 2.84 – 2.76 (m, 2H), 2.72 – 2.61 (m, 2H), 1.87 (d, *J* = 1.4 Hz, 3H). **^{13}C NMR** (101 MHz, $CDCl_3$) δ 166.8, 149.7, 141.8, 128.5, 128.4, 126.0, 118.5, 77.5,

77.2, 76.8, 66.9, 66.8, 46.5, 41.6, 35.5, 34.2, 23.9. **ESI-HR** calcd for $C_{16}H_{22}NO_2^+$ ($[M+H]^+$) 260.1645, found 260.1639. **IR** ν (cm^{-1}) 2971,2854,1621,1425,1113,698.

General Information for Computation

All calculations were performed with the Gaussian 09 program. (Full citation of Gaussian 09: Frisch, M. J.; Trucks, G. W.; Schlegel, H. B.; Scuseria, G. E.; Robb, M. A.; Cheeseman, J. R.; Scalmani, G.; Barone, V.; Mennucci, B.; Petersson, G. A.; Nakatsuji, H.; Caricato, M.; Li, X.; Hratchian, H. P.; Izmaylov, A. F.; Bloino, J.; Zheng, G.; Sonnenberg, J. L.; Hada, M.; Ehara, M.; Toyota, K.; Fukuda, R.; Hasegawa, J.; Ishida, M.; Nakajima, T.; Honda, Y.; Kitao, O.; Nakai, H.; Vreven, T.; Montgomery, J. A., Jr.; Peralta, J. E.; Ogliaro, F.; Bearpark, M.; Heyd, J. J.; Brothers, E.; Kudin, K. N.; Staroverov, V. N.; Keith, T.; Kobayashi, R.; Normand, J.; Raghavachari, K.; Rendell, A.; Burant, J. C.; Iyengar, S. S.; Tomasi, J.; Cossi, M.; Rega, N.; Millam, J. M.; Klene, M.; Knox, J. E.; Cross, J. B.; Bakken, V.; Adamo, C.; Jaramillo, J.; Gomperts, R.; Stratmann, R. E.; Yazyev, O.; Austin, A. J.; Cammi, R.; Pomelli, C.; Ochterski, J. W.; Martin, R. L.; Morokuma, K.; Zakrzewski, V. G.; Voth, G. A.; Salvador, P.; Dannenberg, J. J.; Dapprich, S.; Daniels, A. D.; Farkas, O.; Foresman, J. B.; Ortiz, J. V.; Cioslowski, J.; Fox, D. J.; *Gaussian 09, revision B.01*; Gaussian Inc.: Wallingford, CT, 2010.) Density functional theory calculations using the M06 functional ((a) Zhao, Y.; Truhlar, D. G. *Theor. Chem. Acc.* **2008**, *120*, 215-241. (b) Zhao, Y.; Truhlar, D. G. *Acc. Chem. Res.* **2008**, *41*, 157-167.) Geometry optimizations were conducted with the Gaussian 09 software package, B3LYP functional, and LANL2DZ basis set for Rh and 6-31g(d,p) basis set for all other atoms. Single-point energy calculations were conducted with the M06 functional and SDD basis set for Rh and the 6-311++g(d,p) basis set for all other atoms, along with the SMD *o*-$C_6H_4Cl_2$ solvent correction. Thermal corrections were applied to the optimized geometries to provide Gibbs free energies.

The energy profile of catalytic hydroboration using DIOP ligand was computed. Extensive conformational searches were conducted for all intermediates and transition states, and the lowest energy conformers are shown in Supplementary Figure 3.

Computational results revealed that migratory insertion of the alkene into rhodium hydride has the highest activation barrier. Others steps have much lower activation

energies. To reveal the origin of enantioselectivity, the possible transition state structures for the migratory insertion were considered. The structures and energies were summarized in Supplementary Figure 4 and 5. The transition states leading to the *S*-enantiomer have activation energies of 21.0, 21.3 kcal/mol, respectively, while the transition states leading to the *R*-enantiomer have activation energies of 26.2, 23.4 kcal/mol, respectively. The lower activation energy of **TS-2** is consistent with the major enantiomer observed in the experiments.

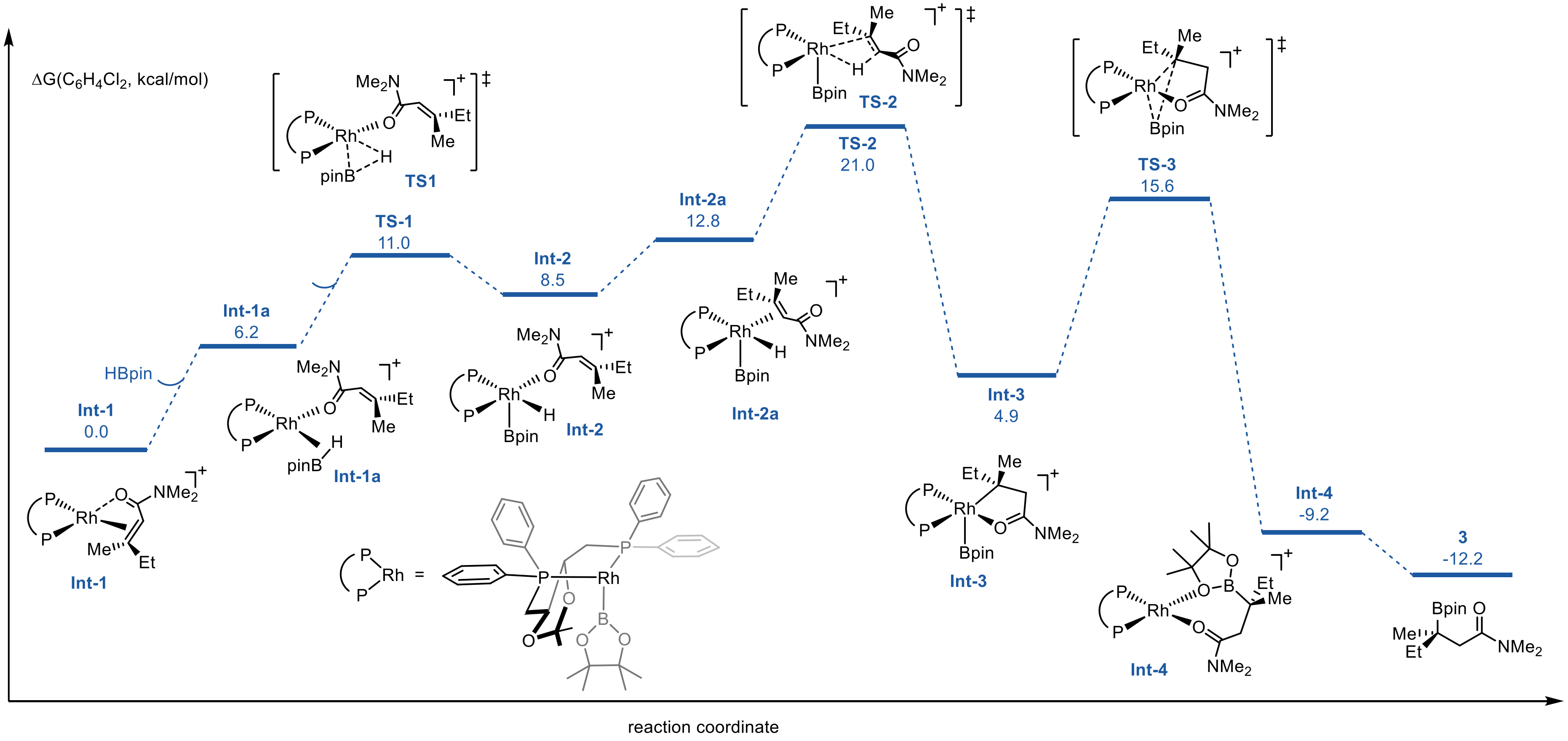

Supplementary Figure 3. Energy profile for Rh-DIOP catalyzed hydroboration. Gibbs free energies in *o*-$C_6H_4Cl_2$ ($\Delta G_{solv298K}$) are reported.

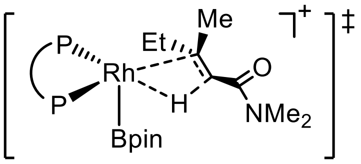

TS-2
21.0 kcal/mol

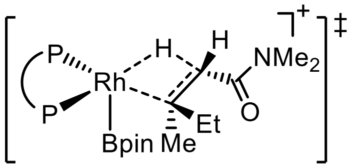

TS-2-2
21.3 kcal/mol

Supplementary Figure 4. transition states of migratory insertion leading to the correct enantiomer

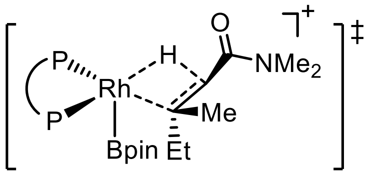

TS-2-3
26.2 kcal/mol

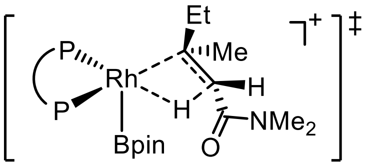

TS-2'
23.4 kcal/mol

Supplementary Figure 5. transition states of migratory insertion leading to the opposite enantiomer

Coordinates and Energies of Stationary Points

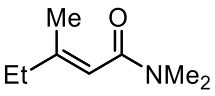

G_{sol} = -443.4854871 Hatree

C	-1.46005500	0.04045000	-0.28059000
C	-0.24654000	-0.42012700	0.08406300
C	-2.65278100	-0.89316800	-0.24957600
H	-2.32343600	-1.90707500	0.00379900
H	-3.08562500	-0.94526300	-1.25918300
H	-0.17645100	-1.46022100	0.39104200
C	0.99863500	0.39548100	0.15869800
O	0.97994200	1.61512500	0.34632100
N	2.19605700	-0.28810400	0.05287700
C	3.43759900	0.43358800	0.28372200
H	4.02006700	0.52504800	-0.64310000
H	4.05277800	-0.09477400	1.02324500
H	3.19404100	1.42945700	0.64949400
C	-1.74079100	1.44847700	-0.73919900
H	-2.22567700	2.01917300	0.06252000
H	-0.83036400	1.98410700	-1.00065700
H	-2.43563900	1.43871800	-1.58791700
C	-3.75397100	-0.45553900	0.73391900
H	-4.58136000	-1.17217500	0.72629100
H	-3.36488600	-0.39768400	1.75558800
H	-4.16206100	0.52556000	0.47361200
C	2.34054100	-1.67148000	-0.36753700
H	2.60879300	-2.33262700	0.46918300
H	1.42450200	-2.03911300	-0.82773000
H	3.13756500	-1.74349500	-1.11790600

HBpin

G_{sol} = -411.5602608 Hatree

C	0.78913600	-0.19125300	0.04531000
C	-0.78913700	-0.19124900	-0.04531600
B	-0.00000100	1.94515800	-0.00003800
H	-0.00001900	3.13323800	-0.00000800
O	-1.08513300	1.20021700	-0.36874600
O	1.08514700	1.20023000	0.36865200
C	1.47927800	-0.48198400	-1.29370300
H	2.54701000	-0.26824400	-1.19527400
H	1.36273900	-1.52889800	-1.58915600
H	1.08169700	0.15079900	-2.09199600
C	1.37896900	-1.07502200	1.14266000
H	1.13832900	-2.12875000	0.96668800
H	2.46787100	-0.97331800	1.14742100
H	1.01097000	-0.79345900	2.13077600
C	-1.37900500	-1.07509800	-1.14258700
H	-1.13832200	-2.12880600	-0.96656200
H	-2.46791100	-0.97343400	-1.14729700
H	-1.01107200	-0.79358100	-2.13074000
C	-1.47925200	-0.48188300	1.29372800
H	-2.54696700	-0.26803800	1.19534100
H	-1.36280100	-1.52880400	1.58919400
H	-1.08157300	0.15087000	2.09199700

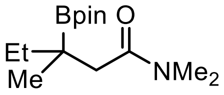

G_{sol} = -855.0651241 Hatree

C	0.66940700	1.47222100	0.27748200
B	-0.60124900	0.52587900	0.14513400
H	1.79834700	0.68042600	-1.40916600
C	0.35387200	2.74890000	-0.56241800
H	-0.55054300	3.21277700	-0.14566200
H	0.09147700	2.43956100	-1.58241100
C	1.90282600	0.75297000	-0.31765700
H	2.81306300	1.33376800	-0.12719300
C	2.06325200	-0.66497000	0.23060100
O	1.15401300	-1.18662600	0.88377700
N	3.22797500	-1.34027500	-0.02802300
C	3.40755900	-2.68379000	0.50542100
H	4.32300900	-2.73437100	1.10803100
H	3.49074000	-3.41625800	-0.30789600
H	2.54718000	-2.92824700	1.12524200
C	-2.11925100	-0.89383900	-0.80955500
C	-2.72630800	-0.24662500	0.50192500
O	-0.94918100	-0.06701500	-1.05066500
O	-1.57281400	0.39369600	1.10741600
C	-3.74536500	0.86707400	0.22140400
H	-3.96536600	1.38740100	1.15786300
H	-4.68293900	0.47068100	-0.18033900
H	-3.34737600	1.60099900	-0.48510900
C	-3.01907600	-0.82099900	-2.04326400
H	-3.93966200	-1.39482200	-1.89194400
H	-2.49363400	-1.24834000	-2.90237900
H	-3.28630500	0.20784700	-2.29141000

C	-3.30460200	-1.24191200	1.50750700
H	-4.15177400	-1.78753700	1.07806900
H	-3.66163800	-0.70287700	2.38994300
H	-2.55200600	-1.96137200	1.83391200
C	-1.61993600	-2.33135900	-0.60937900
H	-1.08313300	-2.64406000	-1.51011000
H	-2.44950600	-3.02684100	-0.44793900
H	-0.92445800	-2.39268300	0.22987300
C	0.92031900	1.83637500	1.75528700
H	1.06903700	0.93587500	2.35598400
H	1.80458100	2.47475000	1.87392200
H	0.06090700	2.36935100	2.17417400
C	1.45434000	3.81820300	-0.63671600
H	2.36674400	3.43795700	-1.10902900
H	1.11430400	4.67252000	-1.23198800
H	1.72587300	4.19774900	0.35329100
C	4.32504600	-0.84367000	-0.84158300
H	4.07684500	0.10788400	-1.30608400
H	5.23402700	-0.71098000	-0.23916800
H	4.55397600	-1.56054000	-1.64053900

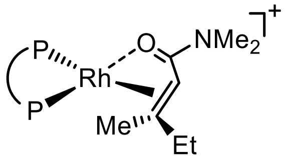

G_{sol} = -2586.55418979 Hatree

Rh	0.58054300	0.82035700	-0.05253200
P	-1.57815500	0.11261800	0.37842200
P	1.54289000	-1.29962500	0.14346400
O	-0.98384100	-3.56333700	-2.16484200
O	-2.77509400	-3.11475200	-0.80055900
C	0.87337800	-2.64767600	-0.95818000
H	1.12008000	-3.63585700	-0.55992900
H	1.41523300	-2.55527900	-1.90310500
C	-0.61439600	-2.53835500	-1.25119400
H	-0.81790900	-1.55020200	-1.68924200
C	-1.58347500	-2.79034600	-0.09324600
H	-1.23371200	-3.67003800	0.47341900
C	-1.85588800	-1.66195400	0.90540400
H	-2.88886800	-1.75789000	1.25201500
H	-1.22090600	-1.79556100	1.78559500
C	-2.38939400	-3.83652000	-1.98404200
C	-2.55777400	-5.33847500	-1.78001900
H	-2.16728700	-5.88217100	-2.64446400
H	-3.61479900	-5.58798700	-1.65595000
H	-2.01180200	-5.66641200	-0.89163300
C	-3.19322600	-3.29411500	-3.15808200
H	-2.92238700	-3.82478300	-4.07456200
H	-2.99548300	-2.22821000	-3.29153800
H	-4.26308800	-3.43053500	-2.97797200
C	-2.33483900	0.93735400	1.85586400
C	-3.55377400	1.63046000	1.82522300
H	-4.10699200	1.72698900	0.89804400
C	-4.06691000	2.21405600	2.98665600
H	-5.01231700	2.74680000	2.94452600
C	-3.37281500	2.11437100	4.19254700
H	-3.77562500	2.56617100	5.09388800
C	-2.15619200	1.42926700	4.23347800
H	-1.60968400	1.34424300	5.16821100
C	-1.63883600	0.85029500	3.07471600
H	-0.68948700	0.32572200	3.12294300
C	-2.76800800	0.36743000	-1.00584200
C	-2.32585900	0.98796200	-2.18392900
H	-1.28456100	1.28547300	-2.26236500
C	-3.19866800	1.19109200	-3.25515100
H	-2.84026100	1.66787100	-4.16271900
C	-4.52696700	0.77464300	-3.16000900
H	-5.20888200	0.93297500	-3.99000100
C	-4.97500200	0.14121100	-1.99789700
H	-6.00374200	-0.19825000	-1.92490400
C	-4.10218600	-0.06919700	-0.93111200
H	-4.46642100	-0.57797300	-0.04515600
C	1.55940300	-1.95961300	1.86417400
C	1.70895900	-1.02981300	2.90837400
H	1.76780400	0.02894600	2.66966300
C	1.79056000	-1.45480800	4.23465600
H	1.91578900	-0.72508000	5.02908000
C	1.70902800	-2.81575600	4.53840800
H	1.76441100	-3.14842500	5.57036300
C	1.56074400	-3.74758300	3.51033800
H	1.50284900	-4.80727000	3.73954000
C	1.49516700	-3.32550000	2.18049900
H	1.40152400	-4.07371800	1.40108100
C	3.32115300	-1.29918100	-0.35089900
C	4.35361000	-1.53682100	0.56553100
H	4.12723800	-1.73097100	1.60786700
C	5.68557300	-1.53992900	0.14299400
H	6.47475900	-1.73275100	0.86339300
C	6.00032600	-1.30913000	-1.19531900

H	7.03544900	-1.32216300	-1.52280500
C	4.97663000	-1.06449200	-2.11519500
H	5.21308900	-0.88825200	-3.16060100
C	3.64804400	-1.05014800	-1.69476300
H	2.86535400	-0.84076200	-2.41919100
C	0.19409900	3.29250300	0.40367800
C	0.60432700	2.99806400	-0.88444600
H	-0.08824500	3.17161700	-1.70348900
C	2.02223300	2.67884700	-1.21418700
C	-1.20197300	3.85216400	0.59418800
H	-1.88295600	3.41041200	-0.13997500
H	-1.57456700	3.57824900	1.58378200
C	-1.23485800	5.38805900	0.45084200
H	-0.91614100	5.70319700	-0.54814600
H	-2.25337600	5.75403900	0.60782400
H	-0.59094400	5.88245700	1.18405100
C	1.12211500	3.44655000	1.58547300
H	2.12526500	3.06466800	1.39949700
H	1.19403800	4.50997500	1.85017700
H	0.70588300	2.93475100	2.45879500
O	2.51025100	1.68750300	-0.59591100
N	2.71674900	3.36260100	-2.13329600
C	2.22924000	4.58572900	-2.76630100
H	2.97659300	5.37732200	-2.65098700
H	2.05763200	4.42419400	-3.83614400
H	1.30291200	4.91829800	-2.30025700
C	4.06838400	2.93736600	-2.50438700
H	4.14673900	2.89675300	-3.59521700
H	4.80635800	3.65246800	-2.12532100
H	4.26662100	1.95440200	-2.08191300

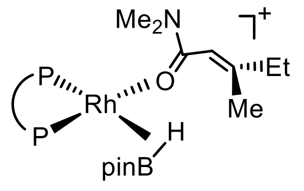

G_{sol} = -2998.10449558 Hatree

Rh	0.12556600	0.23943200	0.84814600
C	-4.85535200	1.07981000	0.24029200
C	-3.69582600	1.31930900	0.88438000
H	0.29375400	1.77774100	1.54552500
C	-5.32060500	2.02630300	-0.84693900
H	-5.41587500	1.46138600	-1.78498700
H	-4.55534300	2.79190900	-1.01404000
B	0.16233300	2.28774800	0.28976500
H	-3.06336900	2.14649900	0.57207300
C	-3.04098600	0.45362600	1.89569400
O	-1.86646300	0.04117000	1.71163000
N	-3.68280800	0.08996700	3.03093300
C	-3.01751600	-0.82139400	3.96398900
H	-3.78134700	-1.36987400	4.52022400
H	-2.39160700	-0.26898400	4.67530100
H	-2.39362900	-1.52242000	3.41297200
C	0.86112200	4.18231500	-0.80450400
C	-0.70669600	4.04824200	-0.91230600
O	1.24862100	2.84972500	-0.34496900
O	-0.99816000	3.00951900	0.08094400
C	-1.48833900	5.30313000	-0.53120300
H	-2.56215900	5.11200300	-0.61448200
H	-1.24347600	6.13004100	-1.20554900
H	-1.27861000	5.61570600	0.49279100
C	1.32174000	5.16640700	0.27786000
H	1.13708200	6.20353400	-0.01614400
H	2.39555800	5.03901000	0.43677500
H	0.81673100	4.97952300	1.22955900
C	-1.17858900	3.52249800	-2.27246500
H	-1.04391000	4.27146600	-3.05769300
H	-2.24465900	3.28435300	-2.21348500
H	-0.64074600	2.61626700	-2.56257200
C	1.58000600	4.47015900	-2.12005600
H	2.65799500	4.52408700	-1.94453000
H	1.26122500	5.43249000	-2.53294000
H	1.39530100	3.69385000	-2.86477400
P	-0.32280700	-1.80376300	-0.33975600
P	2.39666800	0.14961200	0.56575600
O	2.60047000	-0.45024300	-3.46508300
O	1.58272000	-2.50105600	-3.33636800
C	2.99519900	0.14927600	-1.18726400
H	4.02391700	-0.21278200	-1.26629300
H	2.98564000	1.20002300	-1.48799100
C	2.09093500	-0.61711700	-2.14649700
H	1.07420900	-0.20603200	-2.08003000
C	2.03052500	-2.14859200	-2.03223200
H	3.05240600	-2.53032500	-1.87000300
C	1.11131300	-2.80115300	-0.98835300
H	0.73644400	-3.73864100	-1.40846600
H	1.70272300	-3.06229200	-0.10881200
C	2.15210300	-1.56228700	-4.26462700
C	3.36672900	-2.16335800	-4.96399900
H	3.84858500	-1.41244000	-5.59598700

H	3.06462600	-3.00737400	-5.58952900
H	4.09488400	-2.51268700	-4.22747300
C	1.05745000	-1.12192500	-5.22815300
H	1.45792300	-0.40411100	-5.94888800
H	0.23600700	-0.65663500	-4.67845900
H	0.66596200	-1.98442500	-5.77439500
C	-1.22222600	-3.04772200	0.68536900
C	-2.61561300	-3.18763100	0.58767900
H	-3.17084000	-2.59643100	-0.13228700
C	-3.29533700	-4.09766400	1.39926800
H	-4.37193800	-4.20353000	1.30166100
C	-2.59590700	-4.87721900	2.32181700
H	-3.12444800	-5.59040500	2.94710900
C	-1.21098200	-4.73930800	2.43187300
H	-0.65802500	-5.34529000	3.14358300
C	-0.52797800	-3.82915500	1.62393300
H	0.54745100	-3.73586200	1.73344400
C	-1.41480200	-1.54087900	-1.79554700
C	-1.93250400	-0.26615400	-2.06341700
H	-1.69266700	0.55977700	-1.40208700
C	-2.75068700	-0.05798700	-3.17724700
H	-3.13831600	0.93551700	-3.38163000
C	-3.06399100	-1.12064900	-4.02526600
H	-3.69869800	-0.95841000	-4.89141700
C	-2.55609400	-2.39568100	-3.76072300
H	-2.79512100	-3.22540500	-4.41896300
C	-1.73341300	-2.60565400	-2.65580600
H	-1.34399300	-3.60094400	-2.46800300
C	3.10958400	-1.35099000	1.37884500
C	2.46583200	-1.85762600	2.52005300
H	1.53448000	-1.40739600	2.85447500
C	3.01147700	-2.93014600	3.22804800
H	2.50521100	-3.30519700	4.11268000
C	4.20419900	-3.51670100	2.80051400
H	4.62825400	-4.35262700	3.34829400
C	4.85394300	-3.01893400	1.66972400
H	5.78559000	-3.46554400	1.33580000
C	4.31613400	-1.93907700	0.96728500
H	4.85385400	-1.55849100	0.10552600
C	3.36850200	1.48111400	1.39238100
C	3.01014800	1.86278700	2.69409900
H	2.14536200	1.41322100	3.17341600
C	3.75086200	2.82526700	3.37862200
H	3.46111900	3.11202000	4.38512000
C	4.85903500	3.41969700	2.77107300
H	5.43503700	4.17082900	3.30294200
C	5.22561400	3.04105500	1.47929400
H	6.08812100	3.49581300	1.00125500
C	4.48768200	2.07447200	0.79275300
H	4.79379300	1.79853900	-0.21024400
C	-5.73908100	-0.11711400	0.49038000
H	-5.27124600	-0.86008100	1.13865200
H	-6.69123200	0.17986600	0.94603500
H	-5.99019500	-0.59991700	-0.46131300
C	-6.66598300	2.71145800	-0.54335100
H	-6.61255400	3.29090800	0.38402200
H	-6.93244200	3.39786900	-1.35221400
H	-7.48089300	1.98824000	-0.44545100
C	-4.90038200	0.72509800	3.53070600
H	-5.20728400	1.52741600	2.86403600
H	-5.71263900	-0.00439200	3.61711200
H	-4.70739000	1.14598400	4.52371800

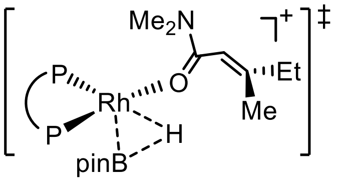

G_{sol} = -2998.09695195 Hatree

Rh	0.11115500	0.17037300	-0.77514200
B	0.25348800	1.95221000	0.15796600
H	0.38224700	1.54203500	-1.55048500
C	4.01624300	0.90636600	-0.76281000
C	5.80207600	1.81057900	0.69452700
H	6.05314500	1.32855100	1.65005100
H	5.02231300	2.54881100	0.90769300
C	5.25515000	0.75994300	-0.24993300
H	3.39066000	1.72885000	-0.42548200
C	3.27483200	-0.07161100	-1.59609800
O	2.16163100	-0.50807000	-1.18922500
N	3.77378200	-0.52055700	-2.76841800
C	3.05355000	-1.55074000	-3.51859700
H	3.78143000	-2.20600100	-4.00471800
H	2.42162200	-1.09734900	-4.29215400
H	2.43169500	-2.13329700	-2.84235900
C	-0.30277400	3.92659800	1.19785200
C	1.17361900	3.51469700	1.56538000
O	-0.82017400	2.68914800	0.60702800
O	1.45982100	2.47565700	0.56878200
C	2.20891800	4.62530700	1.41064100

H	3.20093900	4.24620100	1.67310900
H	1.98460000	5.45912300	2.08367100
H	2.24814900	5.00491800	0.38823000
C	-0.38831200	5.00400800	0.11089400
H	-0.08256200	5.98242400	0.49233600
H	-1.42245700	5.07948400	-0.23482300
H	0.23773900	4.75540400	-0.75031700
C	1.29896600	2.85798200	2.94438800
H	1.16427200	3.58745200	3.74808500
H	2.29513900	2.41942400	3.04305600
H	0.56731000	2.05673200	3.07578400
C	-1.18626500	4.29777900	2.38557200
H	-2.18927200	4.55467600	2.03338000
H	-0.78317300	5.17037500	2.90936200
H	-1.27716400	3.47446900	3.09595400
P	-0.08496800	-1.87734700	0.64324100
P	-2.12211400	0.53025300	-1.04493300
O	-3.42370300	0.29318700	2.82354800
O	-2.82148400	-1.89282500	3.14460200
C	-3.15502000	0.64669500	0.49088000
H	-4.19693600	0.37994300	0.29293500
H	-3.12733200	1.70762200	0.74761700
C	-2.63796100	-0.11760800	1.70695300
H	-1.58279900	0.14054800	1.87273600
C	-2.81671700	-1.64303600	1.74124700
H	-3.80710700	-1.89067000	1.32508800
C	-1.77800000	-2.54676200	1.06147100
H	-1.67437000	-3.46063800	1.65429300
H	-2.16458900	-2.85880200	0.08654900
C	-3.46281200	-0.77728500	3.78509200
C	-4.92374700	-1.09568300	4.08986700
H	-5.42645700	-0.21042300	4.48855100
H	-4.99117200	-1.89998300	4.82704300
H	-5.44567200	-1.40671900	3.18118600
C	-2.65970400	-0.40850000	5.02551500
H	-3.11393000	0.45244000	5.52264000
H	-1.63250100	-0.16024000	4.74914700
H	-2.64156800	-1.24635000	5.72829300
C	0.64881600	-3.38319100	-0.13455100
C	1.50799500	-4.24750500	0.55850600
H	1.78649100	-4.03159100	1.58442300
C	2.00921500	-5.39254700	-0.06496500
H	2.67280600	-6.05451500	0.48352100
C	1.65659200	-5.68925000	-1.38195000
H	2.04449100	-6.58252600	-1.86230700
C	0.80001700	-4.83398000	-2.07928400
H	0.51724800	-5.06112700	-3.10323000
C	0.30431200	-3.68483000	-1.46257300
H	-0.35898800	-3.02644500	-2.01681300
C	0.84517300	-1.67532000	2.21555900
C	2.09916800	-1.04100100	2.15873400
H	2.46483800	-0.65536600	1.21349900
C	2.87424000	-0.91064200	3.31058200
H	3.84372500	-0.42415300	3.25280000
C	2.40684300	-1.39859500	4.53302300
H	3.01073300	-1.29398900	5.42955300
C	1.15856900	-2.01820800	4.59846000
H	0.78604800	-2.39534400	5.54624200
C	0.37870900	-2.16011500	3.44766600
H	-0.59810500	-2.62169800	3.52566300
C	-2.77968500	-0.86286000	-2.06489400
C	-2.06128200	-1.20431900	-3.22513700
H	-1.14775800	-0.66844300	-3.47028200
C	-2.51520800	-2.21452600	-4.07247700
H	-1.95371900	-2.45902500	-4.96934500
C	-3.69160400	-2.90523900	-3.77145300
H	-4.04641800	-3.69177400	-4.43022100
C	-4.41331500	-2.57393100	-2.62476400
H	-5.33250600	-3.10108900	-2.38780100
C	-3.96439100	-1.55666100	-1.77847000
H	-4.55478400	-1.31022400	-0.90282500
C	-2.64461300	2.00339900	-2.02749400
C	-1.81060600	2.51948500	-3.02941100
H	-0.82293100	2.09655200	-3.18015800
C	-2.23250100	3.58559100	-3.82427400
H	-1.57273300	3.97587600	-4.59347500
C	-3.49221700	4.15301100	-3.62721600
H	-3.81820000	4.98628500	-4.24220700
C	-4.33185000	3.64327400	-2.63615300
H	-5.31438600	4.07690100	-2.47621400
C	-3.91527100	2.57211400	-1.84466000
H	-4.59159400	2.19180500	-1.08699800
C	6.15635200	-0.42110800	-0.51250100
H	5.64605800	-1.23961900	-1.02288200
H	7.02439400	-0.13148500	-1.11674700
H	6.55417500	-0.80519200	0.43380200
C	7.05565600	2.53052900	0.16288400
H	6.84750600	3.03262900	-0.78754300
H	7.38789200	3.28936200	0.87712400
H	7.88938500	1.83964900	0.00670200

C	4.91139400	0.07645500	-3.46584500
H	5.25352900	0.96372800	-2.93843000
H	5.73693900	-0.63810100	-3.54921400
H	4.59996500	0.36301300	-4.47618500

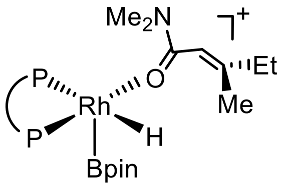

G_{sol} = -2998.10091930 Hatree

Rh	-0.67951500	0.00365200	0.30130100
C	-5.04262000	-0.67712100	-0.33125400
C	-3.85979600	-1.10180800	-0.81701700
H	-2.01049800	0.82383400	0.16916900
C	-5.76917400	0.45955000	-1.02145900
H	-6.79520000	0.13488700	-1.24424900
H	-5.28874500	0.66164600	-1.98464100
B	-0.63463300	0.62064900	-1.59900800
H	-3.40532500	-0.58665200	-1.65896900
C	-2.96743100	-2.13457100	-0.23795300
O	-1.76588000	-1.86110300	0.05190800
N	-3.40076900	-3.39865200	-0.04184100
C	-2.52208000	-4.37685100	0.60483400
H	-1.97173400	-4.96067900	-0.14222000
H	-3.13602100	-5.05945700	1.19819700
H	-1.80890800	-3.86732400	1.24880700
C	-0.40902000	1.92073800	-3.48286400
C	-0.71991100	0.42652900	-3.88391600
O	-0.07865300	1.79150400	-2.05801300
O	-1.15911100	-0.15383900	-2.60672900
C	-1.84476300	0.24793900	-4.90003300
H	-2.01049400	-0.81790000	-5.07975900
H	-1.57916000	0.71077700	-5.85588500
H	-2.78354500	0.68423700	-4.55417900
C	-1.62775900	2.84544000	-3.57142100
H	-1.89489800	3.05488500	-4.61101700
H	-1.39323800	3.79325500	-3.08029800
H	-2.49877500	2.41013700	-3.07315300
C	0.52002400	-0.35517500	-4.32865400
H	0.87977900	-0.00887900	-5.30178200
H	0.26744200	-1.41443800	-4.41281100
H	1.33449100	-0.26488400	-3.60524200
C	0.78539100	2.55184100	-4.19266400
H	0.92392200	3.57733700	-3.83897100
H	0.61835200	2.59065700	-5.27385600
H	1.70811400	2.00214800	-4.00042000
P	1.37485400	-1.48694300	0.54463800
P	0.27525700	1.94517700	1.03417000
O	3.83528800	2.01043300	-1.00394600
O	4.68341800	0.06683400	-0.14258200
C	1.90710600	2.46060600	0.30676200
H	2.46253200	3.08282200	1.01572000
H	1.62219900	3.10445900	-0.52869300
C	2.79758600	1.36223500	-0.27035100
H	2.19942300	0.73466000	-0.94438700
C	3.59690100	0.46909700	0.68955700
H	3.98566400	1.09033800	1.51289500
C	2.93280300	-0.77292200	1.30017800
H	3.68859400	-1.55997200	1.38524700
H	2.62143700	-0.54214800	2.32329700
C	5.00008400	1.16667000	-1.01179400
C	6.17792800	1.96749400	-0.46397200
H	6.34580100	2.85635600	-1.07800900
H	7.08656300	1.35979800	-0.46893400
H	5.97655600	2.29154000	0.56041500
C	5.24956000	0.61667100	-2.41022400
H	5.47396500	1.43479200	-3.09940200
H	4.36636700	0.08203000	-2.76795400
H	6.09791600	-0.07356100	-2.40126400
C	1.03708600	-2.80511000	1.79606600
C	1.51440800	-4.11702800	1.66001500
H	2.08698900	-4.39918700	0.78257000
C	1.25532200	-5.06827400	2.64899500
H	1.63194500	-6.08021300	2.53216500
C	0.52056400	-4.72165300	3.78430900
H	0.32225500	-5.46320100	4.55241900
C	0.04402800	-3.41694100	3.93006300
H	-0.52486600	-3.13891200	4.81255100
C	0.29609600	-2.46604100	2.94020800
H	-0.08008300	-1.45413700	3.06765900
C	1.87076300	-2.42427800	-0.95656500
C	0.85679600	-3.09012500	-1.66903000
H	-0.17413200	-2.99539900	-1.34549700
C	1.17160300	-3.86643100	-2.78312000
H	0.38158800	-4.38474300	-3.31923000
C	2.49679400	-3.97395500	-3.21374600
H	2.74072000	-4.57730400	-4.08311600
C	3.50426800	-3.29976600	-2.52416900
H	4.53547500	-3.37334600	-2.85677600
C	3.19871400	-2.53021500	-1.39749000

H	3.99398500	-2.00158500	-0.88510300
C	0.50192100	1.69933100	2.84692000
C	-0.63865800	1.31937600	3.57990900
H	-1.59298500	1.20631300	3.07128900
C	-0.56045600	1.10772600	4.95591000
H	-1.45094100	0.82433800	5.50909700
C	0.65745200	1.26945200	5.62167800
H	0.71883100	1.10673400	6.69327900
C	1.79253000	1.65073500	4.90618500
H	2.73965100	1.78770700	5.41926400
C	1.71662500	1.86653900	3.52730300
H	2.61042500	2.17516300	2.99597400
C	-0.66565200	3.53047200	0.97386000
C	-1.79052600	3.69831900	0.15800200
H	-2.14505500	2.86877800	-0.44065000
C	-2.45251900	4.92705100	0.11279500
H	-3.32526800	5.04408400	-0.52279700
C	-1.99810100	5.99870400	0.88169200
H	-2.51539900	6.95275500	0.84728700
C	-0.87832200	5.83937200	1.70093300
H	-0.52274700	6.66692300	2.30722700
C	-0.21645900	4.61336200	1.74933700
H	0.64165900	4.50099700	2.40551700
C	-5.70928700	-1.23844600	0.89872100
H	-5.09731100	-1.97933500	1.41562100
H	-5.93749000	-0.43710700	1.60937800
H	-6.66970700	-1.70156000	0.63856100
C	-5.83144700	1.76122900	-0.20047400
H	-4.82679200	2.11983500	0.04574300
H	-6.34192100	2.54238200	-0.77144500
H	-6.38133800	1.63013700	0.73596000
C	-4.63073700	-3.94826400	-0.61033000
H	-5.11225600	-3.21299600	-1.25060900
H	-4.38499900	-4.83253800	-1.20823200
H	-5.32407300	-4.24945100	0.18199800

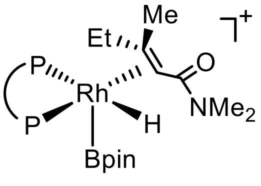

G_{sol} = -2998.09409854 Hatree

Rh	-0.25510700	-0.65750200	-0.84697600
C	0.59115300	-1.62912600	-2.92424700
C	1.63855300	-1.08383200	-2.19084800
H	-1.33893500	-0.96603400	-1.91725100
C	0.26669100	-3.10499200	-2.73423000
H	-0.81079900	-3.25895400	-2.85524700
H	0.53344400	-3.41936800	-1.71864300
B	-0.65870500	1.22694300	-1.51380700
H	2.18982300	-1.75452100	-1.53936500
C	2.31615000	0.20339700	-2.56897700
O	1.67604800	1.26415800	-2.64032600
N	3.65701400	0.16213400	-2.80630700
C	4.37072000	1.40441300	-3.09266000
H	4.64025000	1.46038900	-4.15411500
H	5.28831600	1.44392900	-2.49680100
H	3.73143200	2.24754700	-2.84258600
C	-1.41164500	2.98787300	-2.79076700
C	-1.02506200	3.49477900	-1.33302700
O	-1.36084900	1.52650700	-2.64950800
O	-0.39205400	2.31452000	-0.72720800
C	-0.00943700	4.63514000	-1.29774500
H	0.23006300	4.87770400	-0.25862800
H	-0.41901000	5.53503600	-1.76713000
H	0.91514500	4.36549300	-1.81031500
C	-0.40532800	3.37527800	-3.87887200
H	-0.41249700	4.45527700	-4.05293600
H	-0.69224500	2.88452800	-4.81346700
H	0.60491100	3.05502200	-3.62544700
C	-2.22855400	3.84275800	-0.45093000
H	-2.71305100	4.76497600	-0.78388300
H	-1.89077200	3.98501700	0.57941100
H	-2.97778900	3.04660600	-0.45870900
C	-2.82354600	3.35911100	-3.24484300
H	-3.00724200	2.93603900	-4.23635000
H	-2.93335900	4.44546600	-3.32062500
H	-3.59028300	2.97885100	-2.56804200
P	1.46278700	-0.45224300	1.13744600
P	-2.16176200	-0.59877600	0.50826500
O	-1.30059900	2.41857600	3.16665400
O	0.57184300	1.41655800	4.03664900
C	-2.28993000	0.72802300	1.80872700
H	-2.96416800	0.40837100	2.60970700
H	-2.79420000	1.55270200	1.29827600
C	-0.98357200	1.27575500	2.37197900
H	-0.33406300	1.57590800	1.54137200
C	-0.19770900	0.42242300	3.37251600
H	-0.90475900	-0.03590300	4.08548600
C	0.72197600	-0.68033200	2.84269800

H	1.52800400	-0.82922200	3.56660900
H	0.16467100	-1.61905900	2.79738100
C	-0.22144000	2.61603300	4.10196200
C	-0.82597800	2.78634300	5.49117800
H	-1.50382100	3.64414200	5.50556100
H	-0.03722600	2.94967900	6.23010700
H	-1.39229500	1.89422500	5.77087900
C	0.65412600	3.78742800	3.67548400
H	0.08482000	4.71959500	3.72308500
H	1.01331600	3.63749400	2.65466700
H	1.51966700	3.87131000	4.33827100
C	2.49804900	-1.98770800	1.05899200
C	3.88640800	-1.96201600	0.85771100
H	4.40924400	-1.01363600	0.79956000
C	4.61073400	-3.15281500	0.74972400
H	5.68663100	-3.11362200	0.60585300
C	3.96190700	-4.38448500	0.83809600
H	4.52796900	-5.30770400	0.76113700
C	2.57873500	-4.42222400	1.03060700
H	2.06423900	-5.37578700	1.10586300
C	1.85191300	-3.23577300	1.13300800
H	0.77586800	-3.29108900	1.27570800
C	2.66513100	0.92218300	1.36201400
C	2.68637200	1.99128900	0.45520400
H	1.99190300	2.01324600	-0.37537600
C	3.58993400	3.04377800	0.63306100
H	3.59380900	3.87055300	-0.07132300
C	4.47646600	3.03790400	1.70920900
H	5.17681100	3.85686700	1.84448400
C	4.45788600	1.97668800	2.61907500
H	5.14136600	1.96851800	3.46278100
C	3.55595800	0.92871900	2.45087700
H	3.55503800	0.11543700	3.16956300
C	-2.22193900	-2.23412100	1.35984400
C	-2.08073400	-3.38235700	0.55892700
H	-1.93696000	-3.27767600	-0.51266100
C	-2.14836100	-4.65628300	1.12286200
H	-2.04718400	-5.53203200	0.48878300
C	-2.35589300	-4.80473300	2.49636100
H	-2.41036100	-5.79575100	2.93613200
C	-2.50283200	-3.67359200	3.29963000
H	-2.67304300	-3.78071000	4.36660500
C	-2.43887400	-2.39624400	2.73679800
H	-2.56565200	-1.53512100	3.38357100
C	-3.83495500	-0.49908300	-0.26321000
C	-4.02387700	-0.12527700	-1.60043000
H	-3.17388800	0.11232300	-2.22887000
C	-5.31617000	-0.03695500	-2.12635800
H	-5.45132200	0.25241100	-3.16421700
C	-6.42423300	-0.31880900	-1.32849200
H	-7.42583500	-0.25191700	-1.74231700
C	-6.24235200	-0.69222700	0.00554400
H	-7.09986800	-0.91909200	0.63165300
C	-4.95726600	-0.78379300	0.53559400
H	-4.83295700	-1.09470600	1.56858000
C	0.06149800	-0.99331500	-4.18951900
H	0.08340900	0.09250800	-4.14257400
H	0.68571500	-1.32055700	-5.03133600
H	-0.96130100	-1.31876500	-4.39324500
C	1.01417500	-4.00100300	-3.74317200
H	2.09912400	-3.89050300	-3.64822300
H	0.76802400	-5.05120200	-3.56220900
H	0.73629100	-3.76854800	-4.77472100
C	4.45557900	-1.05118700	-2.94068900
H	3.83310500	-1.94207000	-2.89621100
H	4.97055700	-1.04564000	-3.90828700
H	5.21312000	-1.10954400	-2.15061600

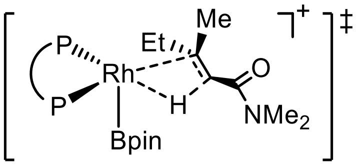

G_{sol} = -2998.08101372 Hatree

Rh	0.68665100	0.45260900	-0.16942700
C	1.35234600	2.35089700	-1.31158300
C	2.45171000	1.43165100	-1.31360200
H	2.19481500	0.08341200	-0.57449200
C	0.60921300	2.60413500	-2.61758800
H	-0.42252800	2.88992100	-2.38753200
H	0.57012600	1.68312800	-3.20293800
B	0.46879100	-0.91849000	-1.64391000
H	2.63161500	0.92185900	-2.25568000
C	3.65501600	1.70846000	-0.42059800
O	3.54039100	1.62139300	0.80923500
N	4.82608300	2.05450500	-1.01836500
C	5.99810600	2.31602900	-0.18260200
H	6.27670800	3.37465100	-0.23659500
H	6.84301400	1.71546000	-0.53623200
H	5.76439200	2.05629500	0.84723600

C	0.40178900	-2.95526800	-2.71461900	H	-2.80424300	3.73301000	-0.75644700
C	0.55731700	-1.79969800	-3.77847800	C	-2.28185800	5.30733400	0.60810100
O	0.03582800	-2.21139800	-1.50028900	H	-2.76365500	6.07462200	0.00937000
O	0.86525500	-0.63550200	-2.93120900	C	-1.65436000	5.64537200	1.80745600
C	1.70770900	-1.97766700	-4.76616300	H	-1.64418000	6.67666600	2.14666300
H	1.75196200	-1.11629000	-5.43841600	C	-1.03990600	4.64960400	2.56994400
H	1.55501300	-2.87177700	-5.37863800	H	-0.54868900	4.90386900	3.50448800
H	2.67068800	-2.06579100	-4.26048800	C	-1.05044100	3.32456900	2.13503200
C	1.71372600	-3.68300200	-2.40132600	H	-0.54812100	2.57275200	2.73697900
H	2.02975800	-4.31026100	-3.23950200	C	-2.95242300	1.12981100	-0.92365600
H	1.56970300	-4.32611400	-1.52987800	C	-2.59512000	0.87244500	-2.25404800
H	2.52050100	-2.98325100	-2.16757400	H	-1.55615300	0.69253200	-2.50423300
C	-0.73591400	-1.48095300	-4.53345000	C	-3.56529200	0.85221900	-3.25970000
H	-0.98474900	-2.27682900	-5.24092800	H	-3.27338400	0.65500200	-4.28675900
H	-0.60095300	-0.55548500	-5.10055700	C	-4.90284900	1.08934800	-2.94397100
H	-1.58051500	-1.34941700	-3.85376200	H	-5.65785900	1.07597100	-3.72423500
C	-0.70499200	-3.96568500	-3.00413000	C	-5.26941900	1.34508800	-1.61978200
H	-0.75040500	-4.70121600	-2.19657400	H	-6.30956400	1.52786900	-1.36782600
H	-0.50334800	-4.50393000	-3.93575700	C	-4.30373000	1.36393700	-0.61520600
H	-1.68398700	-3.49018300	-3.07984800	H	-4.60950400	1.55914000	0.40617500
P	-1.65154100	1.21005300	0.37343800	C	0.75140700	-0.61240900	3.08307800
P	0.70022400	-1.36929100	1.39784500	C	1.54037900	0.53997300	3.25523400
O	-2.97381800	-3.07890900	0.80515400	H	2.10281000	0.95299000	2.42168800
O	-4.14289300	-1.18617700	1.36375700	C	1.64124600	1.14461100	4.51041200
C	-0.71917200	-2.56733400	1.38660600	H	2.26214600	2.02768200	4.62957300
H	-0.83018600	-3.06156200	2.35562600	C	0.95687700	0.61453500	5.60554200
H	-0.43875500	-3.32668400	0.65602400	H	1.03547700	1.08651400	6.58018800
C	-2.05331000	-1.99690500	0.92309400	C	0.17877200	-0.53309700	5.44464600
H	-1.91535400	-1.51676800	-0.05369700	H	-0.34783300	-0.95882700	6.29351100
C	-2.81326500	-1.05167600	1.85611400	C	0.07888500	-1.14613400	4.19426700
H	-2.75561700	-1.44152000	2.88623800	H	-0.52106900	-2.04502100	4.10406900
C	-2.42061000	0.42578200	1.89153600	C	2.18596000	-2.46789500	1.40015000
H	-3.30389000	1.00669900	2.17160500	C	3.45475400	-1.88431300	1.25364500
H	-1.68798500	0.57553800	2.68808000	H	3.55030100	-0.81081300	1.11931900
C	-4.30559600	-2.54154000	0.90743400	C	4.60416000	-2.67268100	1.30403100
C	-5.07336100	-3.36603000	1.93478600	H	5.57912500	-2.20650500	1.19590300
H	-5.11042900	-4.41444700	1.62662300	C	4.50545400	-4.05249200	1.49610500
H	-6.09601800	-2.99251000	2.03270100	H	5.40132100	-4.66490900	1.53123800
H	-4.58243400	-3.30961400	2.90969800	C	3.24983000	-4.63853100	1.65145700
C	-4.97526600	-2.50730700	-0.46087300	H	3.16209900	-5.70907400	1.81096700
H	-5.11682200	-3.52469300	-0.83505000	C	2.09585800	-3.85232200	1.61029100
H	-4.35816600	-1.94859500	-1.16847200	H	1.13516500	-4.33496400	1.75003900
H	-5.95188900	-2.02037400	-0.39287500	C	1.38031700	3.53235600	-0.35442500
C	-1.69066600	2.97235600	0.93397200	H	1.65994300	3.25215100	0.66151200
C	-2.30196100	3.98006900	0.17235800	H	2.12187600	4.26705300	-0.69765900

H	0.41414300	4.03606500	-0.32879200
C	1.24011300	3.72353500	-3.47108500
H	2.27352400	3.48730200	-3.74739700
H	0.67270300	3.85208100	-4.39793500
H	1.24340700	4.68544200	-2.95068200
C	5.03427800	2.26184100	-2.44718400
H	4.11350700	2.13204600	-3.01035200
H	5.40022500	3.27948400	-2.62583200
H	5.78375700	1.55893100	-2.82933600

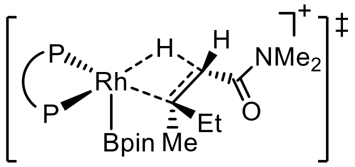

G_{sol} = -2998.08053795 Hatree

Rh	0.70776700	0.45611800	0.54503500
C	2.85153900	1.16437300	0.63869100
C	2.02706800	2.25165400	1.08787900
H	0.44608600	1.95622200	1.08305400
C	3.51492600	0.31659200	1.72566400
H	3.75054400	-0.67020100	1.31378000
H	2.81788400	0.15531100	2.56028100
B	0.12637600	1.16400900	-1.27696000
H	1.98452600	2.39229400	2.16570300
C	1.90983900	3.51776000	0.25381500
O	1.44035900	3.46638600	-0.88578300
N	2.35640600	4.67782100	0.81159400
C	2.19157300	5.92891500	0.07139800
H	3.16944400	6.36630800	-0.15813900
H	1.62045500	6.64528800	0.67247800
H	1.66098600	5.72416100	-0.85516000
C	-1.12653000	2.17968100	-2.91766900
C	0.18013000	1.50979200	-3.55603300
O	-0.99211200	1.90069700	-1.48506100
O	0.78904100	0.79713600	-2.41696200
C	-0.10384000	0.47007300	-4.64259700
H	0.83823500	0.02266100	-4.97331700
H	-0.56745300	0.93994300	-5.51522400
H	-0.76159400	-0.32941900	-4.29638800
C	-2.44801500	1.54983400	-3.36834900
H	-2.64047700	1.75612100	-4.42554300
H	-3.26335400	1.98447500	-2.78422700
H	-2.46487400	0.46798000	-3.22169100
C	1.22545300	2.50775900	-4.06216400
H	0.84360400	3.07402000	-4.91677400
H	2.11096400	1.95800200	-4.39461100
H	1.52721100	3.20304200	-3.27917700
C	-1.20546600	3.69985600	-3.07028000
H	-2.09786500	4.06109600	-2.55197800
H	-1.28744500	3.98687000	-4.12313200
H	-0.33594200	4.18827100	-2.63081800
P	-1.57602900	0.05455500	1.27505800
P	1.13565700	-1.80016800	-0.34764500
O	-2.32081200	-2.25587500	-2.53718000
O	-3.65046500	-2.39898800	-0.68523500
C	-0.10710000	-2.32155200	-1.63571400
H	-0.09234300	-3.40951900	-1.74478900
H	0.24406800	-1.90085000	-2.57977500
C	-1.54024000	-1.84031700	-1.41554000
H	-1.55844500	-0.74475600	-1.36006700
C	-2.30413900	-2.42335100	-0.21502000
H	-1.99139300	-3.46676000	-0.05774400
C	-2.22166400	-1.70086200	1.14021200
H	-3.20907000	-1.74980100	1.60924200
H	-1.54329800	-2.25519900	1.79326700
C	-3.61932100	-2.69563100	-2.08547000
C	-3.74459100	-4.19894600	-2.33127700
H	-3.62830000	-4.41423500	-3.39665900
H	-4.72474400	-4.55686600	-2.00513100
H	-2.97253200	-4.74951100	-1.78723800
C	-4.71153400	-1.89190700	-2.77436200
H	-5.69462800	-2.19763100	-2.40589200
H	-4.67674800	-2.06284800	-3.85322500
H	-4.57361900	-0.82633300	-2.57950100
C	-1.58735800	0.21380200	3.12014800
C	-0.39607900	0.39451200	3.83578300
H	0.54664200	0.47668600	3.30168100
C	-0.40285200	0.48781100	5.22933300
H	0.52968100	0.62892900	5.76804200
C	-1.60909600	0.40534800	5.92495700
H	-1.61966700	0.48055800	7.00801300
C	-2.80511100	0.23438500	5.22312700
H	-3.74765500	0.17958800	5.75936500
C	-2.79610000	0.14097400	3.83207700
H	-3.73602200	0.03045900	3.29962300
C	-2.95136300	1.17374900	0.79307700
C	-4.15149000	0.70246000	0.24501000
H	-4.28804500	-0.35272600	0.03621900
C	-5.18271300	1.59887400	-0.05558800

H	-6.10963100	1.22354400	-0.47929500
C	-5.02727600	2.96160200	0.19264400
H	-5.83214000	3.65358000	-0.03663400
C	-3.83060700	3.43508900	0.74021500
H	-3.70230400	4.49556400	0.93544000
C	-2.79791200	2.54881900	1.03166900
H	-1.86722200	2.92936300	1.44231800
C	1.02625000	-3.11211200	0.95005000
C	1.09309400	-2.75863900	2.30595800
H	1.18616800	-1.71551300	2.58957000
C	1.03311100	-3.73170400	3.30656200
H	1.07989400	-3.43558100	4.35011500
C	0.90964500	-5.07762300	2.96213400
H	0.86059600	-5.83664200	3.73681900
C	0.85468600	-5.44664500	1.61548200
H	0.76814500	-6.49352700	1.34079100
C	0.91577400	-4.47414500	0.61781200
H	0.89640000	-4.78690300	-0.42149100
C	2.73051300	-2.20409100	-1.18990100
C	3.71781800	-2.97055000	-0.54909400
H	3.54187900	-3.37244600	0.44267800
C	4.92922300	-3.24357400	-1.18848300
H	5.67887000	-3.84326300	-0.68091500
C	5.17053200	-2.75923300	-2.47405200
H	6.10911300	-2.97958000	-2.97336000
C	4.19883900	-1.98696100	-3.11415100
H	4.38189300	-1.59962500	-4.11205900
C	2.99137600	-1.70158600	-2.47671500
H	2.26672600	-1.06288700	-2.96845400
C	3.63004600	1.26401300	-0.65888800
H	3.03731200	1.69059300	-1.46477700
H	4.51502800	1.89868300	-0.51168400
H	3.98561200	0.28116300	-0.97111600
C	4.81196200	0.93017800	2.28995600
H	4.62988800	1.90430400	2.75651400
H	5.24079600	0.27366600	3.05303800
H	5.56416900	1.06573800	1.50821800
C	2.96386400	4.81072900	2.13291100
H	3.44702200	3.88737200	2.44861100
H	3.73767000	5.58200500	2.08658200
H	2.22937400	5.11275100	2.89090300

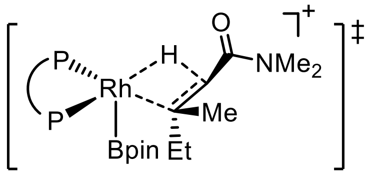

G_{sol} = -2998.07276588 Hatree

Rh	-0.39967800	0.13354400	0.75564300
C	-0.46773500	1.51144000	2.62520700
C	-1.30991800	0.36324500	2.80420600
H	-1.74529400	-0.37670800	1.44339200
C	-1.15279600	2.85703000	2.40536400
H	-2.08639400	2.70291400	1.86157800
H	-0.51104000	3.48149100	1.77320100
B	-1.70794500	1.26123400	-0.28854600
H	-2.37712900	0.53906500	2.91857200
C	-0.69321100	-0.85459800	3.45899300
O	0.22339400	-1.42591900	2.83012900
N	-1.12223500	-1.26550400	4.67043800
C	-0.54109400	-2.46251100	5.27855900
H	-1.32407000	-3.20715800	5.45937100
H	-0.07540000	-2.20685300	6.23614300
H	0.21036900	-2.87387900	4.60838000
C	-3.75842400	2.11555800	-0.91185600
C	-2.60120400	2.85891300	-1.69013900
O	-3.01998500	1.37183800	0.11399500
O	-1.43699700	2.00283900	-1.41195400
C	-2.78758200	2.94049300	-3.20357800
H	-1.91358300	3.41986200	-3.65303900
H	-3.66518600	3.54673200	-3.44935600
H	-2.91279500	1.95688400	-3.65840600
C	-4.50594000	1.07530200	-1.75449700
H	-5.14058700	1.55134200	-2.50755300
H	-5.14499000	0.48094500	-1.09618500
H	-3.81789200	0.39544800	-2.26381100
C	-2.27129300	4.24950000	-1.13757400
H	-3.07339500	4.96064100	-1.35496300
H	-1.35311400	4.61172100	-1.60579000
H	-2.11101400	4.22979000	-0.05750800
C	-4.76019800	3.03126900	-0.21203400
H	-5.49122800	2.42662700	0.33173700
H	-5.30473700	3.63875700	-0.94184000
H	-4.27572800	3.70075100	0.50049700
P	1.66607700	0.97887900	-0.35590000
P	-0.81537000	-1.79807300	-0.66543300
O	2.99531400	-3.31126700	-0.56019400

O	3.82666700	-1.57042200	-1.78645300
C	0.64573200	-2.95428800	-0.53665000
H	0.57909000	-3.69767600	-1.33557500
H	0.56446300	-3.48770200	0.41332500
C	2.01460700	-2.27966900	-0.57438200
H	2.12466000	-1.65686700	0.31998200
C	2.40794400	-1.46057500	-1.82086500
H	2.01214400	-1.96581400	-2.71725500
C	2.04187600	0.03104600	-1.92311700
H	2.87056100	0.52231900	-2.44221800
H	1.15383900	0.13788300	-2.54869700
C	4.14838100	-2.88165100	-1.31524600
C	4.34902500	-3.85084900	-2.47918100
H	4.49434300	-4.86646500	-2.10138100
H	5.22718700	-3.56479400	-3.06424500
H	3.47524600	-3.85264200	-3.13627600
C	5.36513700	-2.78076600	-0.40764400
H	5.61576000	-3.76657400	-0.00726500
H	5.15828900	-2.09947900	0.41972100
H	6.22401600	-2.40326900	-0.96943300
C	1.67986300	2.72988700	-0.94387600
C	1.84122700	3.75858000	0.00003500
H	1.99516700	3.51675300	1.04677100
C	1.84643200	5.09623600	-0.39315700
H	1.98393000	5.87517400	0.35095900
C	1.69281400	5.43145300	-1.74041800
H	1.70769800	6.47208700	-2.04984900
C	1.53186600	4.41913000	-2.68671400
H	1.42244100	4.66841400	-3.73816600
C	1.52052500	3.07889900	-2.29244200
H	1.38894600	2.31609100	-3.05050900
C	3.25295900	0.88156100	0.59518500
C	3.35910200	0.07510700	1.73797900
H	2.50019000	-0.48508400	2.09333400
C	4.56944000	-0.02186700	2.42886400
H	4.63415200	-0.64812100	3.31379700
C	5.68853900	0.68278700	1.98418600
H	6.62885900	0.60931500	2.52224700
C	5.59553800	1.48151300	0.84207400
H	6.46376300	2.02811300	0.48646000
C	4.38810500	1.58138000	0.15261600
H	4.33112300	2.21175800	-0.72889000
C	-1.15481700	-1.82547600	-2.48570700
C	-1.22253900	-0.65535600	-3.25417300
H	-1.11382900	0.31350400	-2.78180400
C	-1.44271000	-0.72544600	-4.63424900
H	-1.48595800	0.19045500	-5.21642100
C	-1.60558700	-1.95981500	-5.26002200
H	-1.77562300	-2.01216200	-6.33109800
C	-1.55321500	-3.13214700	-4.50075900
H	-1.68670200	-4.09829400	-4.97788800
C	-1.32990900	-3.06697700	-3.12715000
H	-1.30848800	-3.98911100	-2.55420900
C	-2.20047100	-2.80974700	0.02509700
C	-3.45019400	-2.86067600	-0.61010200
H	-3.60405000	-2.35261500	-1.55582000
C	-4.50562700	-3.57538200	-0.03968600
H	-5.46522800	-3.60914400	-0.54687900
C	-4.32732900	-4.25004700	1.16806100
H	-5.14589400	-4.81420400	1.60455700
C	-3.08868400	-4.19754500	1.81112500
H	-2.93990500	-4.72431600	2.74939300
C	-2.03433800	-3.47645200	1.25076700
H	-1.08775400	-3.42360800	1.77874400
C	0.85505300	1.61198700	3.36412400
H	0.68160900	2.03197600	4.36372300
H	1.35285500	0.65456600	3.49150500
H	1.54468700	2.28644800	2.85350200
C	-1.46306800	3.62261000	3.70791000
H	-2.01474600	4.53924000	3.47825900
H	-2.08540600	3.02646900	4.38543100
H	-0.55826600	3.91250800	4.24844600
C	-2.16005200	-0.60133300	5.45448500
H	-1.80717500	-0.47014400	6.48224200
H	-2.39076400	0.38311700	5.05194800
H	-3.07627100	-1.20275300	5.47898500

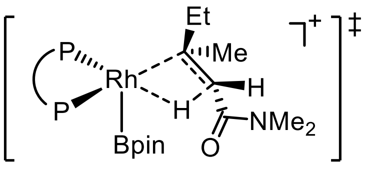

G_{sol} = -2998.07717351 Hatree

Rh	0.66776400	0.67558200	0.14194000
C	1.27278200	2.73509100	-0.80982900
C	2.45776900	2.06139900	-0.39986800
H	2.25106400	0.58433400	0.25280700
C	0.73426900	3.82566100	0.11014800
H	0.87719200	3.53906500	1.16007800
H	-0.34369300	3.92133100	-0.03628000

B	0.84451900	-0.61201000	-1.42882600
H	2.88895600	2.40327900	0.54020500
C	3.45790300	1.52927400	-1.41997500
O	3.21027400	0.50613200	-2.06495000
N	4.61364600	2.23015300	-1.58393900
C	5.61169200	1.74241000	-2.53536600
H	6.58147100	1.64403100	-2.03549700
H	5.71932600	2.44678000	-3.36810200
H	5.29528200	0.77553400	-2.91879500
C	0.51161700	-1.45955900	-3.54603400
C	1.34772100	-2.49455900	-2.66534100
O	0.38583600	-0.28496100	-2.66915900
O	1.29749900	-1.89770100	-1.32317000
C	0.73879000	-3.89437100	-2.56564900
H	1.36722500	-4.51447600	-1.92000800
H	0.70359500	-4.37289600	-3.54918200
H	-0.26853300	-3.88384300	-2.14536800
C	-0.91097600	-1.92322600	-3.86802400
H	-0.90199600	-2.77545300	-4.55349300
H	-1.45881100	-1.10695100	-4.34405600
H	-1.45679100	-2.21214600	-2.96731200
C	2.82702200	-2.60454800	-3.04722600
H	2.94443100	-3.06477200	-4.03265100
H	3.33136700	-3.24304600	-2.31630100
H	3.31587500	-1.63064900	-3.04350200
C	1.21115000	-0.99494200	-4.82321400
H	0.58174700	-0.26104500	-5.33455700
H	1.37149400	-1.83306700	-5.50836600
H	2.17223800	-0.52799600	-4.60481300
P	-1.85940300	0.91036600	0.14532400
P	0.78270700	-1.20808100	1.59932600
O	-2.15162400	-3.61970500	0.05202300
O	-3.83872200	-2.11928200	0.44610200
C	-0.28772200	-2.69115200	1.24443000
H	-0.49966900	-3.25262400	2.15954200
H	0.34119700	-3.32372900	0.61488400
C	-1.56665700	-2.38887900	0.47028900
H	-1.31243500	-1.78547900	-0.41127700
C	-2.71842500	-1.71965200	1.22617700
H	-2.79347800	-2.16825300	2.23124700
C	-2.70563500	-0.20016900	1.40052100
H	-3.73723700	0.14028900	1.52647400
H	-2.19875100	0.03509500	2.33928500
C	-3.58607600	-3.45597000	-0.01041500
C	-4.22551200	-4.48269200	0.92083500
H	-3.94109100	-5.49353200	0.61681500
H	-5.31495800	-4.39905600	0.88824800
H	-3.89171800	-4.32930300	1.95035100
C	-4.07291900	-3.56964100	-1.44768000
H	-3.84536200	-4.56287400	-1.84280800
H	-3.58760200	-2.81492200	-2.07003500
H	-5.15446000	-3.41525800	-1.49412500
C	-2.33909600	2.52550500	0.91897700
C	-3.12220100	3.47586000	0.24768900
H	-3.48351600	3.27553000	-0.75482100
C	-3.45226500	4.68440600	0.86694400
H	-4.06307400	5.40797600	0.33526900
C	-3.00966000	4.95846500	2.16110500
H	-3.27342500	5.89574800	2.64145700
C	-2.22681500	4.01906900	2.83627900
H	-1.87896400	4.22235200	3.84491700
C	-1.88790600	2.81565100	2.21876500
H	-1.26683300	2.10607900	2.75890100
C	-2.90114300	0.84206800	-1.37091200
C	-2.35361300	1.26448800	-2.59193200
H	-1.29950600	1.49497400	-2.65861700
C	-3.15111500	1.35124100	-3.73499700
H	-2.71353000	1.68603200	-4.67101200
C	-4.50032800	1.00133600	-3.67686500
H	-5.12036900	1.06591500	-4.56603800
C	-5.04916700	0.56062700	-2.47067400
H	-6.09609200	0.27718500	-2.41915800
C	-4.26004200	0.48464500	-1.32308100
H	-4.70843100	0.12857400	-0.40454700
C	0.34752100	-0.59103200	3.28401400
C	0.99546500	0.57972000	3.72052600
H	1.71909100	1.06874100	3.07274000
C	0.74379900	1.10736700	4.98681700
H	1.26155400	2.00566500	5.30977700
C	-0.16580900	0.47672900	5.83945300
H	-0.36304500	0.88529500	6.82565000
C	-0.81246800	-0.68618600	5.42086300
H	-1.51409300	-1.18725900	6.08088100
C	-0.55657300	-1.21913000	4.15436000
H	-1.06483900	-2.13129300	3.86121300
C	2.43158200	-1.98547500	1.89214500
C	3.47738200	-1.85415700	0.96790700
H	3.33001400	-1.28606600	0.05688000
C	4.70195900	-2.48370100	1.20074500
H	5.50458500	-2.37767200	0.47687600

C	4.89433900	-3.24845600	2.35179600
H	5.84846800	-3.73523000	2.53015800
C	3.85582100	-3.38623400	3.27497700
H	3.99862900	-3.97795600	4.17410100
C	2.63175500	-2.75800300	3.04878000
H	1.83909800	-2.86282000	3.78329900
C	0.96517100	2.91289500	-2.28485200
H	1.61817900	3.68625500	-2.71291600
H	1.10910100	1.99374800	-2.85183200
H	-0.06298100	3.25610300	-2.42338400
C	1.37571700	5.20918000	-0.11842700
H	1.23236400	5.55571800	-1.14587300
H	0.91563200	5.94652300	0.54603600
H	2.45072500	5.20499400	0.08960900
C	4.96916600	3.47642700	-0.91555700
H	5.21888200	4.23858200	-1.66236500
H	4.14859900	3.85675800	-0.31281400
H	5.84514200	3.33211400	-0.27164100

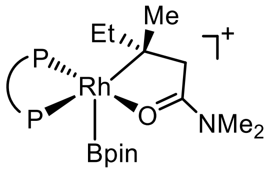

G_{sol} = -2998.10660510 Hatree

Rh	0.52844100	0.58623100	0.56140800
C	0.57654300	2.56935900	1.40299800
C	1.88384000	3.19015100	0.84474900
H	1.72386300	3.55100800	-0.17769200
C	-0.58360700	3.55146500	1.17893200
H	-1.51915000	3.07940300	1.49190000
H	-0.67732600	3.75123600	0.10446400
B	0.37646300	1.15757800	-1.35502600
H	2.18431000	4.05065400	1.45320400
C	2.97472900	2.15858800	0.77149200
O	2.62538100	0.94550400	0.65540400
N	4.27636900	2.47740500	0.81672200
C	5.30458700	1.43342700	0.79878900
H	5.91646700	1.50519400	1.70434900
H	5.95265000	1.56654200	-0.07315600
H	4.83161000	0.45659100	0.74830100
C	-0.16925300	1.18105300	-3.59760000
C	0.91866100	2.28208700	-3.29643100
O	-0.61152400	0.81442400	-2.24729700
O	1.36876600	1.91496500	-1.94810000
C	2.12829500	2.26996800	-4.22762300
H	2.83946500	3.04148100	-3.91926900
H	1.82520800	2.48708600	-5.25679300
H	2.64337800	1.30805100	-4.21607100
C	0.39627100	-0.09018000	-4.24193600
H	0.67406900	0.08307800	-5.28548300
H	-0.36643600	-0.87333600	-4.21738700
H	1.27930900	-0.45551100	-3.71066700
C	0.34596600	3.70110800	-3.20247000
H	0.03060600	4.07133200	-4.18179900
H	1.11748200	4.37535100	-2.82010700
H	-0.51175800	3.74814600	-2.52564200
C	-1.38322600	1.66512700	-4.38674100
H	-2.08715600	0.83958900	-4.52132800
H	-1.08337500	2.01368900	-5.38031900
H	-1.90809300	2.47428800	-3.87726100
P	-1.77869200	0.07123500	0.89865700
P	1.22825200	-1.80757400	-0.06211700
O	-1.82403000	-2.98056800	-2.53834900
O	-3.42845700	-2.52964500	-0.95691800
C	0.23370900	-2.77743200	-1.31345300
H	0.28932100	-3.85557500	-1.13268900
H	0.73610300	-2.59960300	-2.26874600
C	-1.20697700	-2.30412200	-1.44217600
H	-1.21507300	-1.22448000	-1.62374300
C	-2.16916500	-2.63418800	-0.30421100
H	-2.00442900	-3.67855900	0.01418900
C	-2.14013700	-1.77180800	0.95576600
H	-3.09153300	-1.90035500	1.47931300
H	-1.37823400	-2.17278600	1.62804500
C	-3.24697600	-2.92955100	-2.33136300
C	-3.80879800	-4.32772000	-2.55904100
H	-3.58789700	-4.66210500	-3.57638400
H	-4.89253400	-4.32950500	-2.41610900
H	-3.35849900	-5.03441100	-1.85733900
C	-3.88480700	-1.86754800	-3.21962500
H	-3.77764200	-2.14340000	-4.27228500
H	-3.40542600	-0.90039700	-3.04928500
H	-4.94928700	-1.77050500	-2.98960200
C	-2.15689000	0.41470000	2.67993800
C	-3.09986200	1.37652200	3.07164500
H	-3.65272100	1.93495000	2.32432100
C	-3.34770300	1.61474200	4.42563300
H	-4.08291200	2.36099200	4.71147400
C	-2.66363900	0.89407700	5.40479300

H	-2.86306100	1.07662500	6.45629400
C	-1.72352400	-0.06665300	5.02525400
H	-1.18752200	-0.63407700	5.78024800
C	-1.46700400	-0.30140500	3.67483500
H	-0.71820700	-1.04162900	3.40632200
C	-3.20844700	0.81332600	0.00258300
C	-3.06295500	1.98453200	-0.75229300
H	-2.08444500	2.42494900	-0.87263700
C	-4.16791000	2.58322700	-1.36022000
H	-4.03670200	3.49330200	-1.93821100
C	-5.43471900	2.01627900	-1.22477500
H	-6.29478300	2.48089900	-1.69761100
C	-5.59204500	0.84714400	-0.47749600
H	-6.57379000	0.39664400	-0.36776900
C	-4.49036200	0.24931200	0.13190500
H	-4.64139600	-0.66024000	0.69951100
C	1.28496700	-2.80468100	1.49031100
C	1.94788400	-2.21821200	2.58567100
H	2.40768200	-1.23909000	2.47428200
C	2.04215400	-2.88959100	3.80412900
H	2.56592100	-2.42715700	4.63561600
C	1.46535400	-4.15386200	3.95431800
H	1.53491000	-4.67606400	4.90357800
C	0.80350700	-4.74367800	2.87765300
H	0.35742600	-5.72787000	2.98487000
C	0.71603200	-4.07659000	1.65201300
H	0.20405600	-4.56317600	0.82875300
C	2.93110300	-2.02433700	-0.74444700
C	3.45237700	-1.05236500	-1.61301800
H	2.89637200	-0.14075000	-1.80151400
C	4.69804100	-1.24083700	-2.21502600
H	5.09022100	-0.48394600	-2.88853300
C	5.44150700	-2.39186700	-1.94760000
H	6.41123200	-2.53596900	-2.41463800
C	4.93537500	-3.35617700	-1.07429200
H	5.50967800	-4.25238900	-0.85926500
C	3.68727800	-3.17592300	-0.47606600
H	3.30641300	-3.93443200	0.20010600
C	0.76823900	2.25582000	2.89894000
H	1.44514300	1.40870900	3.07237700
H	1.21394300	3.11265900	3.42547700
H	-0.17691400	2.02911900	3.39199400
C	-0.47969800	4.90610400	1.90992700
H	0.40217400	5.48064400	1.60768700
H	-1.35644100	5.52046500	1.68171300
H	-0.44281400	4.78152000	2.99614700
C	4.78110100	3.84668900	0.91723800
H	4.02816600	4.56979800	0.61220100
H	5.10584400	4.07148900	1.93983900
H	5.64242300	3.95286700	0.25180900

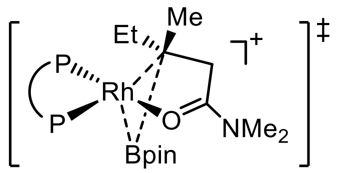

G_{sol} = -2998.08954067 Hatree

Rh	-0.18399400	-0.32451800	-0.56888700
C	-2.03465900	-0.86150300	-2.03108800
B	-2.21263200	-0.74969800	-0.09002700
H	-2.79202100	-2.88732600	-1.75406300
C	-3.50114000	-0.38392700	-2.28769800
H	-3.59646700	0.65484200	-1.96216700
H	-4.16945300	-0.96734800	-1.65027000
C	-1.96005000	-2.39223700	-2.26315300
H	-2.07316700	-2.59377800	-3.33506400
C	-0.68341700	-3.04038100	-1.77452100
O	0.13555900	-2.40115900	-1.05937100
N	-0.41130100	-4.31516200	-2.11003600
C	0.84643700	-4.94120300	-1.69681000
H	1.23557700	-5.53672200	-2.52755000
H	0.68532000	-5.60398600	-0.83874100
H	1.57038200	-4.17425800	-1.43152900
C	-3.39449700	-1.87517400	1.56437900
C	-3.94011200	-0.39209700	1.43727800
O	-2.54090900	-2.01084500	0.39252000
O	-2.97330000	0.22924100	0.53319800
C	-5.33152900	-0.27905300	0.79775100
H	-5.52546900	0.76887600	0.55383500
H	-6.10709700	-0.61651700	1.49160600
H	-5.42677100	-0.85866100	-0.12057700
C	-4.46631600	-2.96434500	1.49661300
H	-5.16278000	-2.88134200	2.33693200
H	-3.99124300	-3.94783900	1.55336000
H	-5.03970100	-2.91880400	0.56901100
C	-3.95556300	0.39507600	2.74950600
H	-4.63665800	-0.07093500	3.46854800
H	-4.31560700	1.41126100	2.56485400
H	-2.96796900	0.46555500	3.20344700
C	-2.51319800	-2.10818000	2.79657000

H	-2.06774400	-3.10348600	2.73333300
H	-3.09454100	-2.05221900	3.72135900
H	-1.69489300	-1.38727700	2.85255600
P	1.76729200	-0.50822600	0.75308800
P	-0.36693800	1.98430400	-0.28693300
O	3.40928300	2.86788800	-1.67044200
O	4.44425300	1.87116700	0.11689000
C	1.09657000	2.85359100	-1.06689900
H	1.16670200	3.87417800	-0.67954200
H	0.89144600	2.93633600	-2.13649900
C	2.43484000	2.14289900	-0.92961600
H	2.34232800	1.12512000	-1.33478600
C	3.07895000	2.07734200	0.45894500
H	2.95669500	3.05690000	0.95037200
C	2.62374300	0.99388900	1.44448300
H	3.50395500	0.66145700	2.00273900
H	1.92806600	1.43874100	2.15703600
C	4.70408700	2.62743500	-1.07559100
C	5.32994600	3.97788200	-0.73934700
H	5.43187000	4.58171300	-1.64509600
H	6.31988500	3.83668100	-0.29775200
H	4.70309100	4.52563100	-0.03096000
C	5.56685500	1.77961700	-1.99959700
H	5.76865600	2.32190200	-2.92699800
H	5.05759300	0.84260800	-2.23538400
H	6.51960900	1.54646800	-1.51625000
C	1.50037500	-1.54660300	2.25306100
C	1.10233600	-2.88242200	2.06736400
H	0.92842900	-3.25236900	1.06326100
C	0.92227200	-3.72469200	3.16239800
H	0.62529000	-4.75736100	3.00357800
C	1.12104400	-3.24640100	4.46031500
H	0.98181700	-3.90477200	5.31240600
C	1.50061000	-1.91914300	4.65545000
H	1.65996000	-1.53884700	5.66006300
C	1.69228200	-1.07215400	3.56023500
H	2.00116800	-0.04960600	3.74373900
C	3.12400600	-1.40022300	-0.13015600
C	3.11138100	-1.53807900	-1.52502300
H	2.27000200	-1.15984200	-2.09599900
C	4.16863400	-2.16914000	-2.18494100
H	4.14748100	-2.26673000	-3.26650700
C	5.24729000	-2.67256500	-1.45609500
H	6.06962900	-3.16287700	-1.96838700
C	5.26506700	-2.54574900	-0.06524500

H	6.10018700	-2.93758000	0.50745400
C	4.21071800	-1.91564900	0.59437700
H	4.23505400	-1.83312300	1.67688400
C	-0.40916800	2.65911700	1.43327400
C	-0.56815200	1.79765000	2.52638500
H	-0.67391700	0.73302900	2.34901200
C	-0.58859600	2.29831900	3.83094500
H	-0.70864400	1.61614100	4.66752900
C	-0.45775800	3.66890100	4.05628400
H	-0.47305200	4.05947600	5.06928200
C	-0.31402400	4.53933600	2.97255600
H	-0.22137200	5.60808300	3.14010900
C	-0.29307700	4.03981200	1.67081900
H	-0.20291500	4.73545100	0.84185200
C	-1.77270900	2.90058400	-1.07926500
C	-2.92921500	3.17185100	-0.32883100
H	-2.99164100	2.84216300	0.70002000
C	-4.00045600	3.86161100	-0.89536200
H	-4.88130800	4.06887300	-0.29484200
C	-3.94129200	4.29065100	-2.22263000
H	-4.77464000	4.83148300	-2.66066900
C	-2.80017900	4.02713900	-2.97966000
H	-2.73693800	4.36345500	-4.01011400
C	-1.72518400	3.33852800	-2.41372600
H	-0.85113100	3.16281600	-3.03120000
C	-1.14205800	-0.18105600	-3.11180400
H	-0.07271600	-0.40623400	-3.02817100
H	-1.45066000	-0.54449100	-4.10060700
H	-1.26607300	0.90160500	-3.11933900
C	-4.02346700	-0.49866200	-3.73240300
H	-3.93132000	-1.51045300	-4.14198700
H	-5.08827300	-0.24555500	-3.74299100
H	-3.52329900	0.19005200	-4.41756300
C	-1.32080500	-5.18365300	-2.85570800
H	-2.31686400	-4.75354900	-2.92451700
H	-0.93945200	-5.36741900	-3.86682500
H	-1.40171900	-6.14479300	-2.33851300

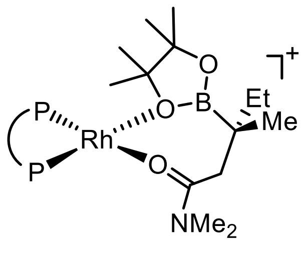

G_{sol} = -2998.12908358 Hatree

Rh	-0.35926600	0.06533000	0.12014900
C	-2.96604100	-1.60539200	-1.80802700
B	-3.31535500	-0.74884500	-0.51536100
H	-3.44480300	-3.58258800	-1.05735800
C	-4.29558000	-1.72674000	-2.62656800
H	-4.61117600	-0.71338800	-2.90929300
H	-5.08008500	-2.11031400	-1.96460600
C	-2.54454300	-3.02447100	-1.34324700
H	-2.09820500	-3.57281400	-2.18142500
C	-1.59530900	-3.08219500	-0.14736300
O	-1.20710400	-2.03927300	0.43800400
N	-1.23691700	-4.29872900	0.31485700
C	-0.37001400	-4.44018900	1.48462100
H	0.58246400	-4.89418200	1.19358000
H	-0.85943000	-5.08660900	2.22090200
H	-0.18308800	-3.46596600	1.92427700
C	-4.41903300	-0.20466900	1.42749400
C	-3.58876000	1.03792600	0.93248400
O	-4.38619300	-1.07908800	0.26241600
O	-2.68120500	0.41735600	-0.07785900
C	-4.43438700	2.05981500	0.16909200
H	-3.78181100	2.79679200	-0.30011800
H	-5.11425300	2.58449600	0.84591900
H	-5.03123300	1.58085000	-0.61194000
C	-5.87956700	0.09485500	1.75996700
H	-5.95159800	0.80948900	2.58597300
H	-6.37827200	-0.82743600	2.06949200
H	-6.41915900	0.49686700	0.90135900
C	-2.76528000	1.71924100	2.01543500
H	-3.42454000	2.06671100	2.81786900
H	-2.25059100	2.59062100	1.60634000
H	-2.02109300	1.04406700	2.44144600
C	-3.76324600	-0.97242200	2.58159400
H	-4.31420700	-1.90344100	2.74120100
H	-3.79133800	-0.39858300	3.51204700
H	-2.72724000	-1.22723500	2.34820900
P	1.71090300	-0.64515500	0.75038200
P	0.35895900	2.13818200	-0.44725500
O	3.21892100	1.00211000	-3.17699200
O	4.50088800	0.01549500	-1.54800100
C	1.52065500	2.14184200	-1.91657000
H	2.08175200	3.07995000	-1.95452700
H	0.89025200	2.11705600	-2.80920900
C	2.46689000	0.95409600	-1.96994400
H	1.87595100	0.02838200	-1.93014100
C	3.57517700	0.89334800	-0.91740300
H	4.01838100	1.89841000	-0.81786600
C	3.24876900	0.38318400	0.49082000
H	4.11017300	-0.20491600	0.81908700
H	3.15031300	1.23103500	1.17258000
C	4.45899000	0.29489400	-2.95780700
C	5.60643200	1.21389900	-3.36425400
H	5.50687700	1.49853800	-4.41520700
H	6.56517400	0.70711600	-3.22648400
H	5.59866900	2.12430900	-2.75941300
C	4.45767000	-1.03212300	-3.70454600
H	4.40226200	-0.85752300	-4.78219900
H	3.60209800	-1.63644200	-3.39441100
H	5.37317600	-1.58900500	-3.48634600
C	1.75408000	-0.88051300	2.58897800
C	0.54959000	-1.16724500	3.25554400
H	-0.37041700	-1.23297500	2.68482200
C	0.52413800	-1.35885500	4.63737300
H	-0.41677900	-1.58055100	5.13265000
C	1.70109600	-1.25901000	5.38176400
H	1.68195300	-1.40374900	6.45767600
C	2.90185800	-0.96617600	4.73508600
H	3.82200300	-0.88076800	5.30540100
C	2.93019900	-0.77775800	3.35132100
H	3.87950200	-0.54498500	2.88261600
C	2.19374600	-2.29355700	0.05850300
C	1.66617400	-2.71867400	-1.16924000
H	0.94478500	-2.09426900	-1.68672000
C	2.05825500	-3.93677800	-1.72896000
H	1.64750200	-4.24796100	-2.68532000
C	2.97976500	-4.74849300	-1.06449900
H	3.28844300	-5.69420000	-1.49997500
C	3.50745600	-4.33589800	0.16141300
H	4.22518200	-4.96124400	0.68402800
C	3.11716800	-3.11826200	0.71999700
H	3.52912200	-2.82111200	1.67900600
C	1.20709500	3.15109100	0.84394100
C	1.04846300	2.79934800	2.19348800
H	0.46258700	1.92172400	2.44793200
C	1.64721900	3.55791900	3.20196900
H	1.51777100	3.26994700	4.24090100
C	2.41224800	4.67773300	2.87384300
H	2.88179900	5.26614300	3.65633400
C	2.56993500	5.04312500	1.53444300
H	3.15847300	5.91751600	1.27353300

C	1.96879600	4.28943700	0.52641200
H	2.08526100	4.60688200	-0.50489900
C	-0.95706600	3.27641300	-1.07775700
C	-1.32203800	4.44431900	-0.39104100
H	-0.83789600	4.70238300	0.54426100
C	-2.29632600	5.29881300	-0.91374400
H	-2.56045100	6.20323900	-0.37388300
C	-2.91758600	4.99967600	-2.12636000
H	-3.66718100	5.66983700	-2.53596000
C	-2.56858600	3.83364100	-2.81268400
H	-3.04764800	3.59249500	-3.75693600
C	-1.60181200	2.97615100	-2.28959100
H	-1.34955400	2.07125200	-2.83306700
C	-1.87398200	-0.96722300	-2.68877600
H	-0.97177600	-0.71895000	-2.11273600
H	-1.57831300	-1.63222700	-3.50835100
H	-2.23847000	-0.03789500	-3.13843200
C	-4.24493400	-2.58828700	-3.89626700
H	-4.00457400	-3.63463900	-3.67936400
H	-5.22163200	-2.58093500	-4.38967000
H	-3.51276400	-2.21969500	-4.62082600
C	-1.61790200	-5.57197000	-0.29399800
H	-2.32971700	-5.44067700	-1.10350100
H	-0.72768700	-6.07666800	-0.68650000
H	-2.07240300	-6.21809000	0.464277

Copies of NMR Spectra

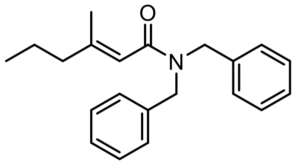

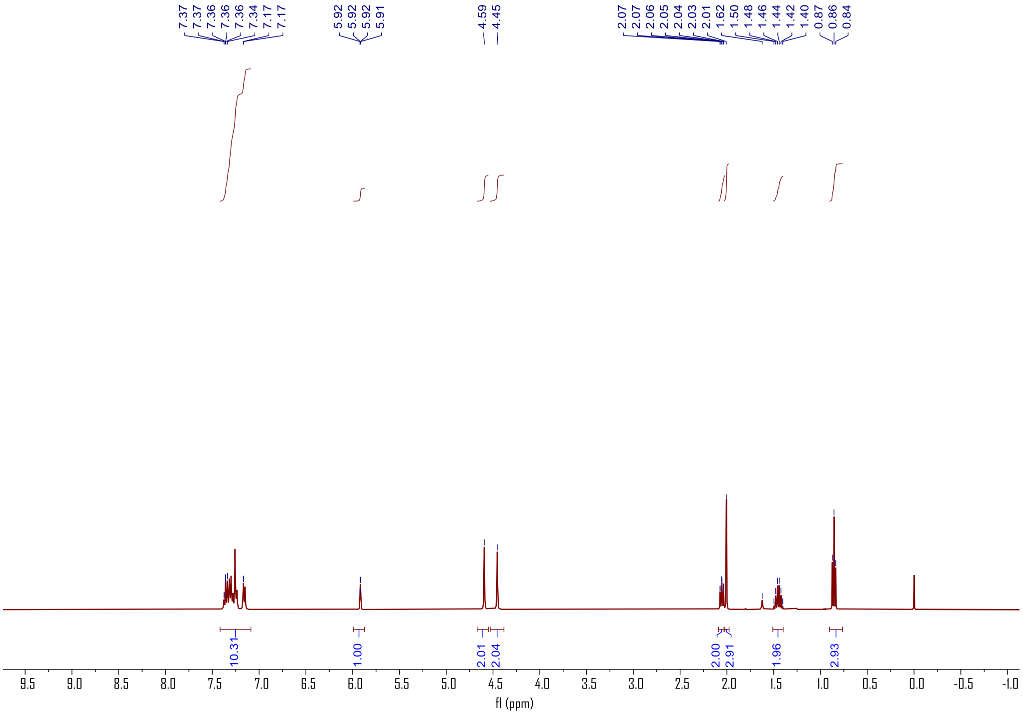

Supplementary Figure 6. ^{1}H NMR (400 MHz, $CDCl_3$) of **1a**.

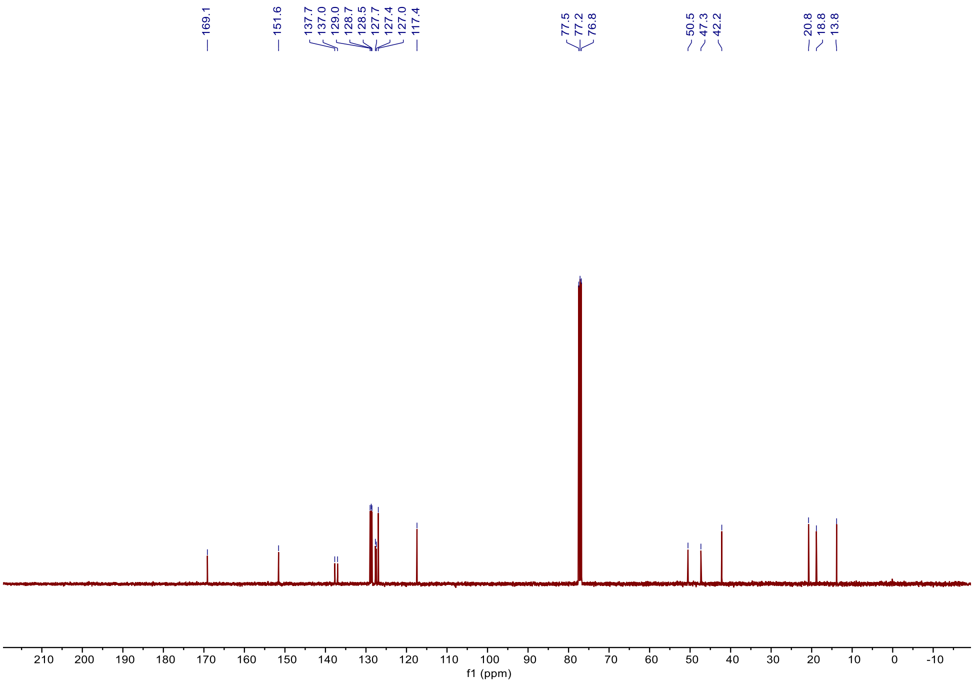

Supplementary Figure 7. ^{13}C NMR (101 MHz, $CDCl_3$) of **1a**.

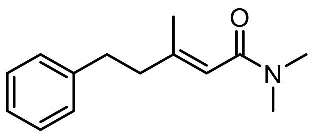

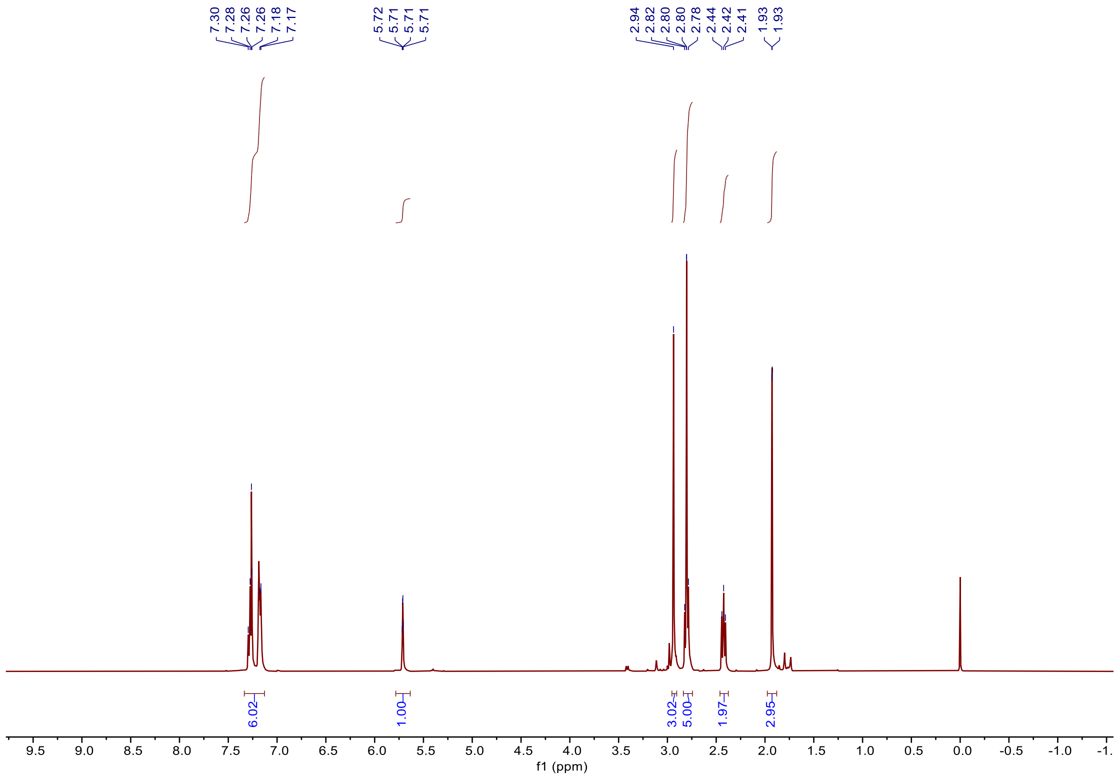

Supplementary Figure 8. ^{1}H NMR (400 MHz, $CDCl_3$) of **1b**.

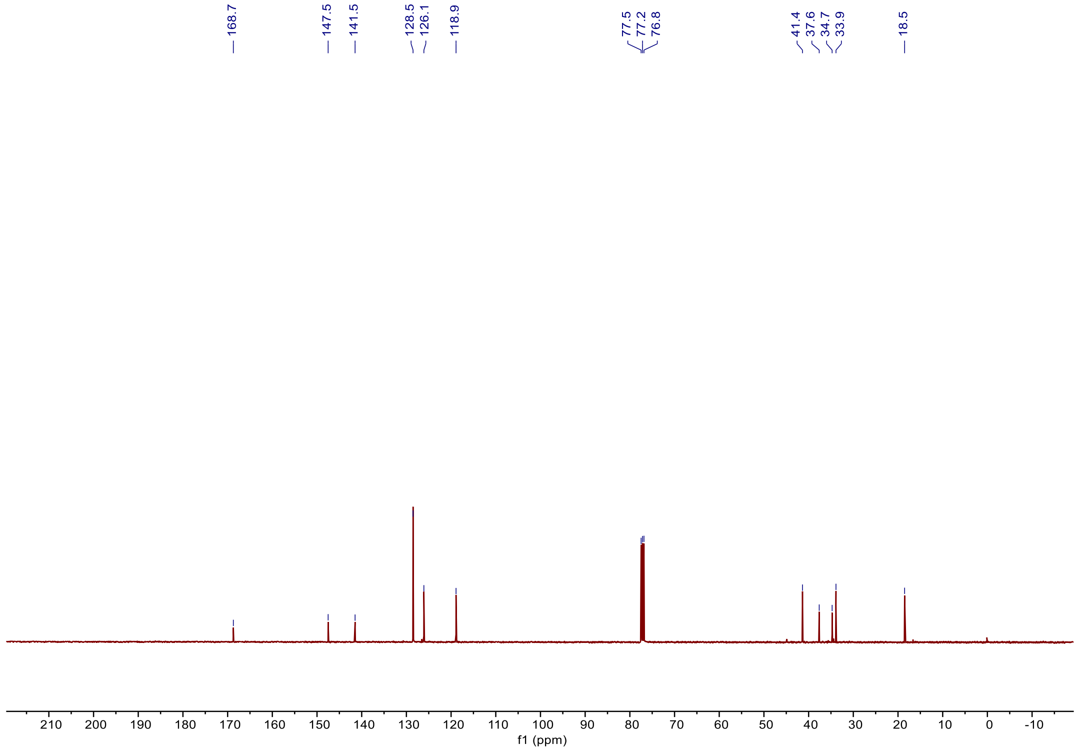

Supplementary Figure 9. ^{13}C NMR (101 MHz, $CDCl_3$) of **1b**.

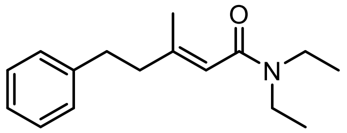

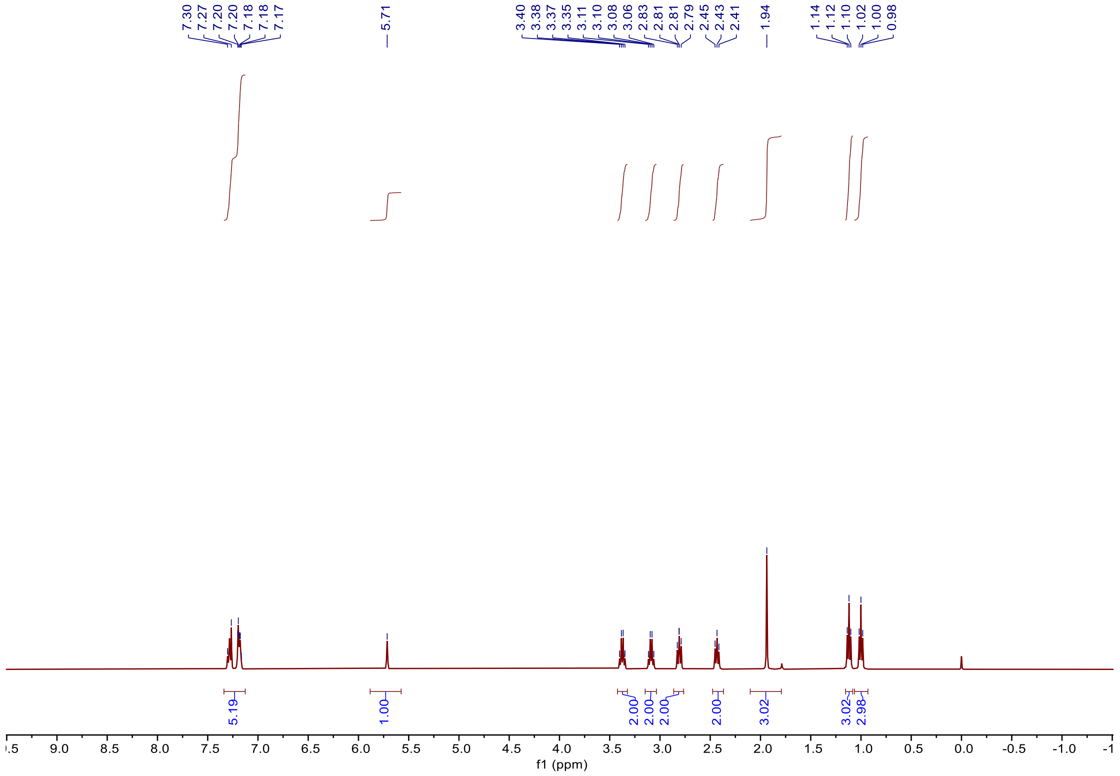

Supplementary Figure 10. ^{1}H NMR (400 MHz, $CDCl_3$) of **1c**.

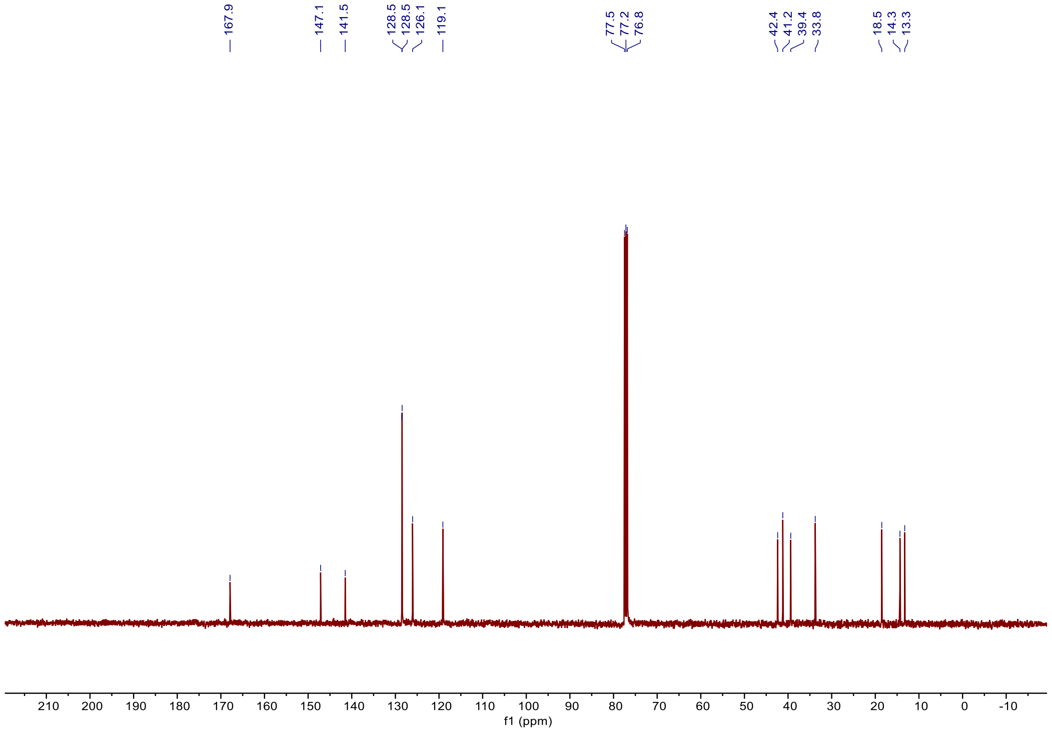

Supplementary Figure 11. ^{13}C NMR (101 MHz, $CDCl_3$) of **1c**.

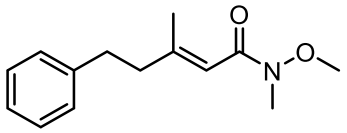

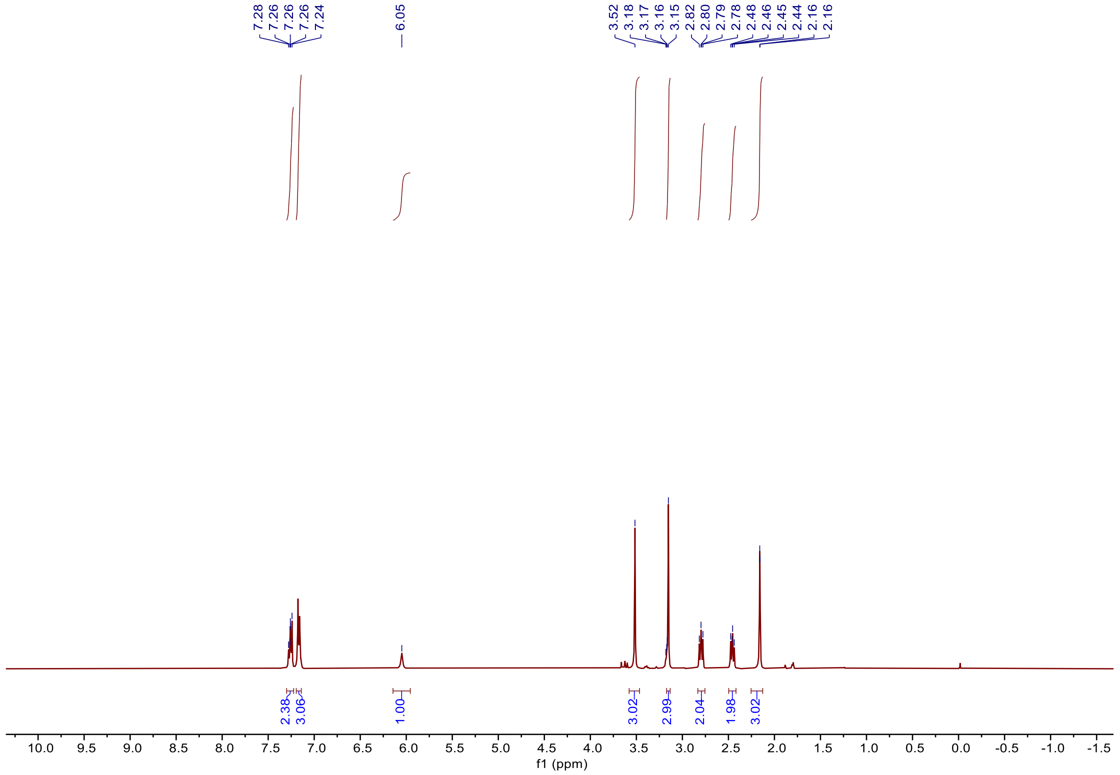

Supplementary Figure 12. ^{1}H NMR (400 MHz, $CDCl_3$) of **1d**.

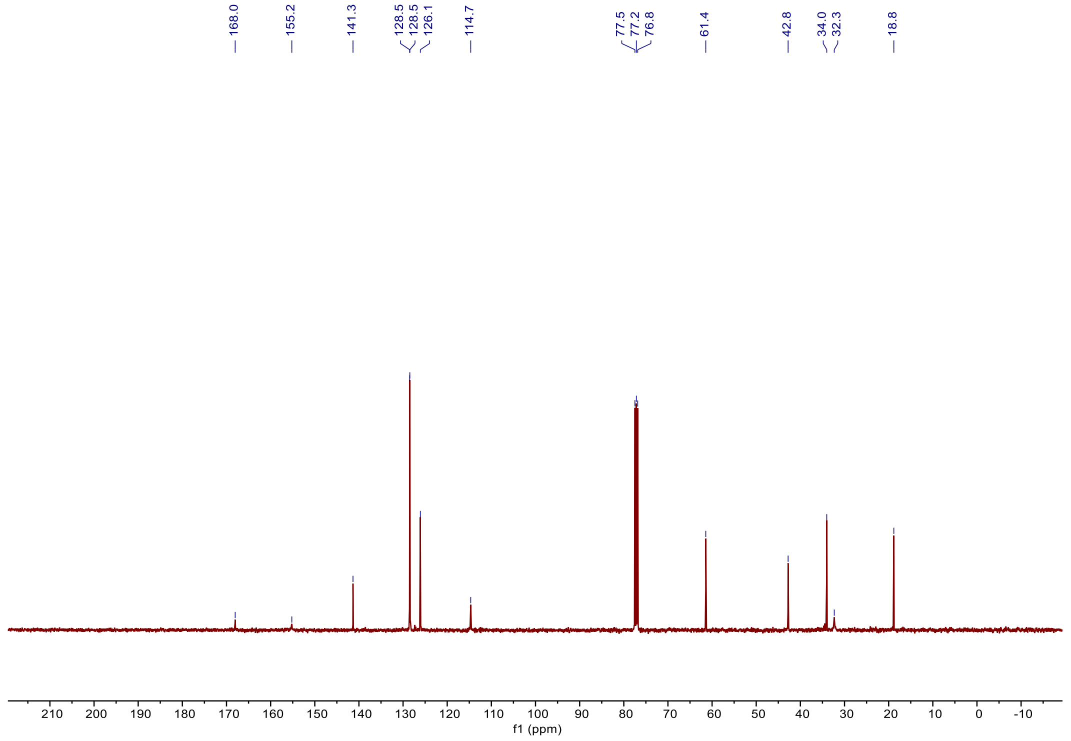

Supplementary Figure 13. ^{13}C NMR (101 MHz, $CDCl_3$) of **1d**.

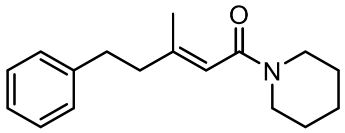

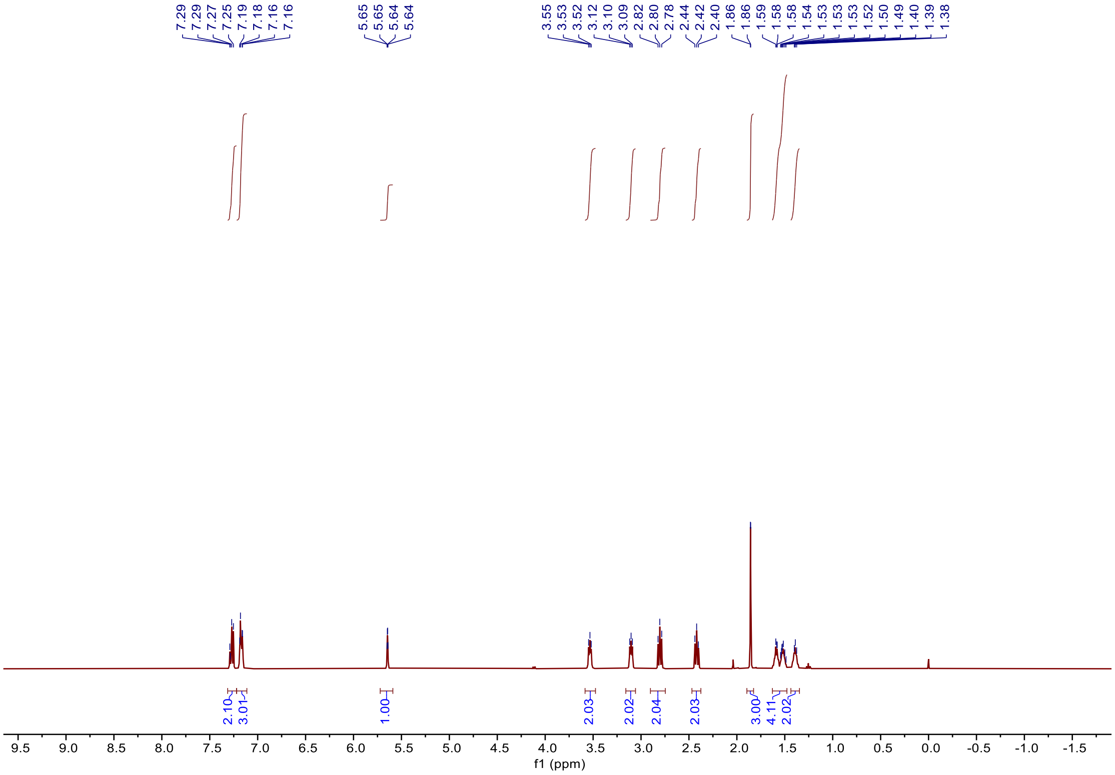

Supplementary Figure 14. ^{1}H NMR (400 MHz, $CDCl_3$) of **1e**.

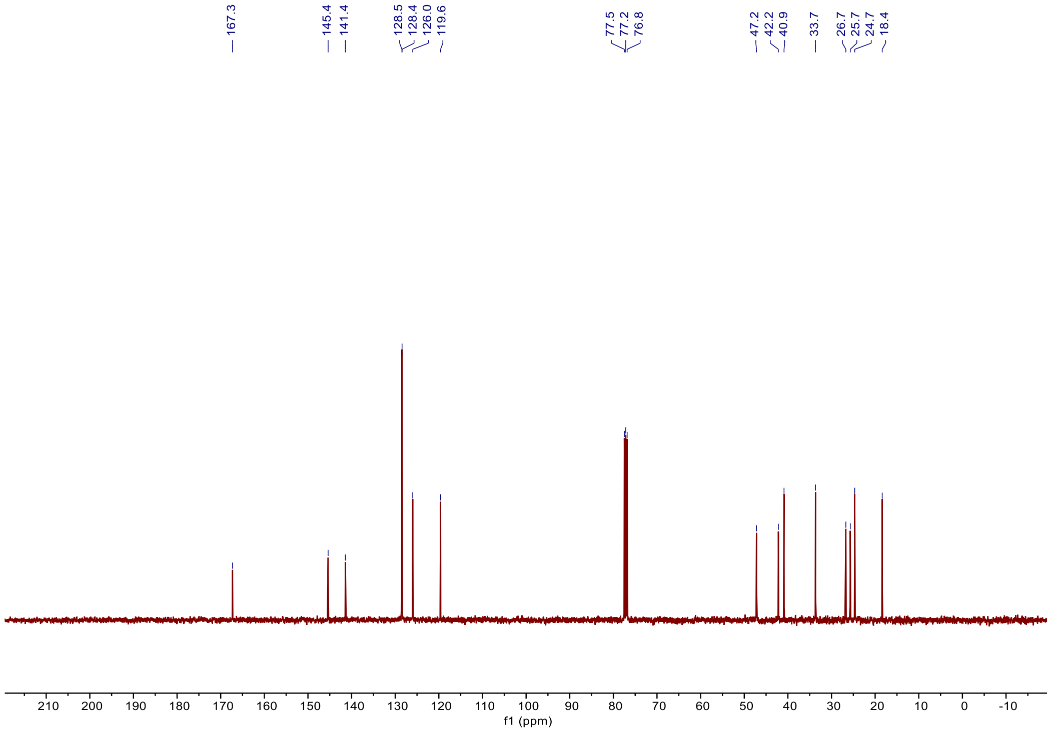

Supplementary Figure 15. ^{13}C NMR (101 MHz, $CDCl_3$) of **1e**.

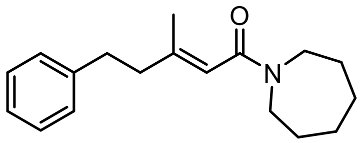

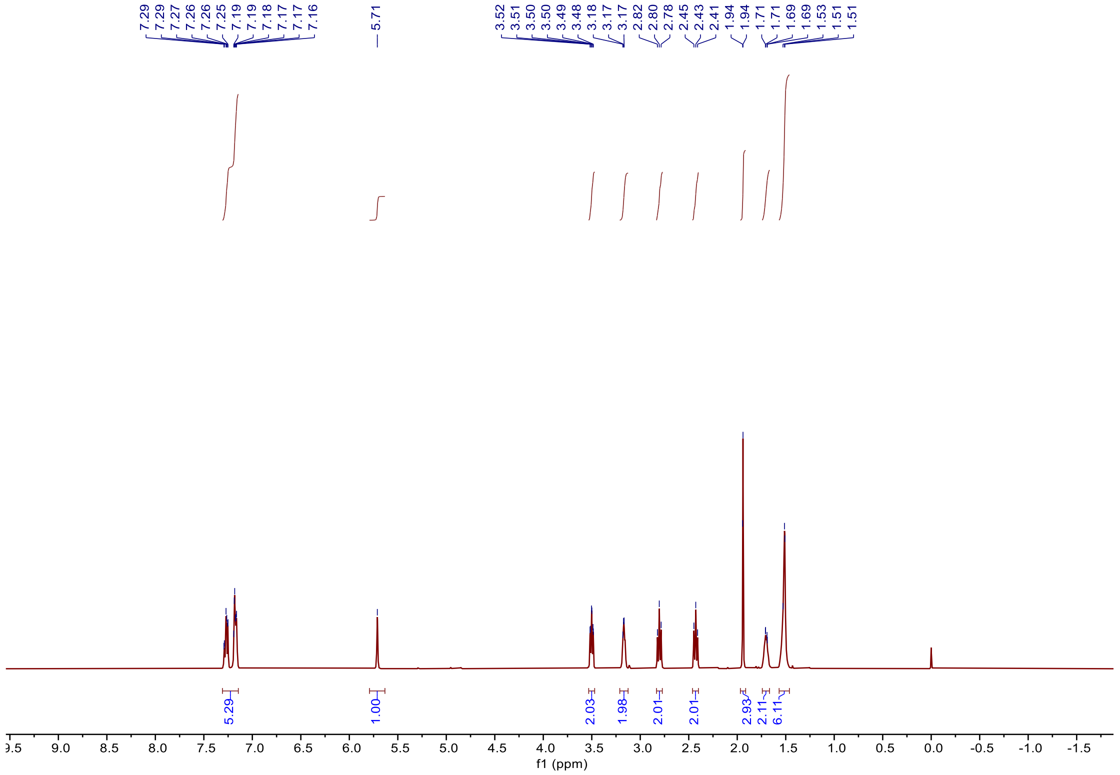

Supplementary Figure 16. ^{1}H NMR (400 MHz, $CDCl_3$) of **1f**.

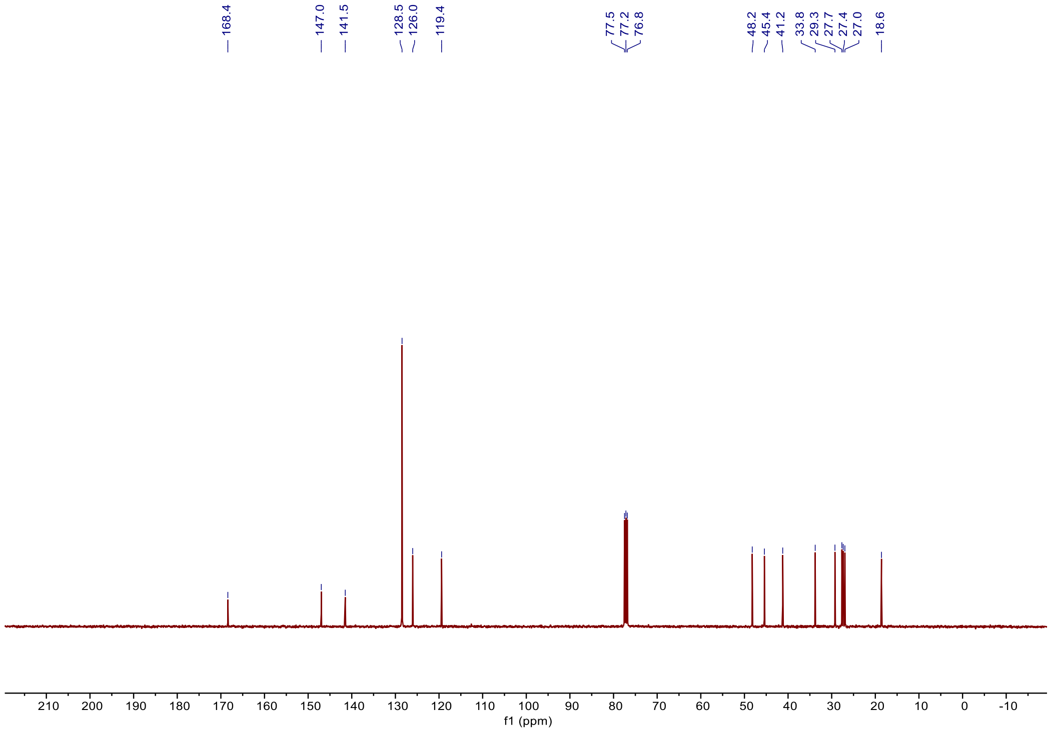

Supplementary Figure 17. ^{13}C NMR (101 MHz, $CDCl_3$) of **1f**.

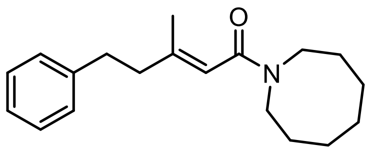

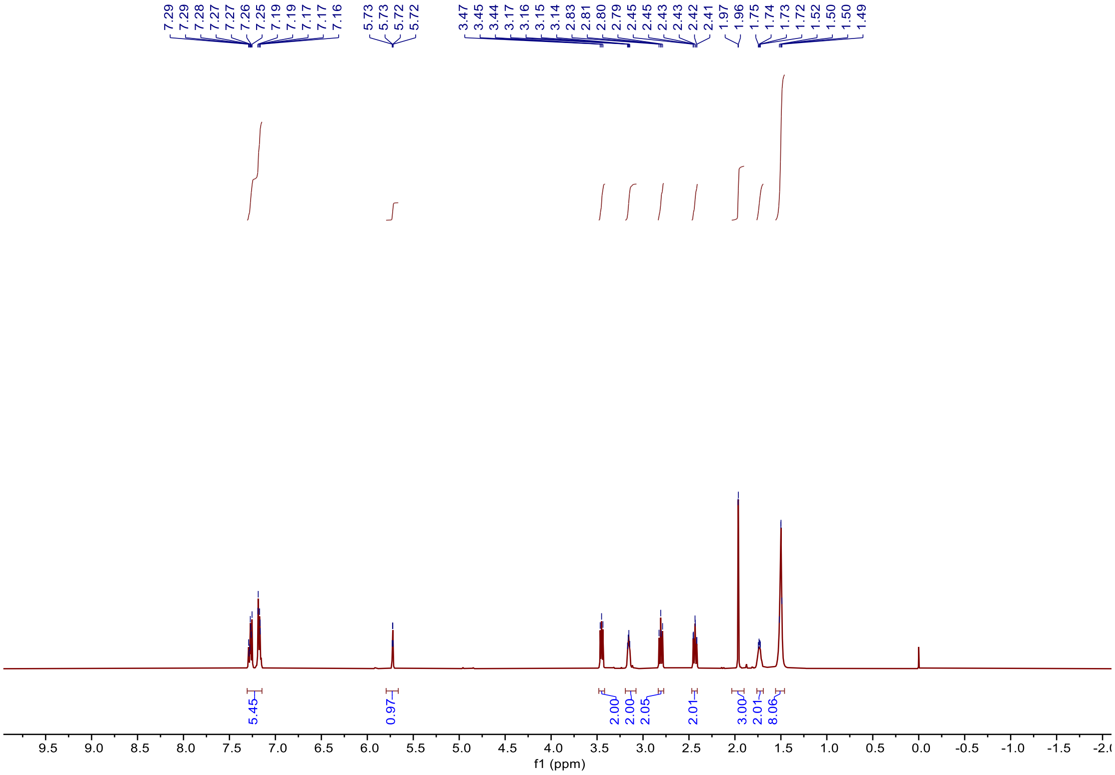

Supplementary Figure 18. ^{1}H NMR (400 MHz, $CDCl_3$) of **1g**.

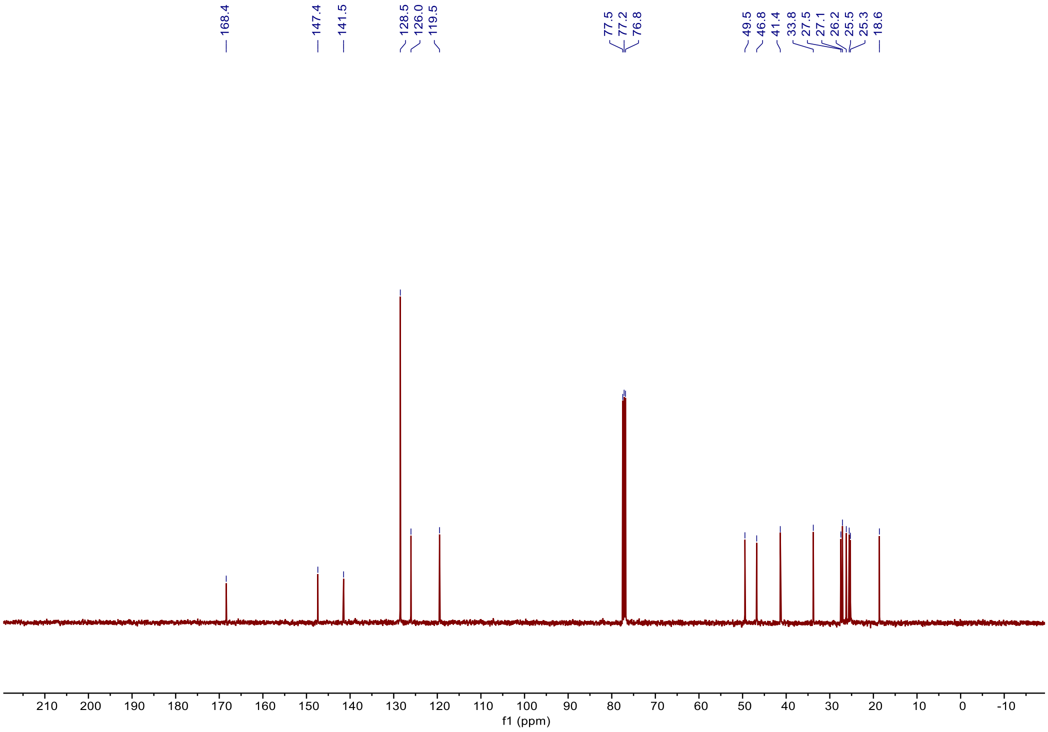

Supplementary Figure 19. ^{13}C NMR (101 MHz, $CDCl_3$) of **1g**.

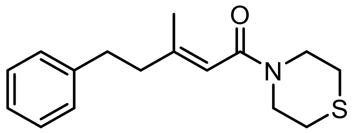

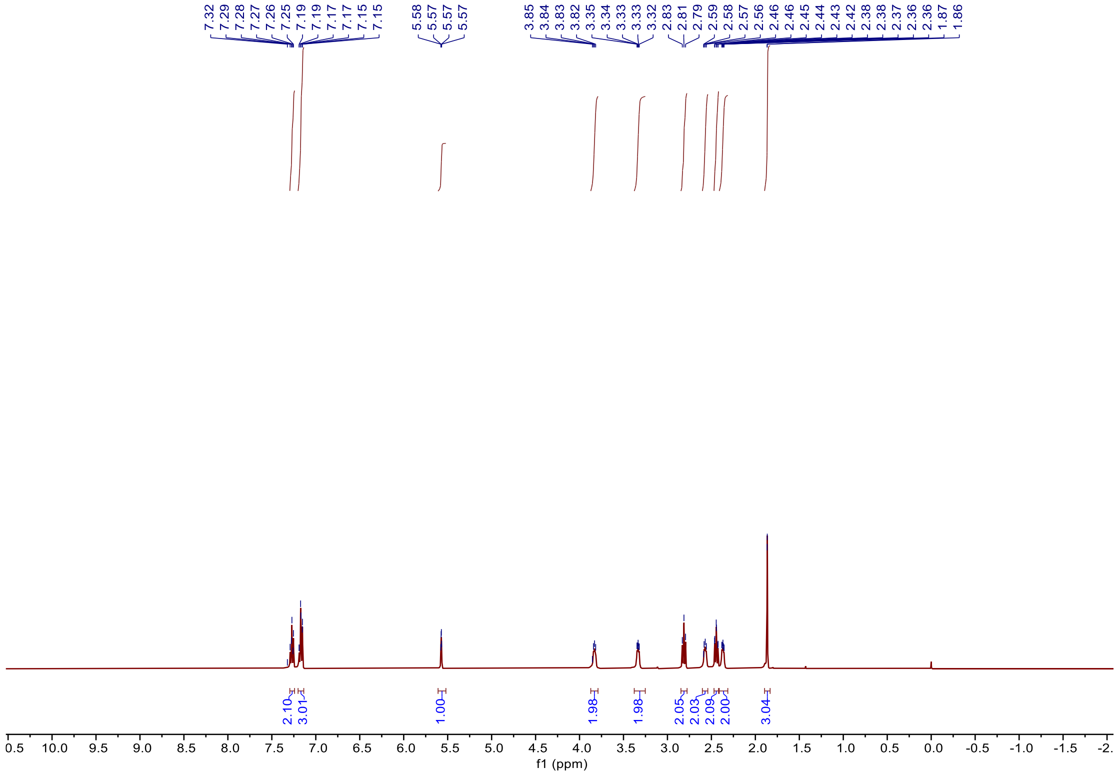

Supplementary Figure 20. ^{1}H NMR (400 MHz, $CDCl_3$) of **1h**.

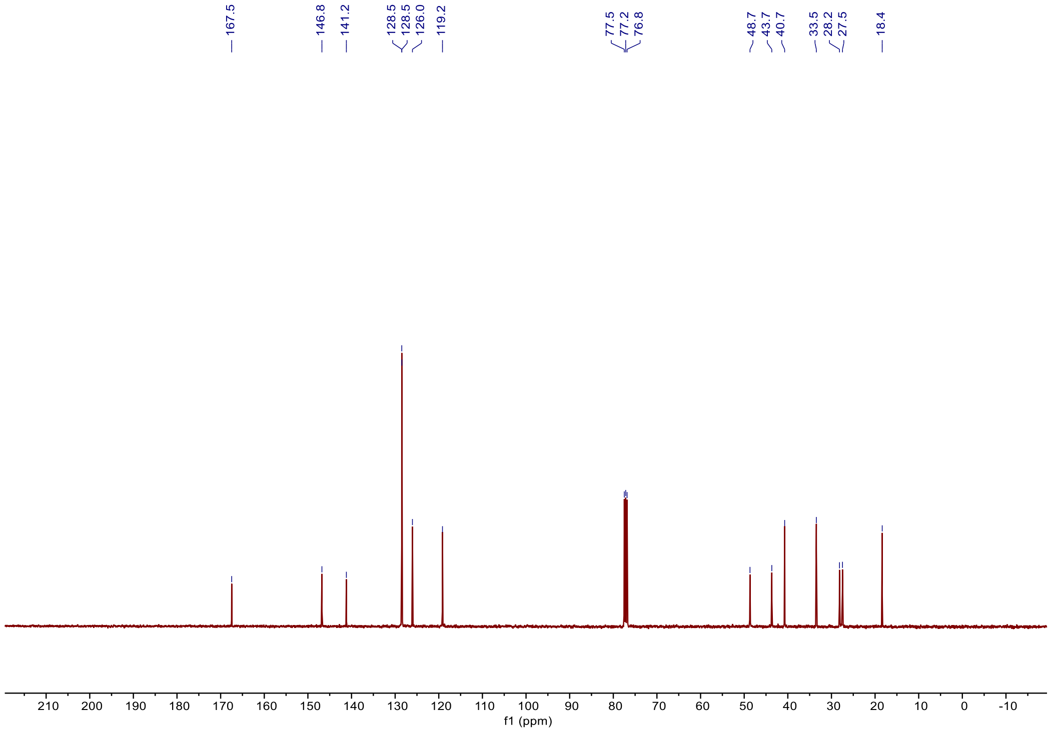

Supplementary Figure 21. ^{13}C NMR (101 MHz, $CDCl_3$) of **1h**.

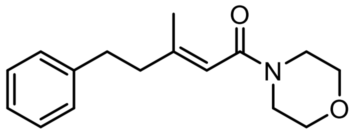

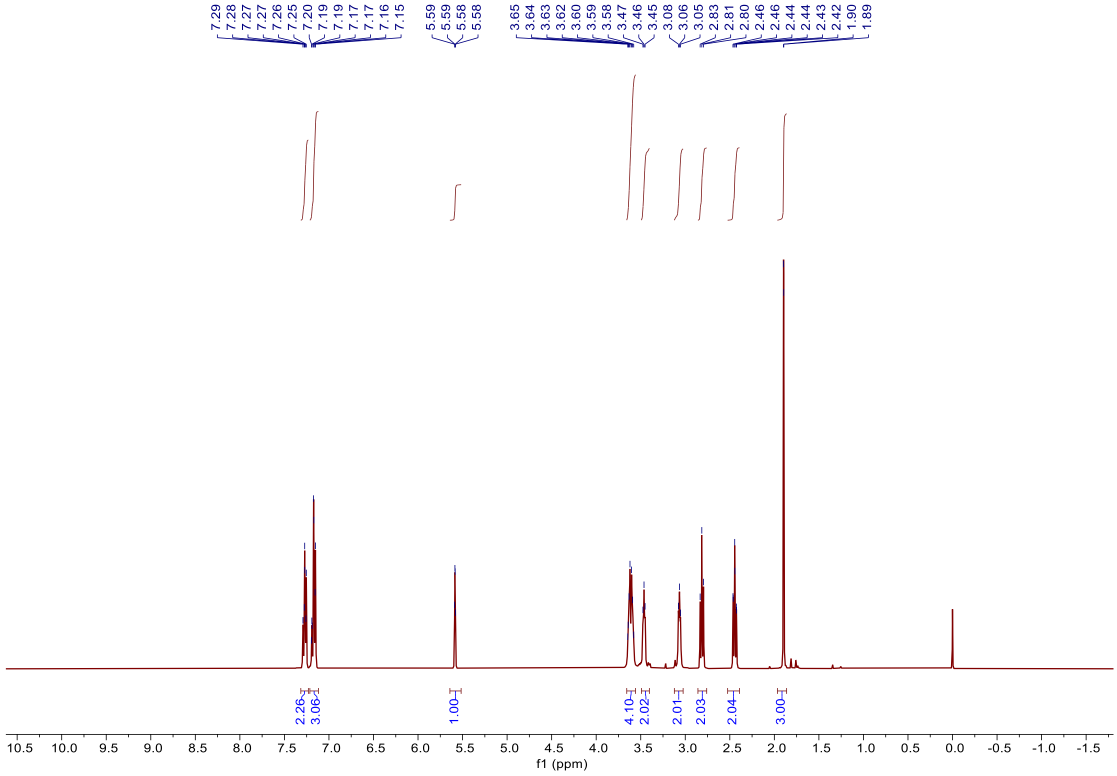

Supplementary Figure 22. ^{1}H NMR (400 MHz, $CDCl_3$) of **1i**.

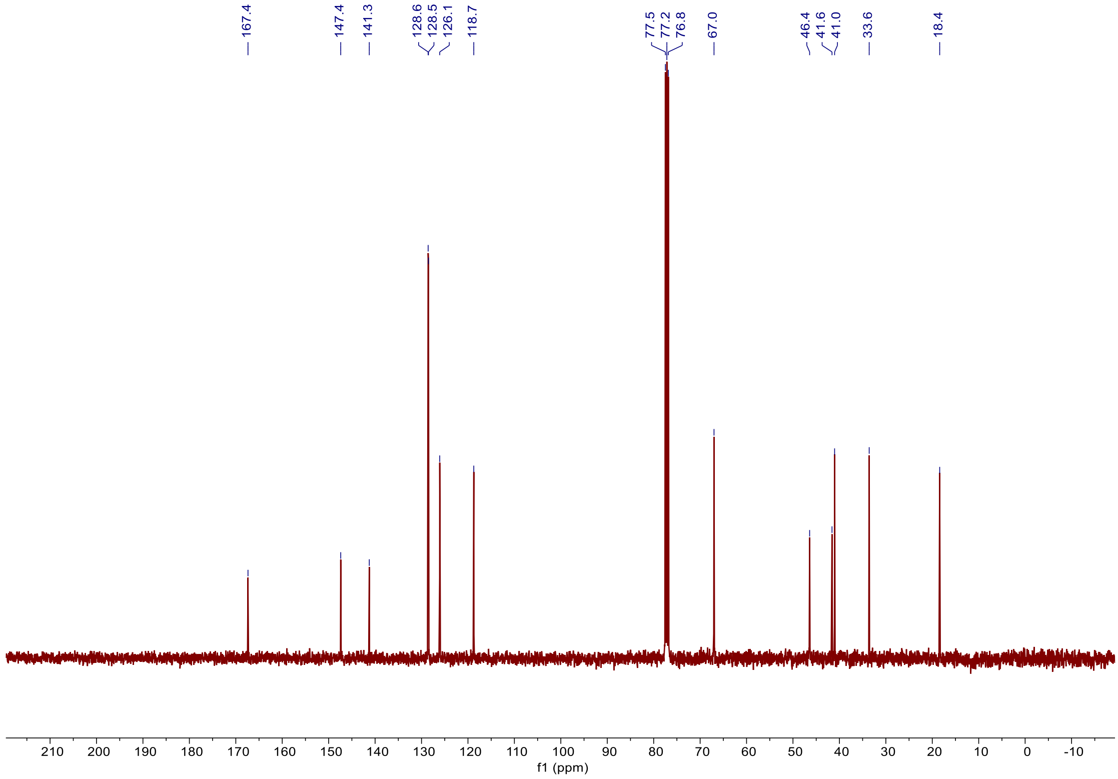

Supplementary Figure 23. ^{13}C NMR (101 MHz, $CDCl_3$) of **1i**.

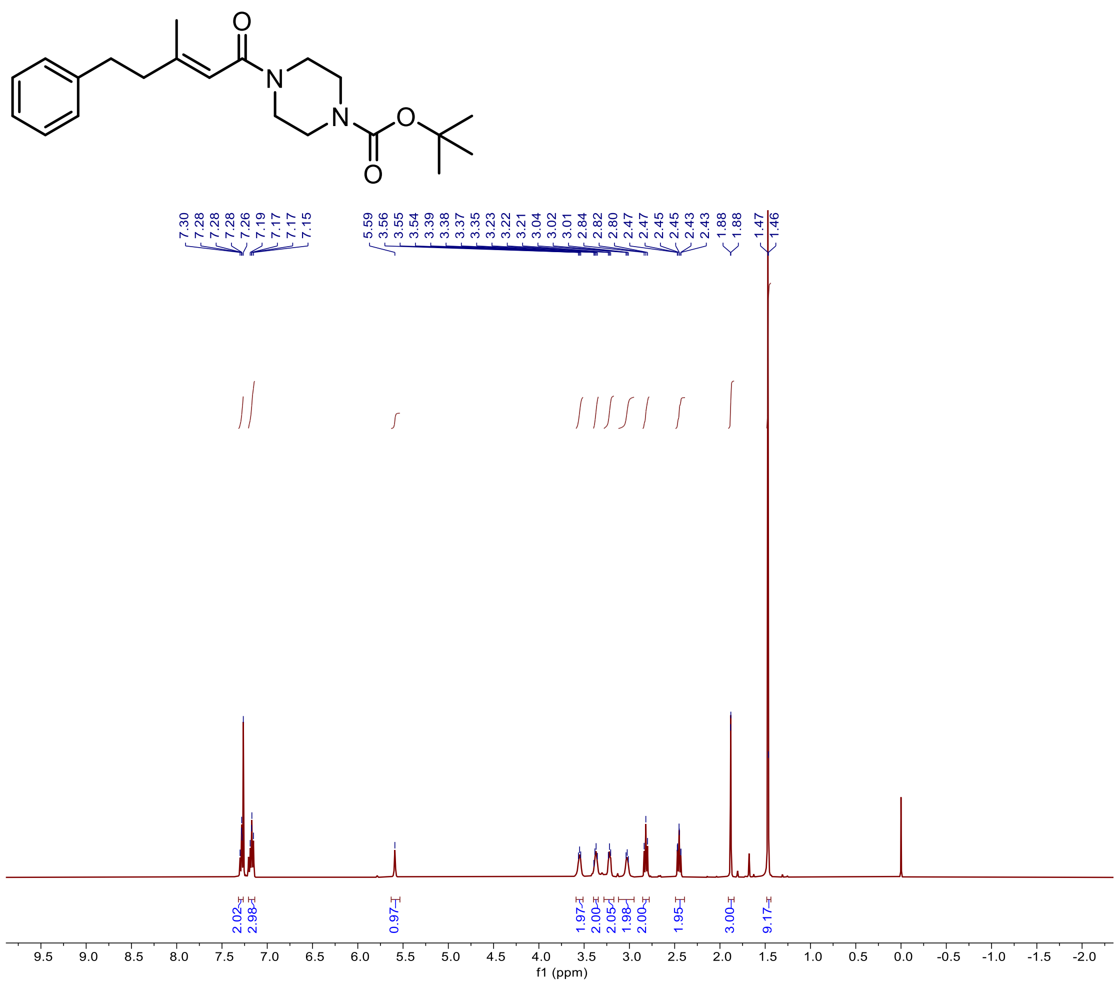

Supplementary Figure 24. ^{1}H NMR (400 MHz, $CDCl_3$) of **1j**.

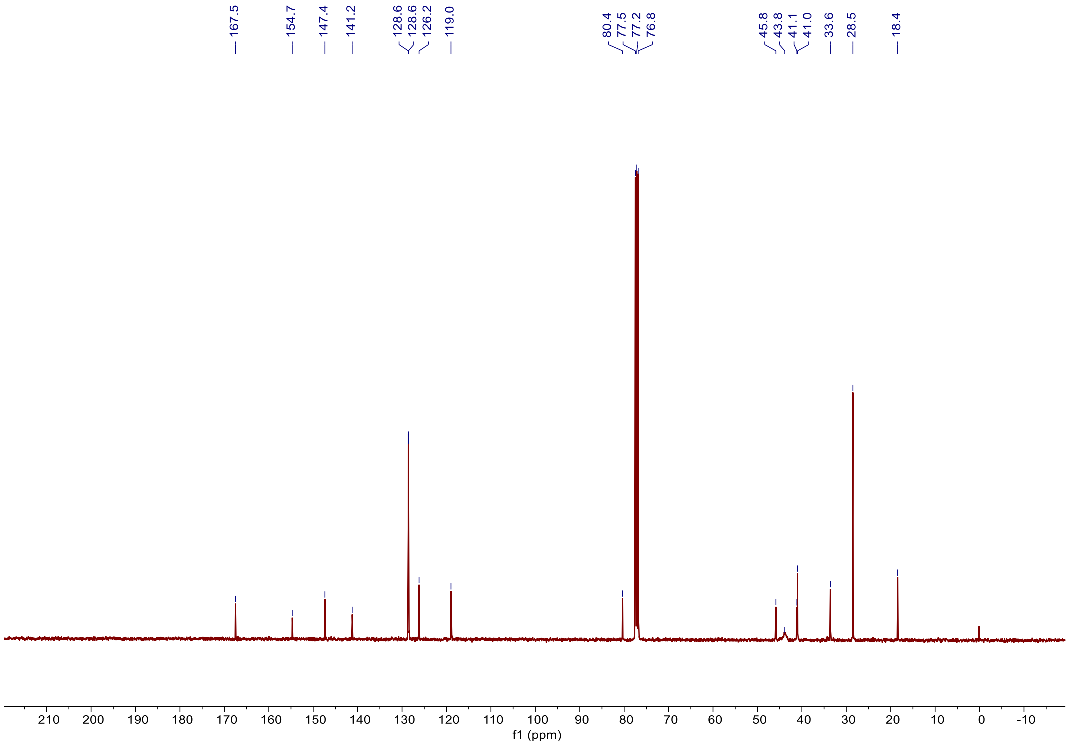

Supplementary Figure 25. ^{13}C NMR (101 MHz, $CDCl_3$) of **1j**.

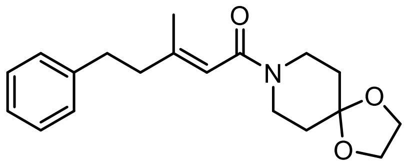

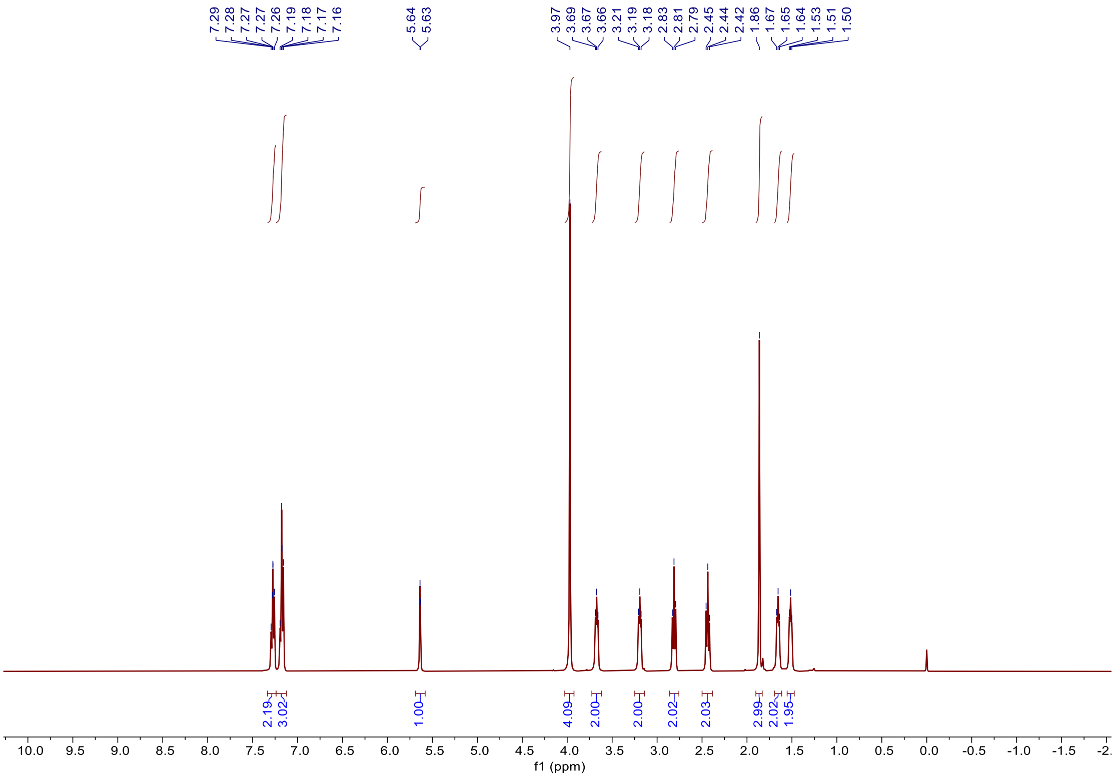

Supplementary Figure 26. ^{1}H NMR (400 MHz, $CDCl_3$) of **1k**.

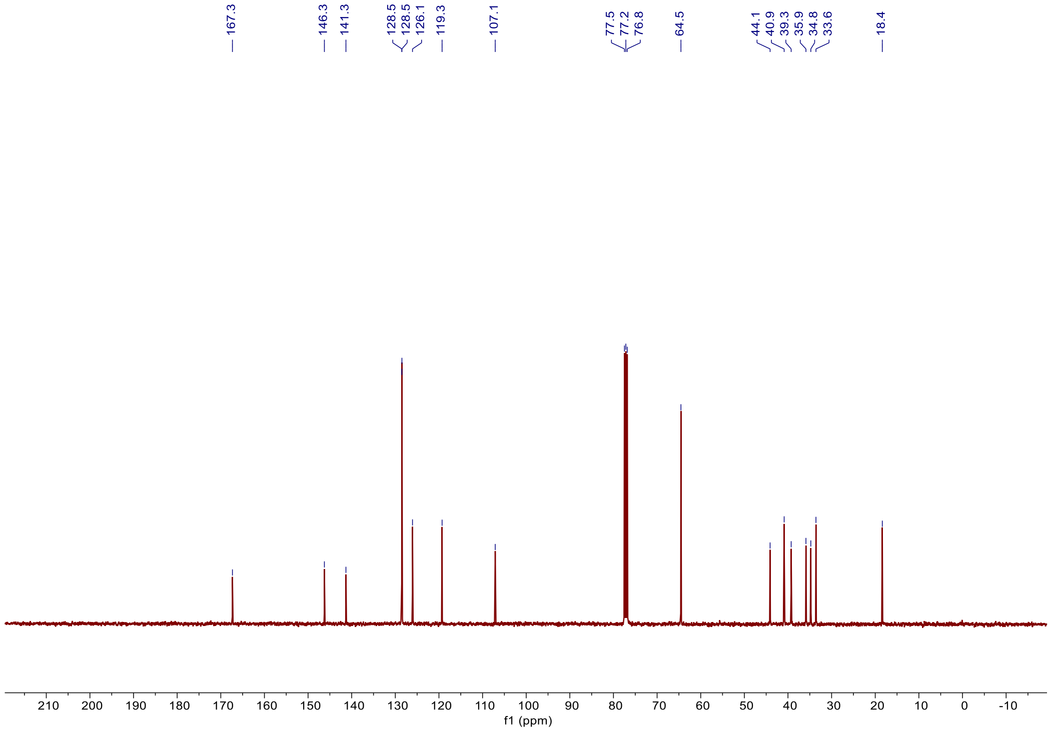

Supplementary Figure 27. ^{13}C NMR (101 MHz, $CDCl_3$) of **1k**.

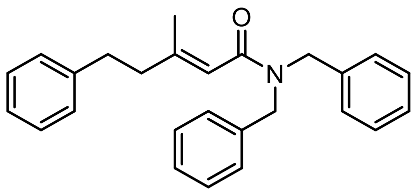

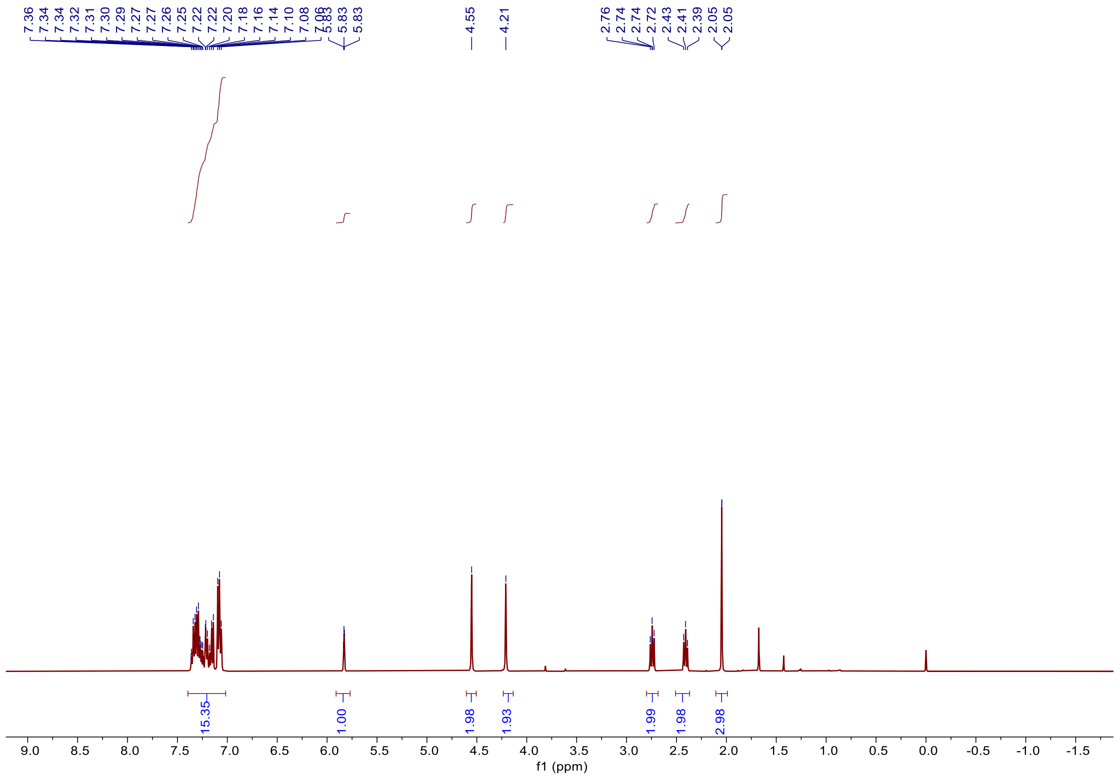

Supplementary Figure 28. ^{1}H NMR (400 MHz, $CDCl_3$) of **1l**.

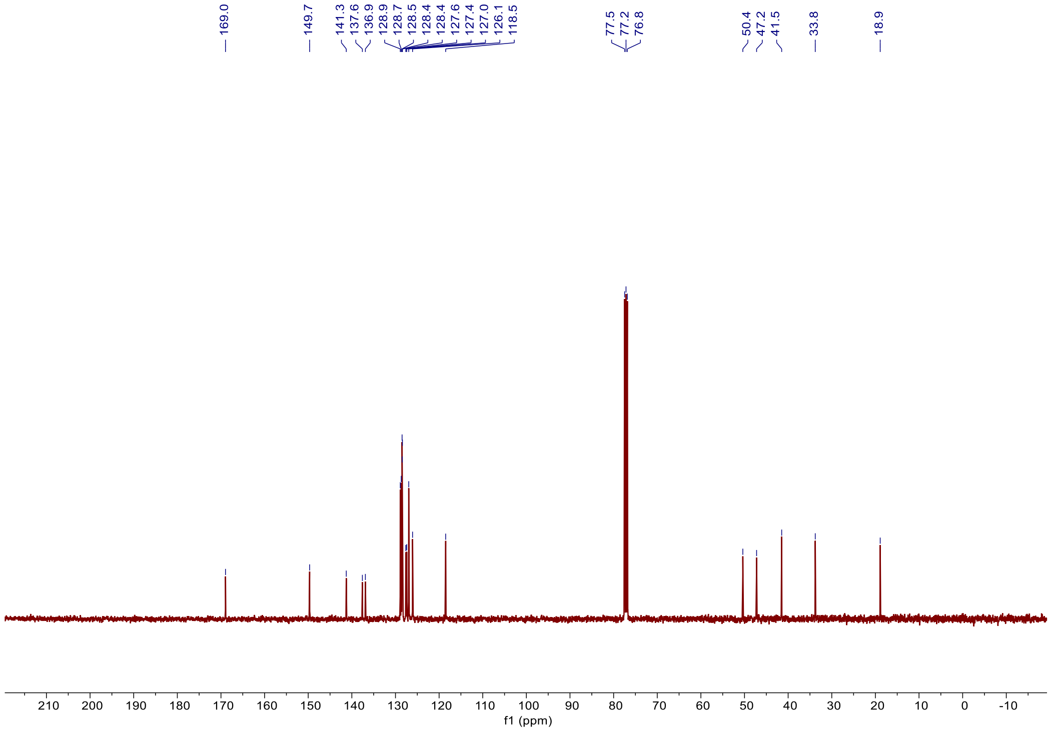

Supplementary Figure 29. ^{13}C NMR (101 MHz, $CDCl_3$) of **1l**.

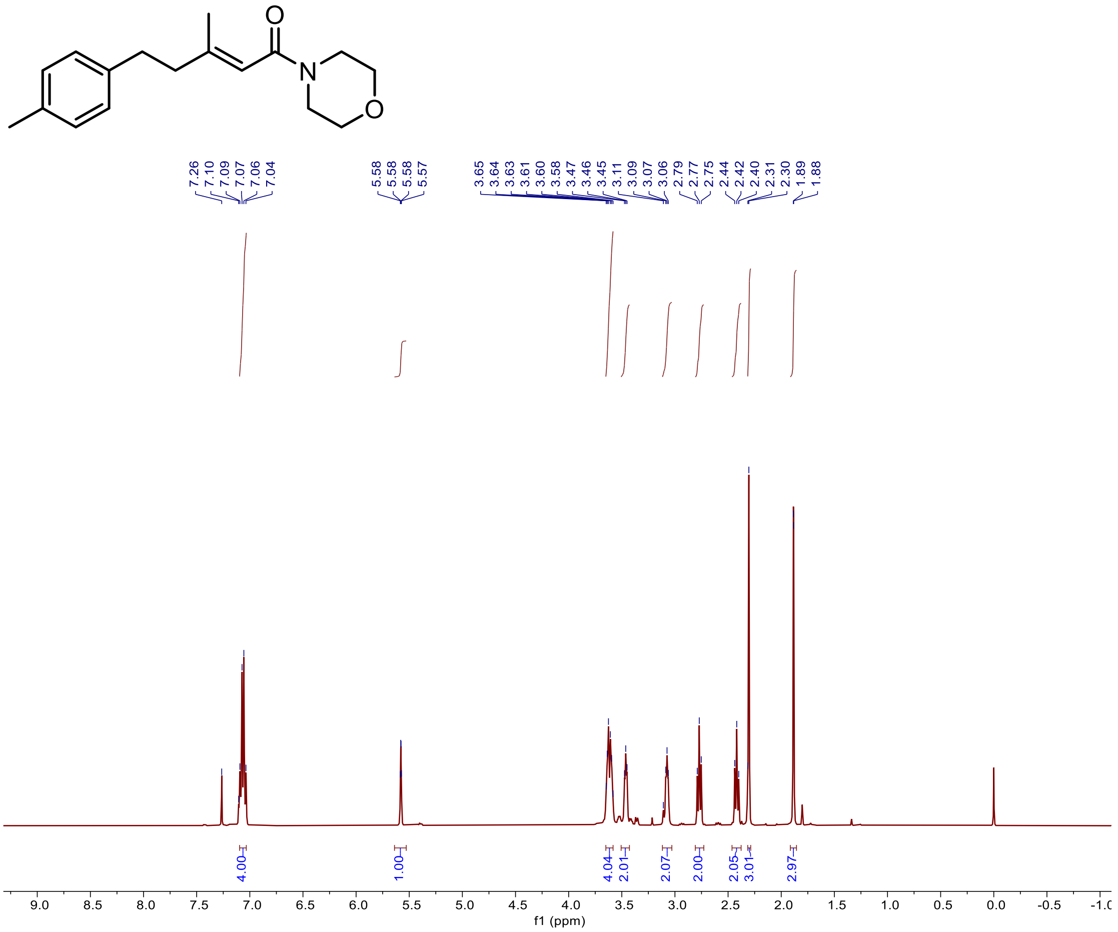

Supplementary Figure 30. ^{1}H NMR (400 MHz, $CDCl_3$) of **1m**.

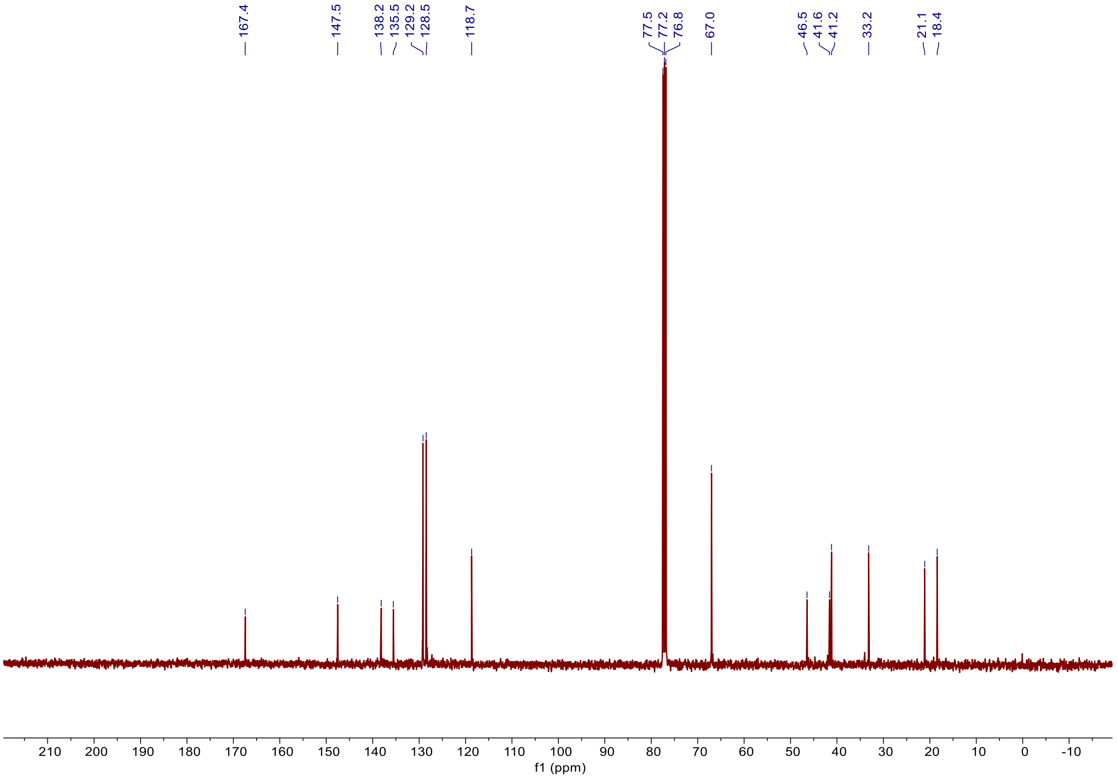

Supplementary Figure 31. ^{13}C NMR (101 MHz, $CDCl_3$) of **1m**.

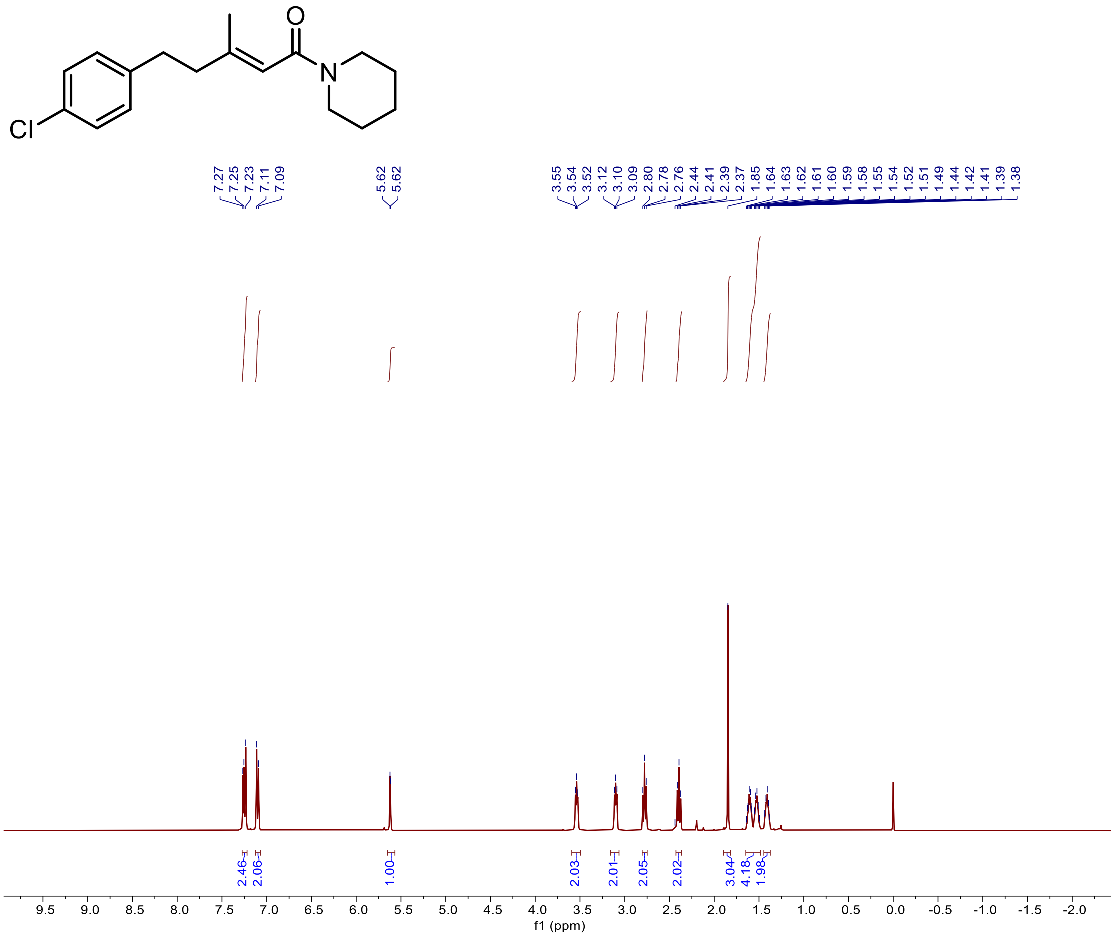

Supplementary Figure 32. ^{1}H NMR (400 MHz, $CDCl_3$) of **1n**.

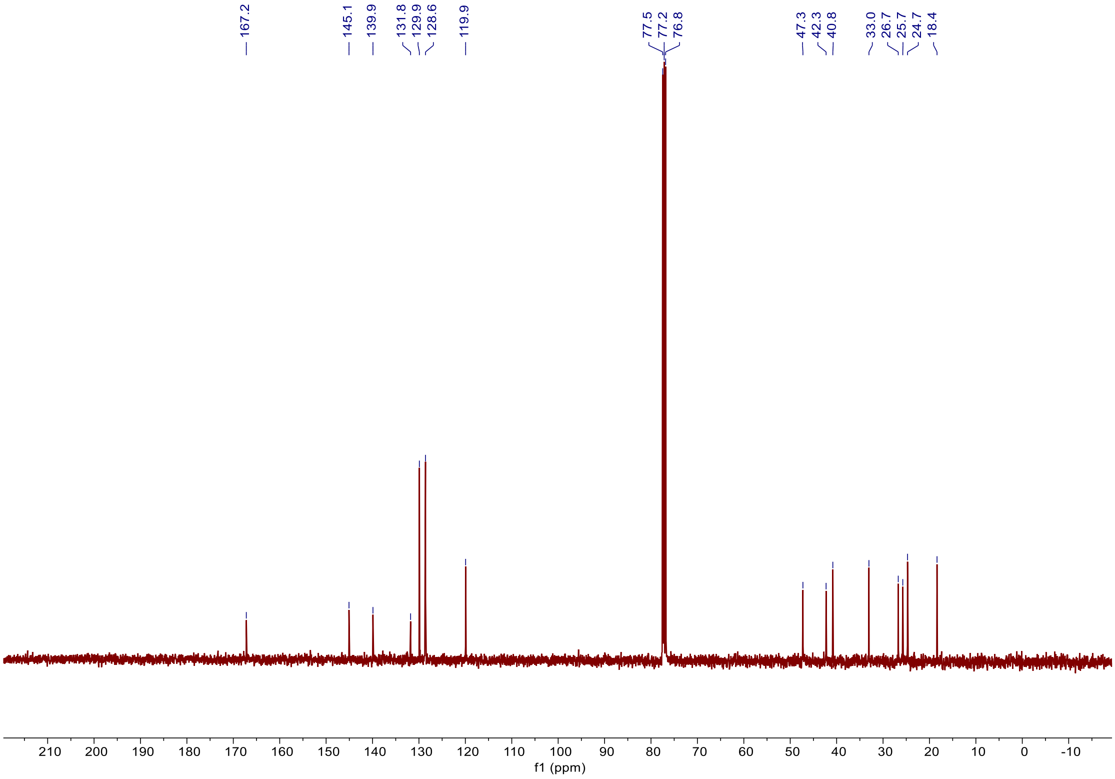

Supplementary Figure 33. ^{13}C NMR (101 MHz, $CDCl_3$) of **1n**.

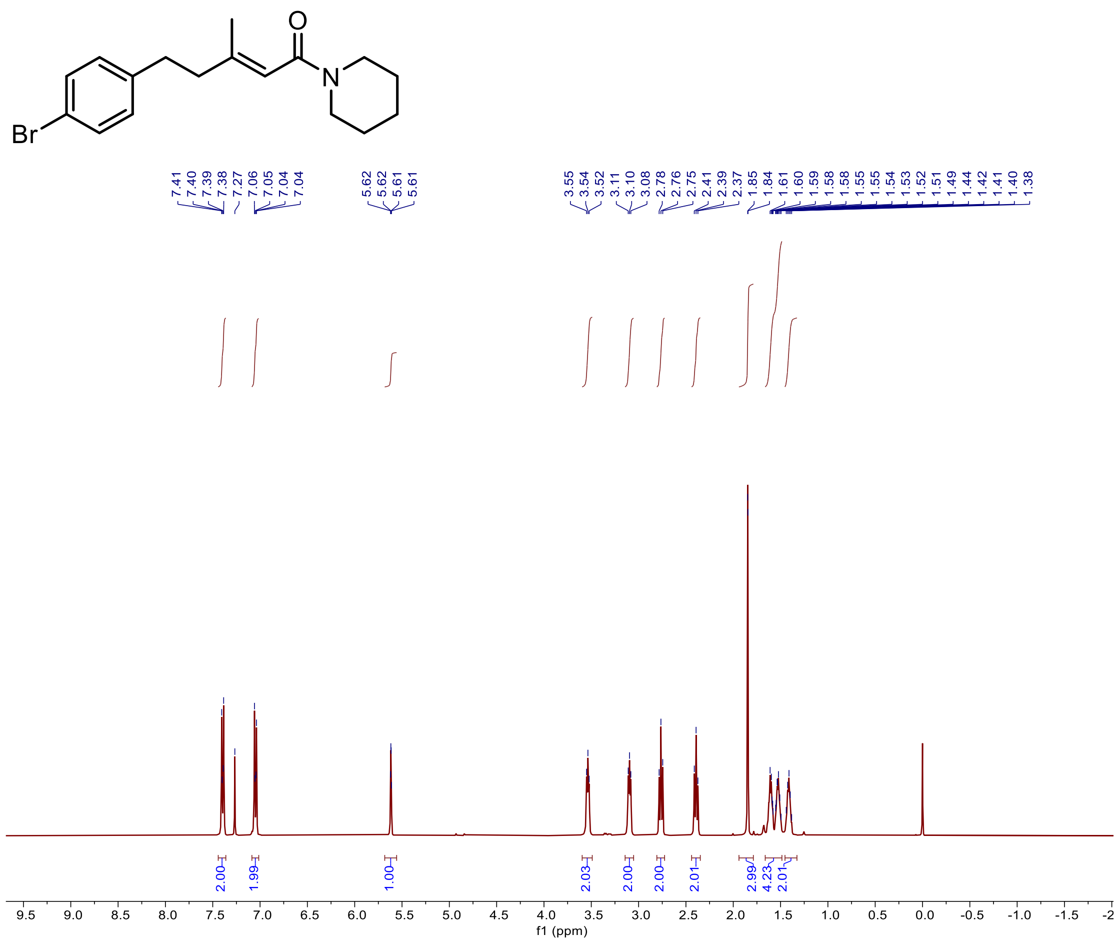

Supplementary Figure 34. ^{1}H NMR (400 MHz, $CDCl_3$) of **1o**.

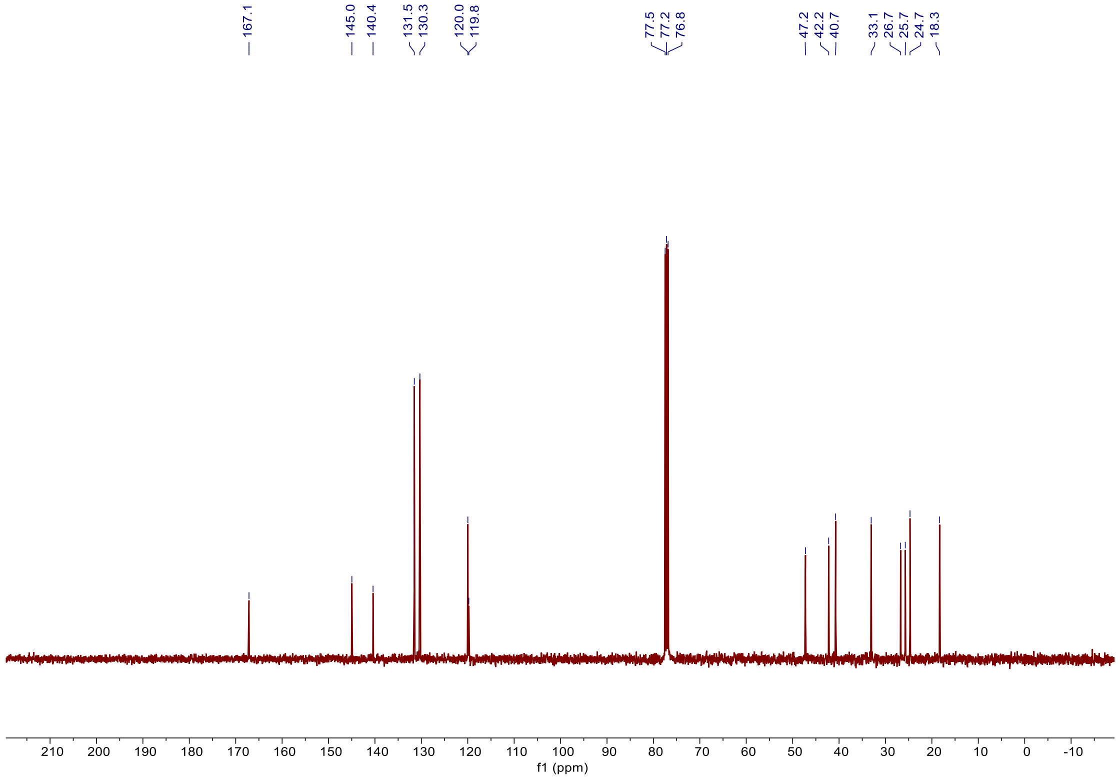

Supplementary Figure 35. ^{13}C NMR (101 MHz, $CDCl_3$) of **1o**.

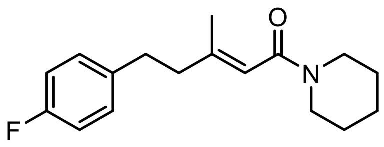

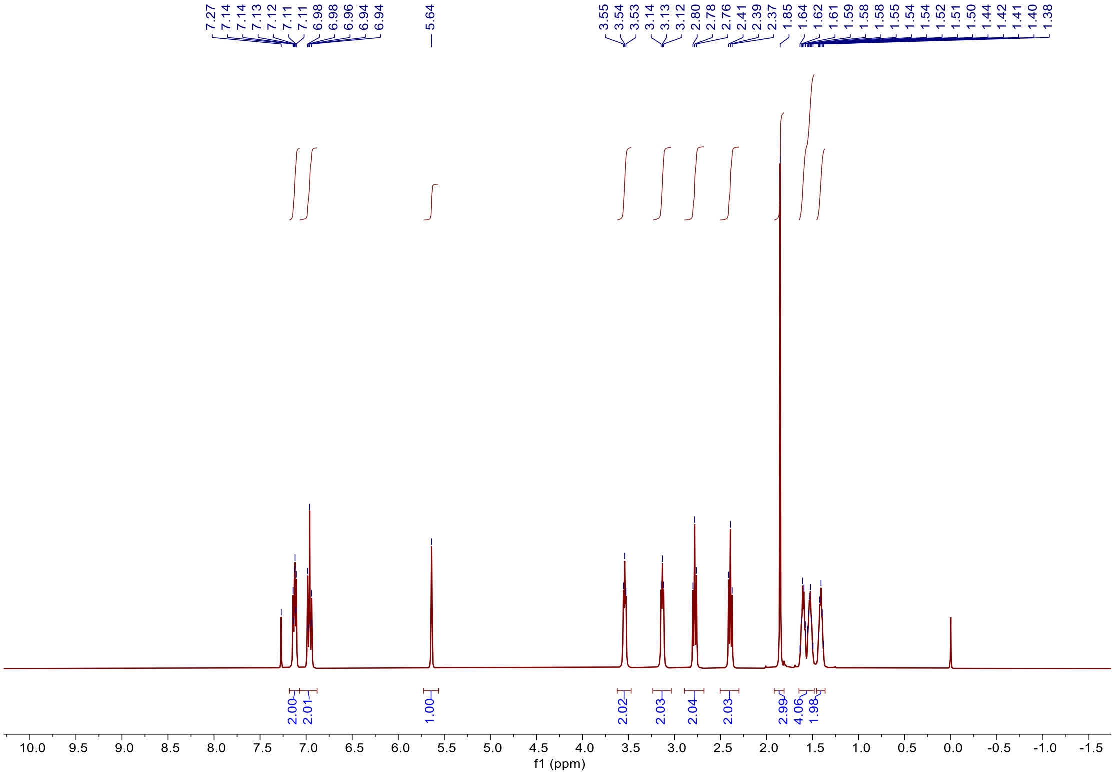

Supplementary Figure 36. ^{1}H NMR (400 MHz, $CDCl_3$) of **1p**.

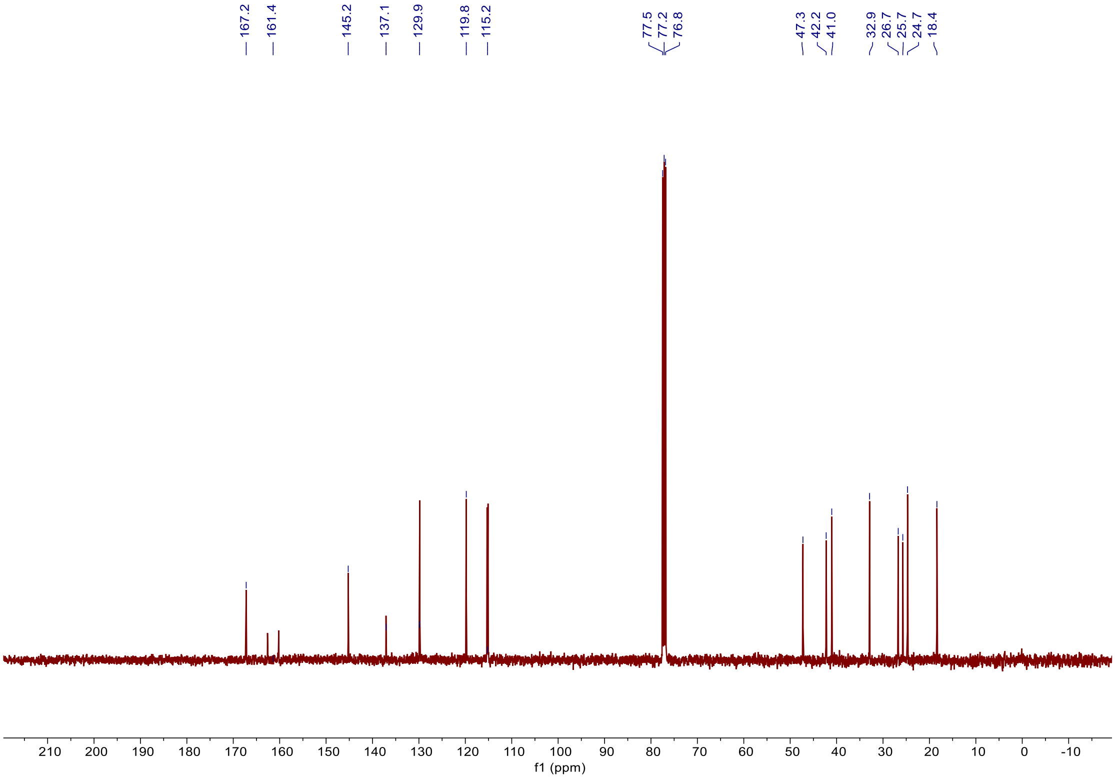

Supplementary Figure 37. ^{13}C NMR (101 MHz, $CDCl_3$) of **1p**.

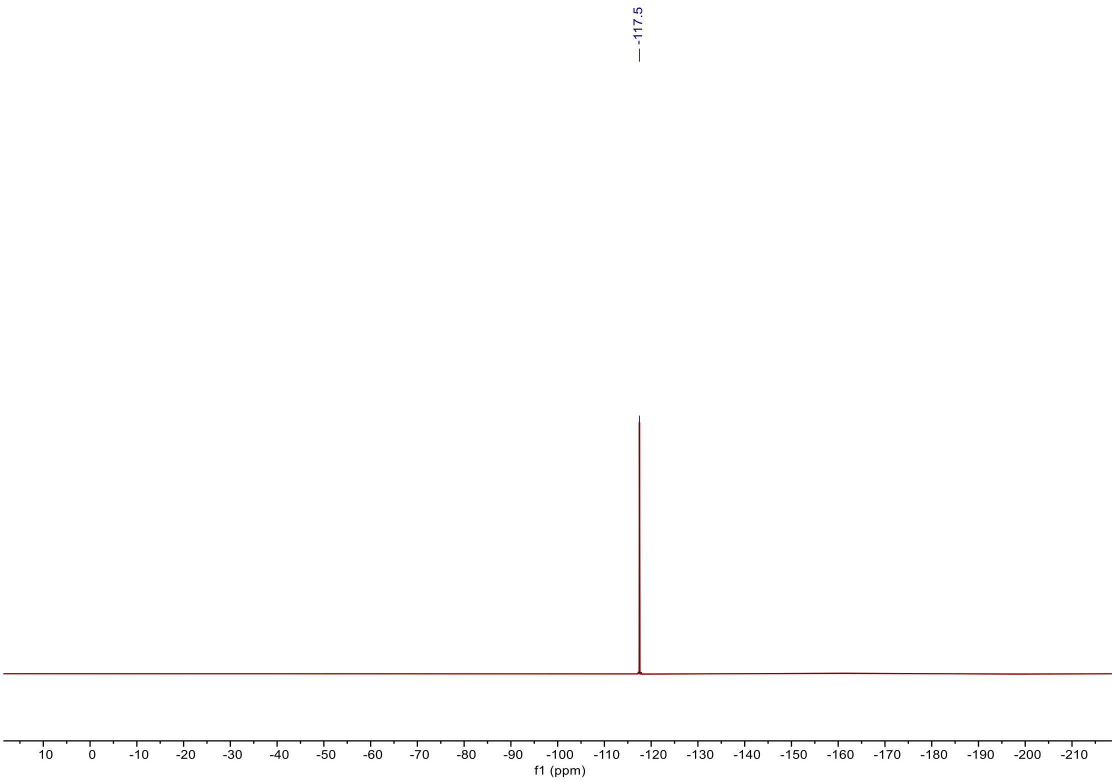

Supplementary Figure 38. **^{19}F NMR** (376 MHz, CDCl3) of **1p.**

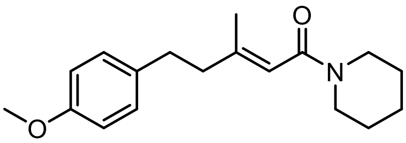

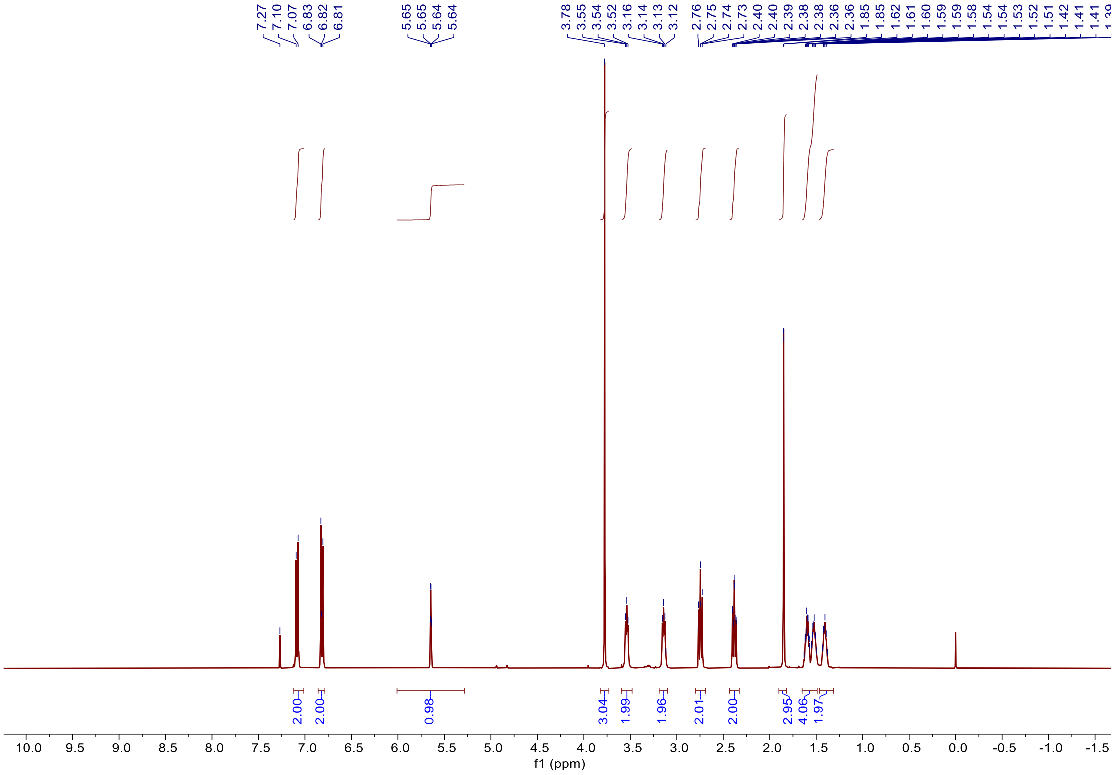

Supplementary Figure 39. ^{1}H NMR (400 MHz, $CDCl_3$) of **1q**.

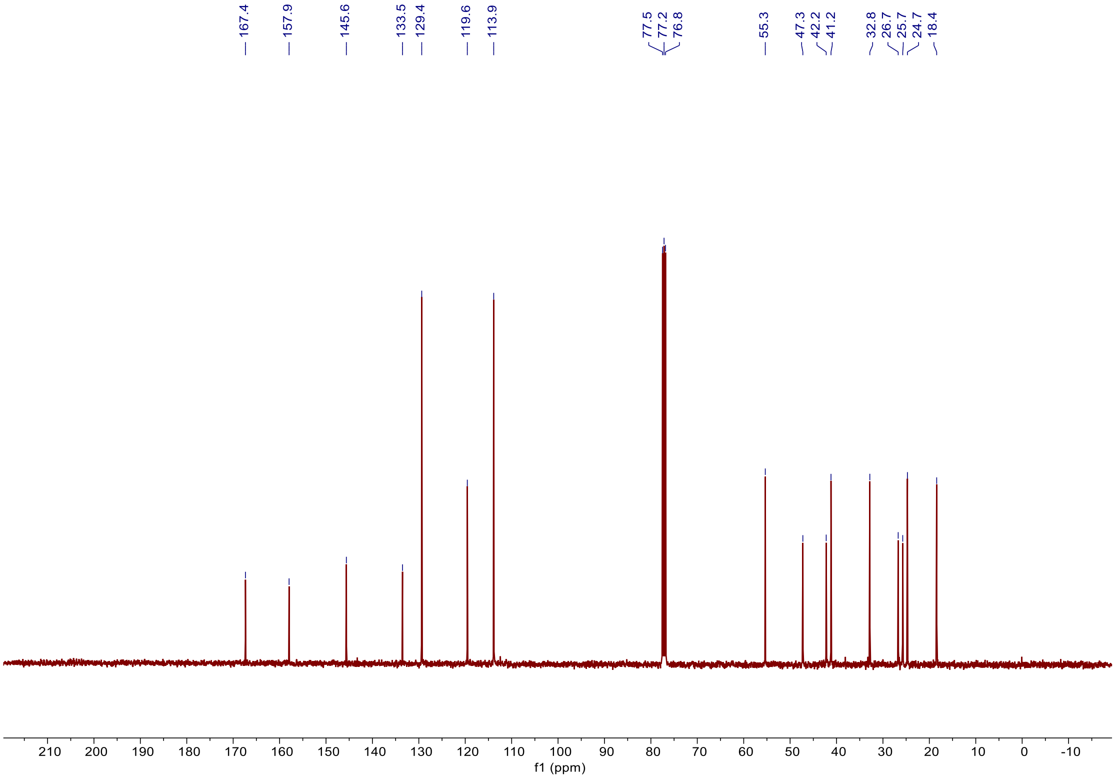

Supplementary Figure 40. ^{13}C NMR (101 MHz, $CDCl_3$) of **1q**.

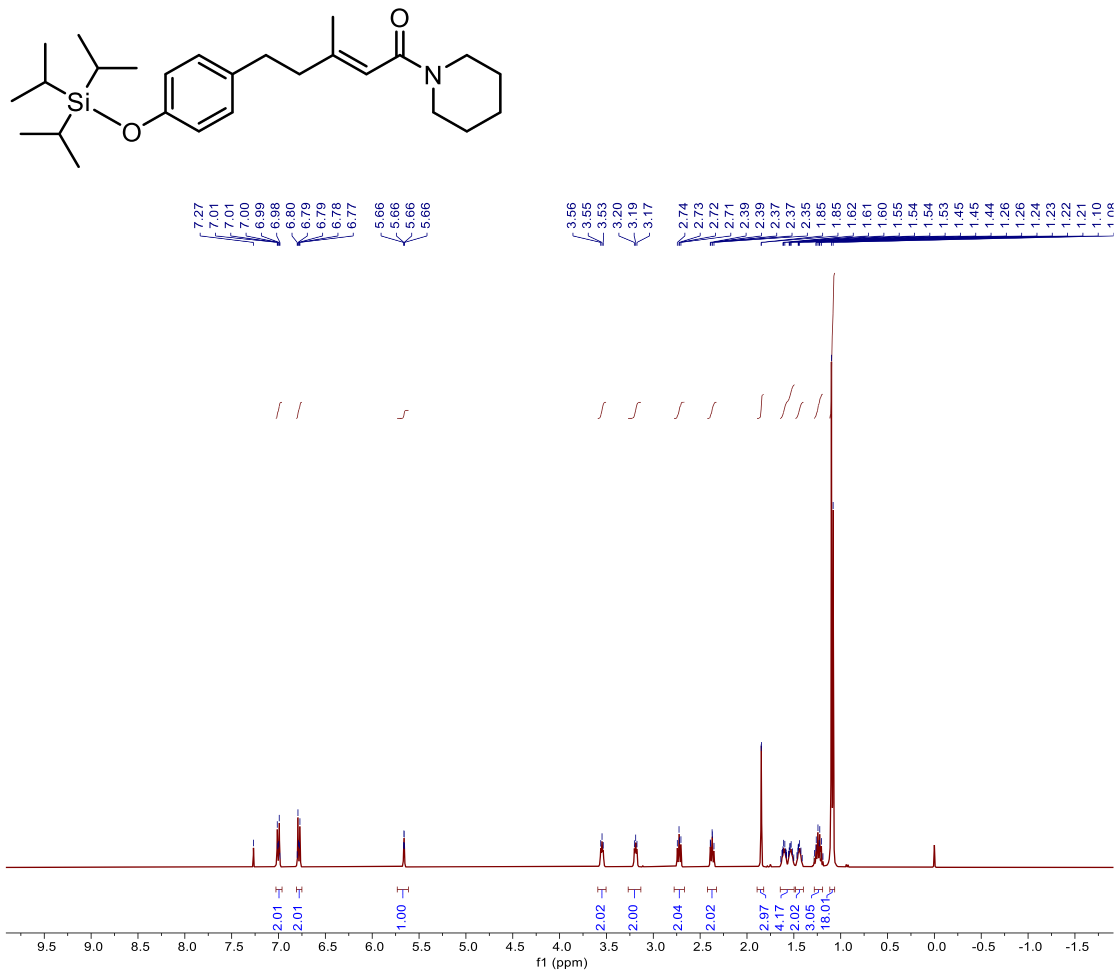

Supplementary Figure 41. ^{1}H NMR (400 MHz, $CDCl_3$) of **1r**.

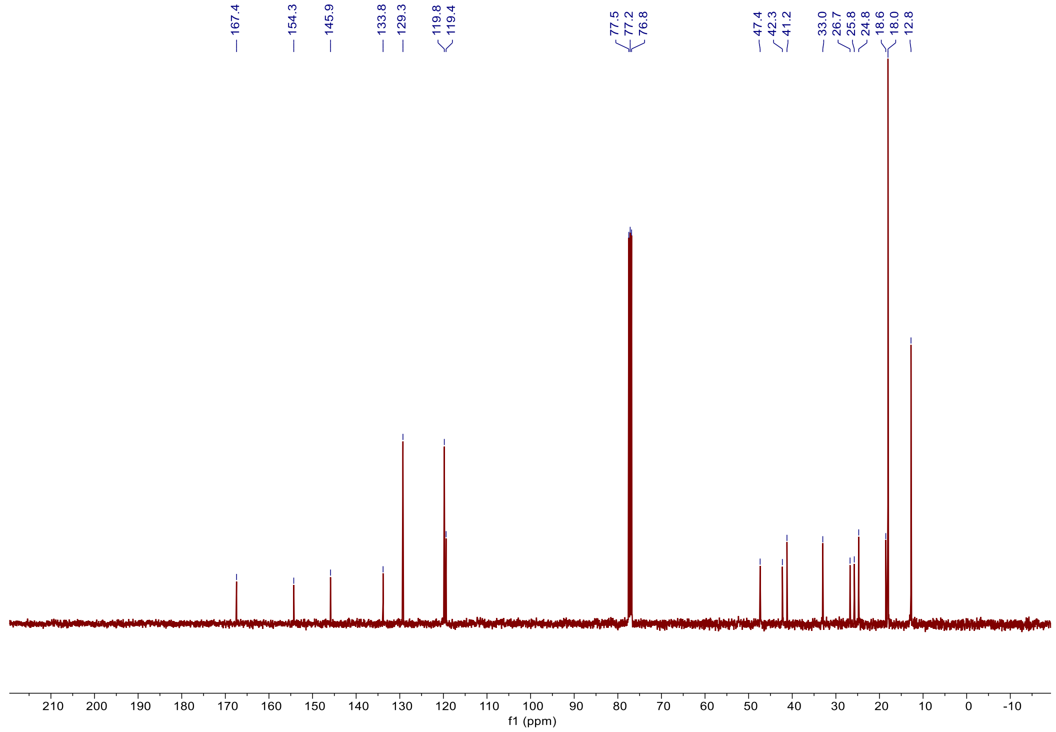

Supplementary Figure 42. ^{13}C NMR (101 MHz, $CDCl_3$) of **1r**.

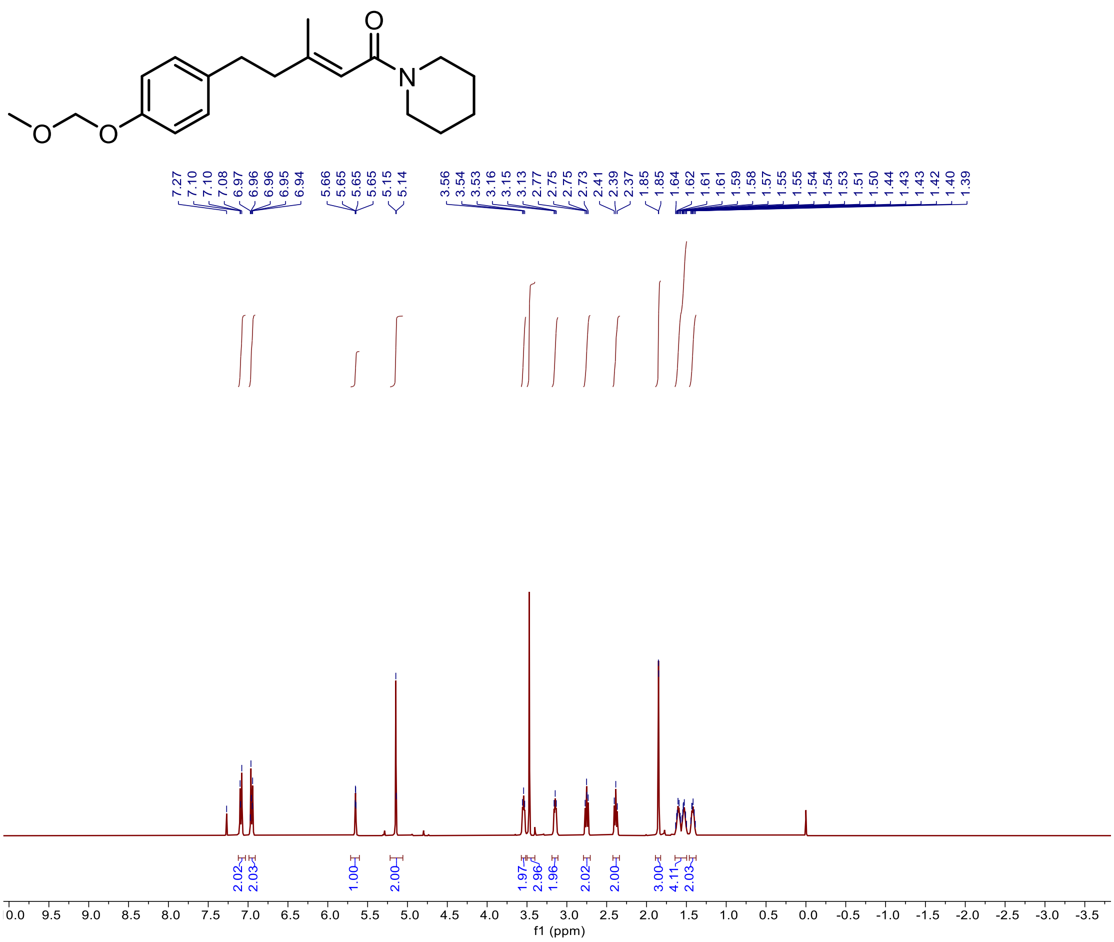

Supplementary Figure 43. ^{1}H NMR (400 MHz, $CDCl_3$) of **1s**.

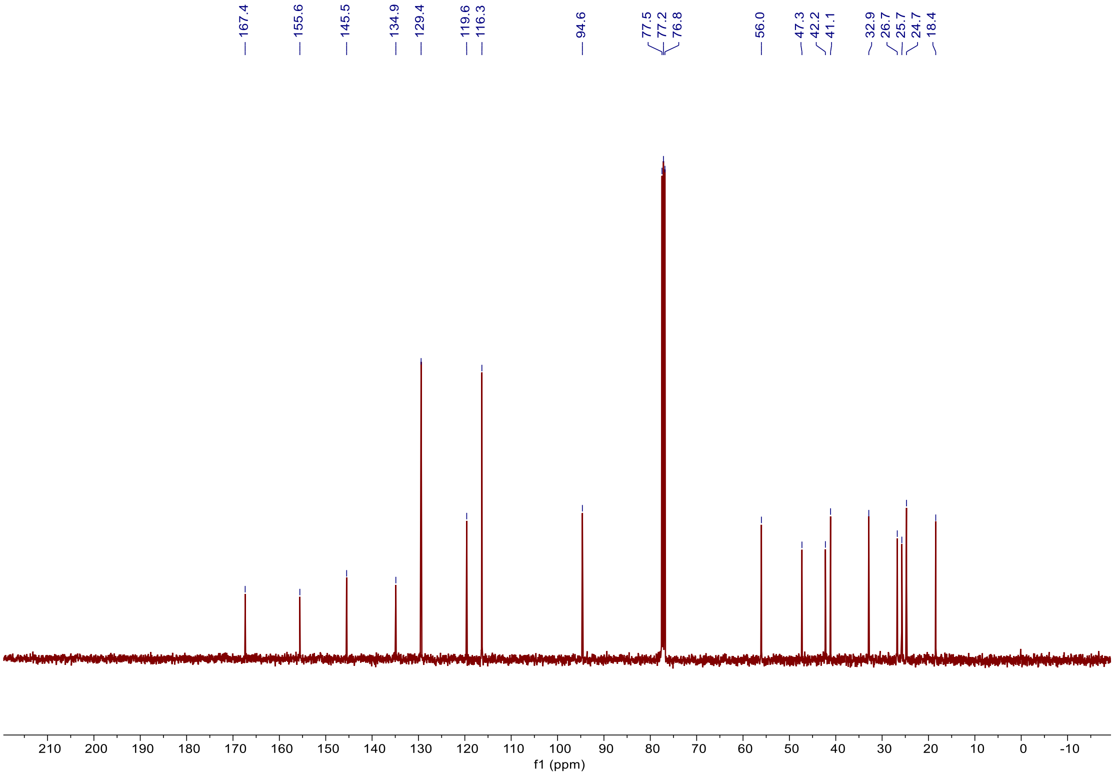

Supplementary Figure 44. ^{13}C NMR (101 MHz, $CDCl_3$) of **1s**.

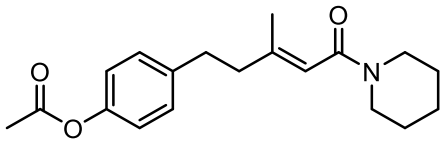

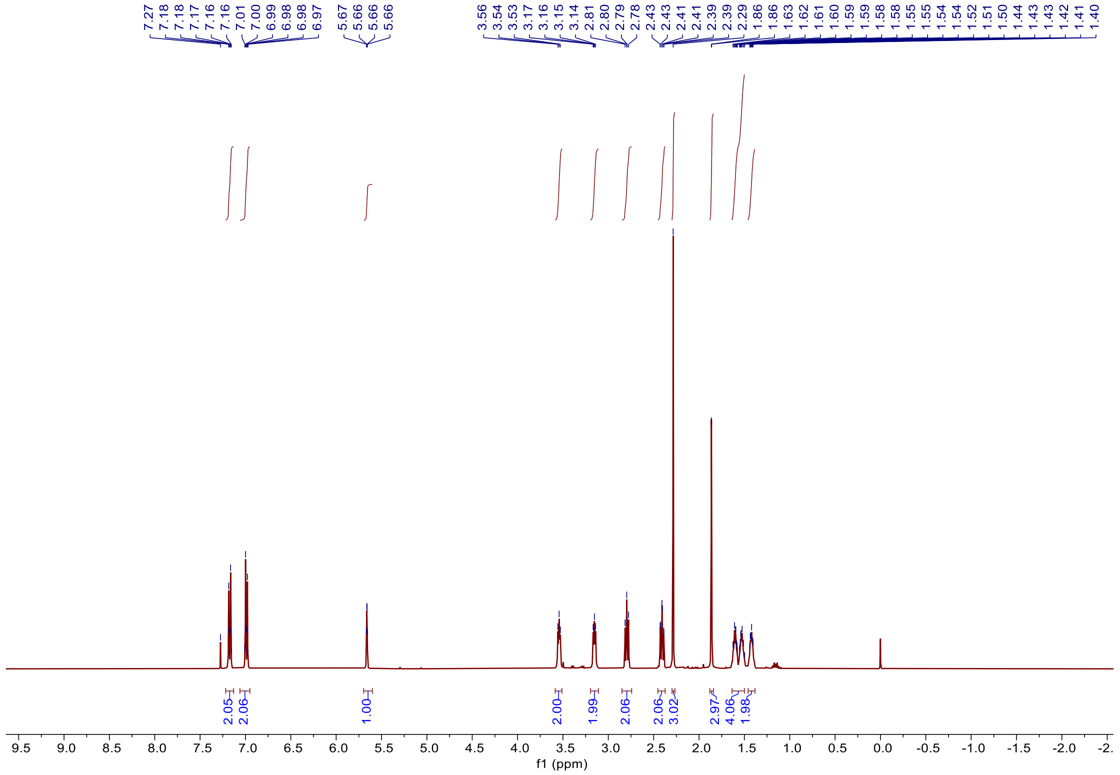

Supplementary Figure 45. ^{1}H NMR (400 MHz, $CDCl_3$) of **1t**.

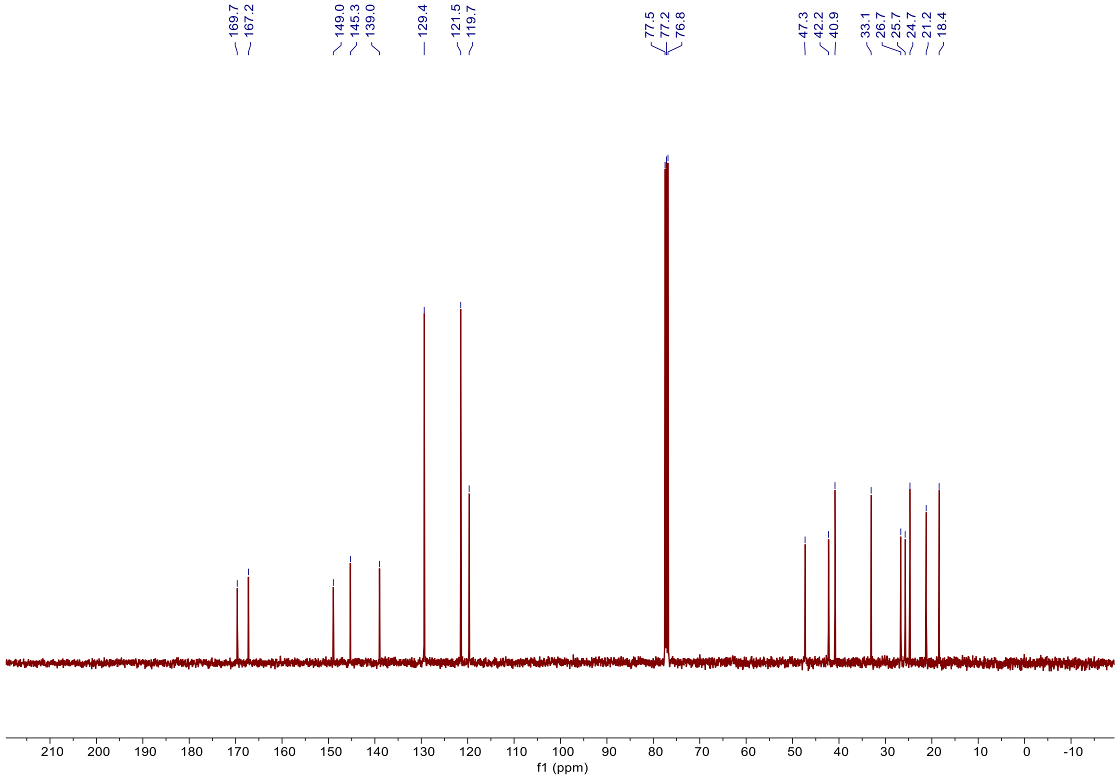

Supplementary Figure 46. ^{13}C NMR (101 MHz, $CDCl_3$) of **1t**.

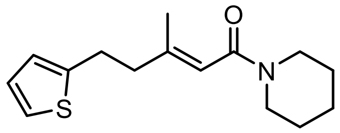

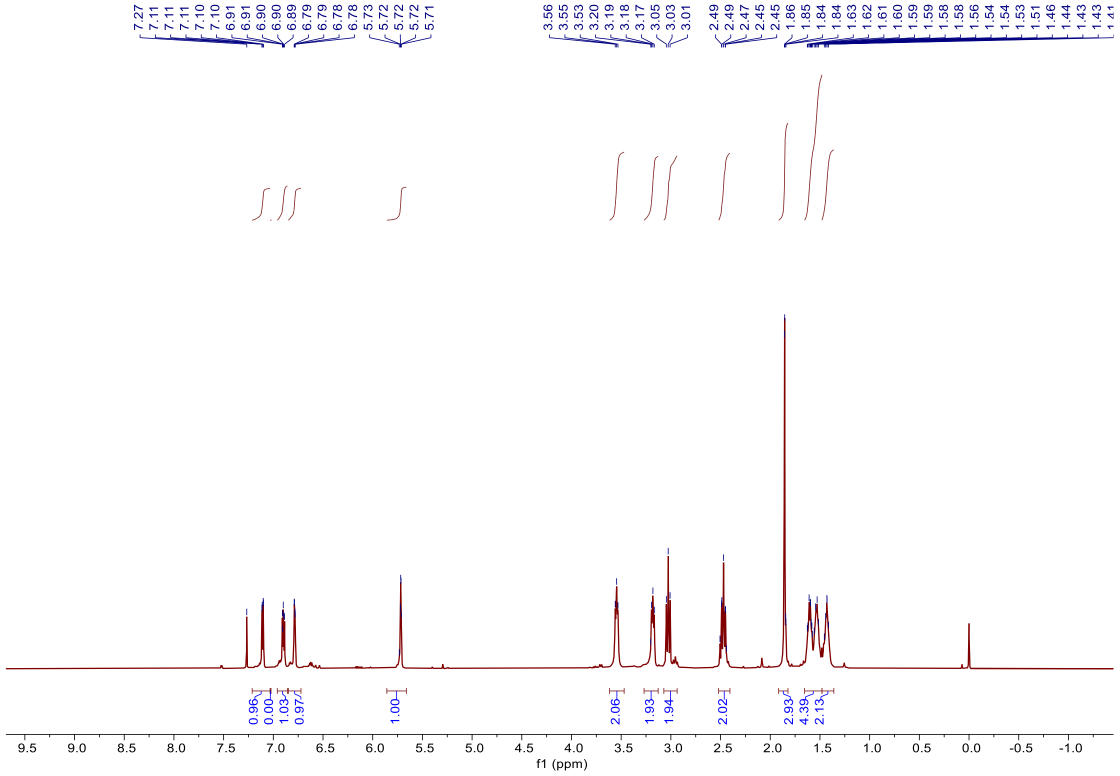

Supplementary Figure 47. ^{1}H NMR (400 MHz, $CDCl_3$) of **1u**.

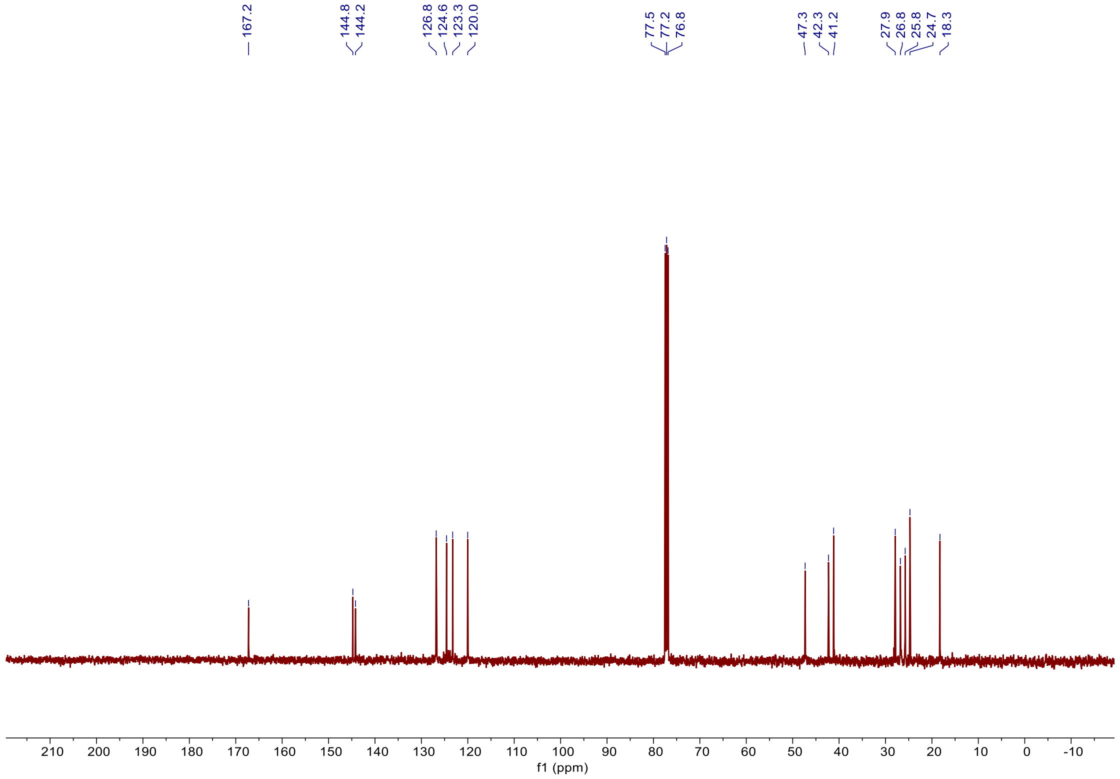

Supplementary Figure 48. ^{13}C NMR (101 MHz, $CDCl_3$) of **1u**.

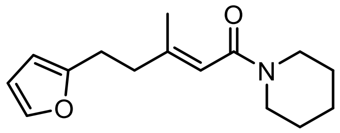

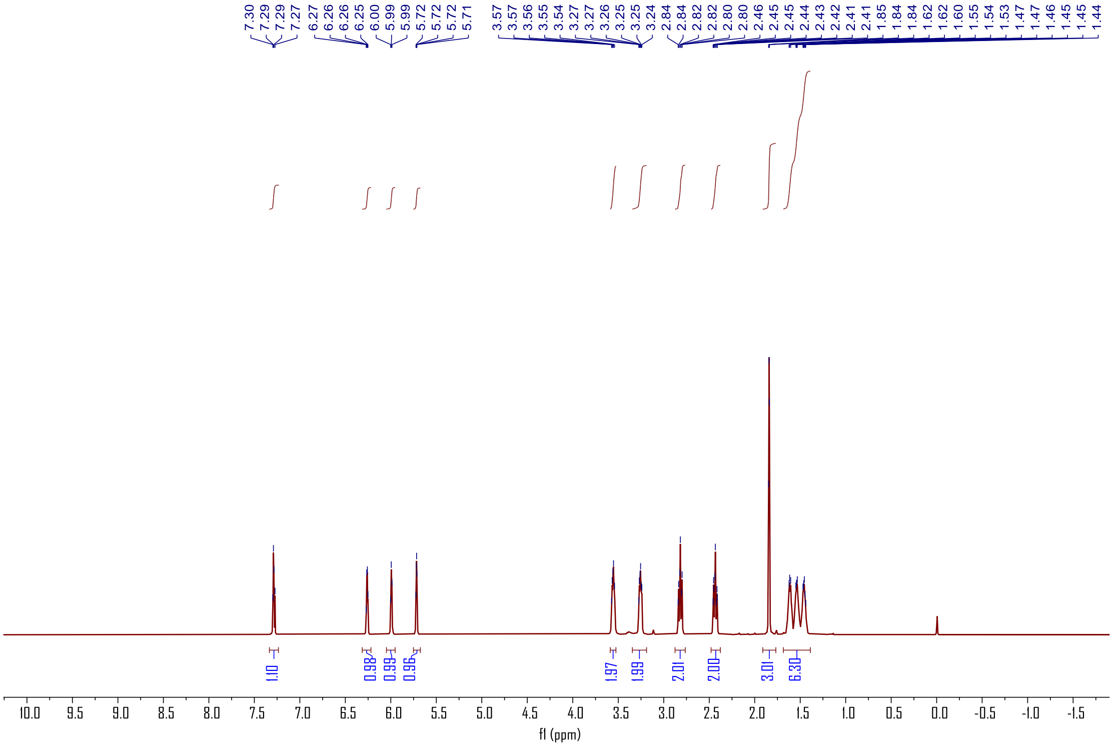

Supplementary Figure 49. ^{1}H NMR (400 MHz, $CDCl_3$) of **1v**.

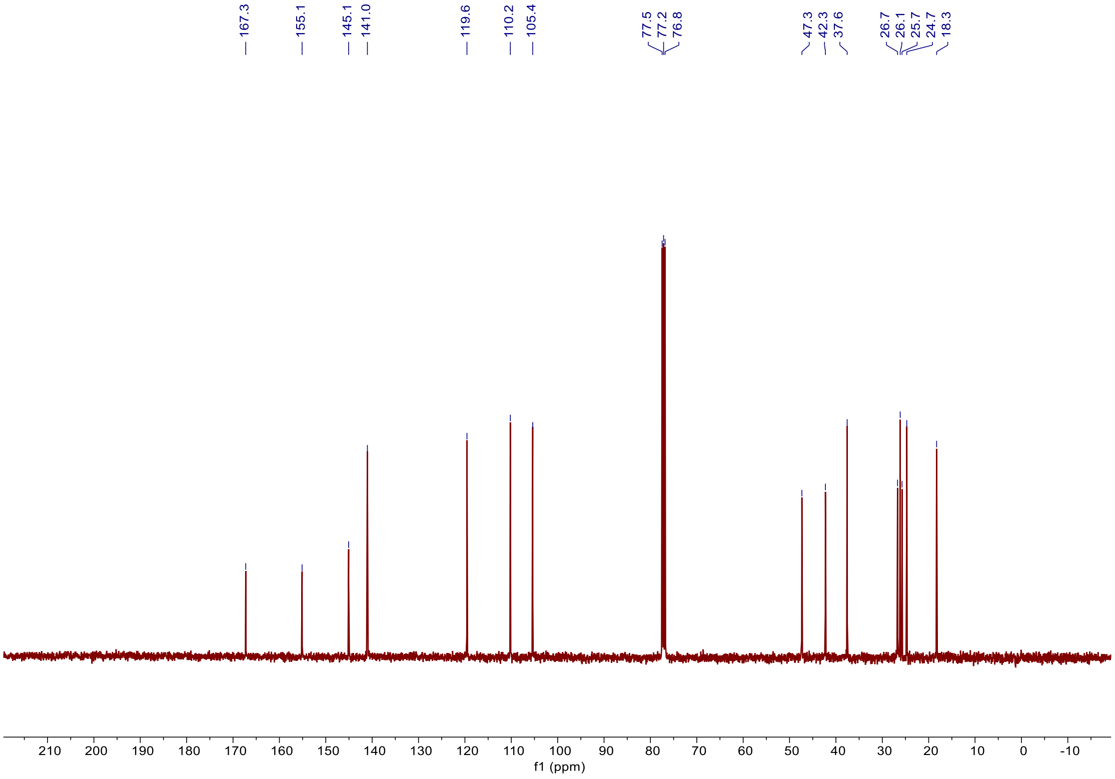

Supplementary Figure 50. ^{13}C NMR (101 MHz, $CDCl_3$) of **1v**.

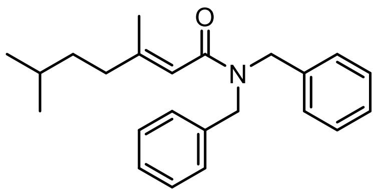

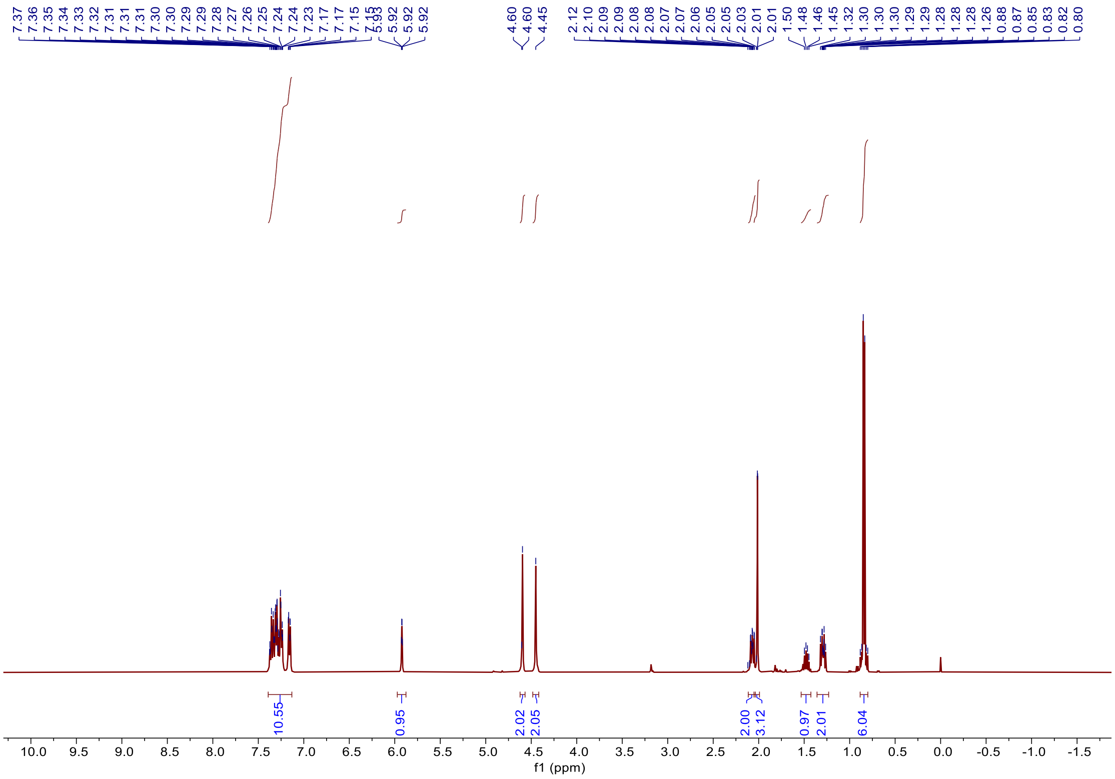

Supplementary Figure 51. ^{1}H NMR (400 MHz, $CDCl_3$) of **1w**.

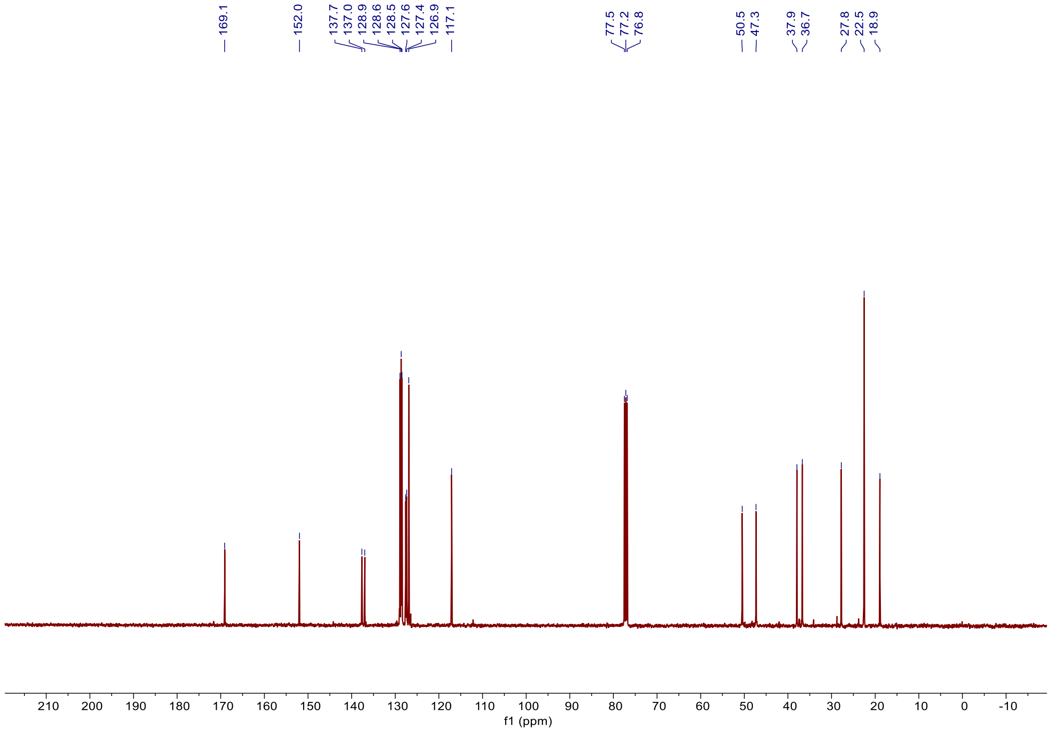

Supplementary Figure 52. ^{13}C NMR (101 MHz, $CDCl_3$) of **1w**.

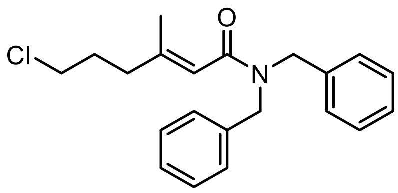

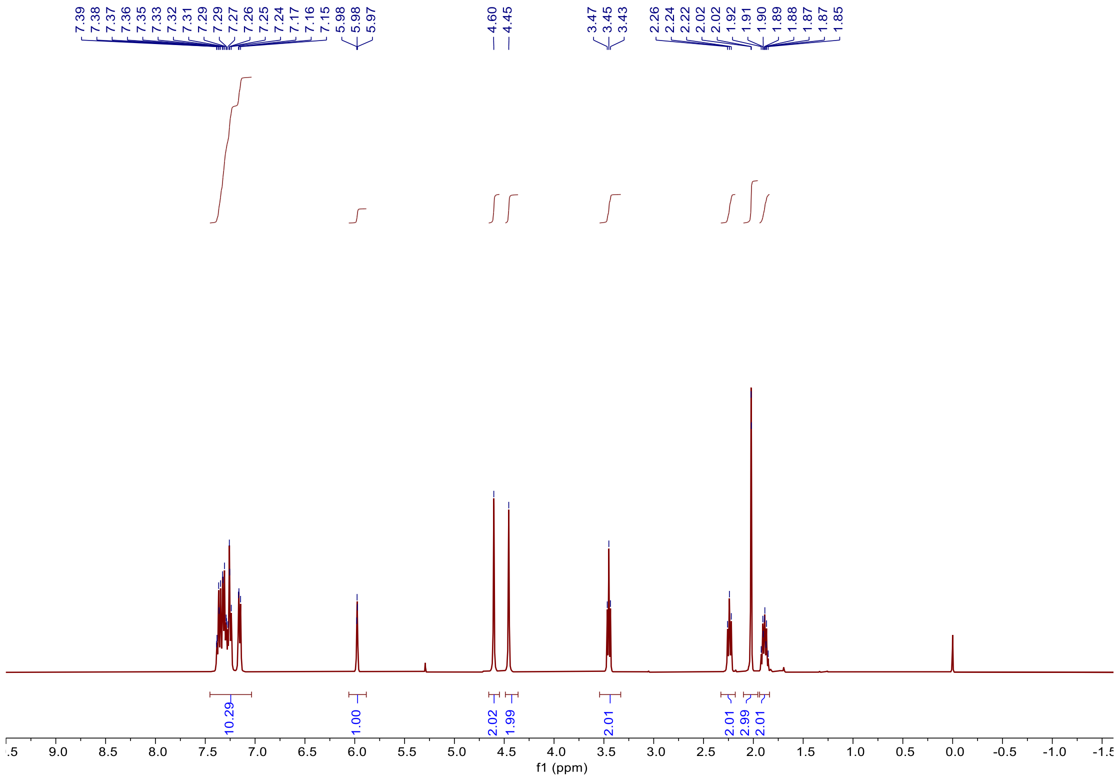

Supplementary Figure 53. ^{1}H NMR (400 MHz, $CDCl_3$) of **1x**.

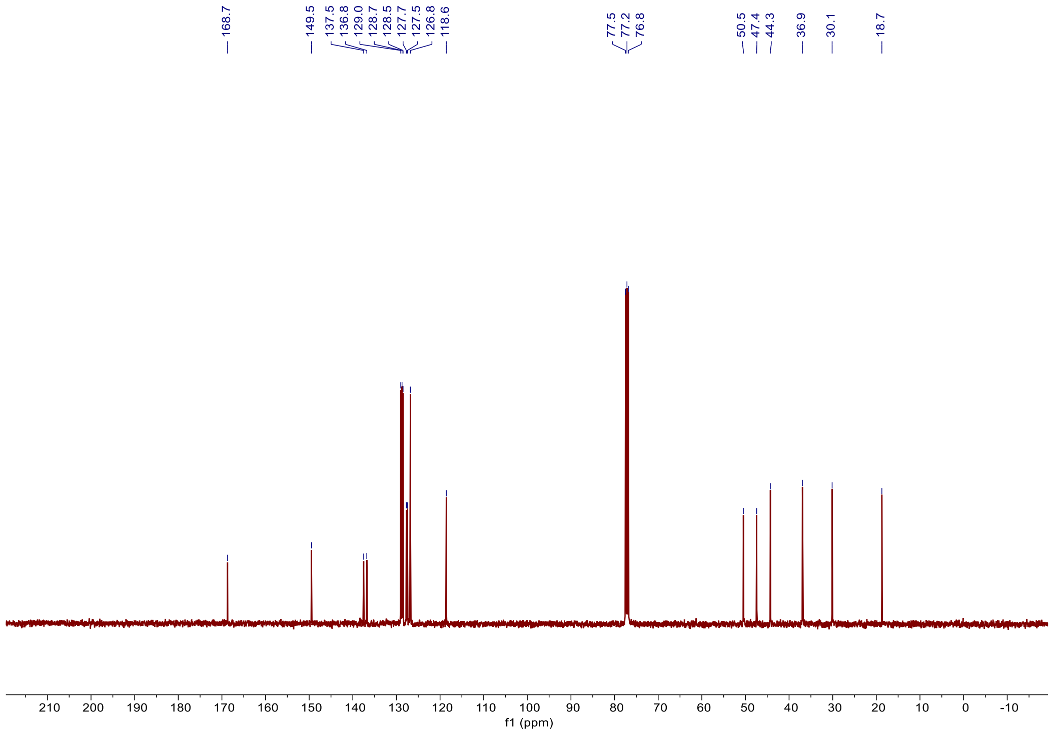

Supplementary Figure 54. ^{13}C NMR (101 MHz, $CDCl_3$) of **1x**.

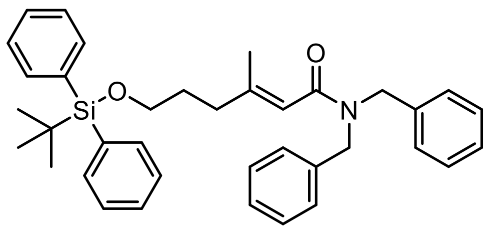

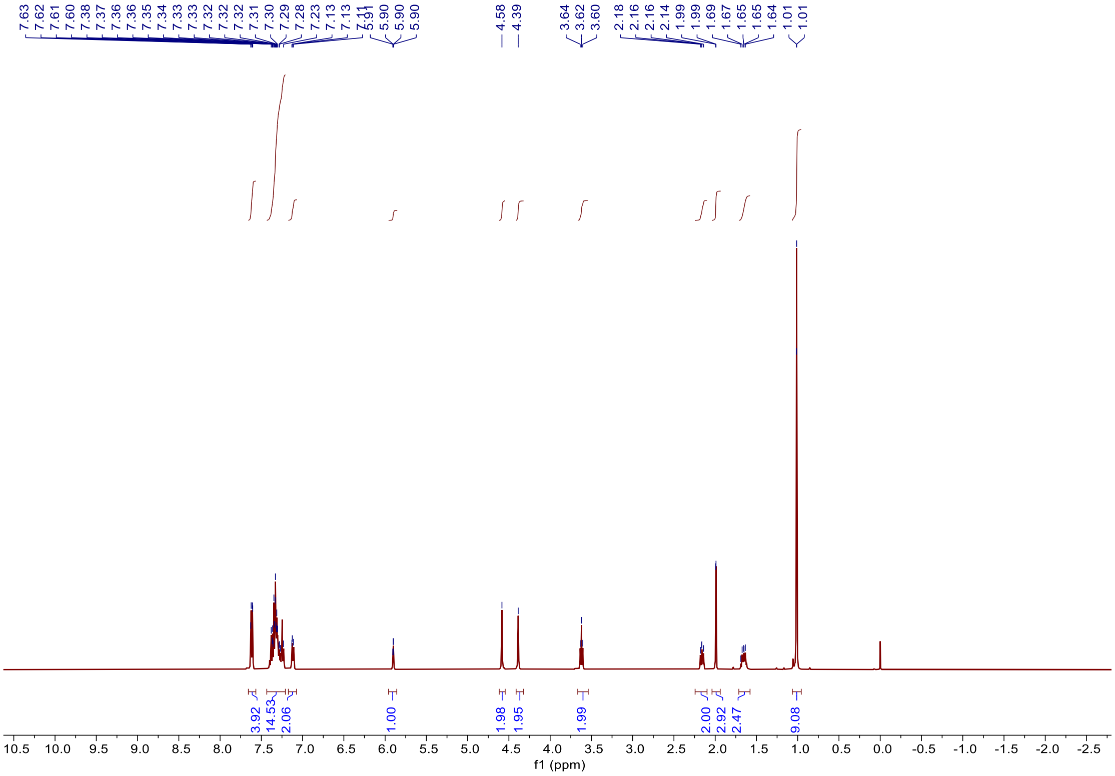

Supplementary Figure 55. ^{1}H NMR (400 MHz, $CDCl_3$) of **1y**.

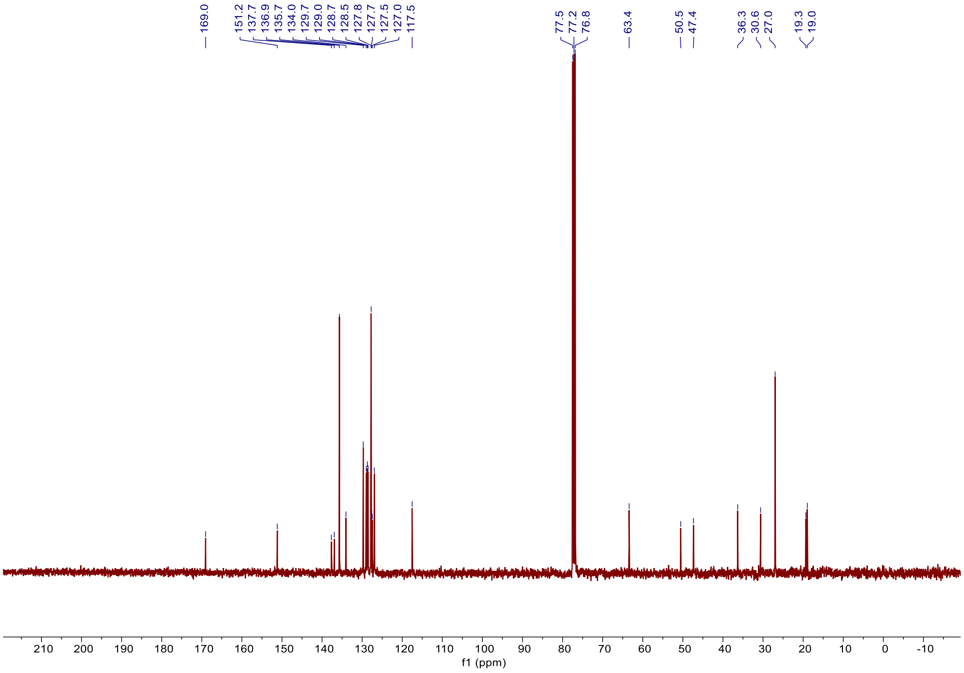

Supplementary Figure 56. ^{13}C NMR (101 MHz, $CDCl_3$) of **1y**.

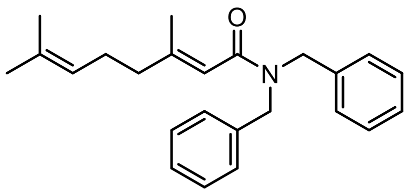

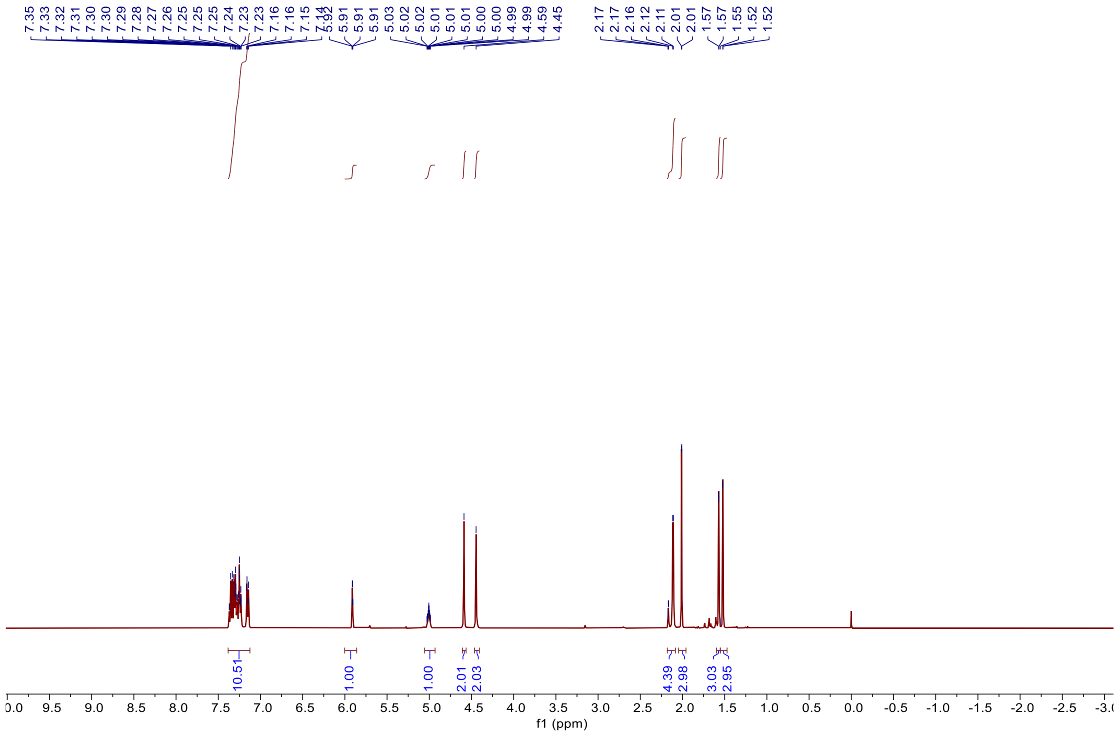

Supplementary Figure 57. ^{1}H NMR (400 MHz, $CDCl_3$) of **1z**.

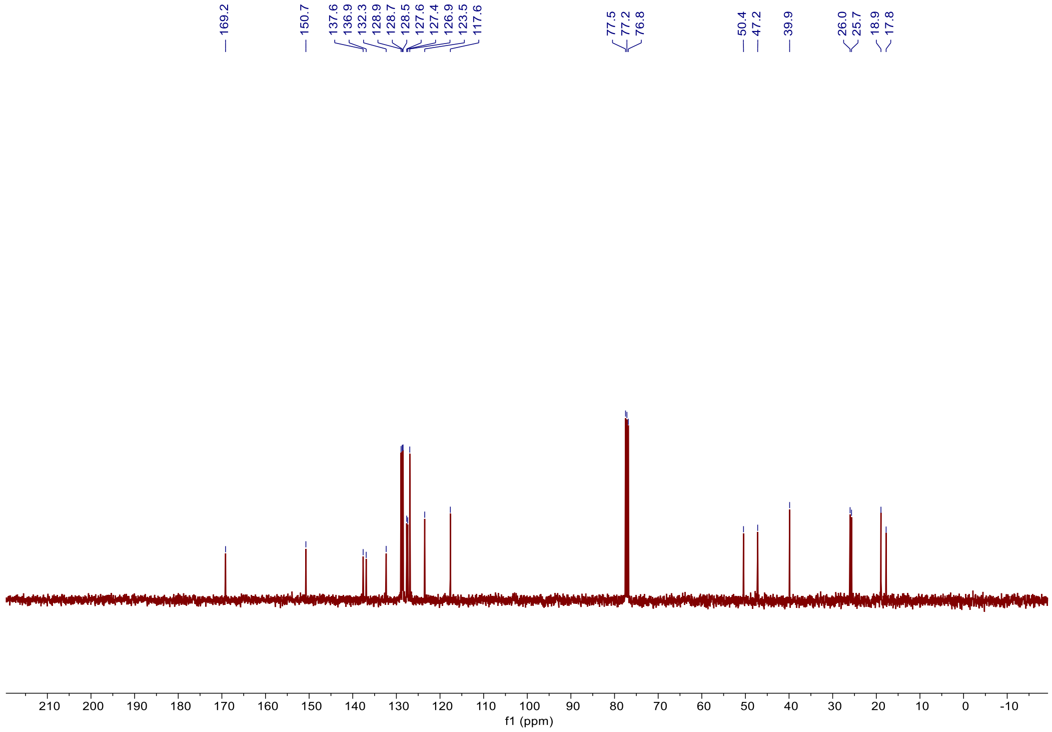

Supplementary Figure 58. ^{13}C NMR (101 MHz, $CDCl_3$) of **1z**.

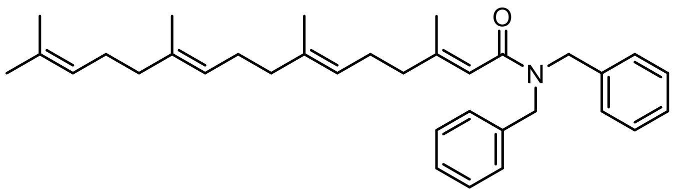

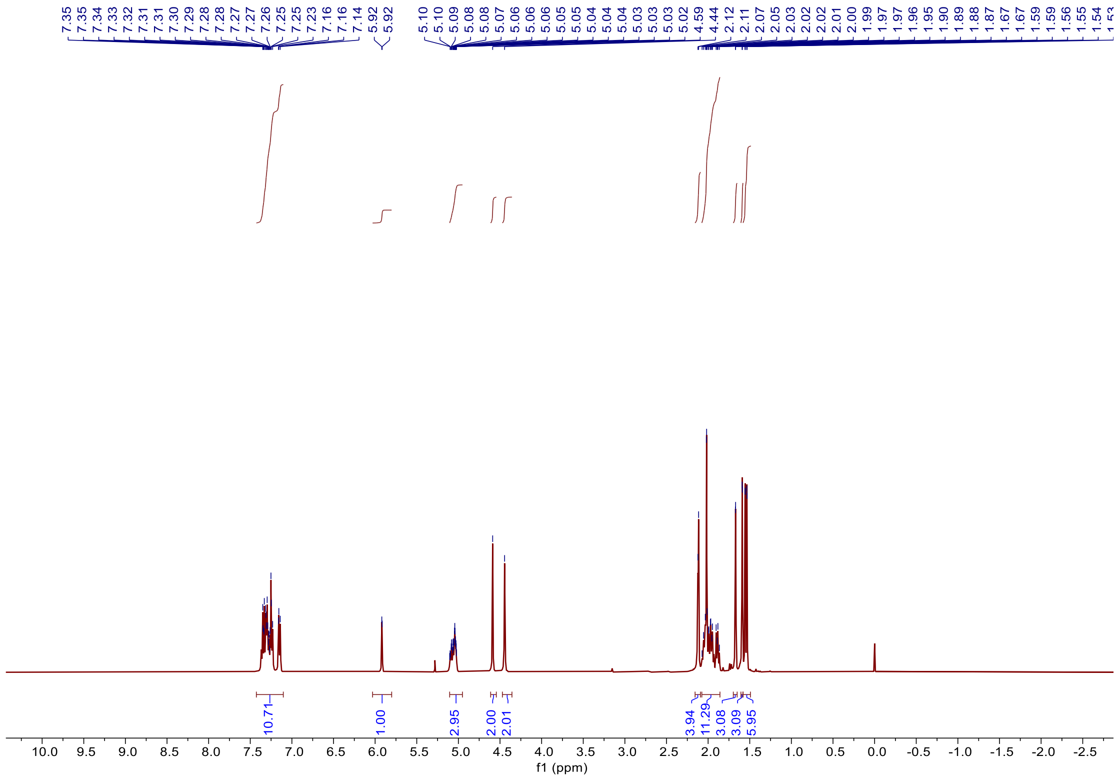

Supplementary Figure 59. ^{1}H NMR (400 MHz, CDCl$_3$) of **1aa**.

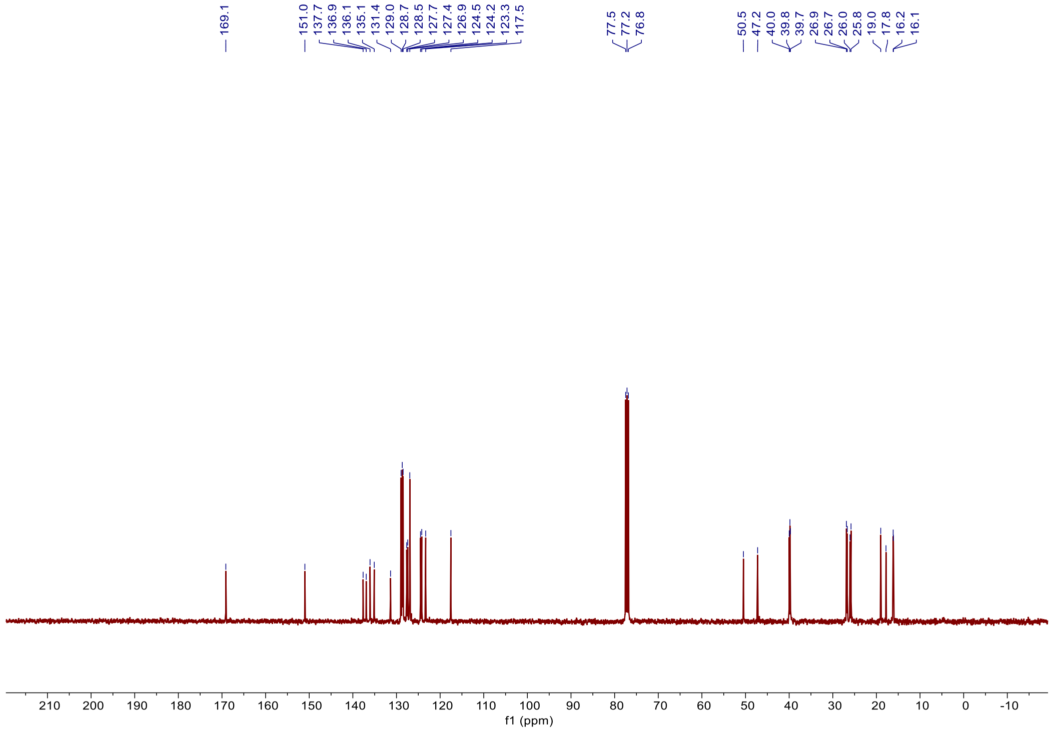

Supplementary Figure 60. ^{13}C NMR (101 MHz, CDCl$_3$) of **1aa**.

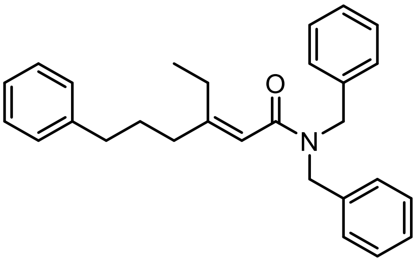

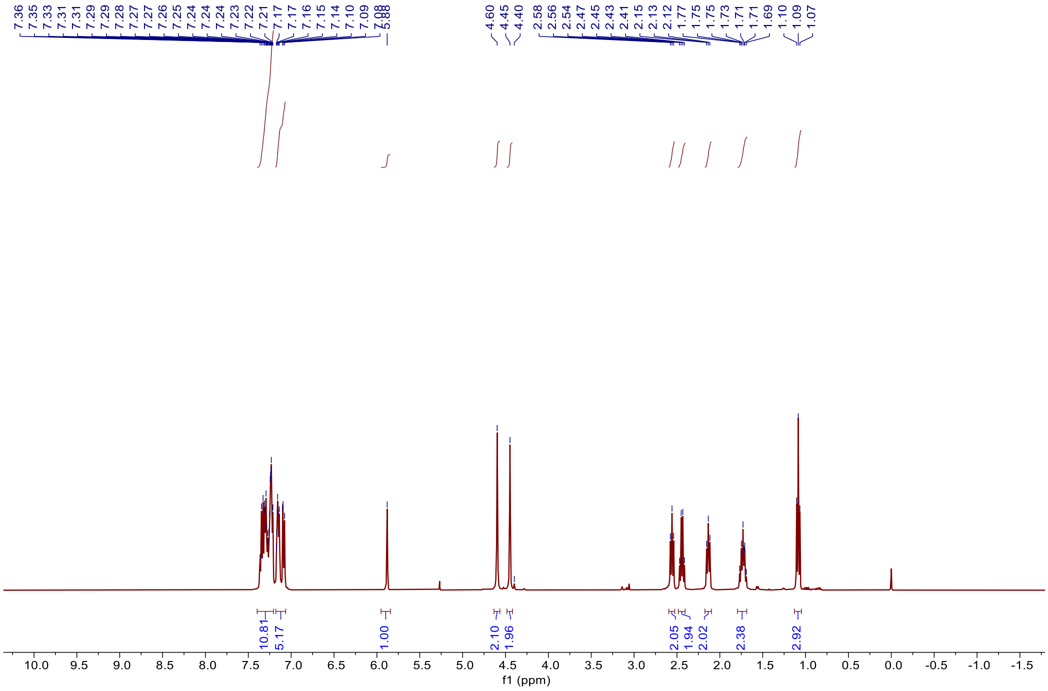

Supplementary Figure 61. ^{1}H NMR (400 MHz, $CDCl_3$) of **1ab**.

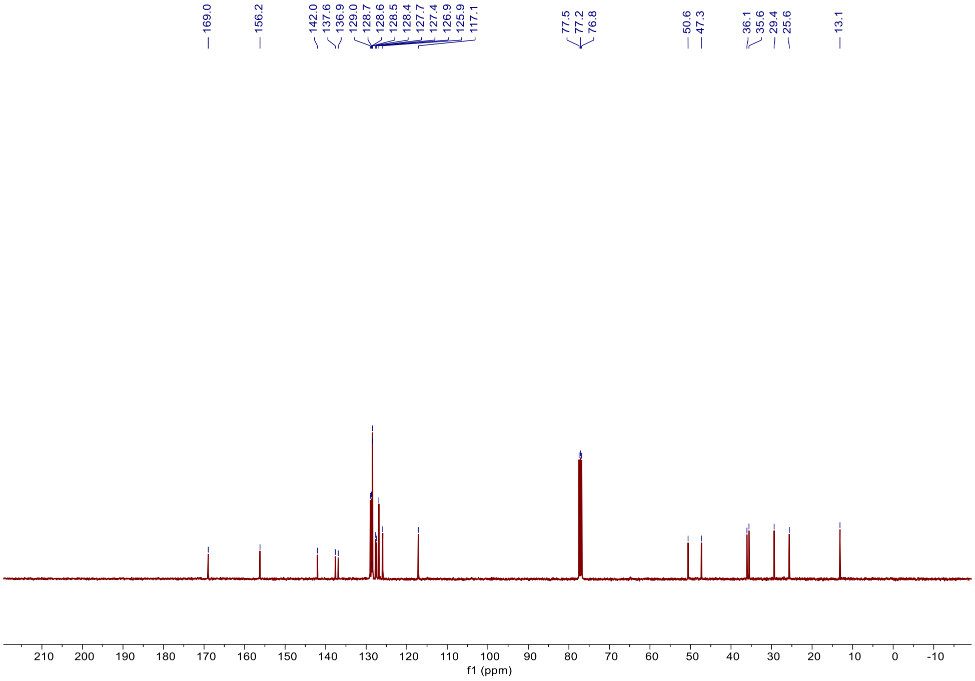

Supplementary Figure 62. ^{13}C NMR (101 MHz, $CDCl_3$) of **1ab**.

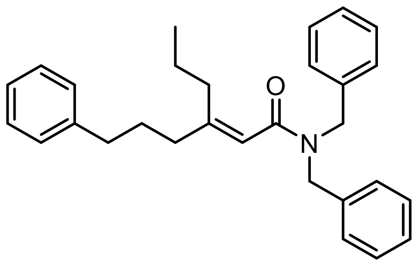

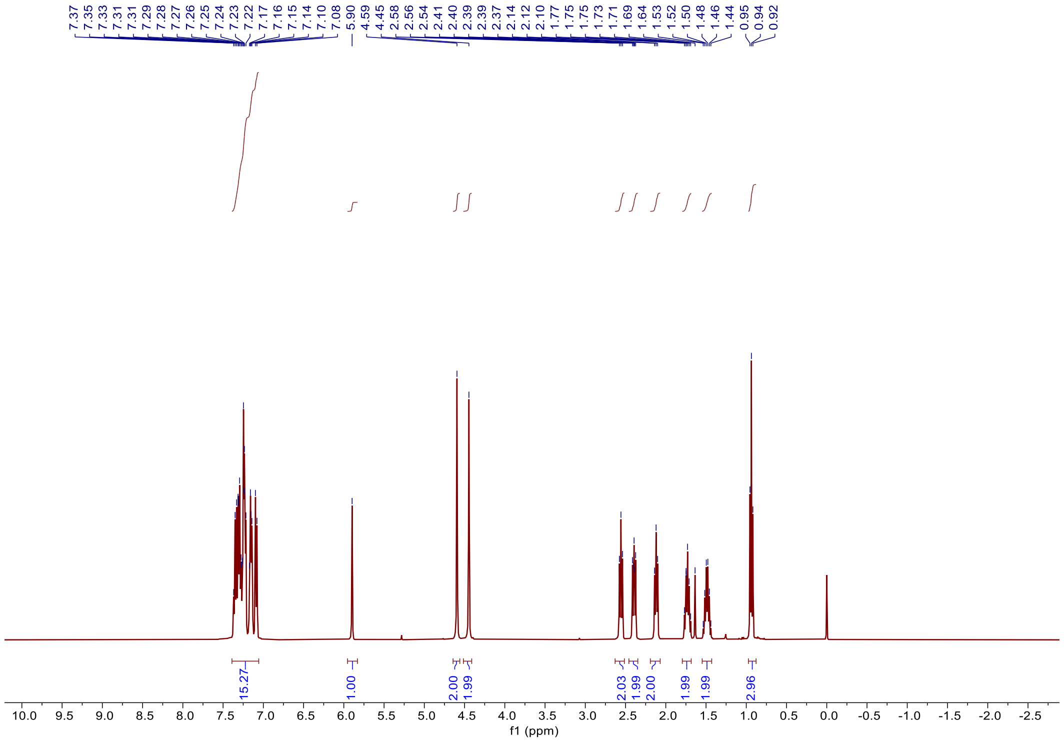

Supplementary Figure 63. ^{1}H NMR (400 MHz, $CDCl_3$) of **1ac**.

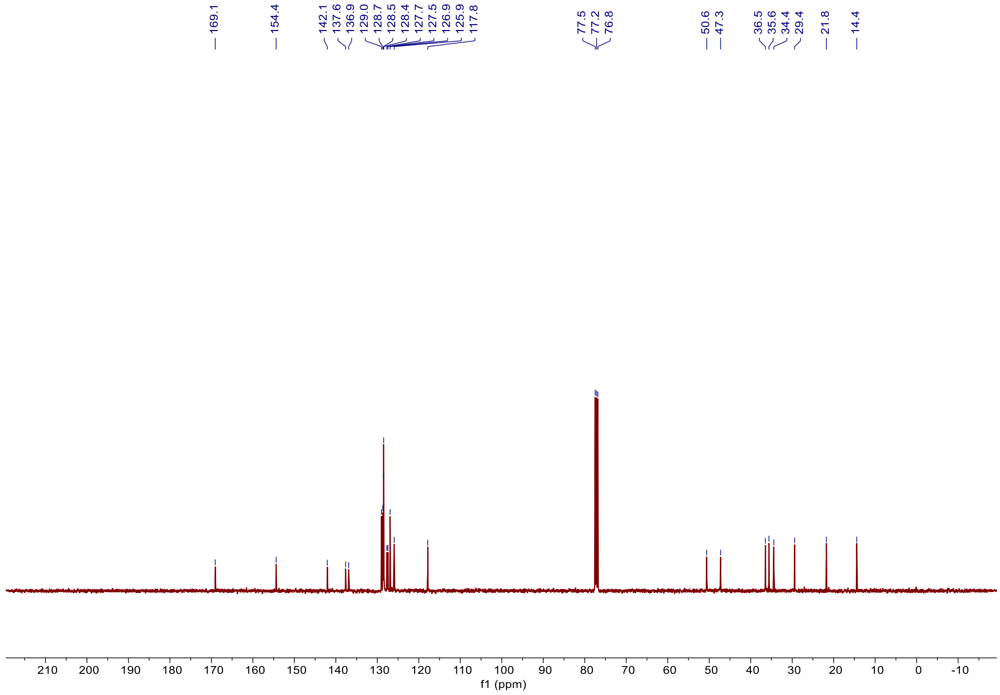

Supplementary Figure 64. ^{13}C NMR (101 MHz, $CDCl_3$) of **1ac**.

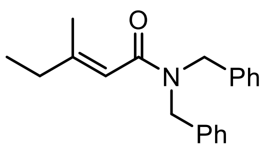

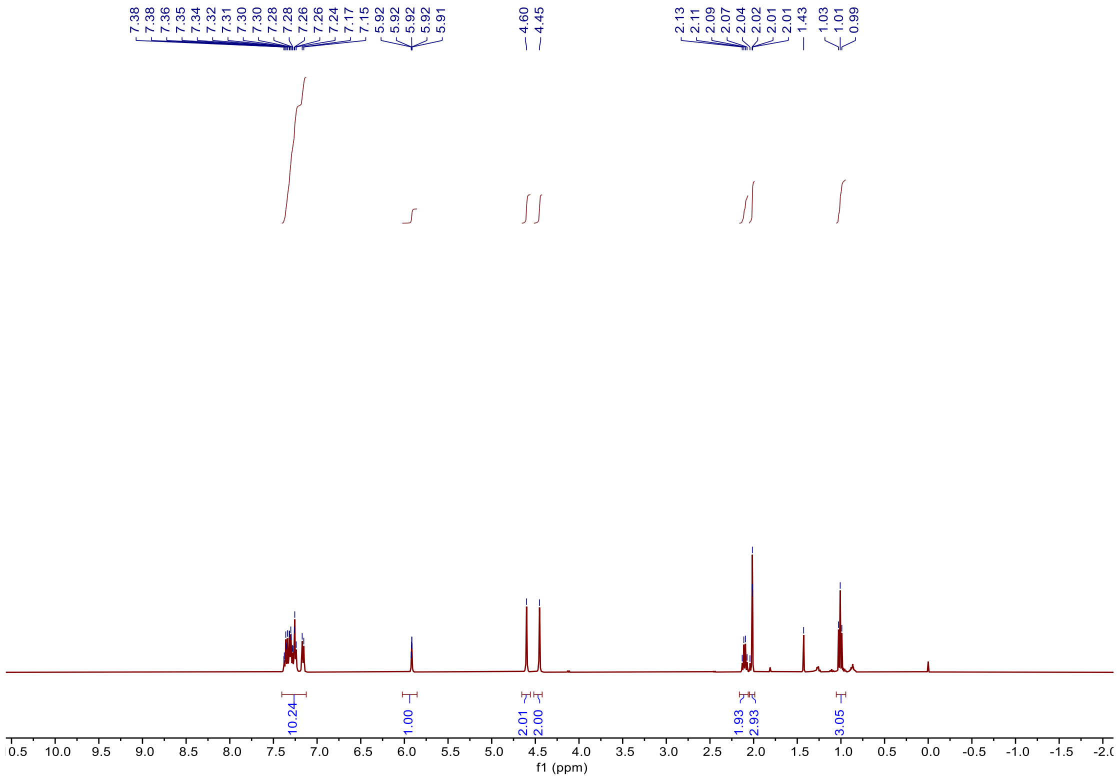

Supplementary Figure 65. ^{1}H NMR (400 MHz, $CDCl_3$) of **1ad**.

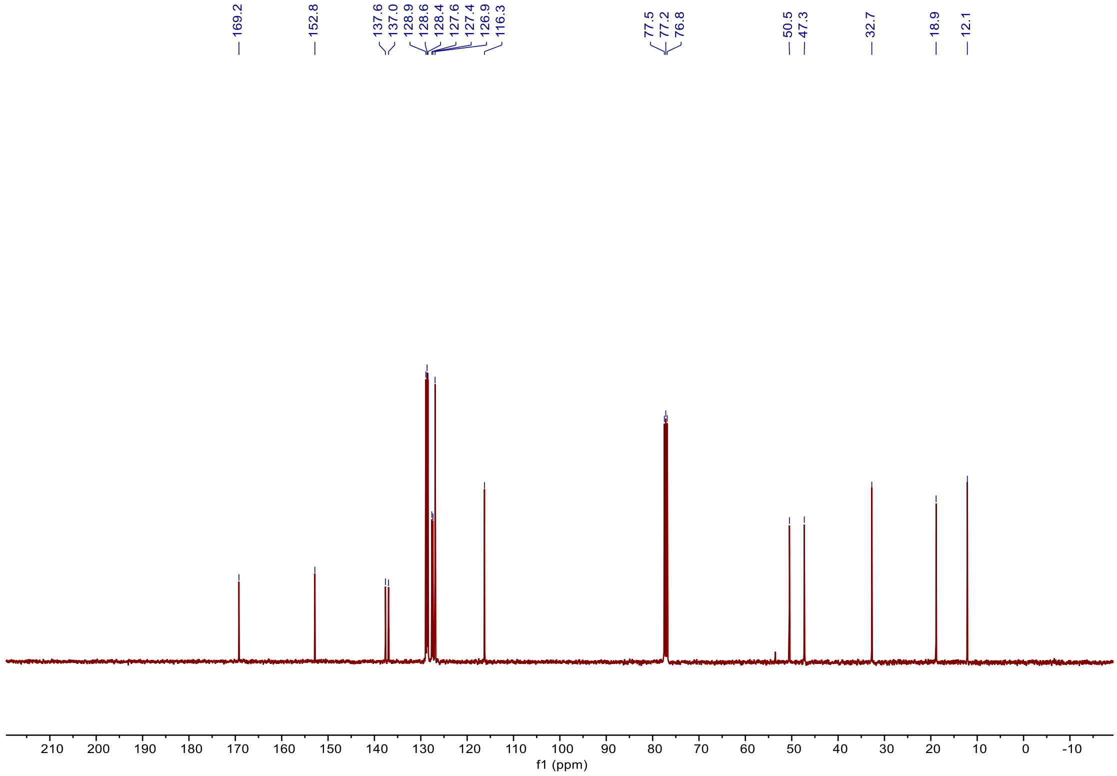

Supplementary Figure 66. ^{13}C NMR (101 MHz, $CDCl_3$) of **1ad**.

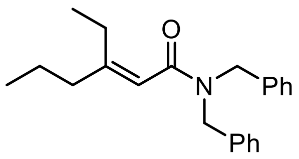

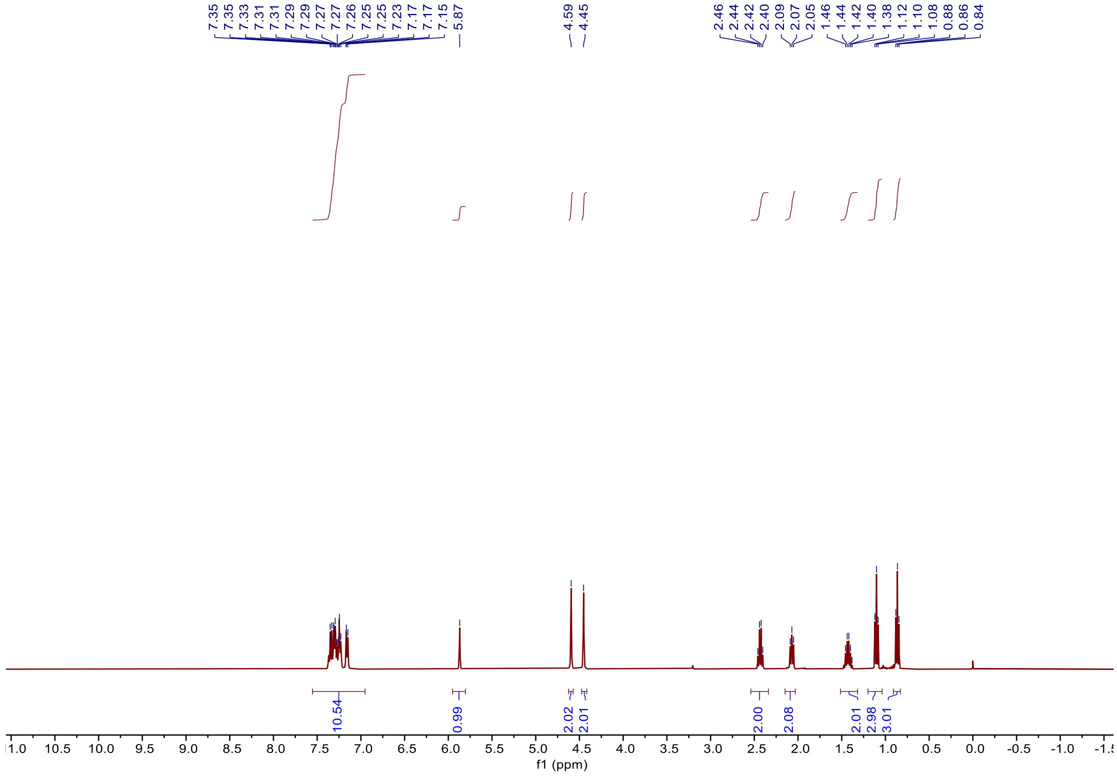

Supplementary Figure 67. ^{1}H NMR (400 MHz, $CDCl_3$) of **1ae**.

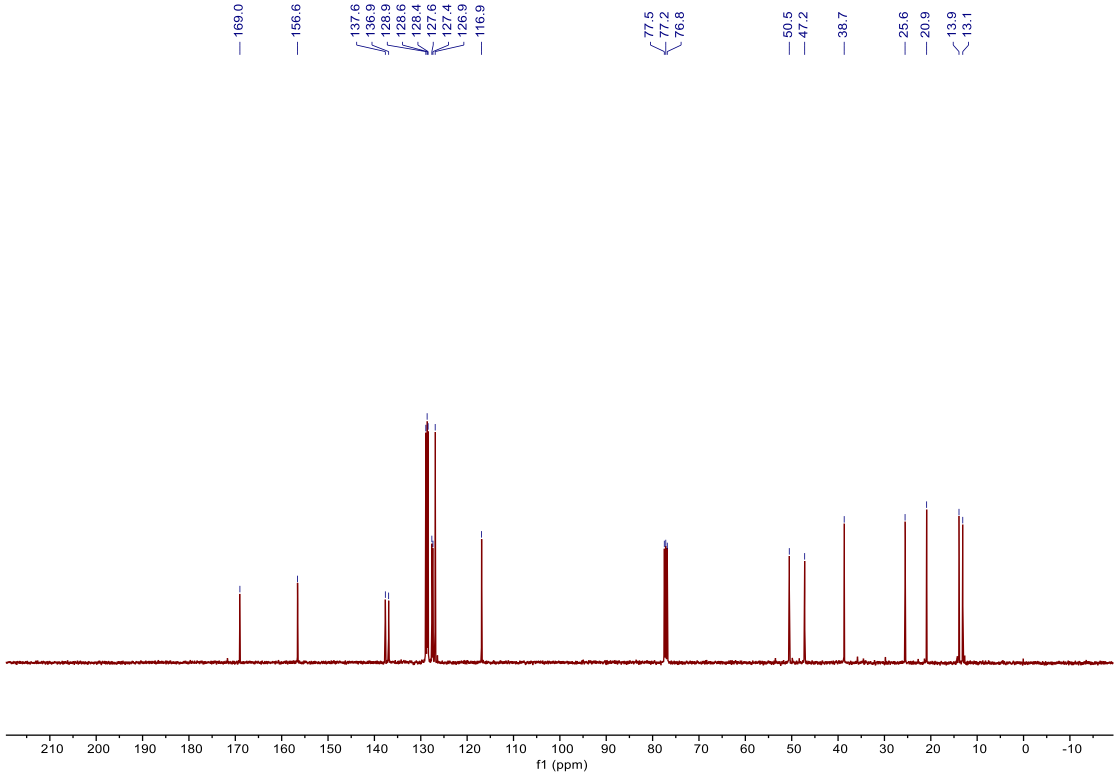

Supplementary Figure 68. ^{13}C NMR (101 MHz, $CDCl_3$) of **1ae**.

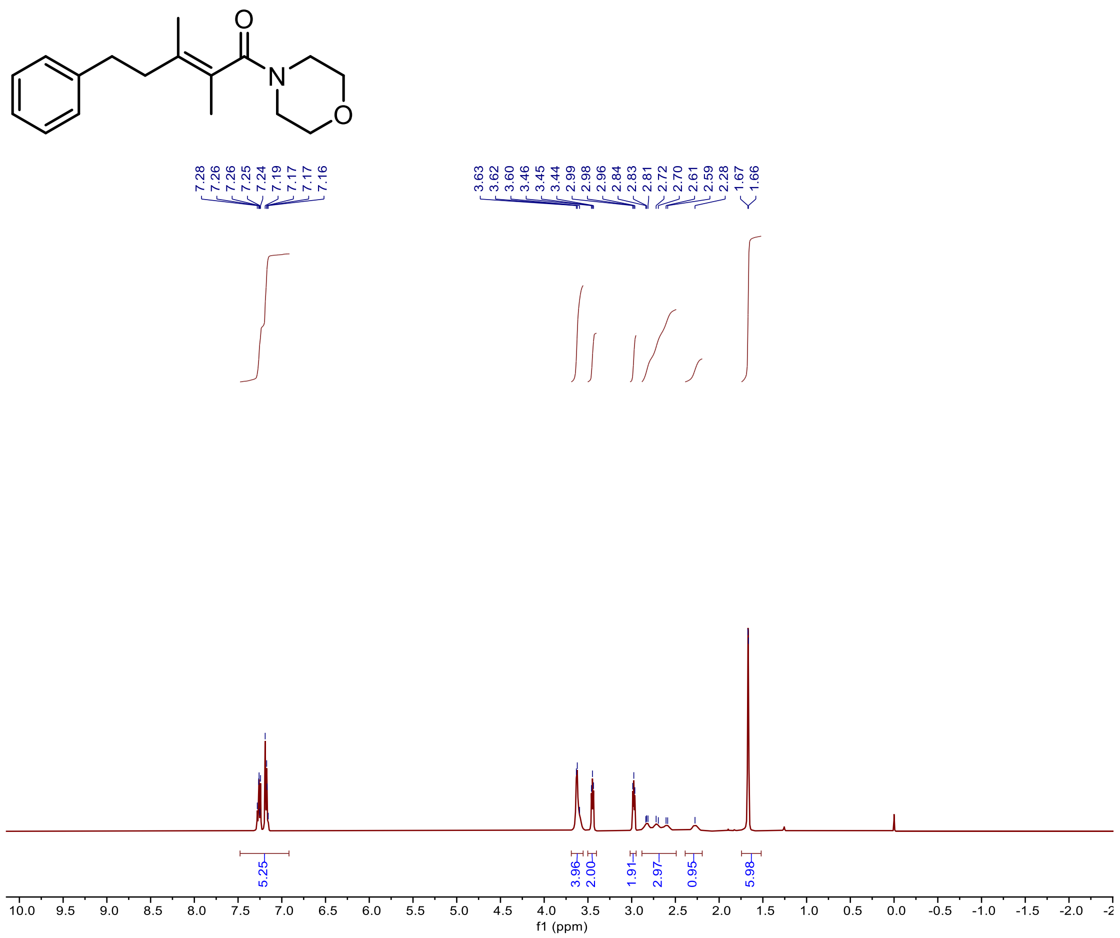

Supplementary Figure 69. ^{1}H NMR (400 MHz, $CDCl_3$) of **1af**.

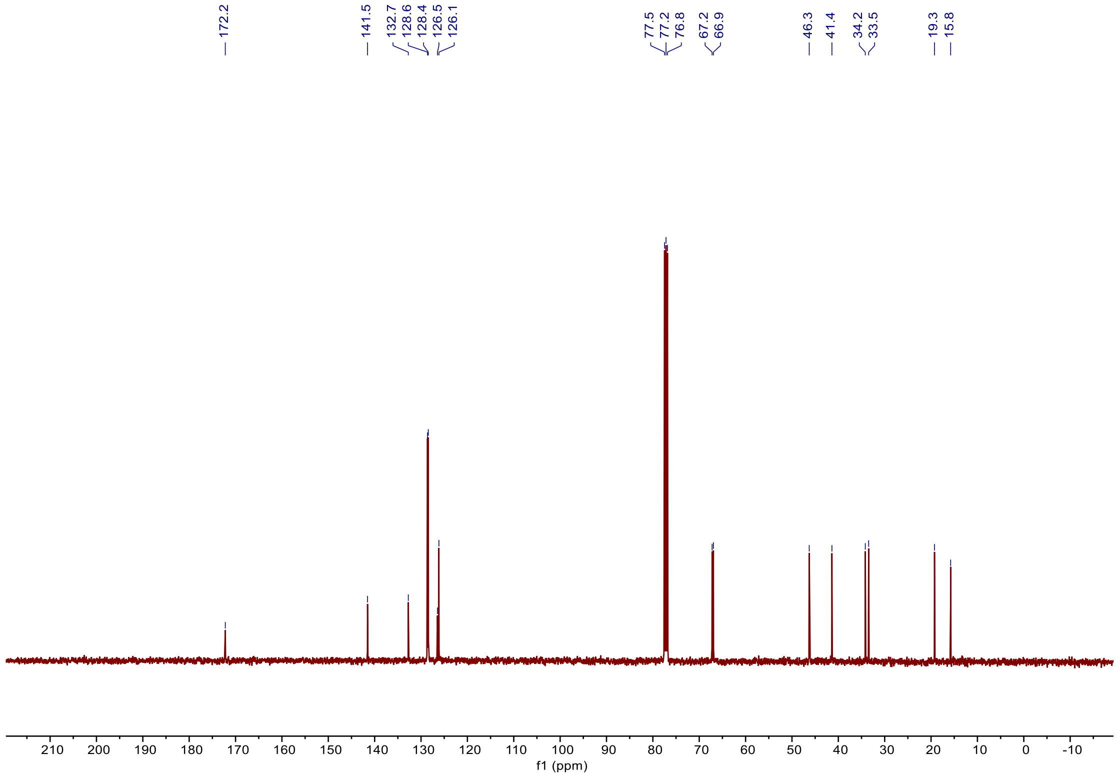

Supplementary Figure 70. ^{13}C NMR (101 MHz, $CDCl_3$) of **1af**.

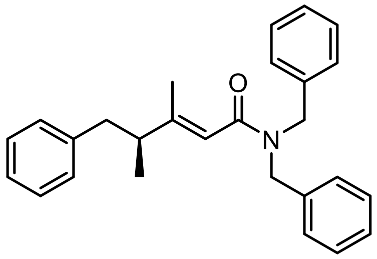

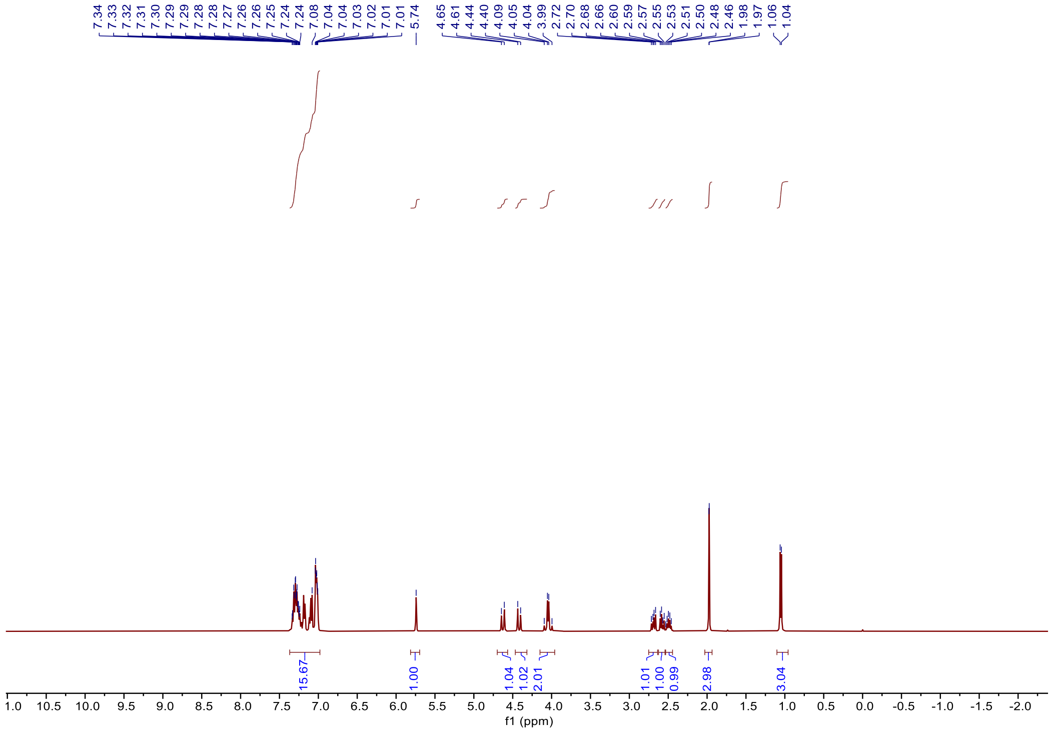

Supplementary Figure 71. ^{1}H NMR (400 MHz, $CDCl_3$) of **1ag**.

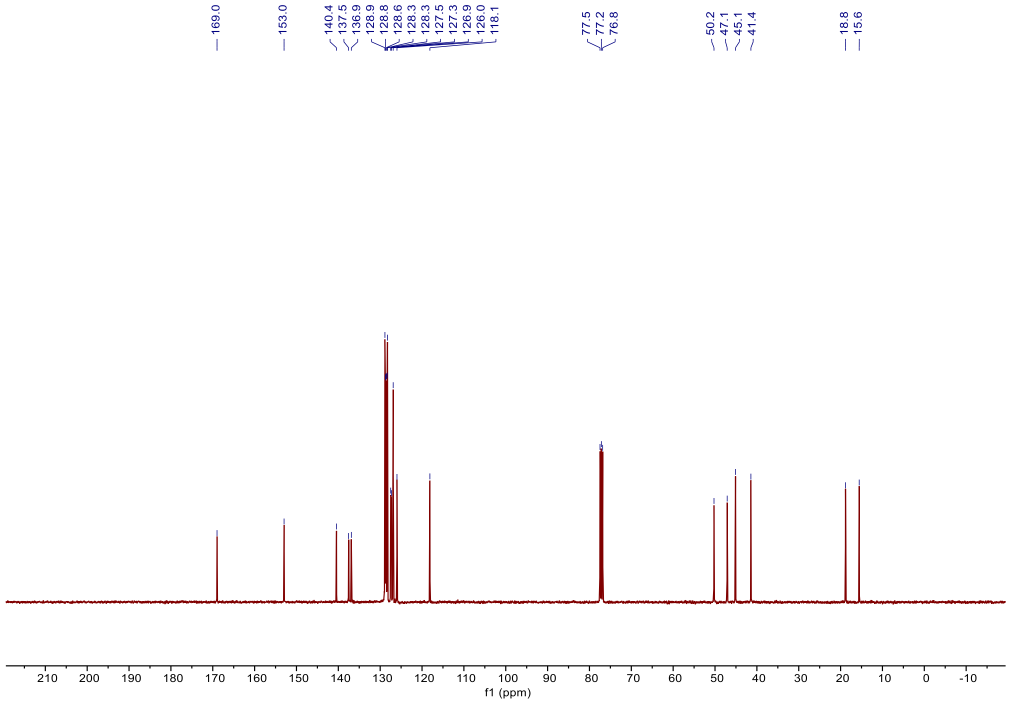

Supplementary Figure 72. ^{13}C NMR (101 MHz, $CDCl_3$) of **1ag**.

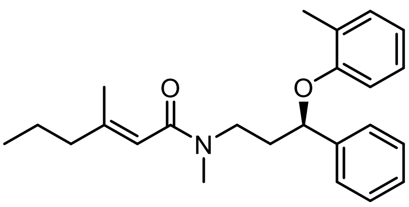

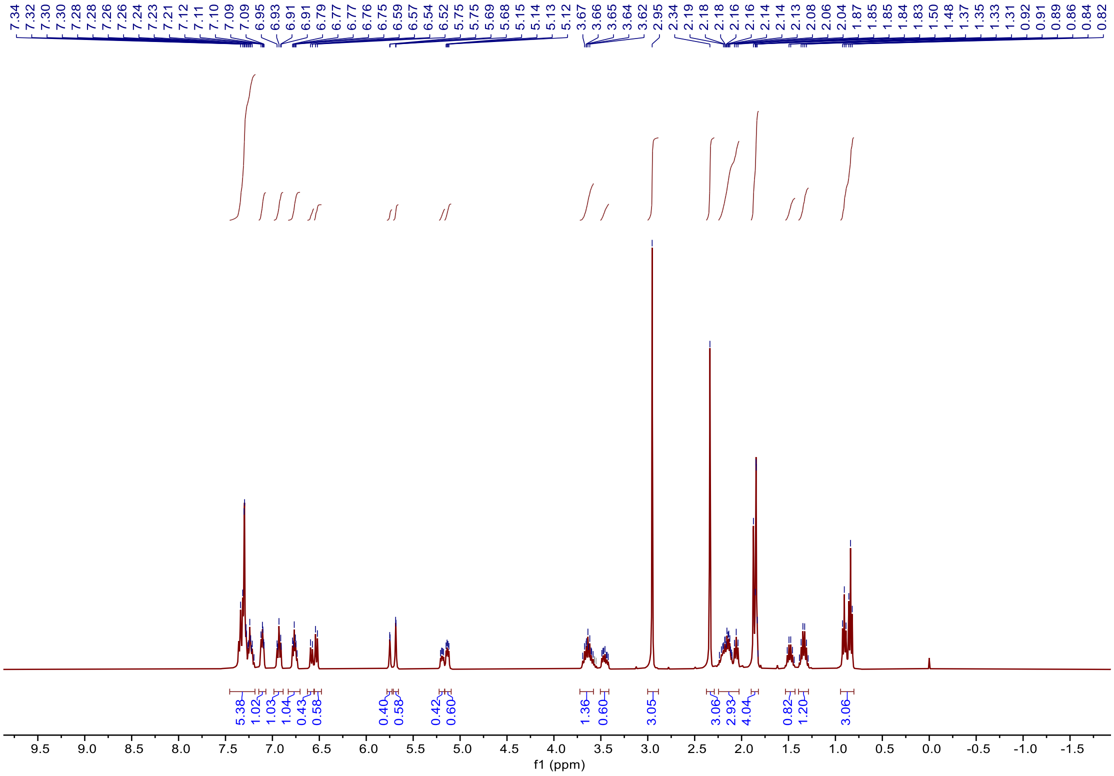

Supplementary Figure 73. ^{1}H NMR (400 MHz, $CDCl_3$) of **1ah**.

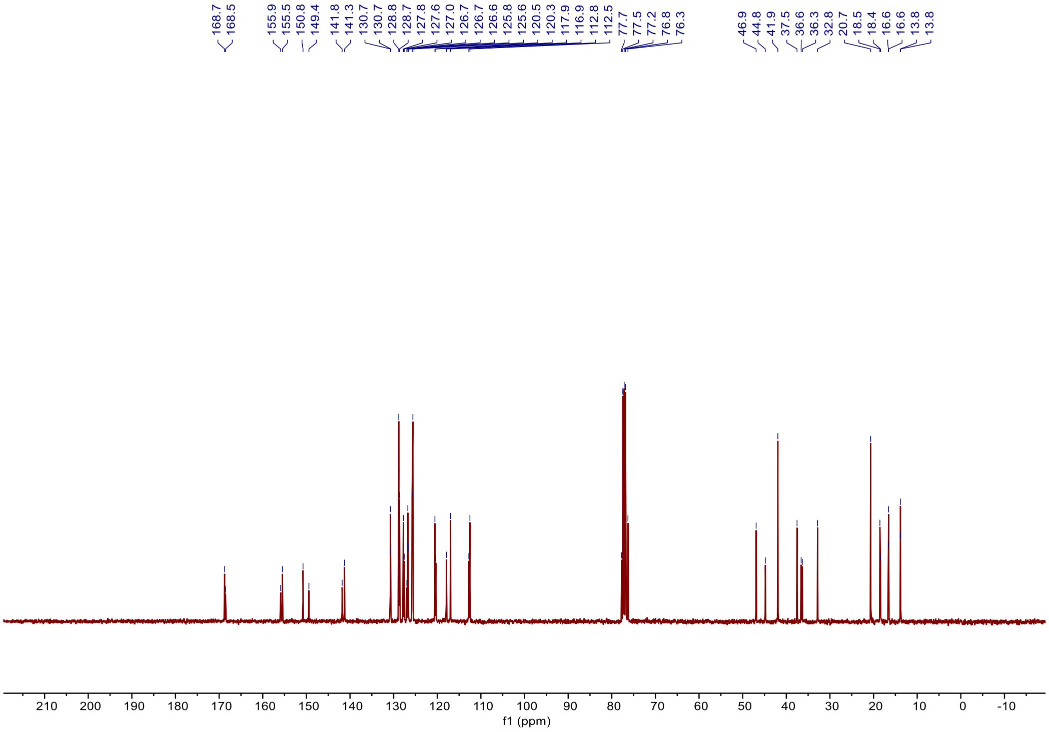

Supplementary Figure 74. ^{13}C NMR (101 MHz, $CDCl_3$) of **1ah**.

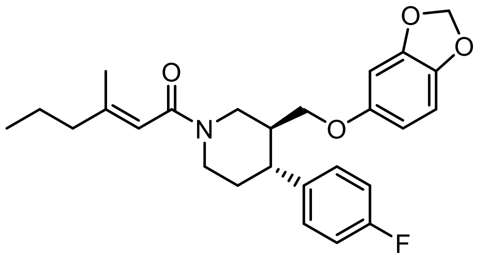

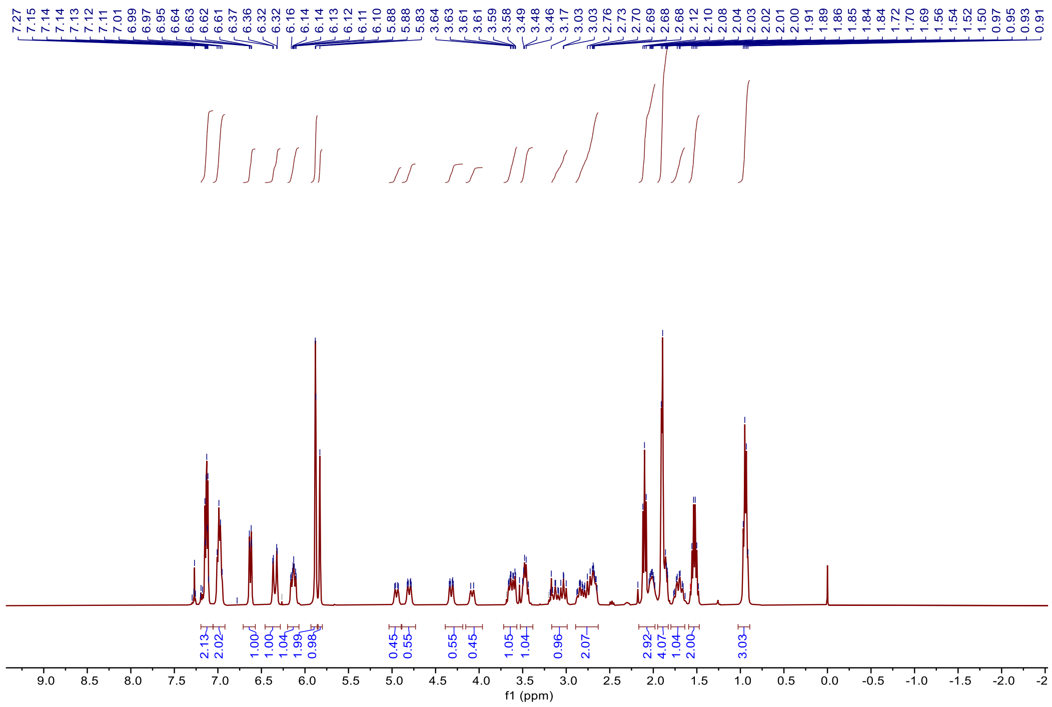

Supplementary Figure 75. ^{1}H NMR (400 MHz, $CDCl_3$) of **1ai**.

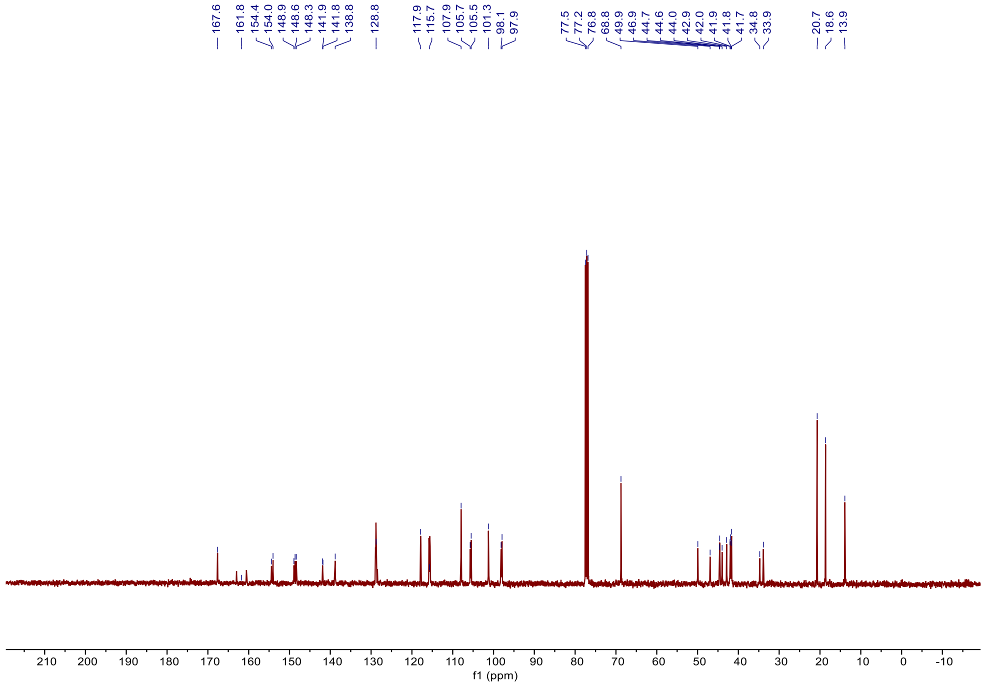

Supplementary Figure 76. ^{13}C NMR (101 MHz, $CDCl_3$) of **1ai**.

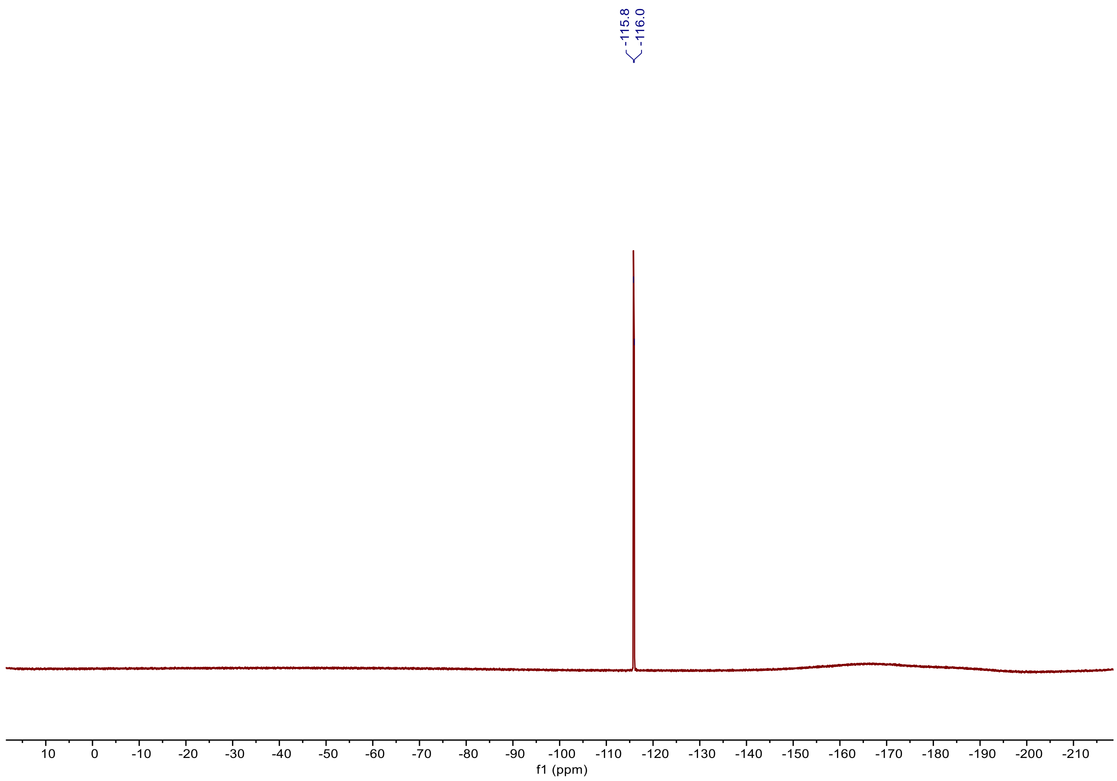

Supplementary Figure 77. ^{19}F NMR (376 MHz, $CDCl_3$) of **1ai**.

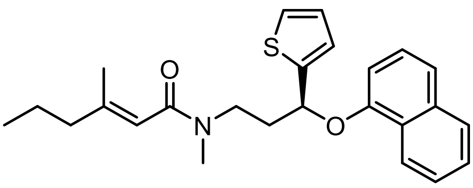

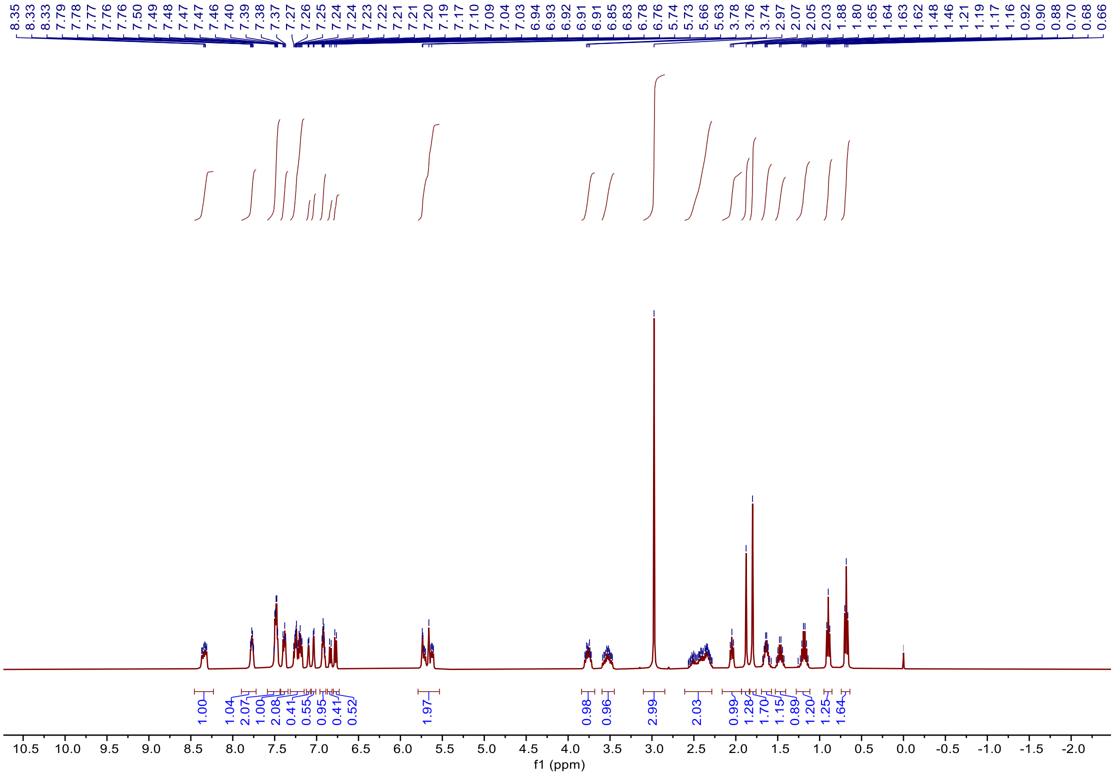

Supplementary Figure 78. ^{1}H NMR (400 MHz, $CDCl_3$) of **1aj**.

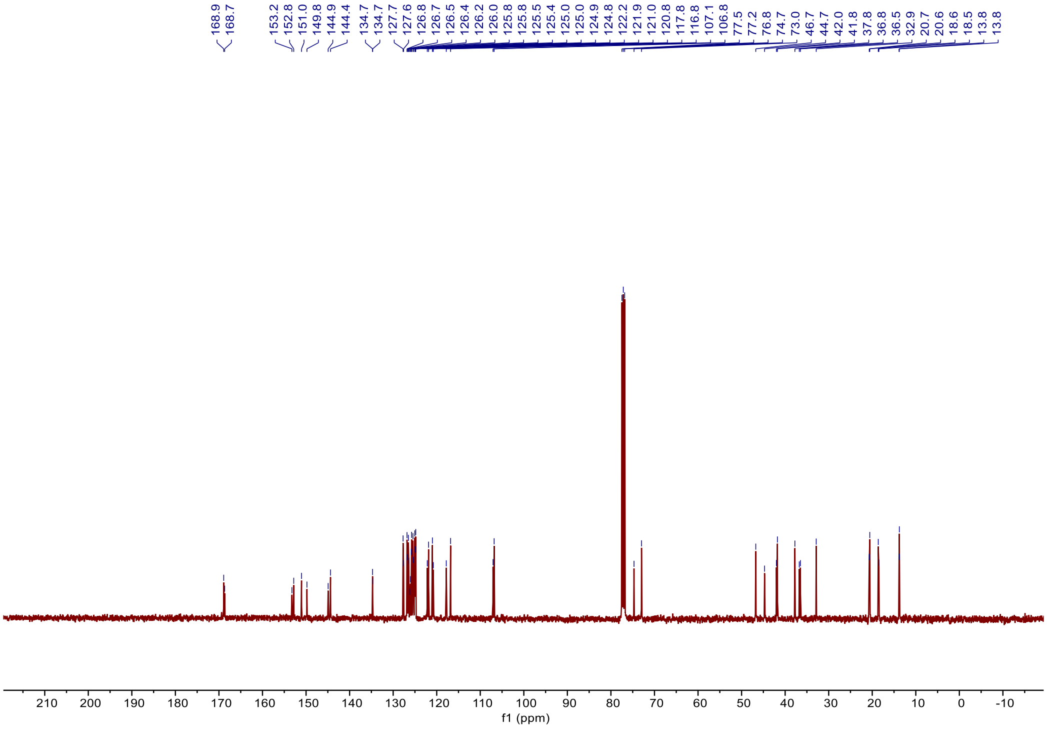

Supplementary Figure 79. ^{13}C NMR (101 MHz, $CDCl_3$) of **1aj**.

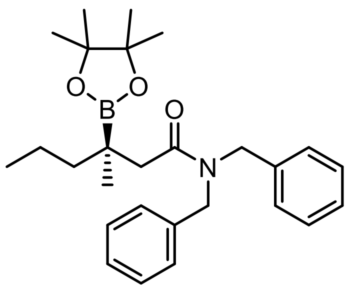

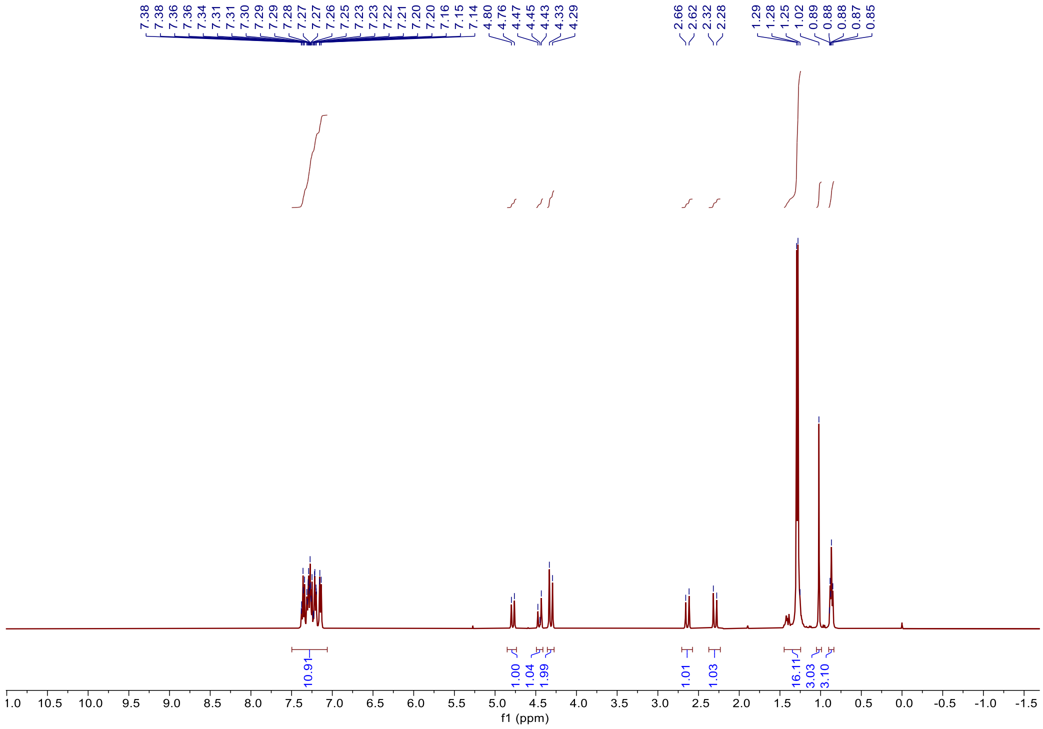

Supplementary Figure 80. ^{1}H NMR (400 MHz, $CDCl_3$) of **3a**.

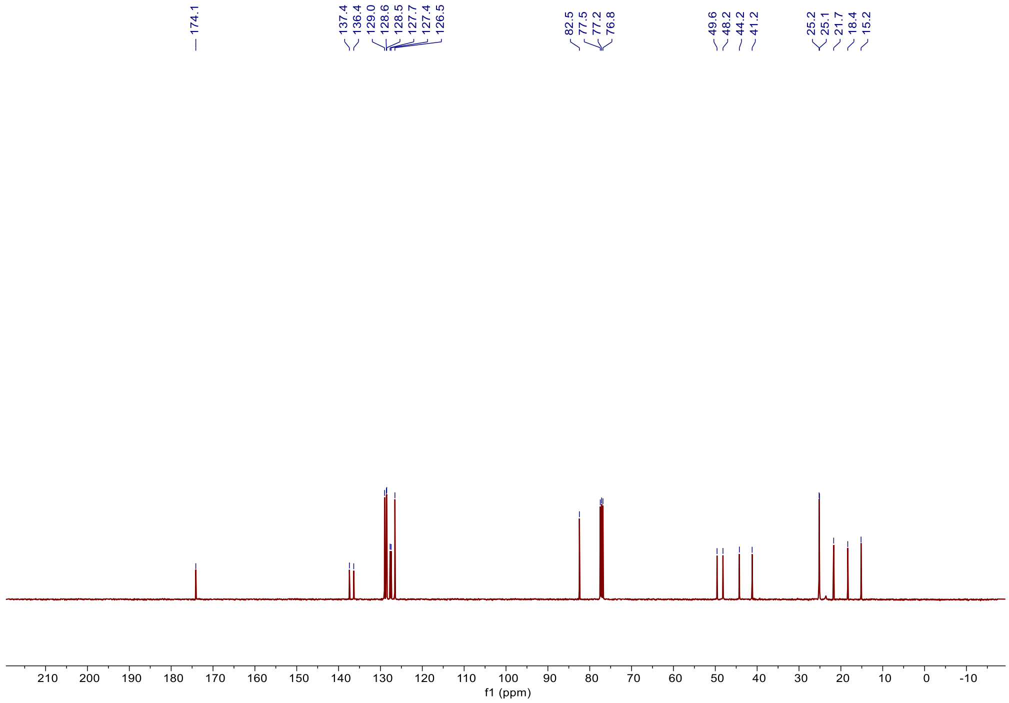

Supplementary Figure 81. ^{13}C NMR (101 MHz, $CDCl_3$) of **3a**.

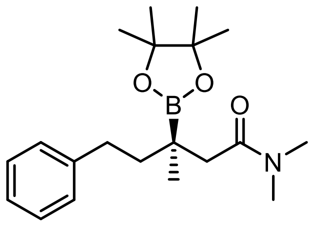

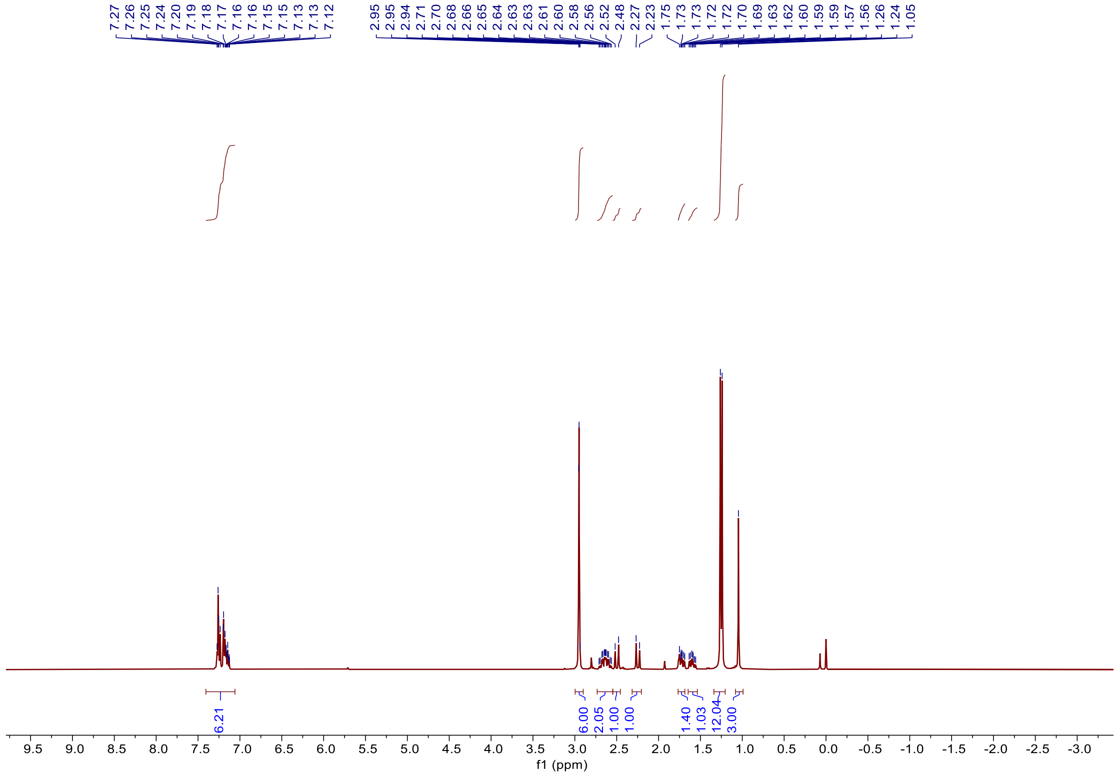

Supplementary Figure 82. ^{1}H NMR (400 MHz, $CDCl_3$) of **3b**.

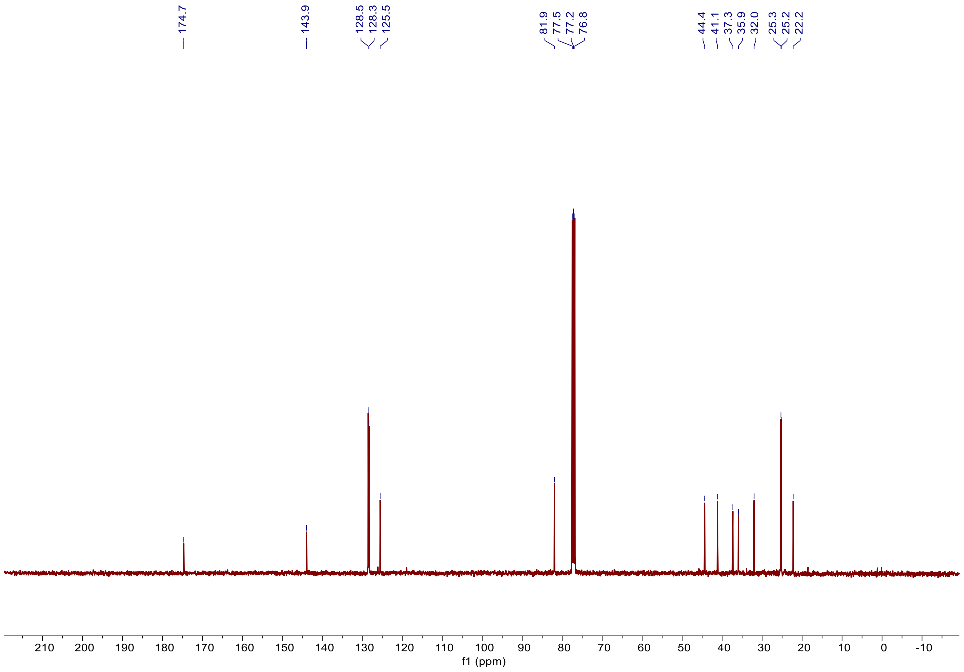

Supplementary Figure 83. ^{13}C NMR (101 MHz, $CDCl_3$) of **3b**.

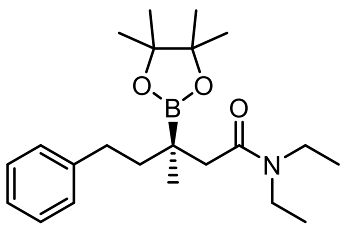

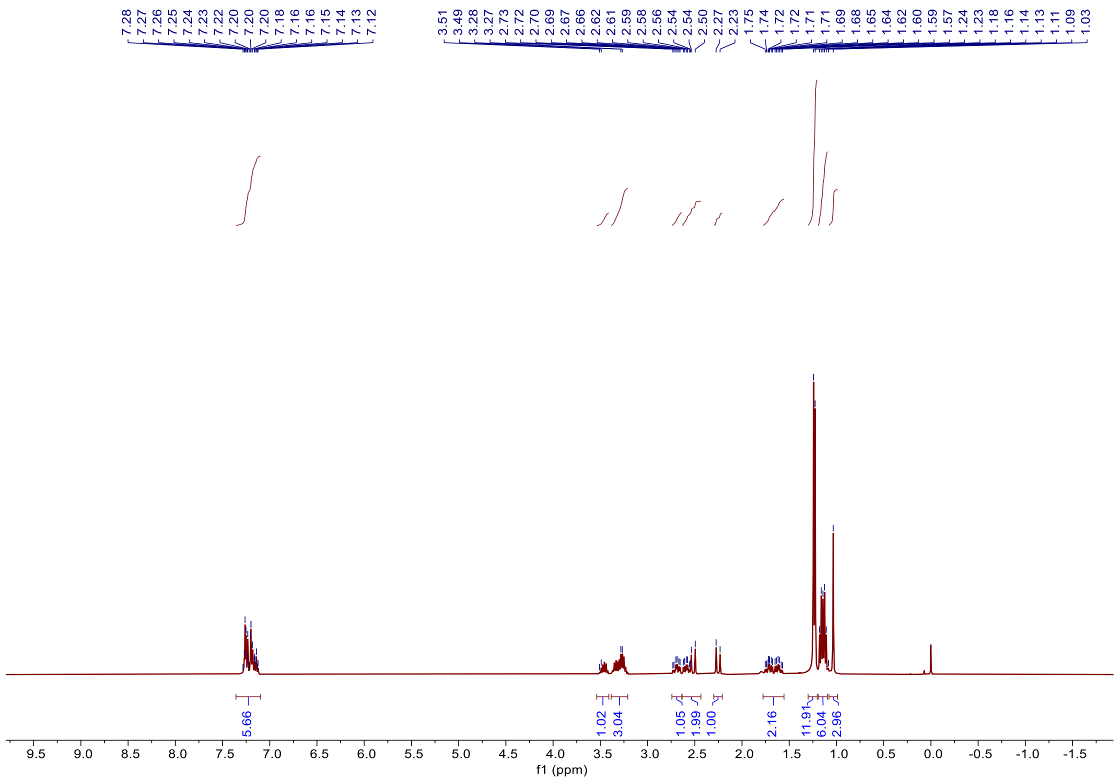

Supplementary Figure 84. ^{1}H NMR (400 MHz, $CDCl_3$) of **3c**.

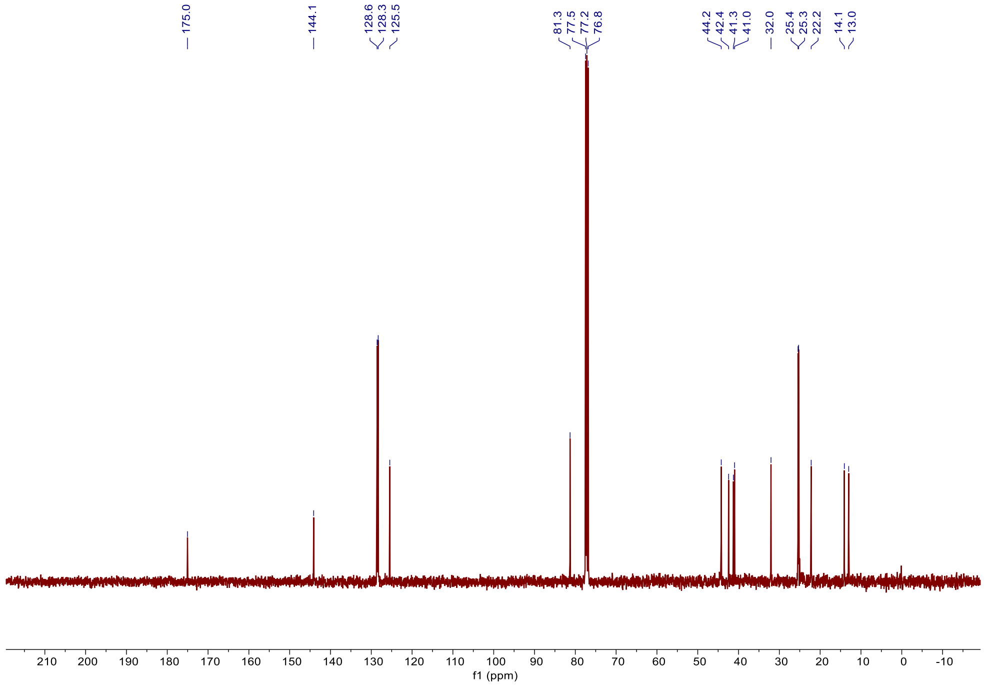

Supplementary Figure 85. ^{13}C NMR (101 MHz, $CDCl_3$) of **3c**.

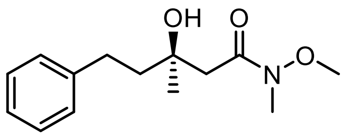

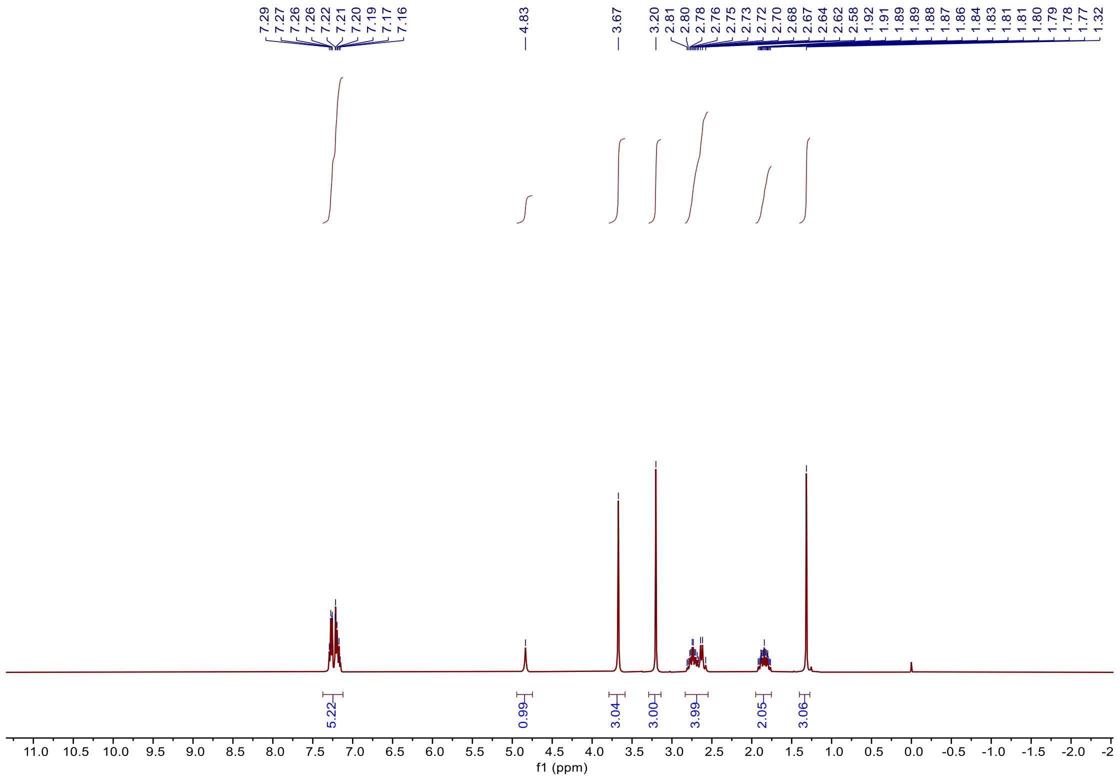

Supplementary Figure 86. ^{1}H NMR (400 MHz, $CDCl_3$) of **3d-ol**.

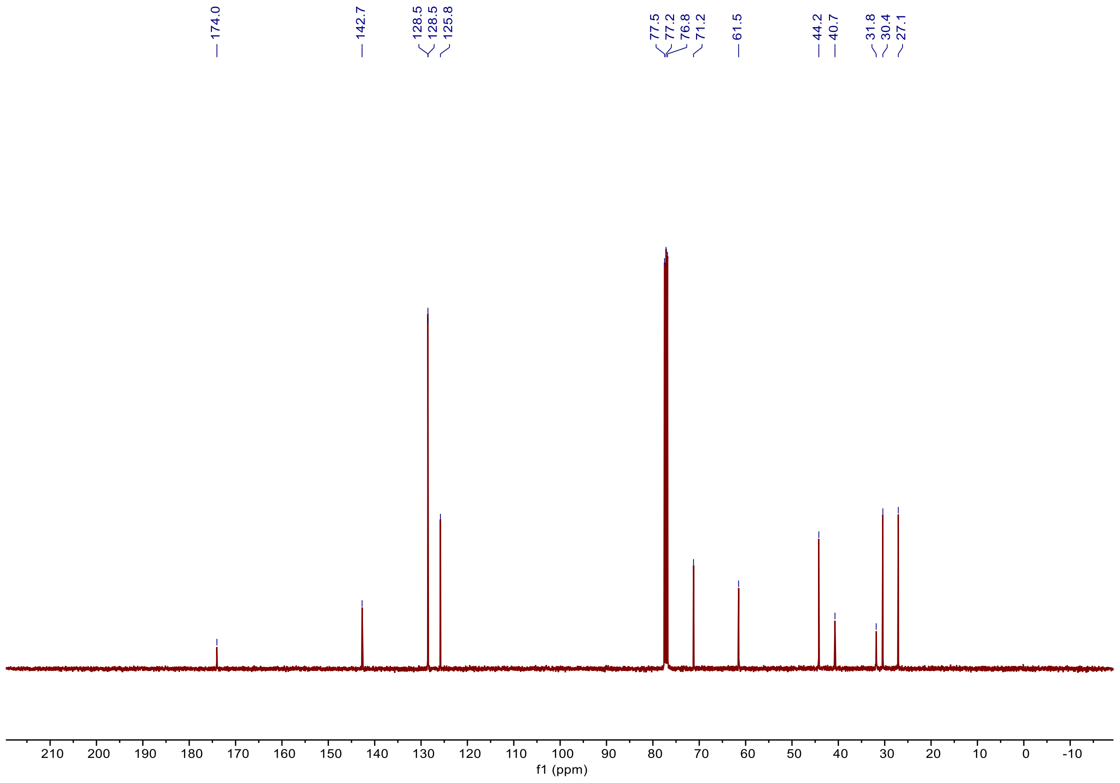

Supplementary Figure 87. ^{13}C NMR (101 MHz, $CDCl_3$) of **3d-ol**.

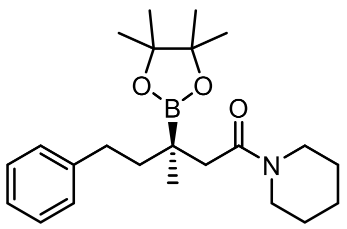

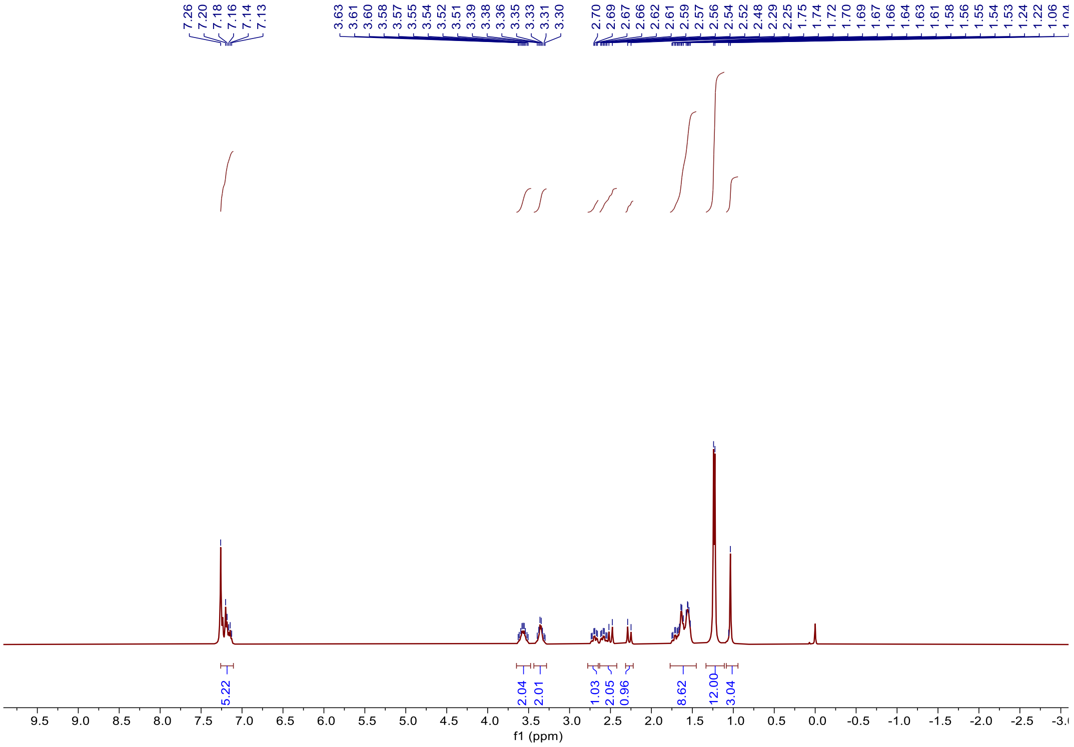

Supplementary Figure 88. ^{1}H NMR (400 MHz, $CDCl_3$) of **3e**.

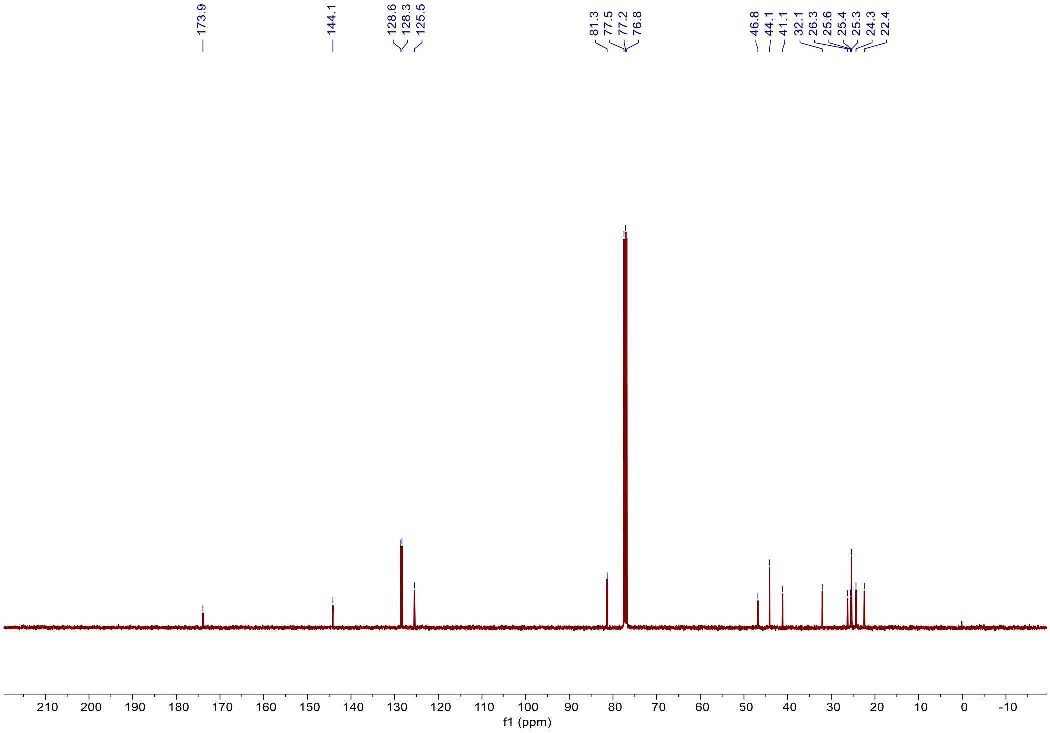

Supplementary Figure 89. ^{13}C NMR (101 MHz, $CDCl_3$) of **3e**.

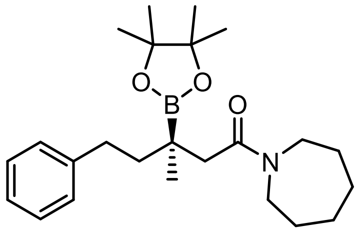

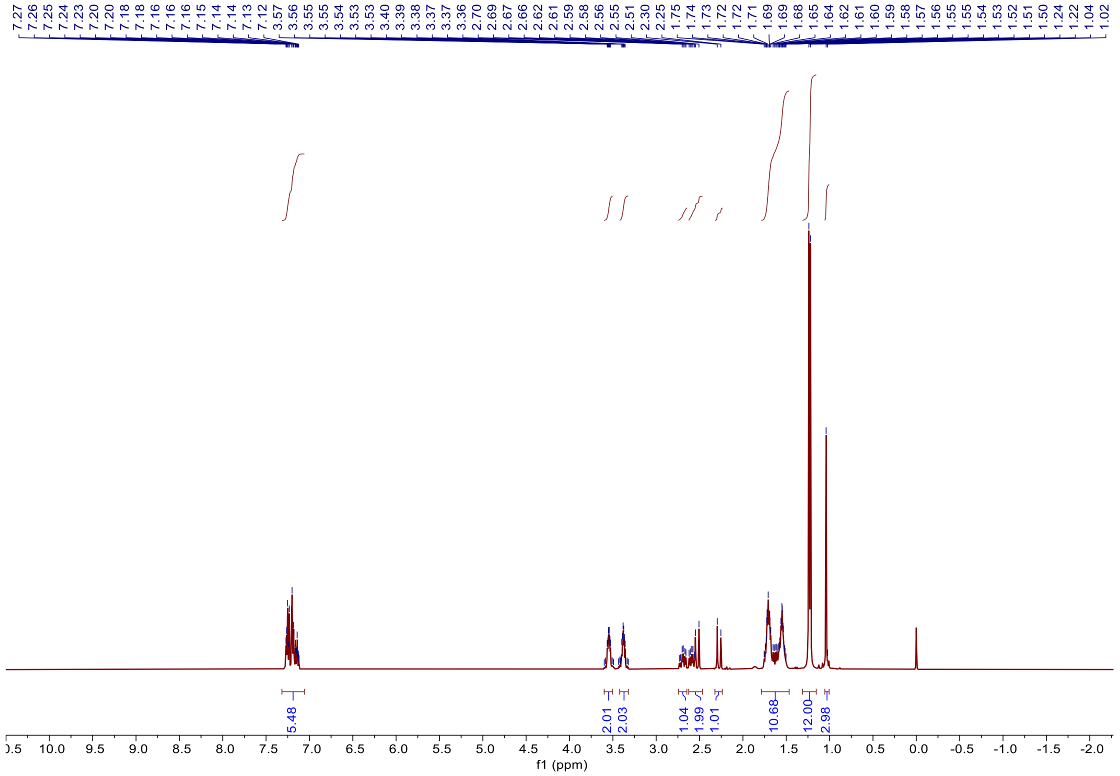

Supplementary Figure 90. ^{1}H NMR (400 MHz, $CDCl_3$) of **3f**.

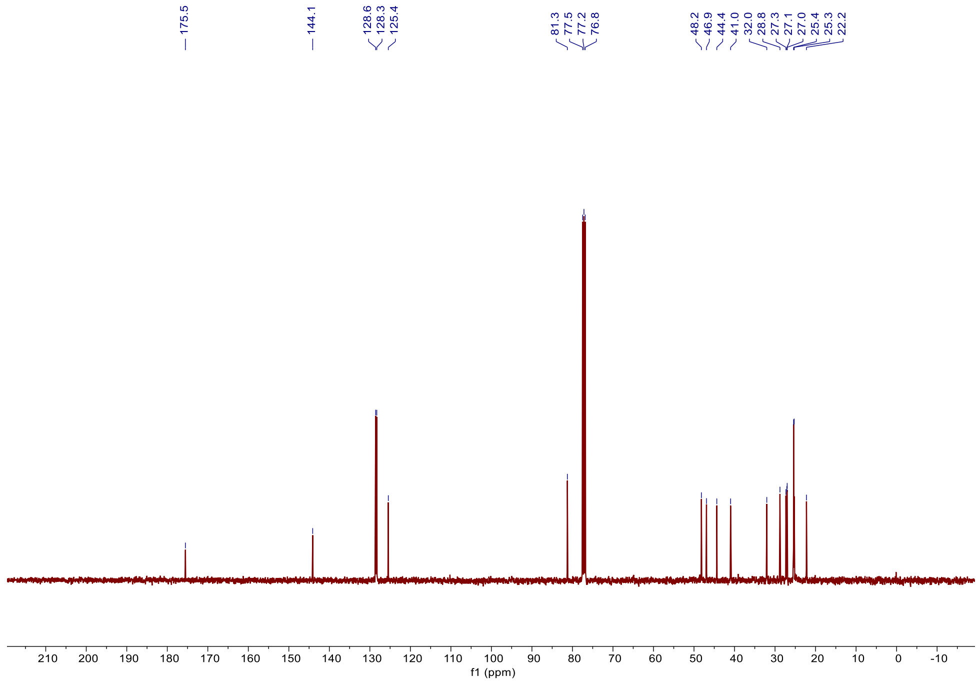

Supplementary Figure 91. ^{13}C NMR (101 MHz, $CDCl_3$) of **3f**.

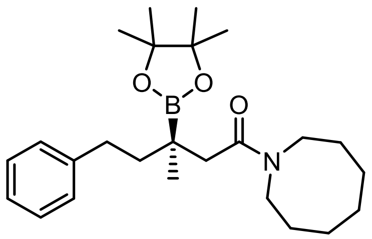

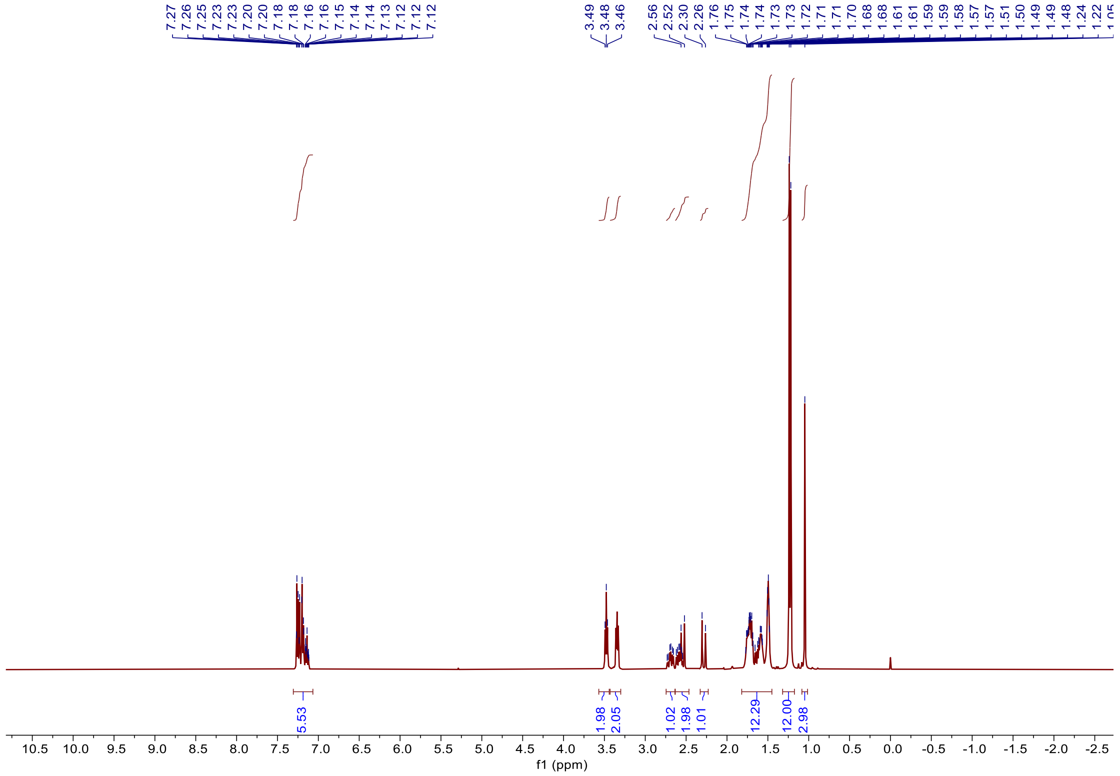

Supplementary Figure 92. ^{1}H NMR (400 MHz, $CDCl_3$) of **3g**.

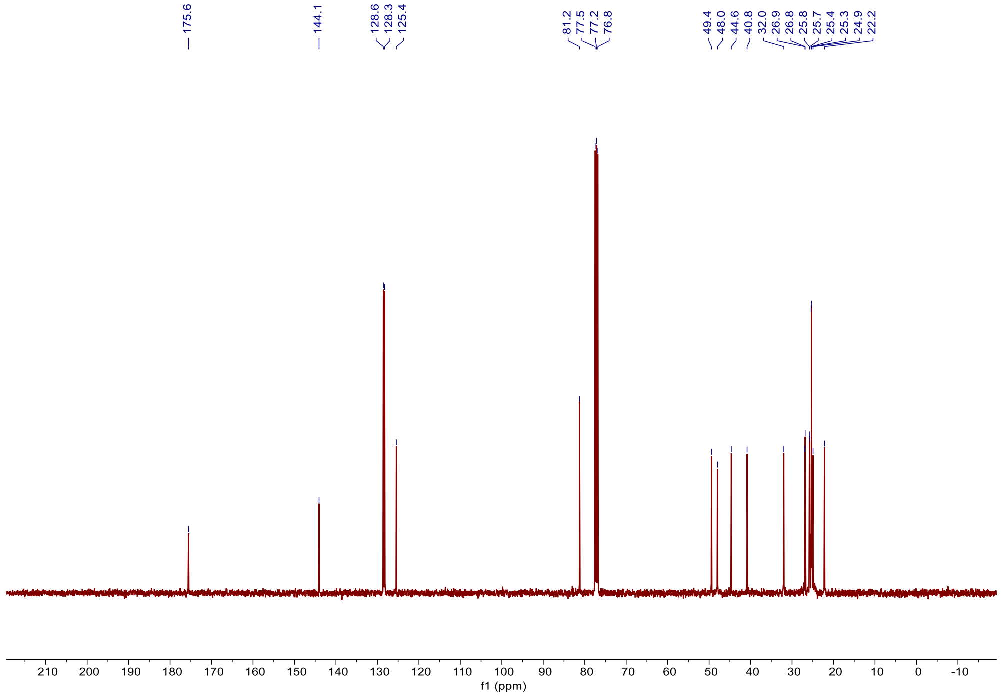

Supplementary Figure 93. ^{13}C NMR (101 MHz, $CDCl_3$) of **3g**.

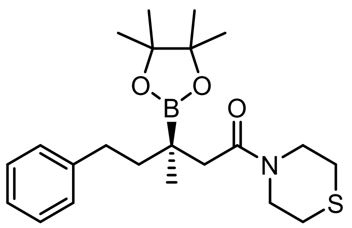

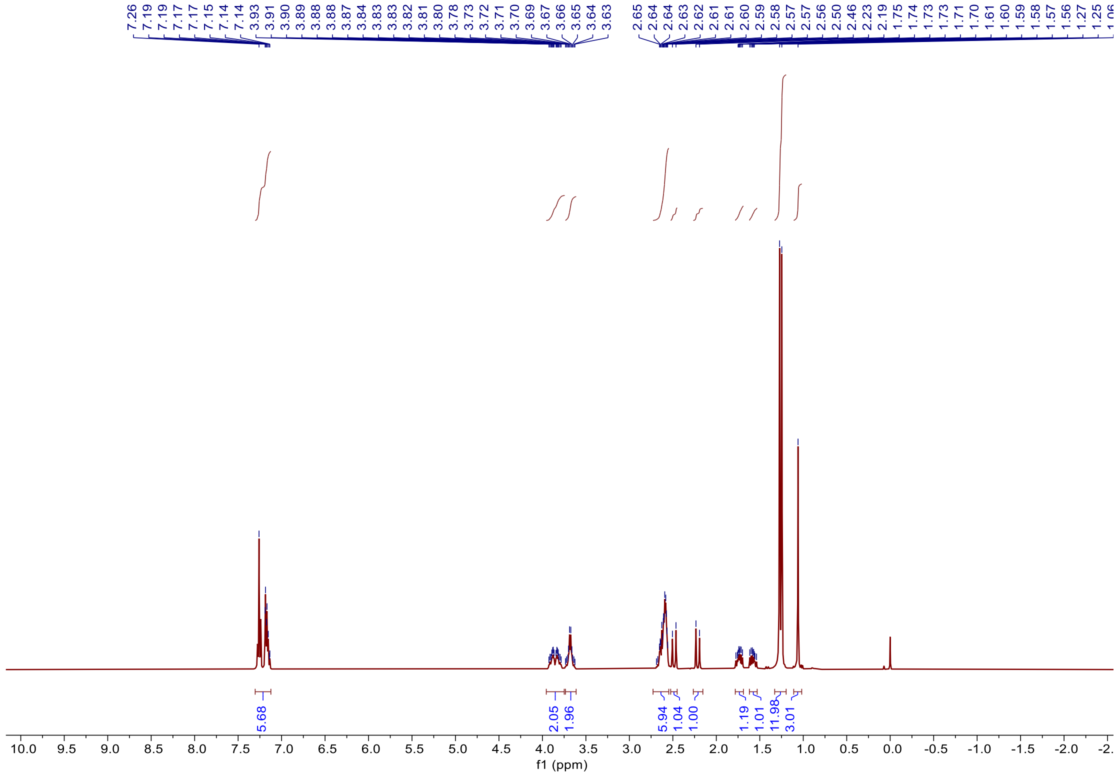

Supplementary Figure 94. ^{1}H NMR (400 MHz, $CDCl_3$) of **3h**.

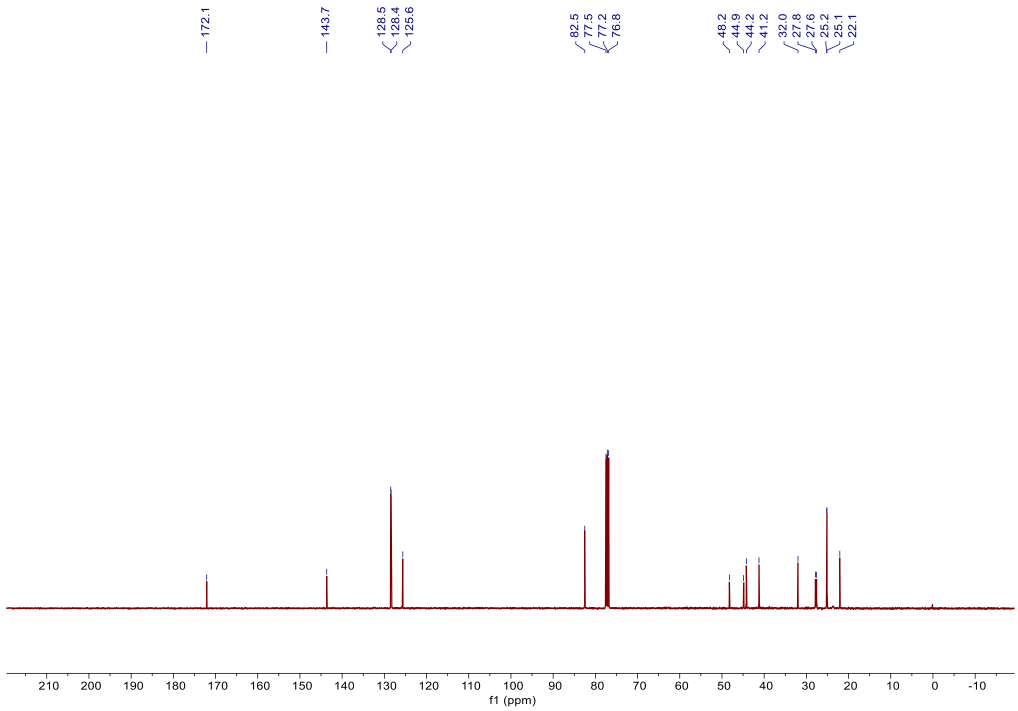

Supplementary Figure 95. ^{13}C NMR (101 MHz, $CDCl_3$) of **3h**.

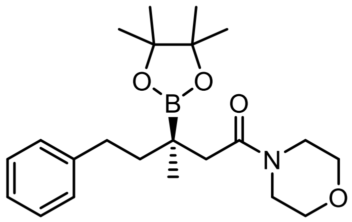

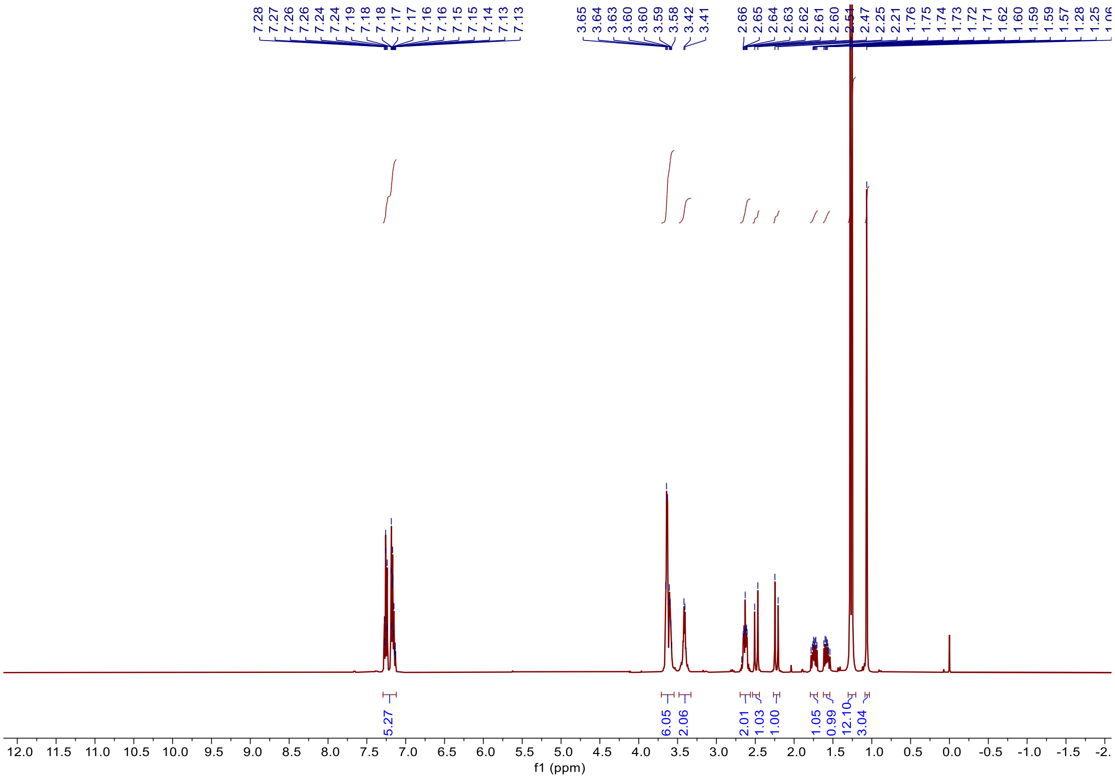

Supplementary Figure 96. ^{1}H NMR (400 MHz, $CDCl_3$) of **3i**.

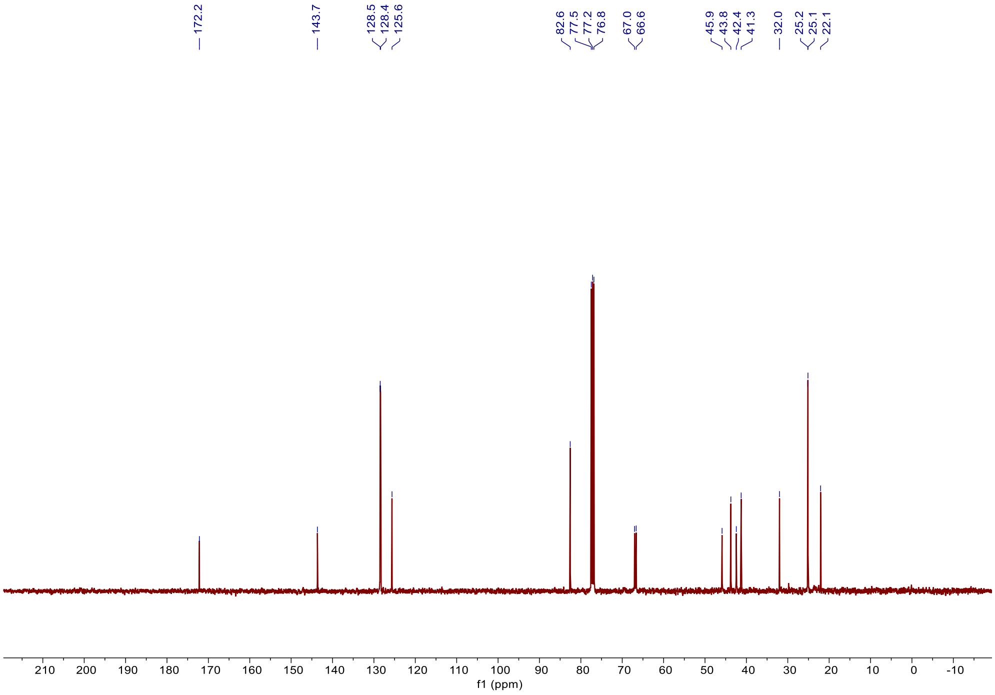

Supplementary Figure 97. ^{13}C NMR (101 MHz, $CDCl_3$) of **3i**.

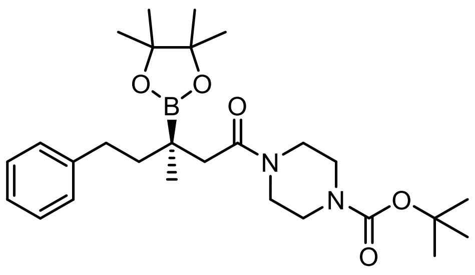

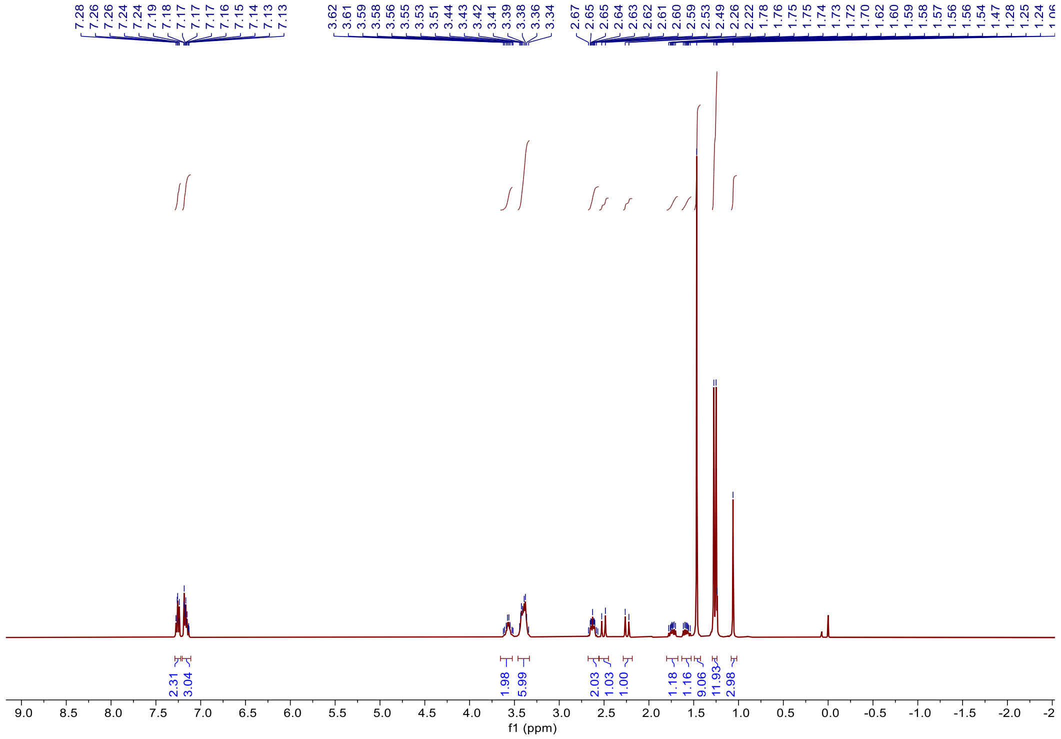

Supplementary Figure 98. ^{1}H NMR (400 MHz, $CDCl_3$) of **3j**.

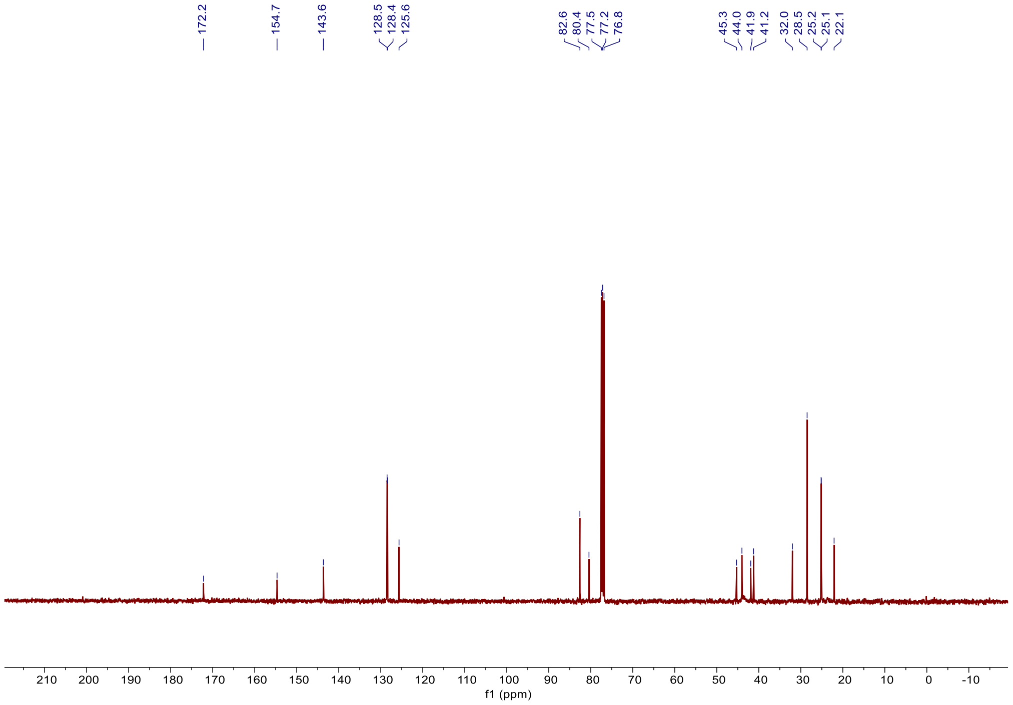

Supplementary Figure 99. ^{13}C NMR (101 MHz, $CDCl_3$) of **3j**.

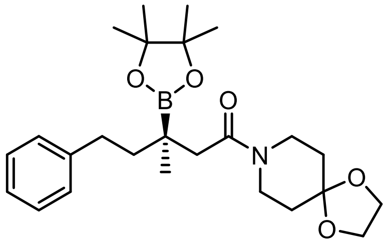

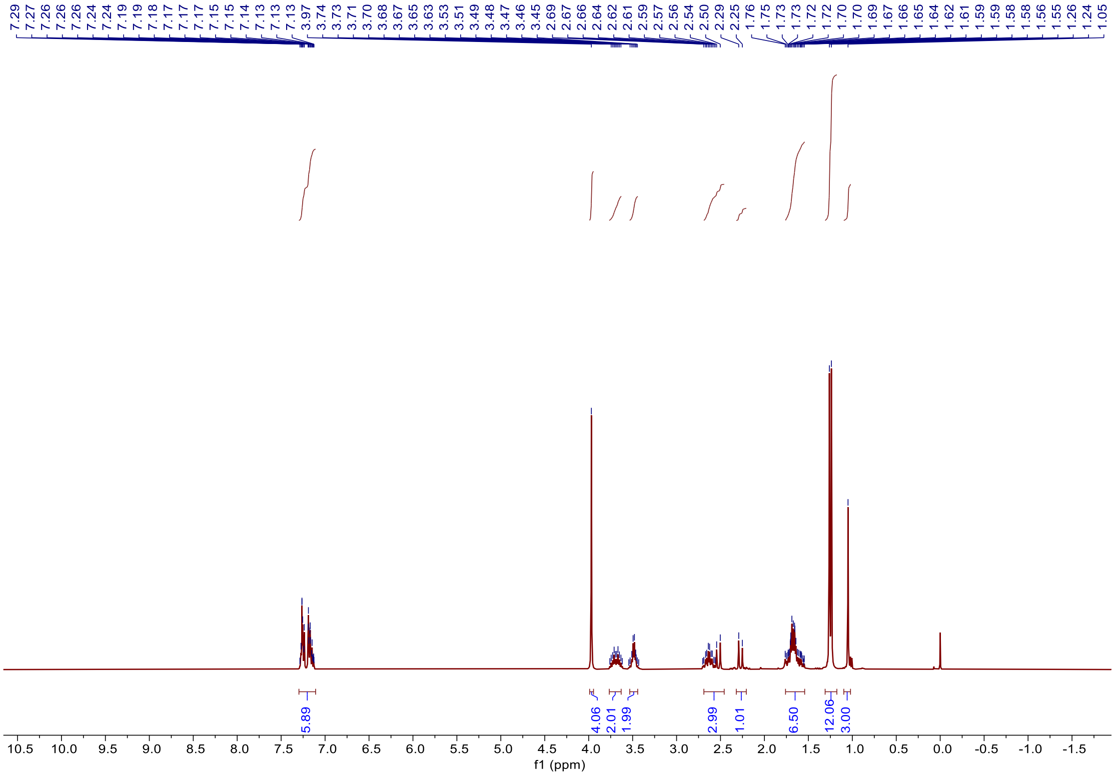

Supplementary Figure 100. ^{1}H NMR (400 MHz, $CDCl_3$) of **3k**.

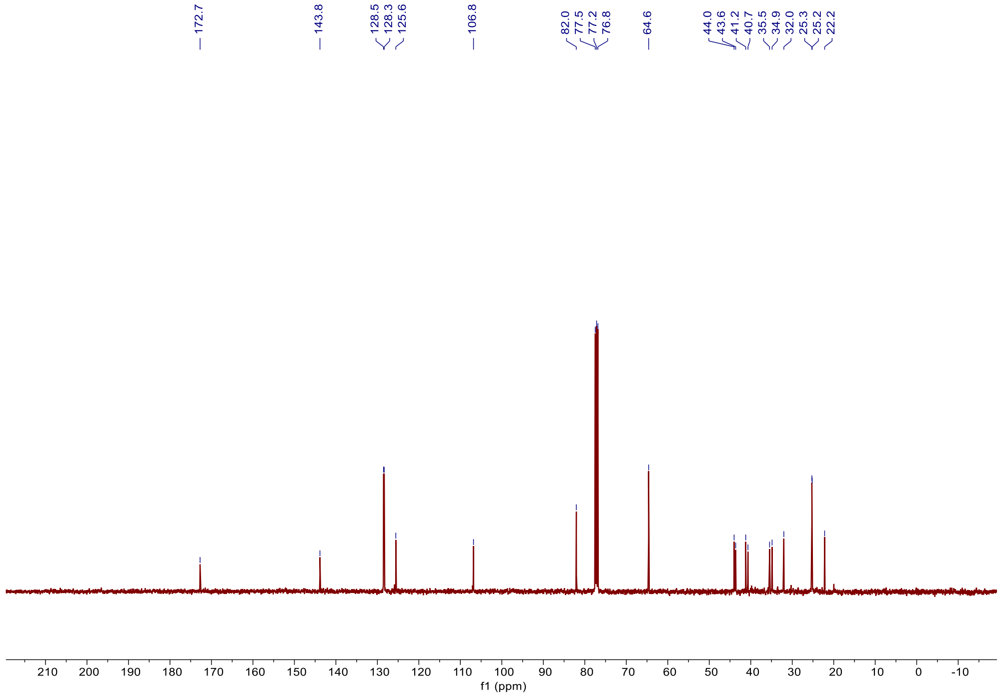

Supplementary Figure 101. ^{13}C NMR (101 MHz, $CDCl_3$) of **3k**.

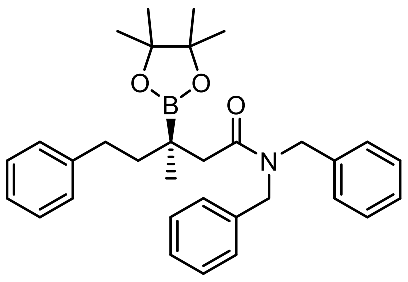

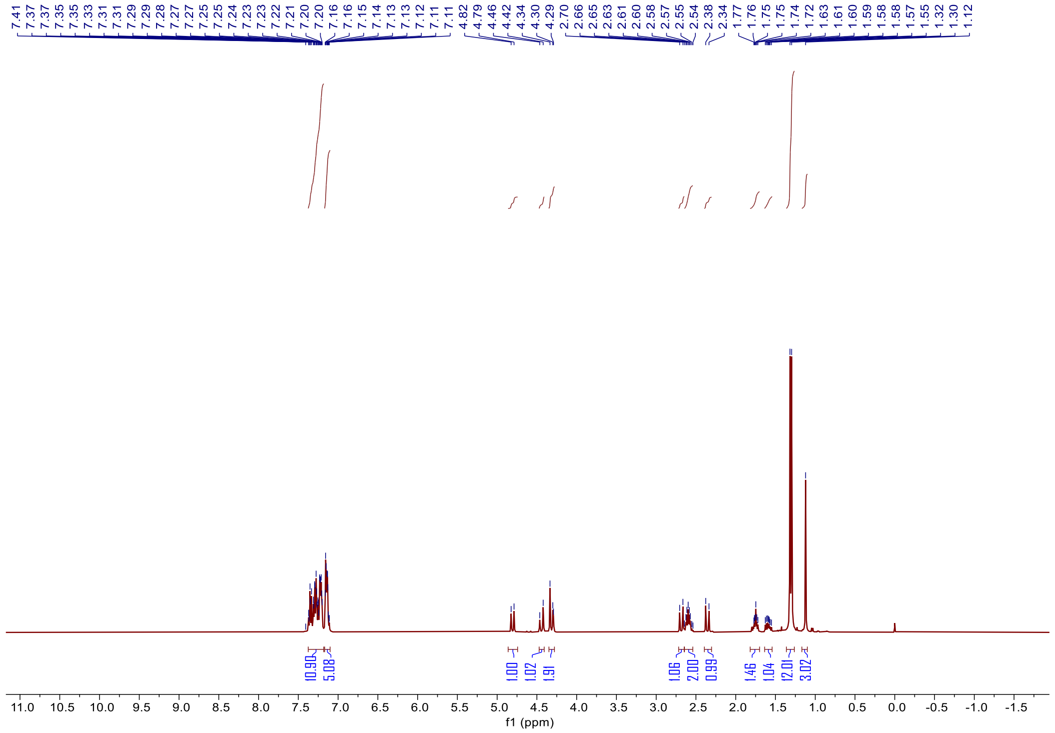

Supplementary Figure 102. ^{1}H NMR (400 MHz, $CDCl_3$) of **3l**.

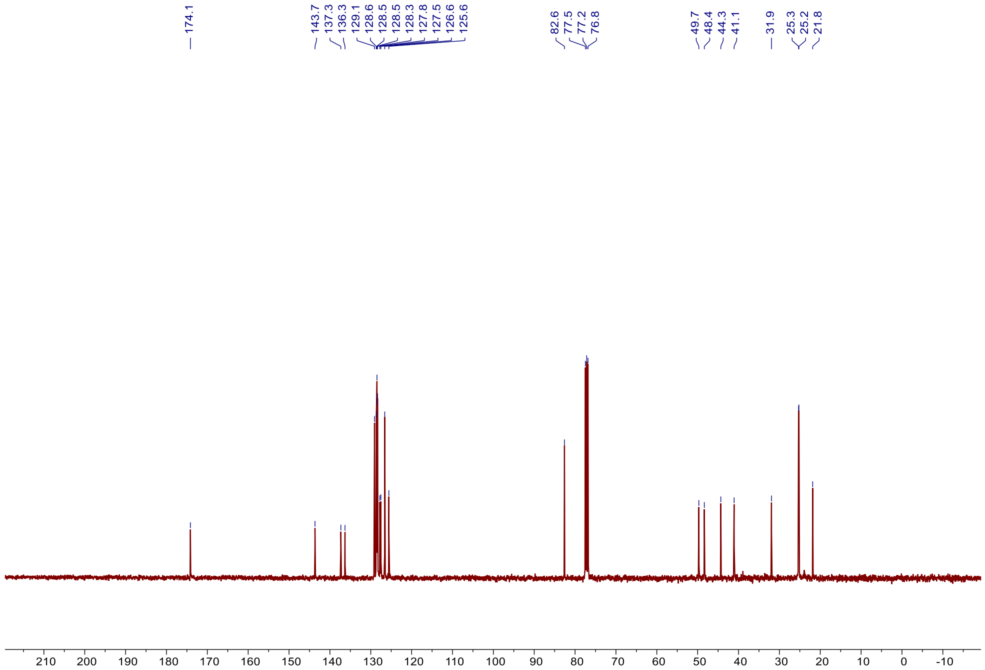

Supplementary Figure 103. ^{13}C NMR (101 MHz, $CDCl_3$) of **3l**.

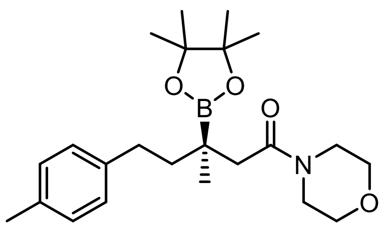

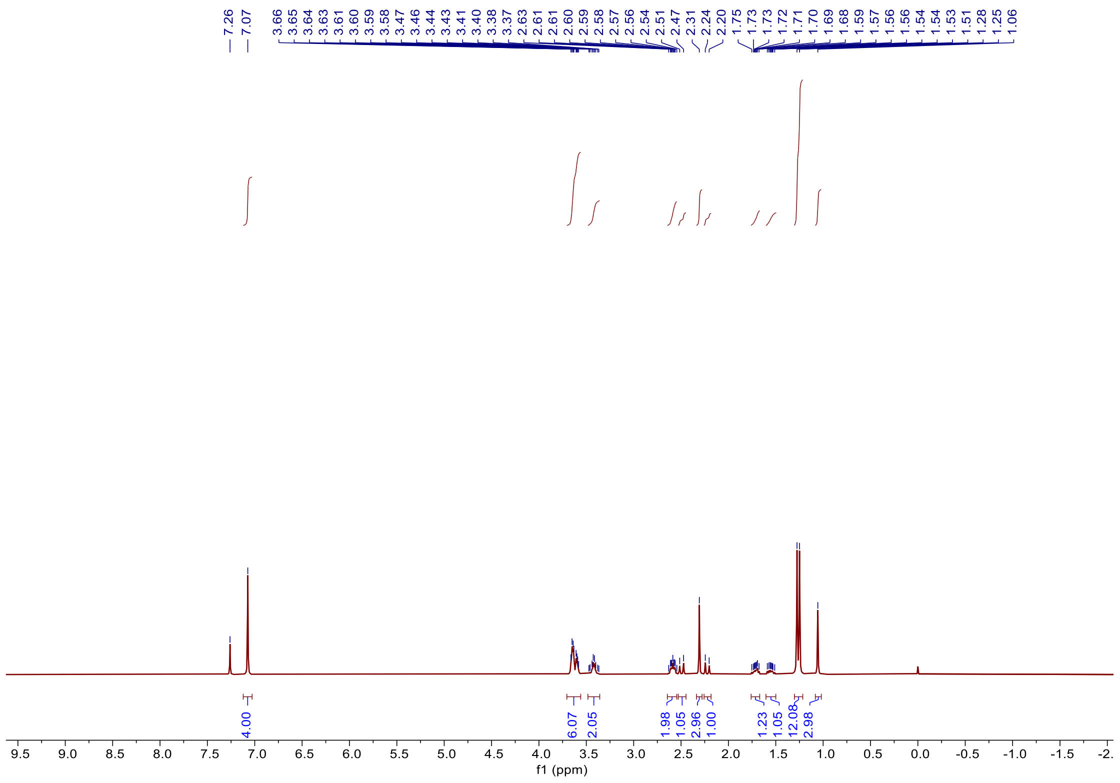

Supplementary Figure 104. ^{1}H NMR (400 MHz, $CDCl_3$) of **3m**.

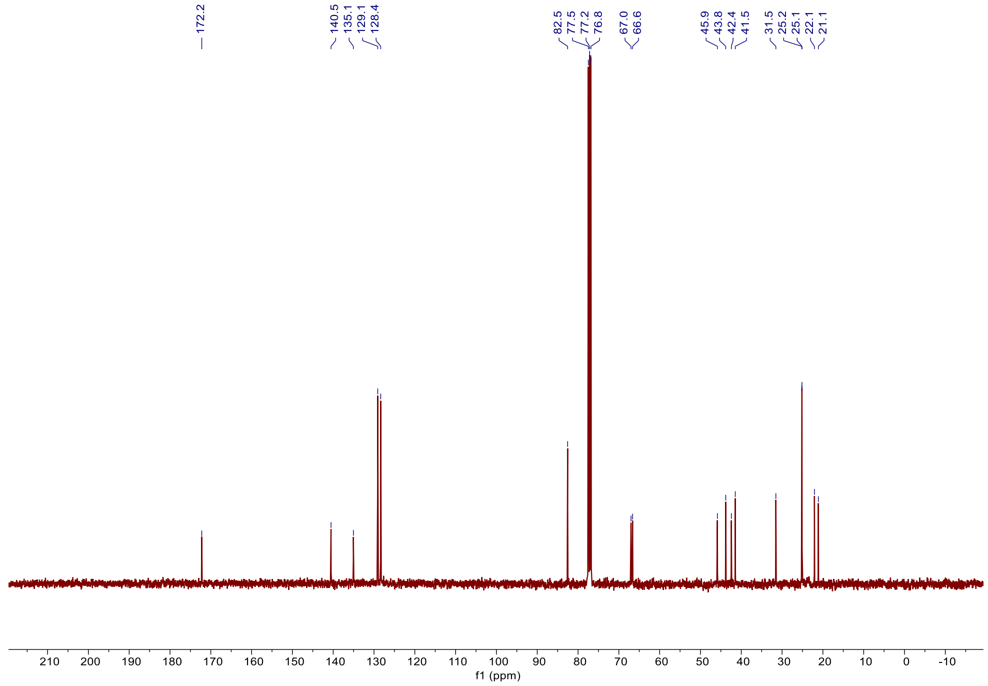

Supplementary Figure 105. ^{13}C NMR (101 MHz, $CDCl_3$) of **3m**.

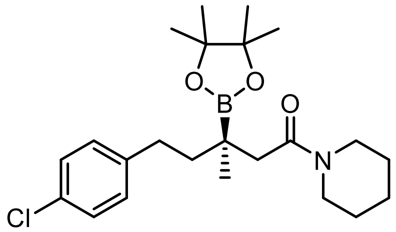

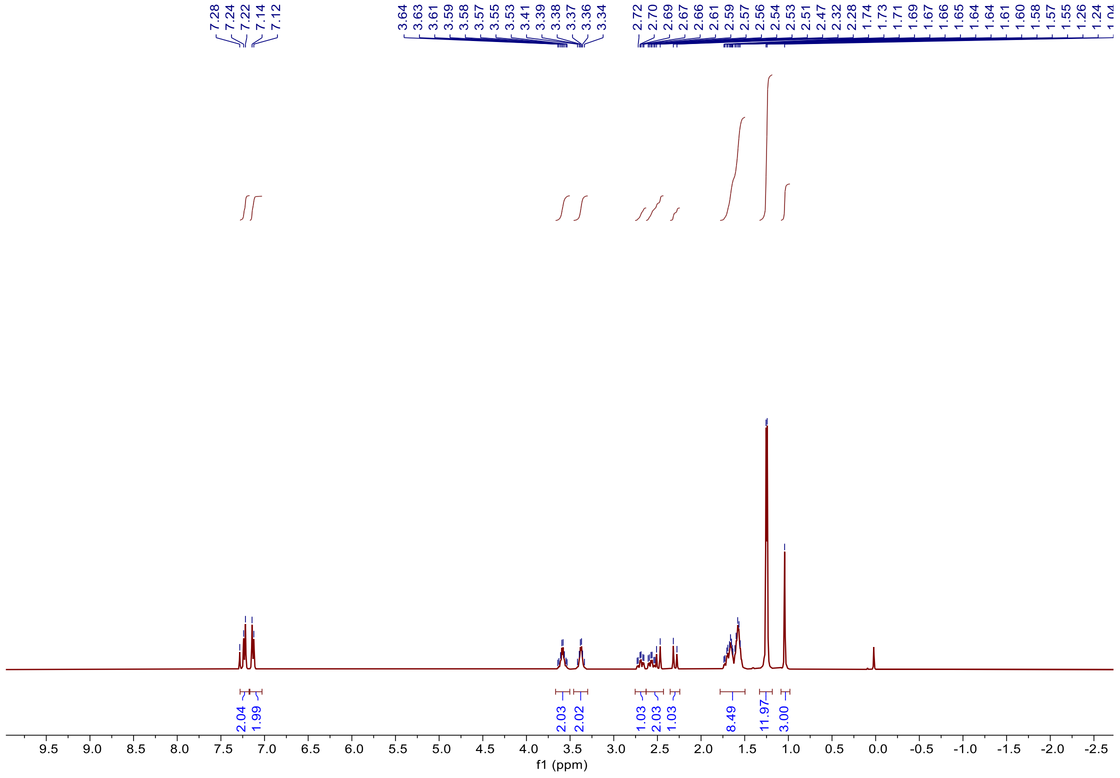

Supplementary Figure 106. ^{1}H NMR (400 MHz, $CDCl_3$) of **3n**.

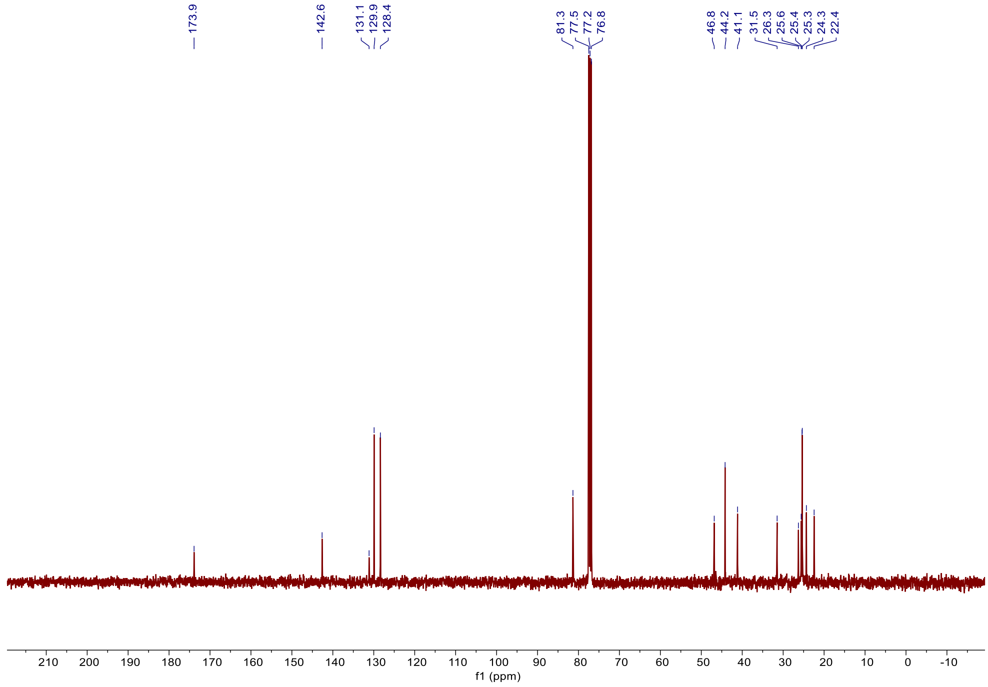

Supplementary Figure 107. ^{13}C NMR (101 MHz, $CDCl_3$) of **3n**.

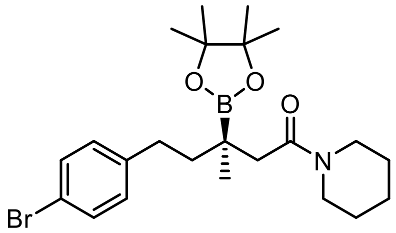

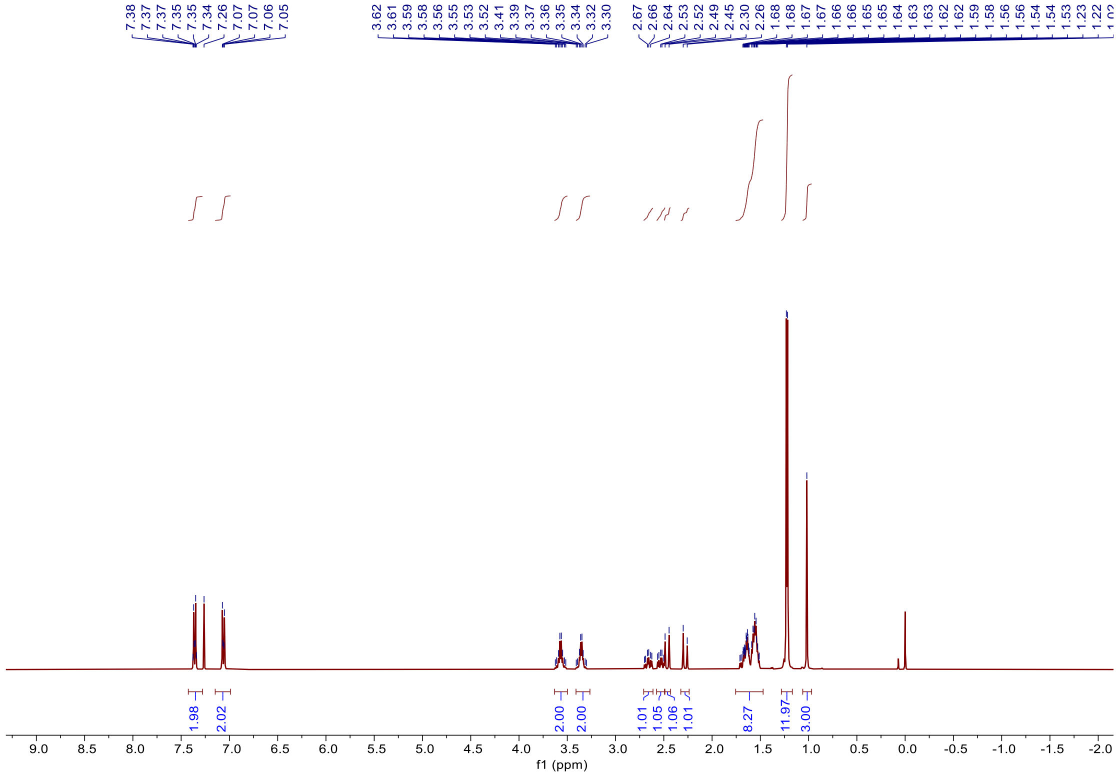

Supplementary Figure 108. ^{1}H NMR (400 MHz, $CDCl_3$) of **3o**.

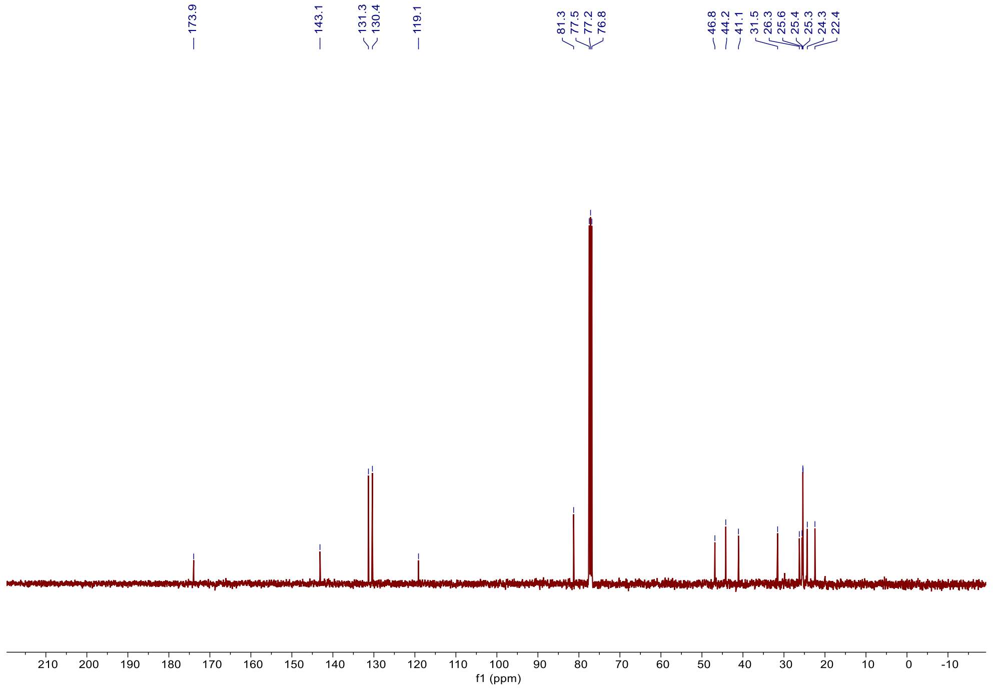

Supplementary Figure 109. ^{13}C NMR (101 MHz, $CDCl_3$) of **3o**.

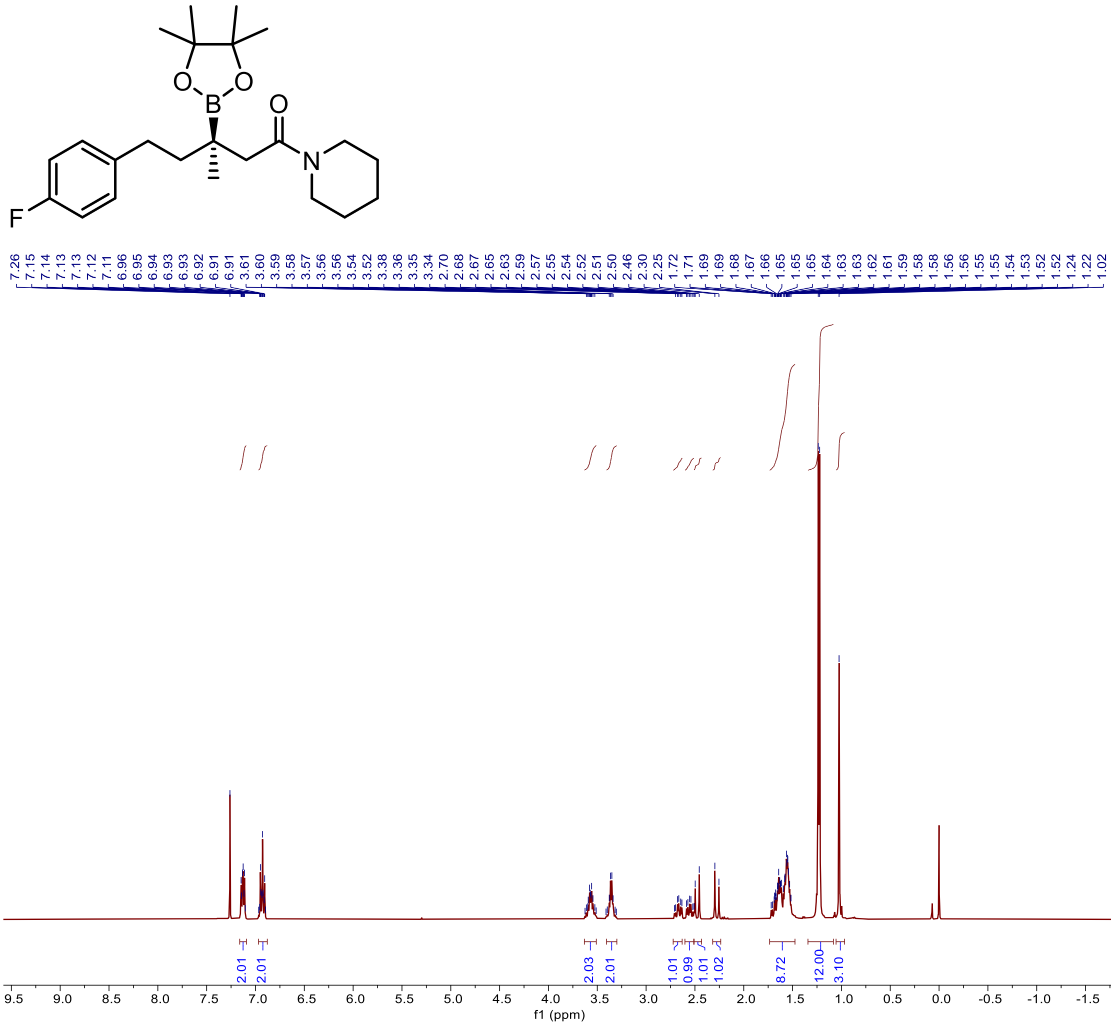

Supplementary Figure 110. ^{1}H NMR (400 MHz, $CDCl_3$) of **3p**.

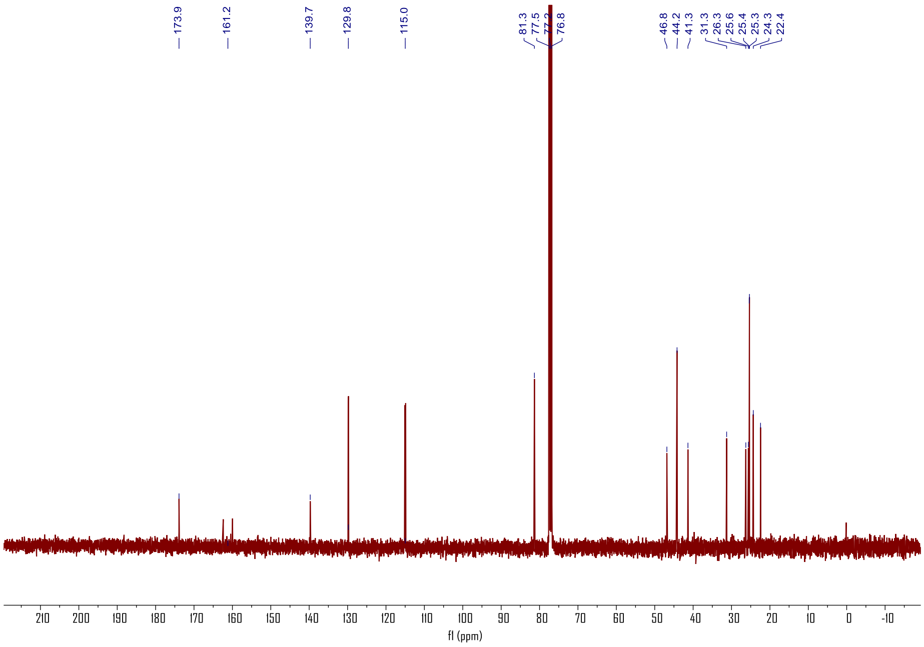

Supplementary Figure 111. ^{13}C NMR (101 MHz, $CDCl_3$) of **3p**.

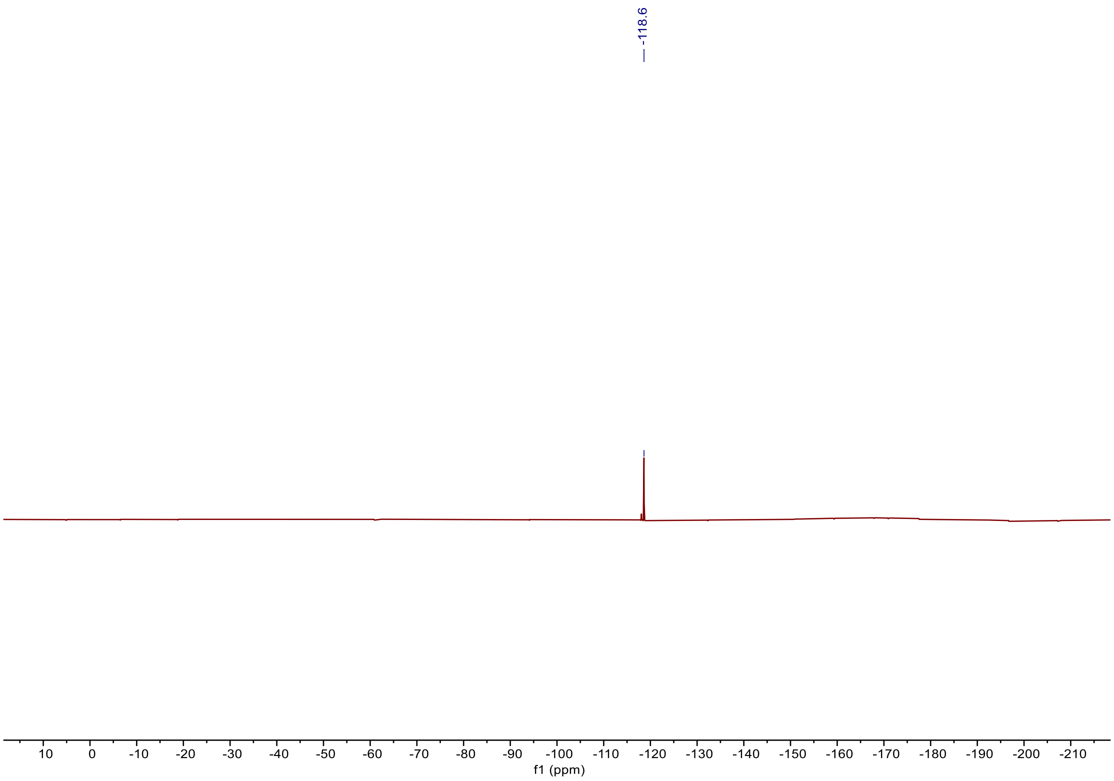

Supplementary Figure 112. ^{19}**F NMR** (376 MHz, $CDCl_3$) of **3p**.

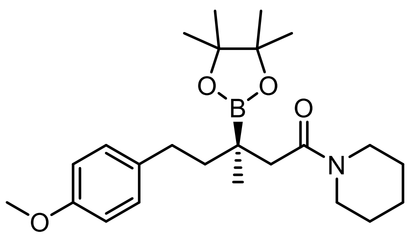

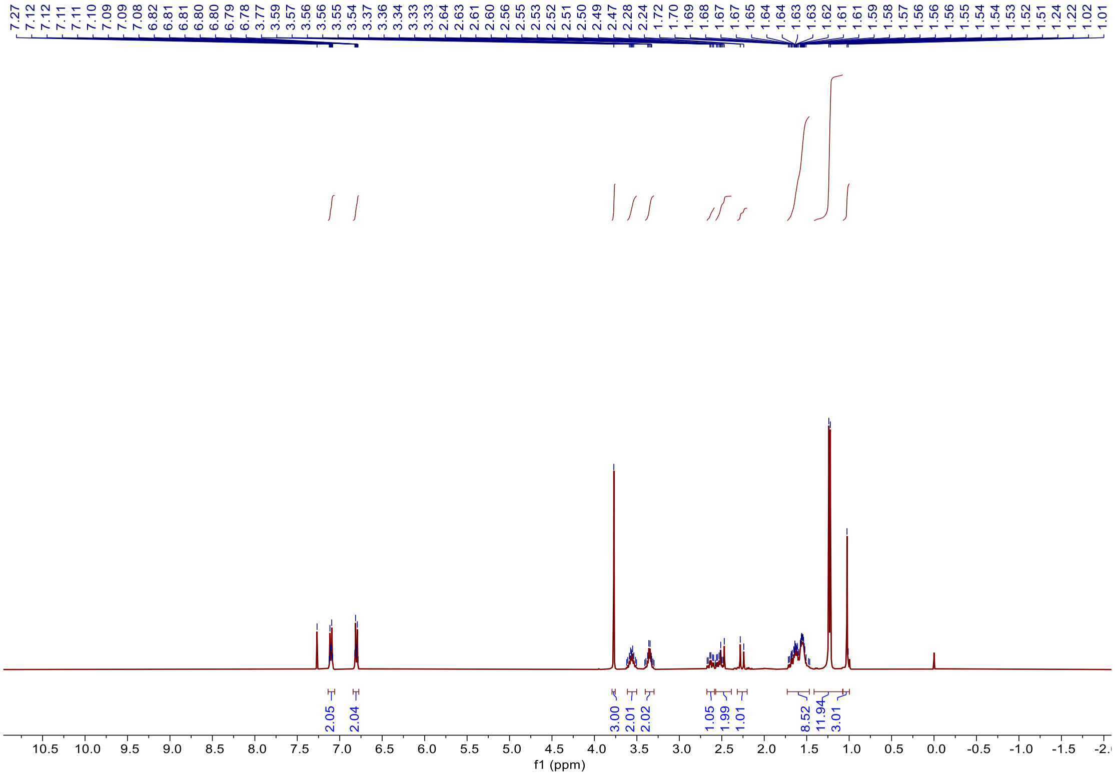

Supplementary Figure 113. ^{1}H NMR (400 MHz, $CDCl_3$) of **3q**.

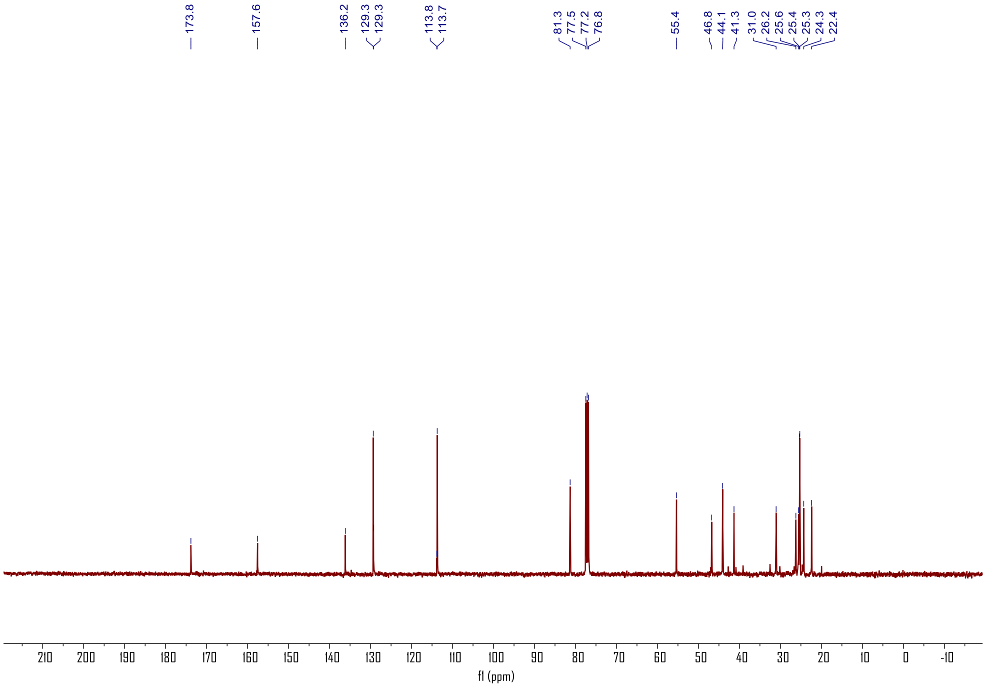

Supplementary Figure 114. ^{13}C NMR (101 MHz, $CDCl_3$) of **3q**.

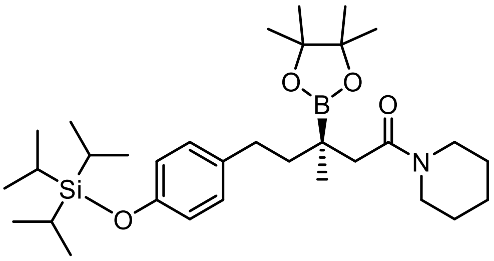

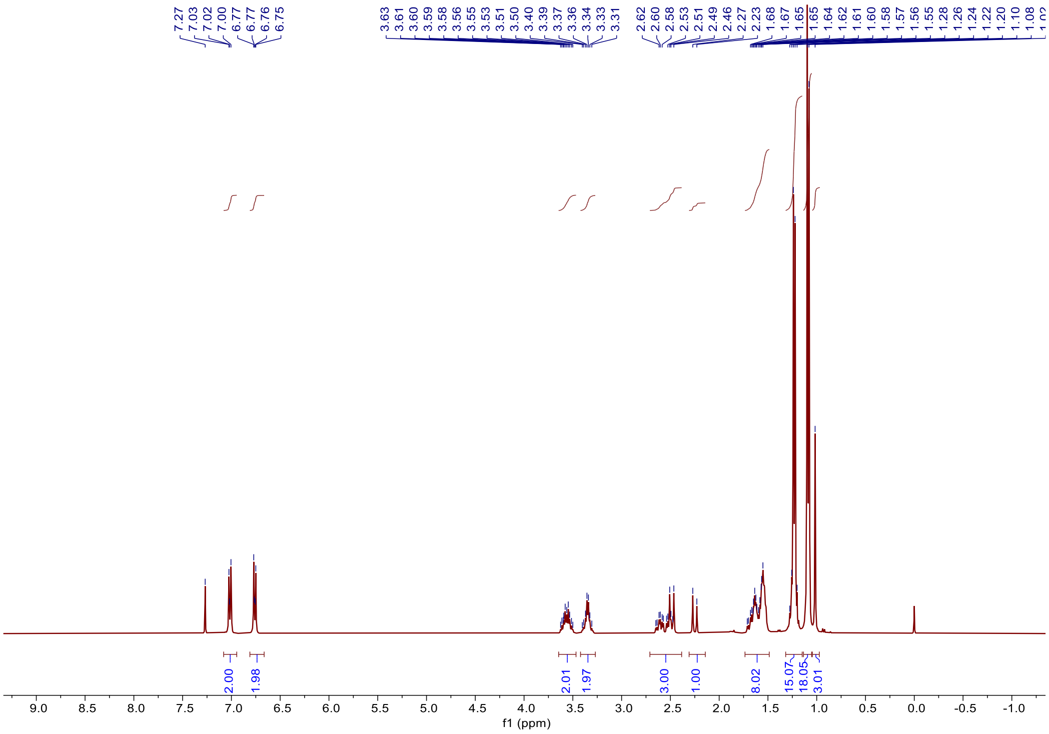

Supplementary Figure 115. ^{1}H NMR (400 MHz, $CDCl_3$) of **3r**.

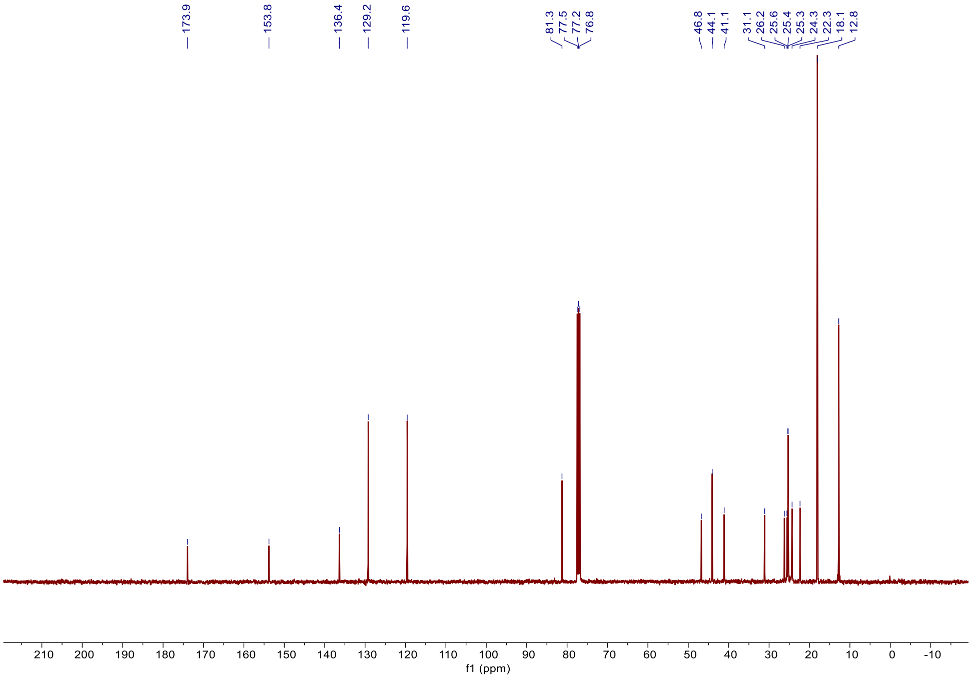

Supplementary Figure 116. ^{13}C NMR (101 MHz, $CDCl_3$) of **3r**.

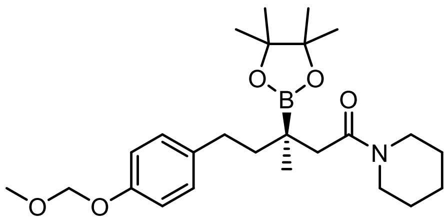

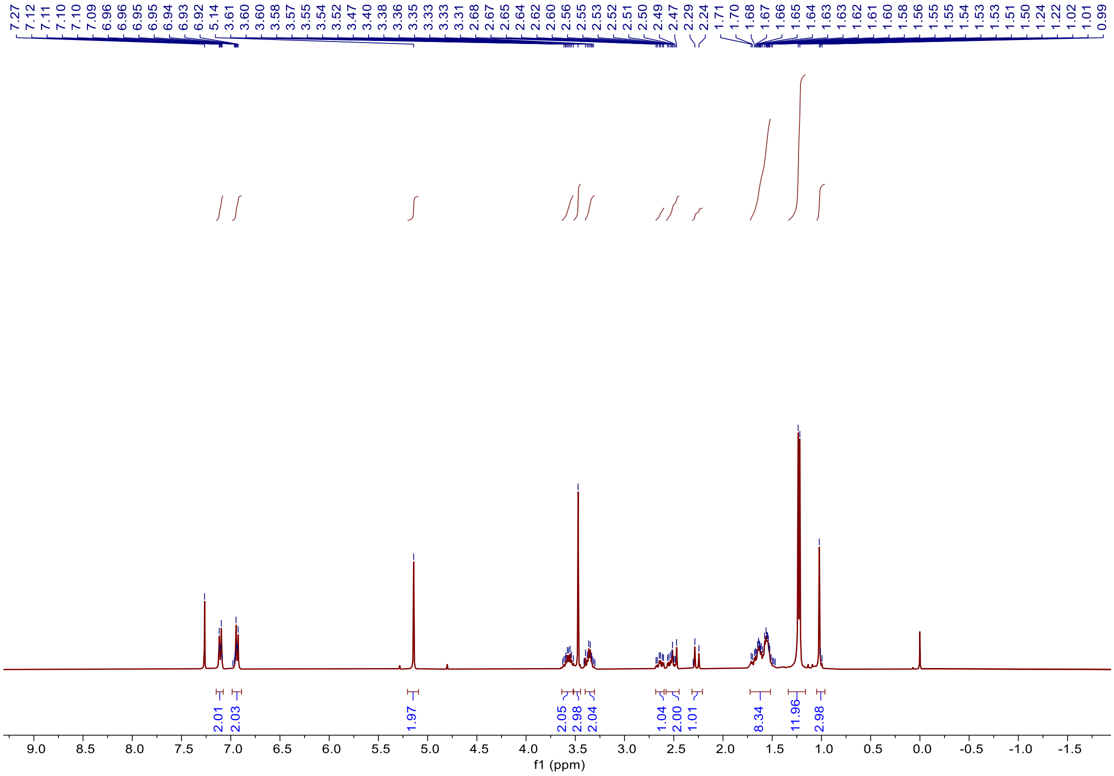

Supplementary Figure 117. ^{1}H NMR (400 MHz, $CDCl_3$) of **3s.**

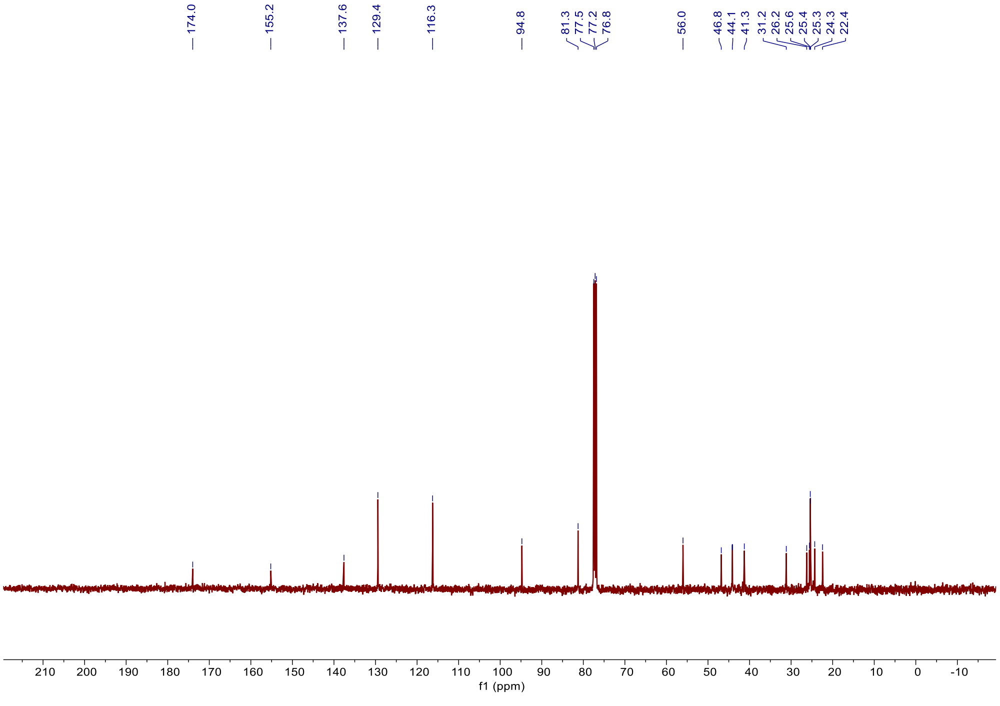

Supplementary Figure 118. ^{13}C NMR (101 MHz, $CDCl_3$) of **3s**.

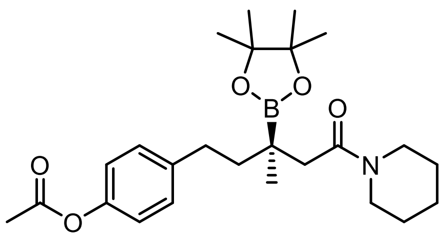

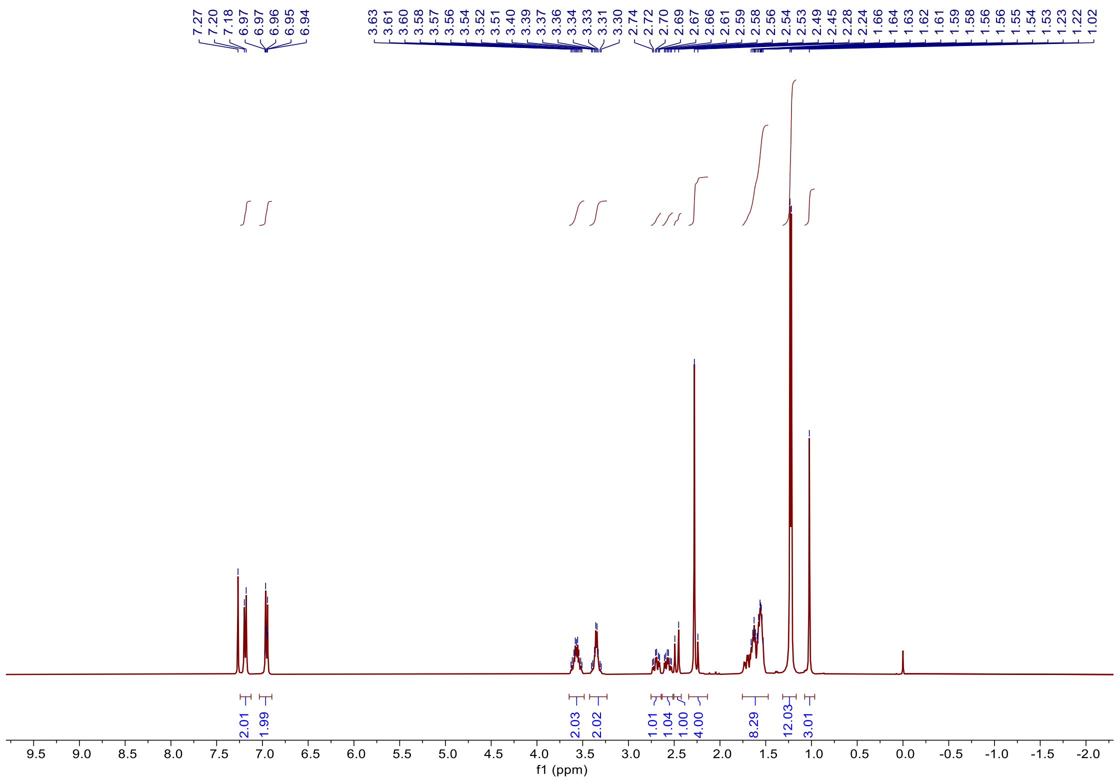

Supplementary Figure 119. ^{1}H NMR (400 MHz, $CDCl_3$) of **3t.**

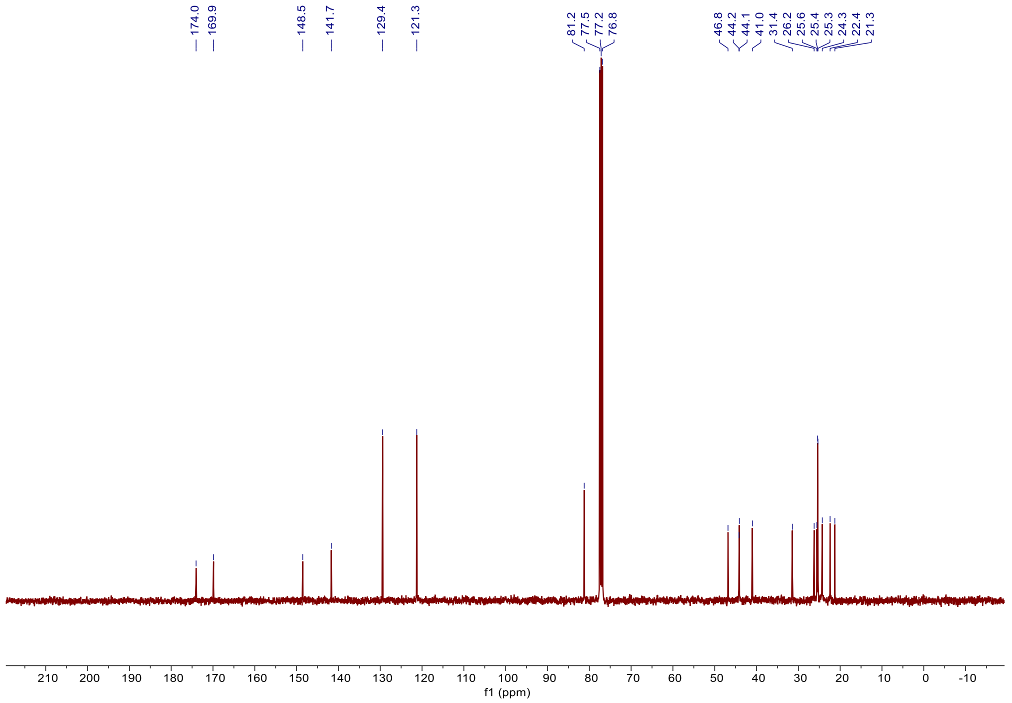

Supplementary Figure 120. ^{13}C NMR (101 MHz, $CDCl_3$) of **3t**:

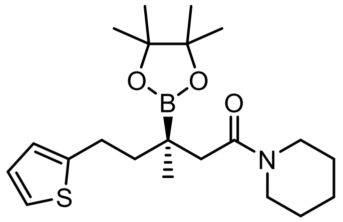

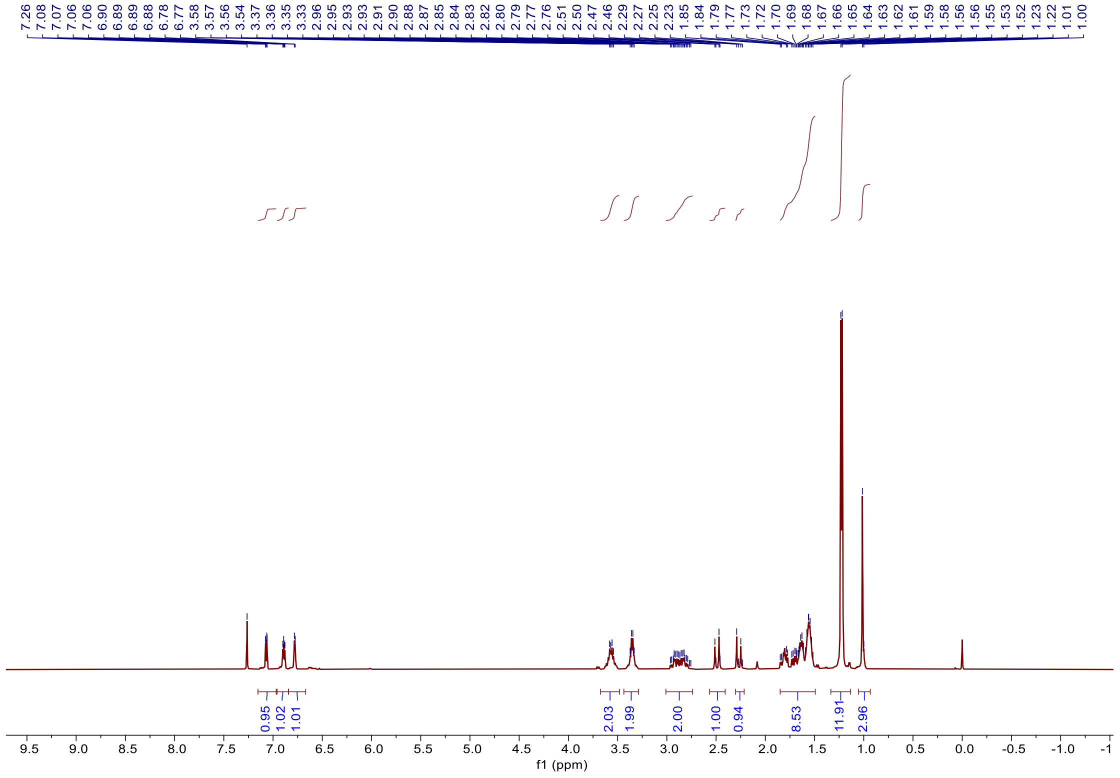

Supplementary Figure 121. ^{1}H NMR (400 MHz, $CDCl_3$) of **3u.**

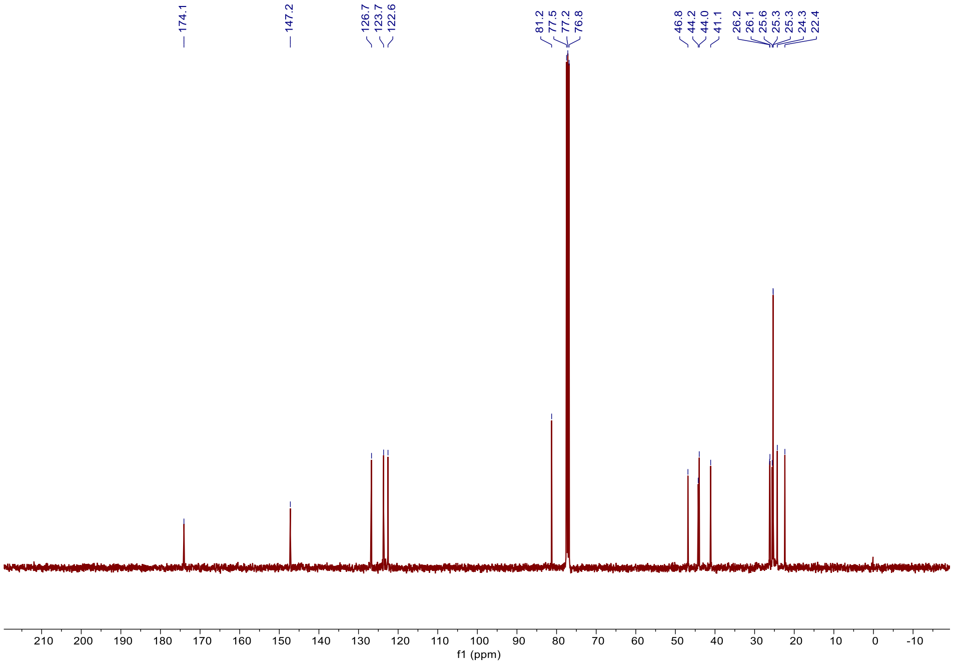

Supplementary Figure 122. ^{13}C NMR (101 MHz, $CDCl_3$) of **3u**.

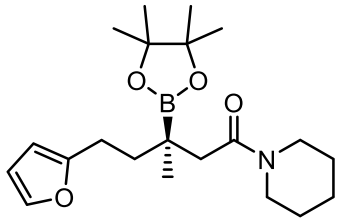

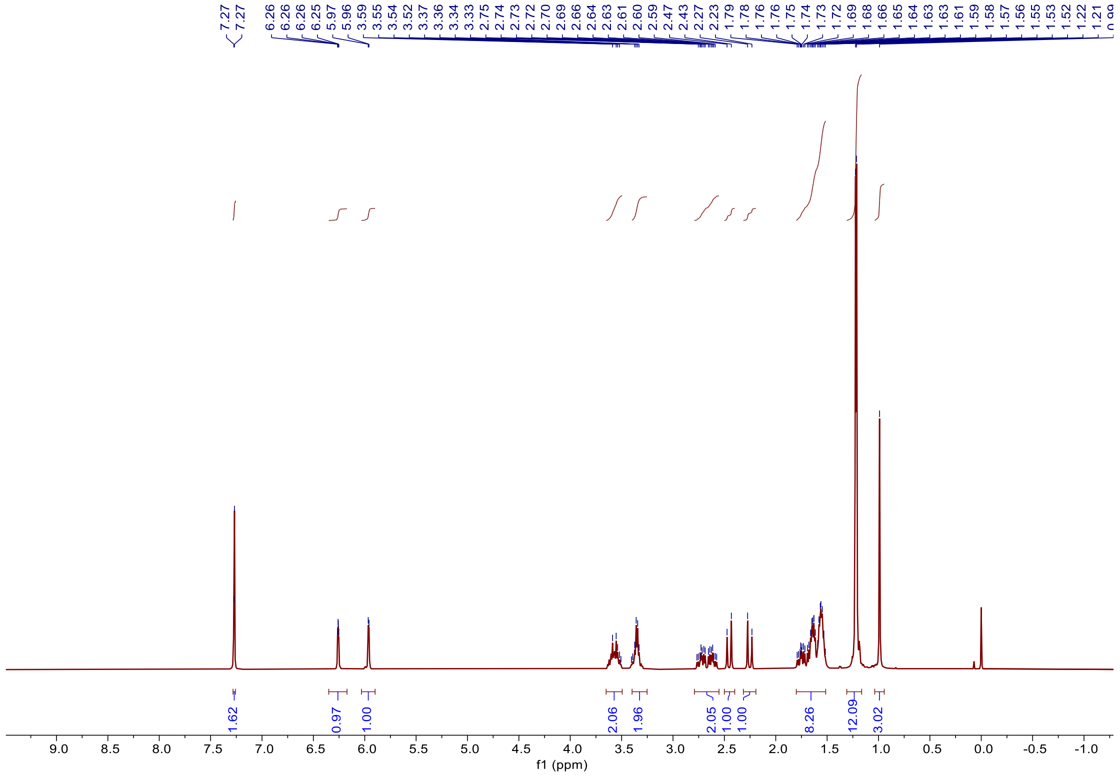

Supplementary Figure 123. ^{1}H NMR (400 MHz, $CDCl_3$) of **3v.**

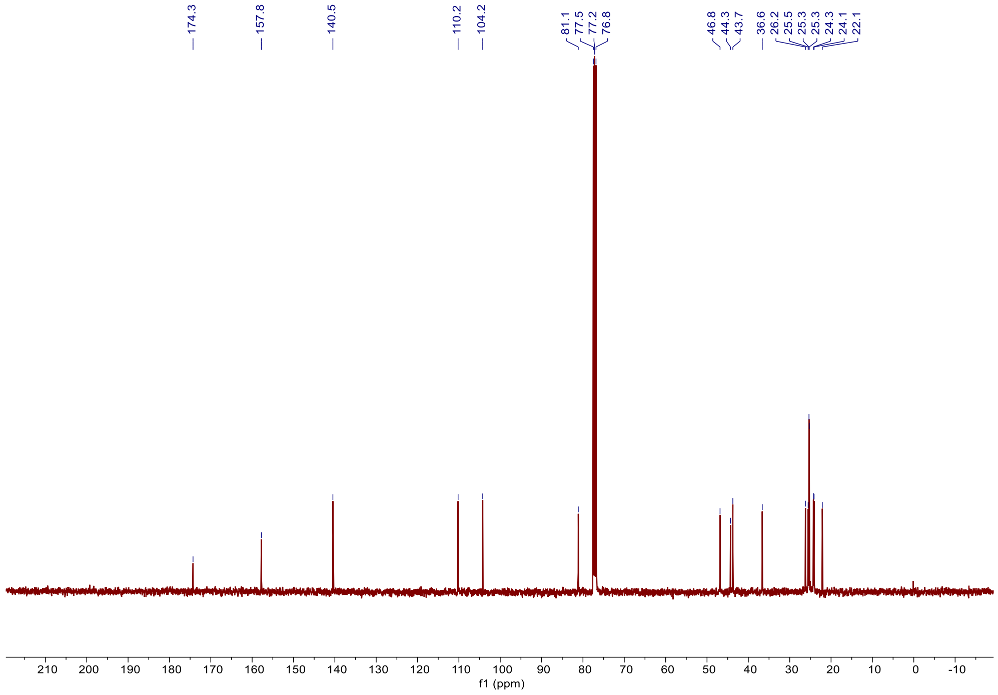

Supplementary Figure 124. ^{13}C NMR (101 MHz, $CDCl_3$) of **3v**.

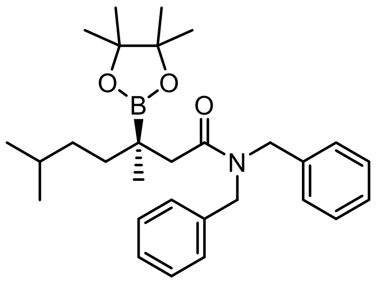

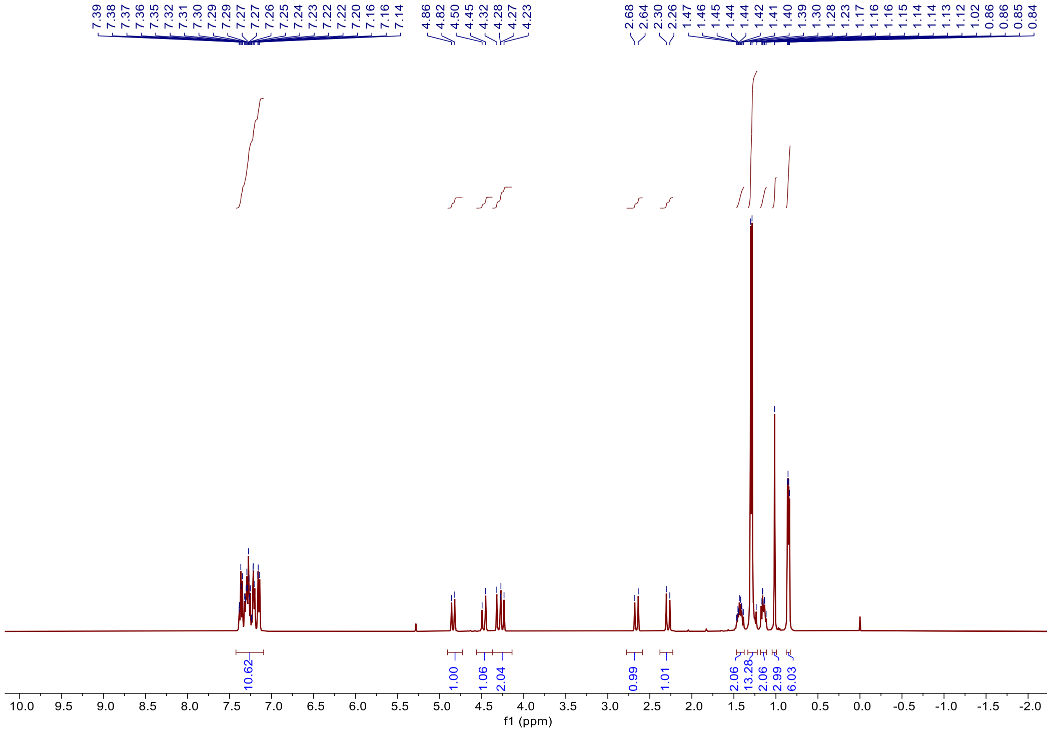

Supplementary Figure 125. ^{1}H NMR (400 MHz, $CDCl_3$) of **3w.**

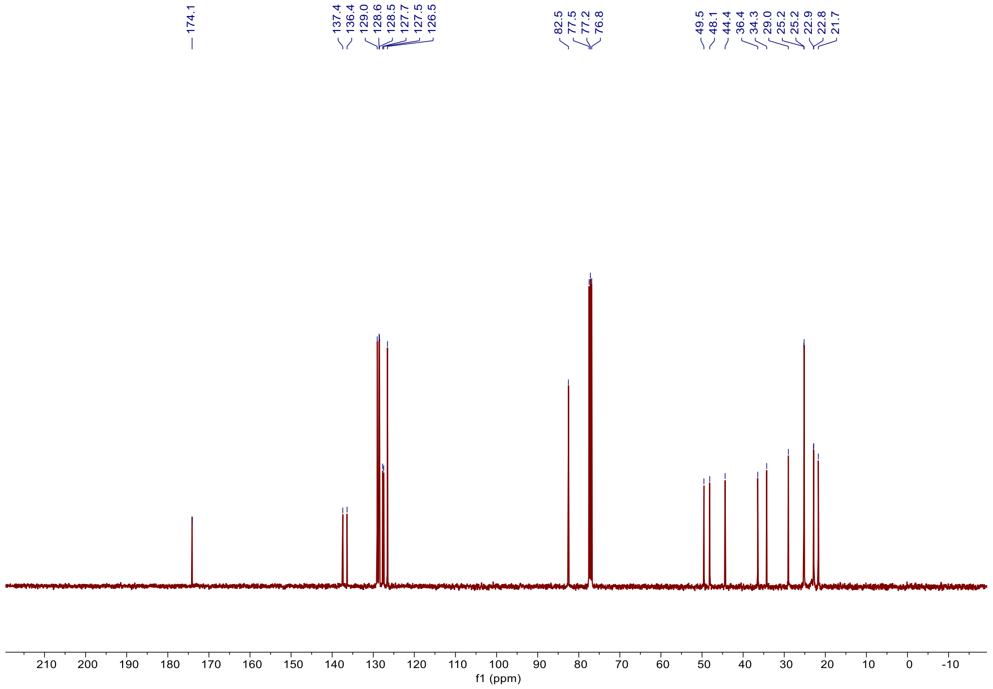

Supplementary Figure 126. ^{13}C NMR (101 MHz, $CDCl_3$) of **3w**.

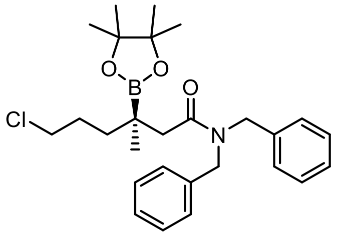

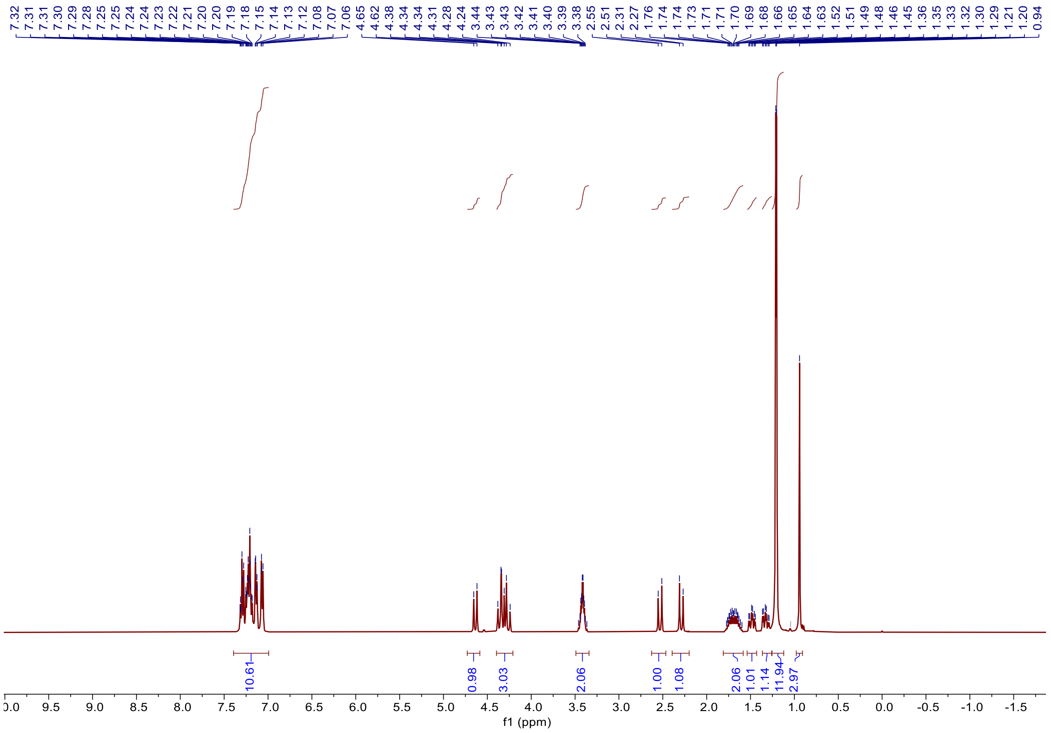

Supplementary Figure 127. ^{1}H NMR (400 MHz, $CDCl_3$) of **3x.**

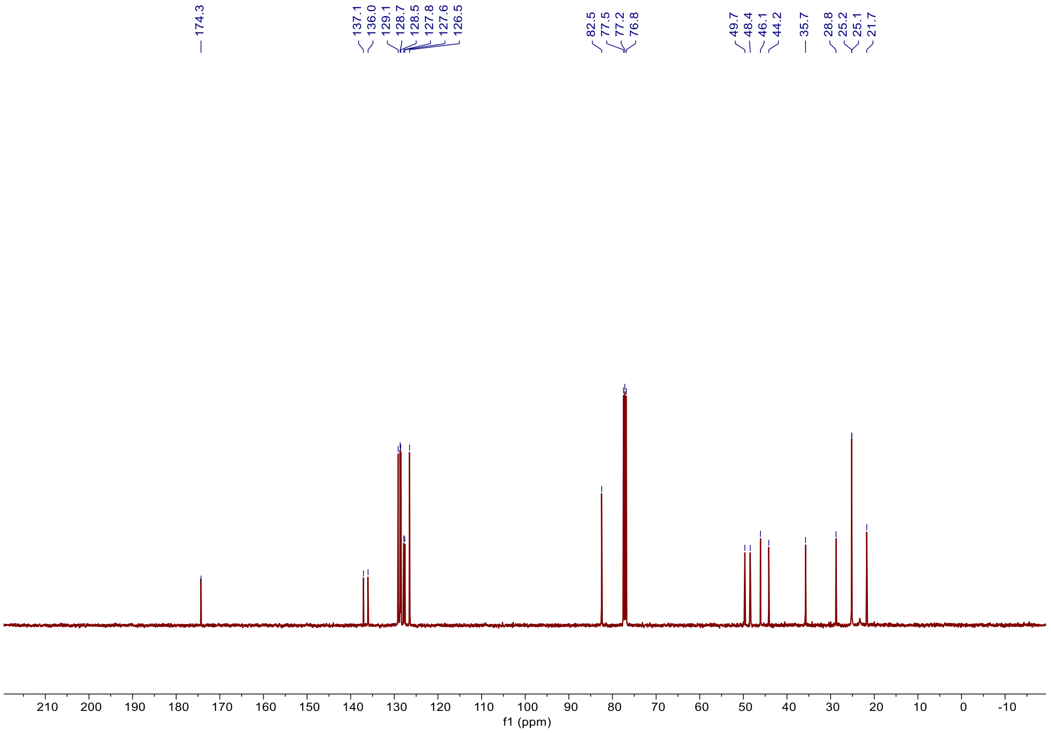

Supplementary Figure 128. ^{13}C NMR (101 MHz, $CDCl_3$) of **3x**.

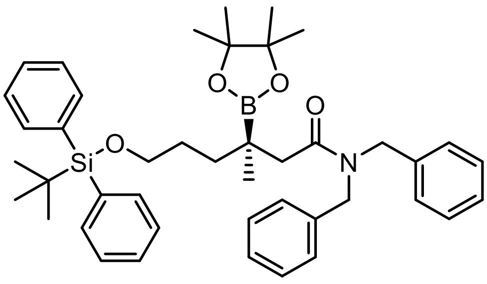

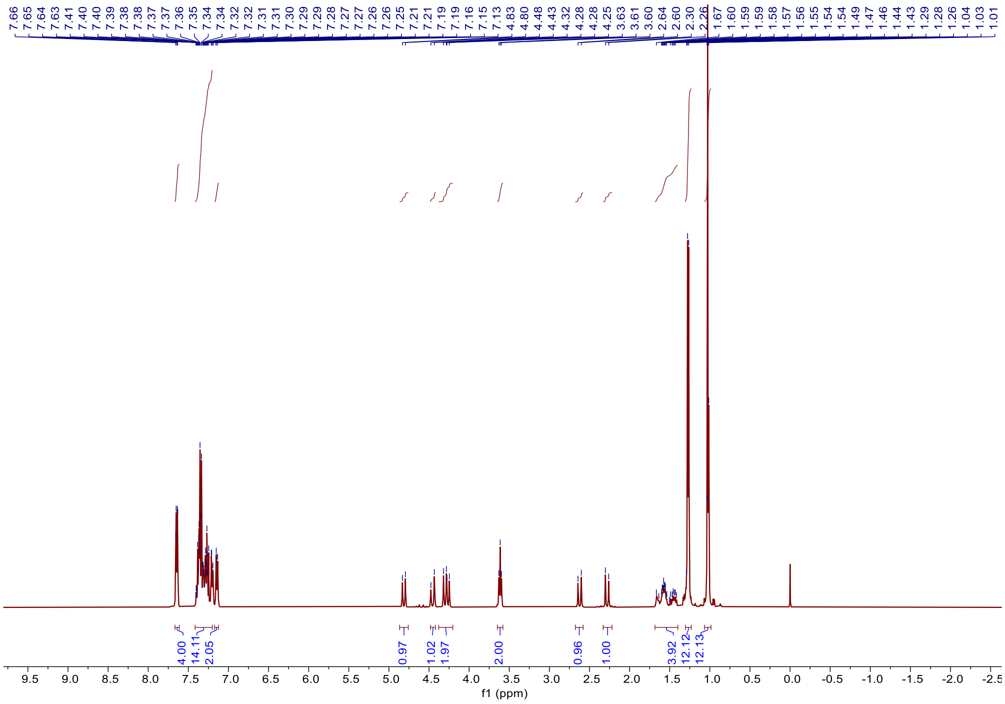

Supplementary Figure 129. ^{1}H NMR (400 MHz, $CDCl_3$) of **3y.**

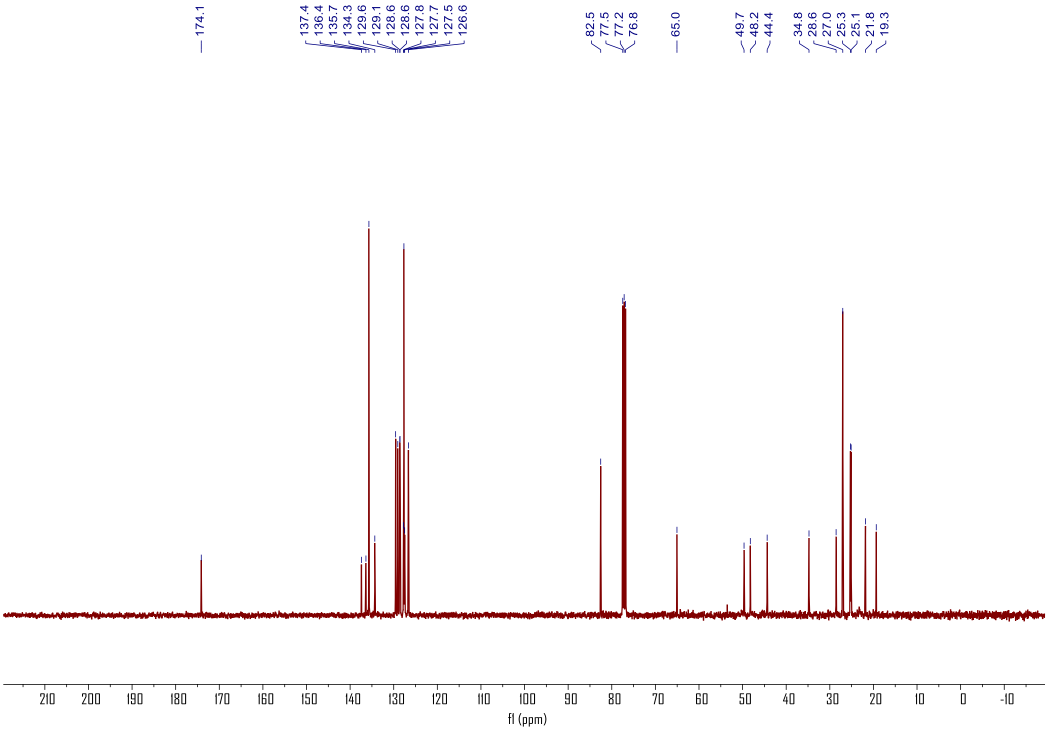

Supplementary Figure 130. ^{13}C NMR (101 MHz, $CDCl_3$) of **3y**.

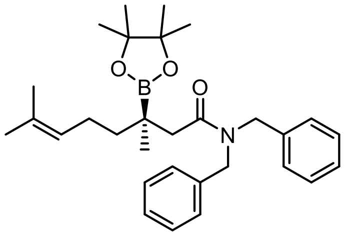

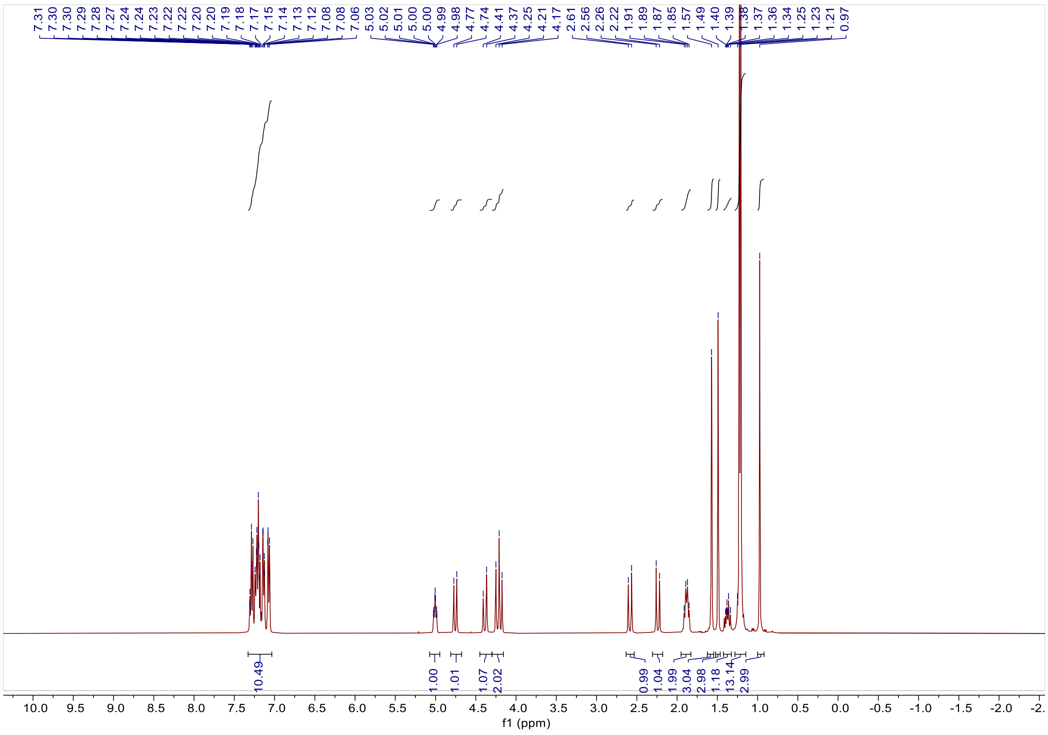

Supplementary Figure 131. ^{1}H NMR (400 MHz, $CDCl_3$) of **3z.**

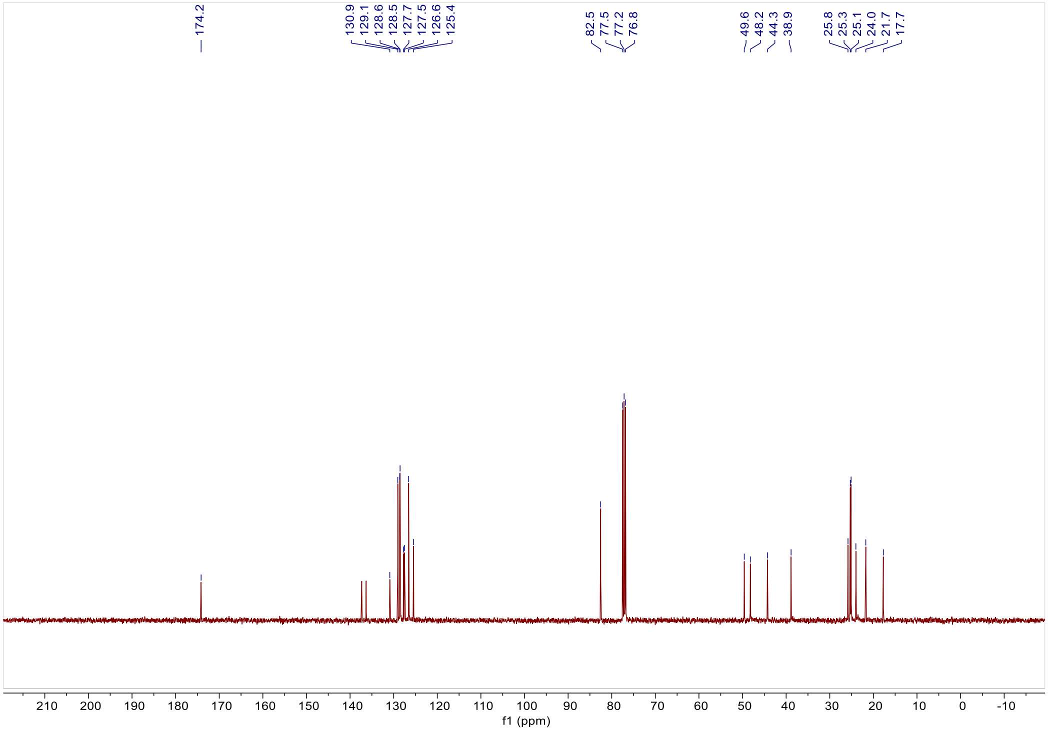

Supplementary Figure 132. ^{13}C NMR (101 MHz, $CDCl_3$) of **3z**.

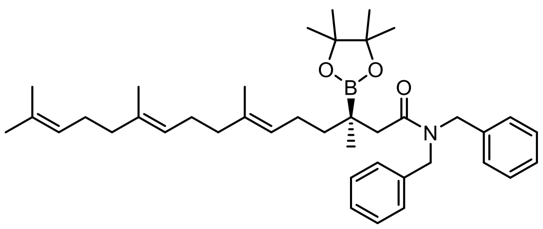

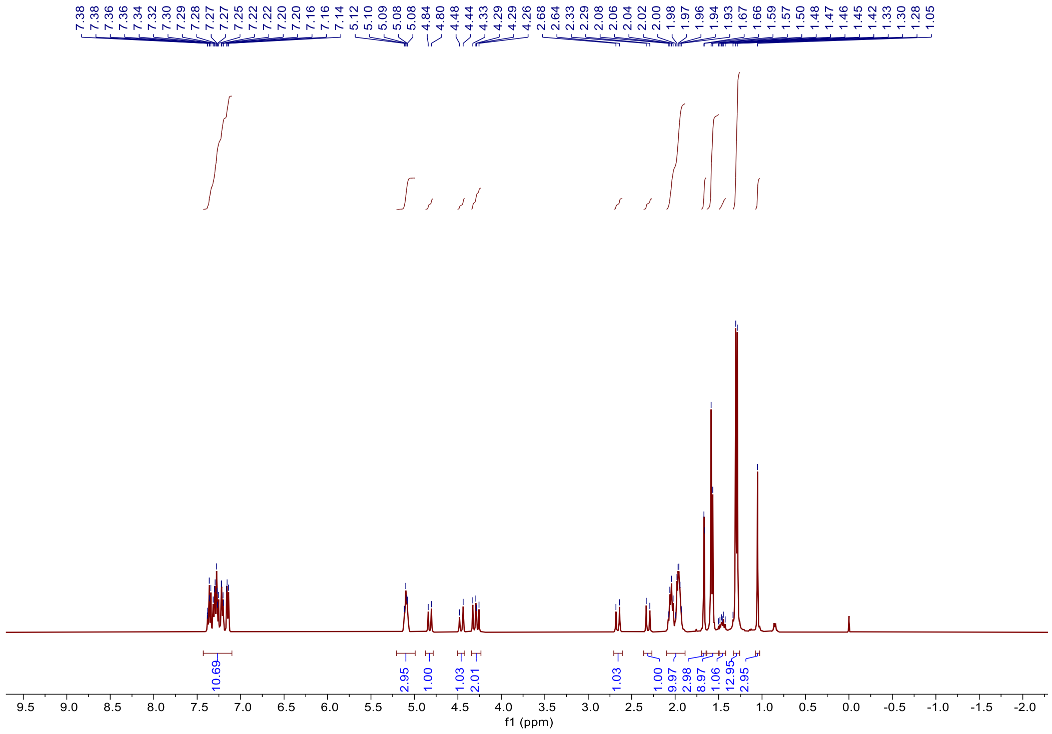

Supplementary Figure 133. ^{1}H NMR (400 MHz, $CDCl_3$) of **3aa.**

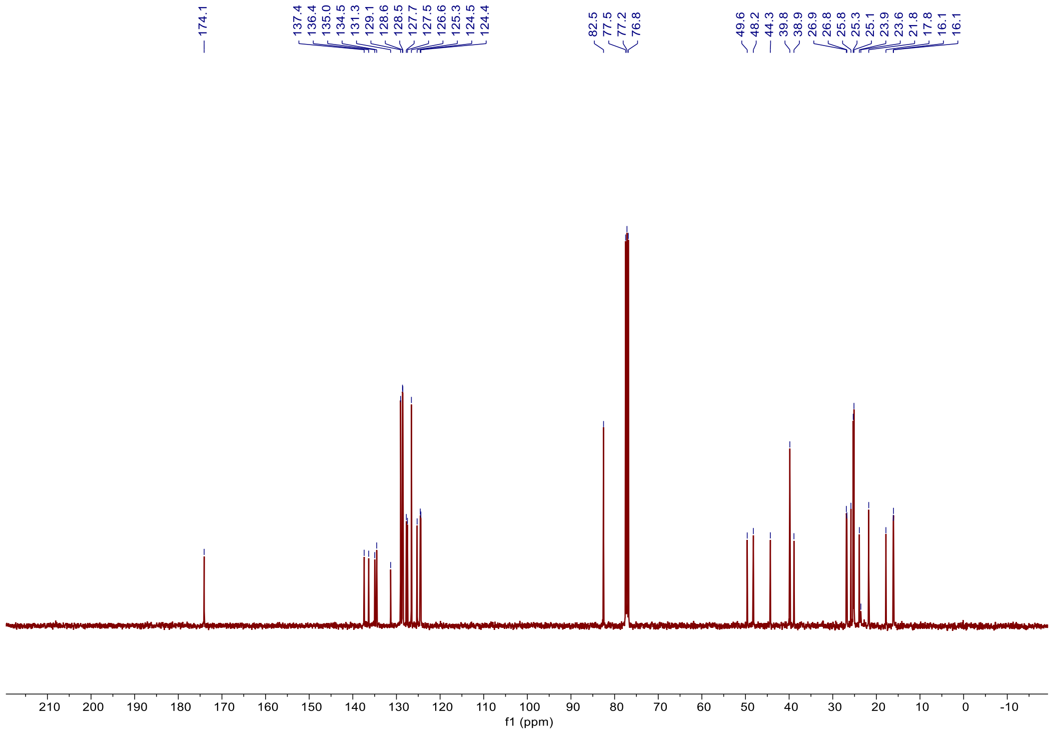

Supplementary Figure 134. ^{13}C NMR (101 MHz, $CDCl_3$) of **3aa**.

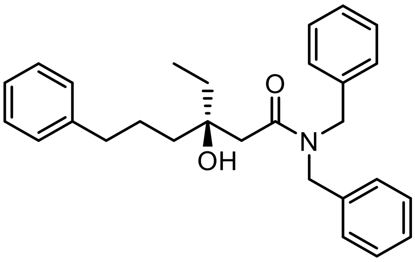

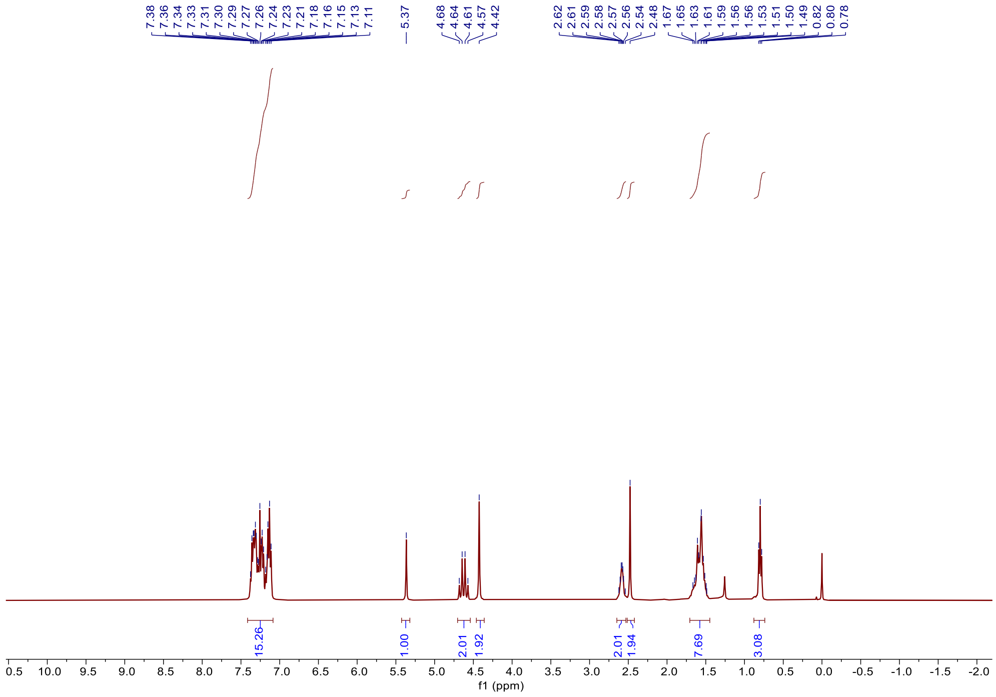

Supplementary Figure 135. ^{1}H NMR (400 MHz, $CDCl_3$) of **3ab-ol.**

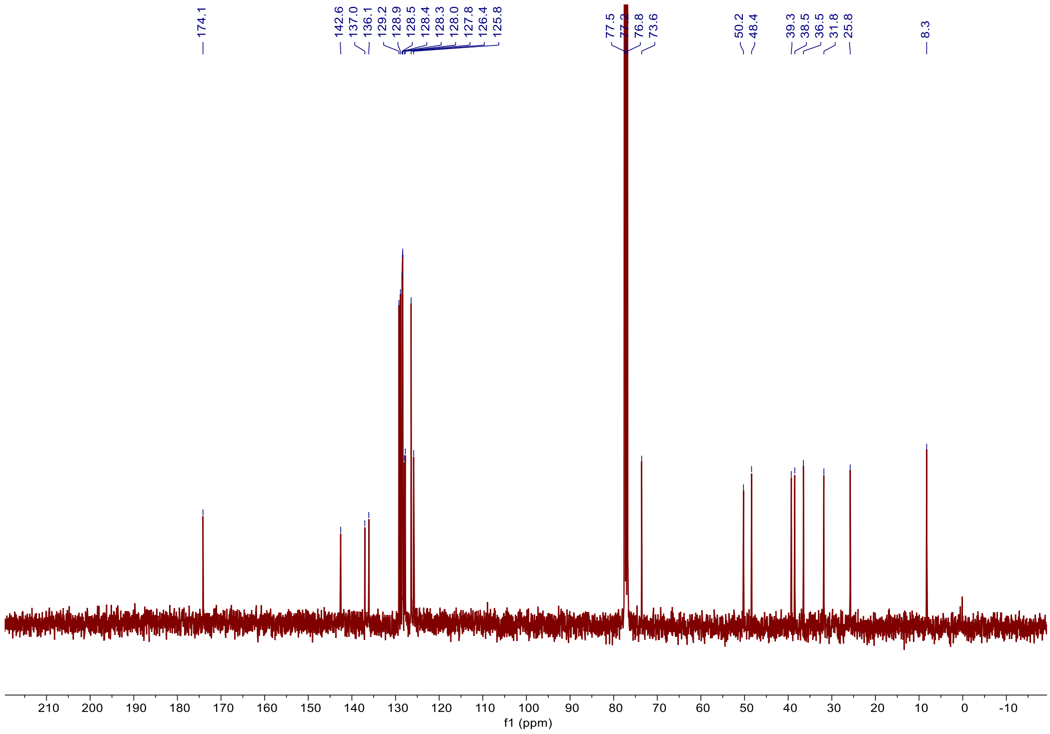

Supplementary Figure 136. ^{13}C NMR (101 MHz, $CDCl_3$) of **3ab-ol**.

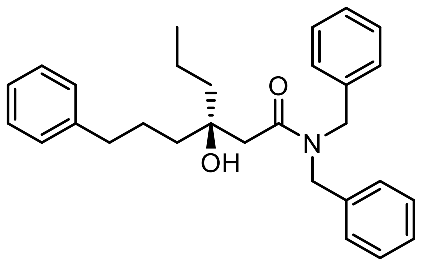

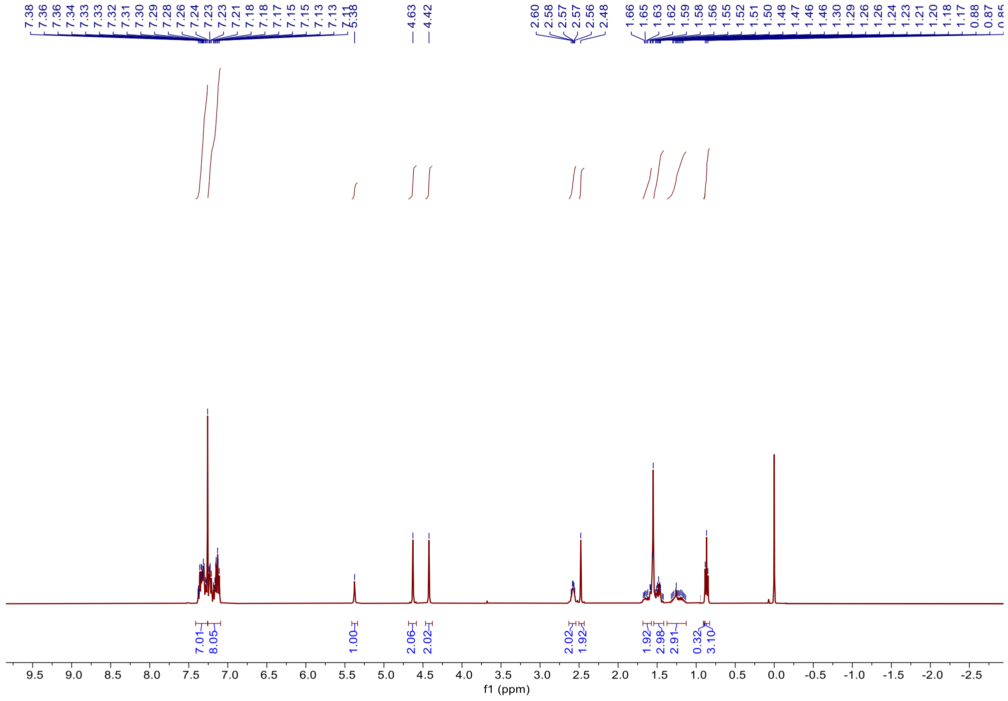

Supplementary Figure 137. ^{1}H NMR (400 MHz, $CDCl_3$) of **3ac-ol.**

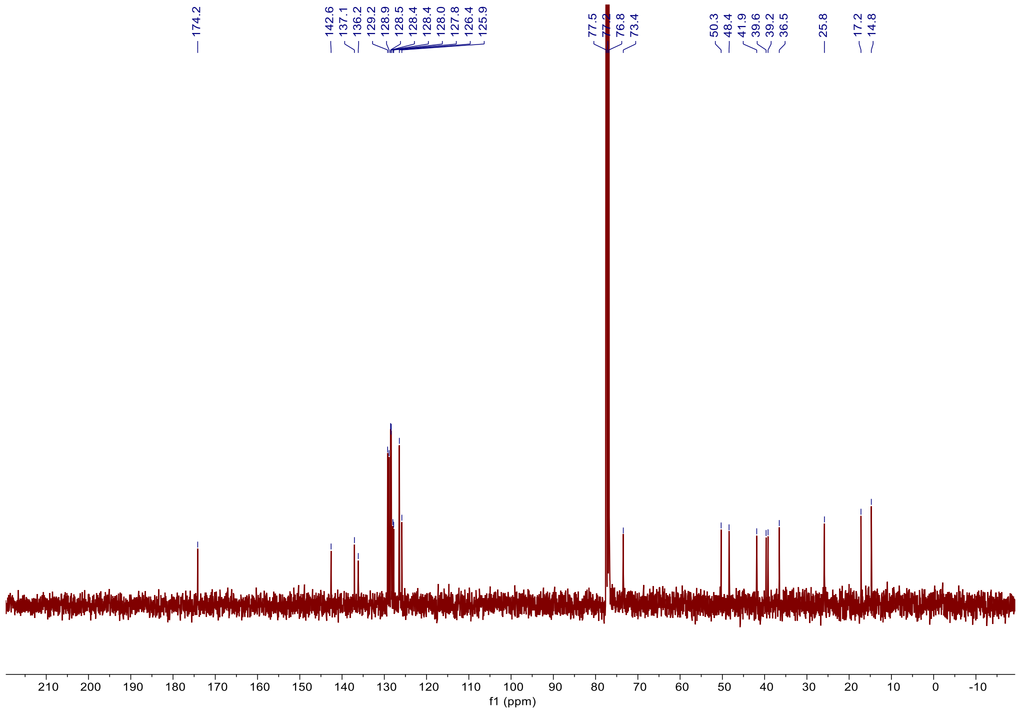

Supplementary Figure 138. ^{13}C NMR (101 MHz, $CDCl_3$) of **3ac-ol**.

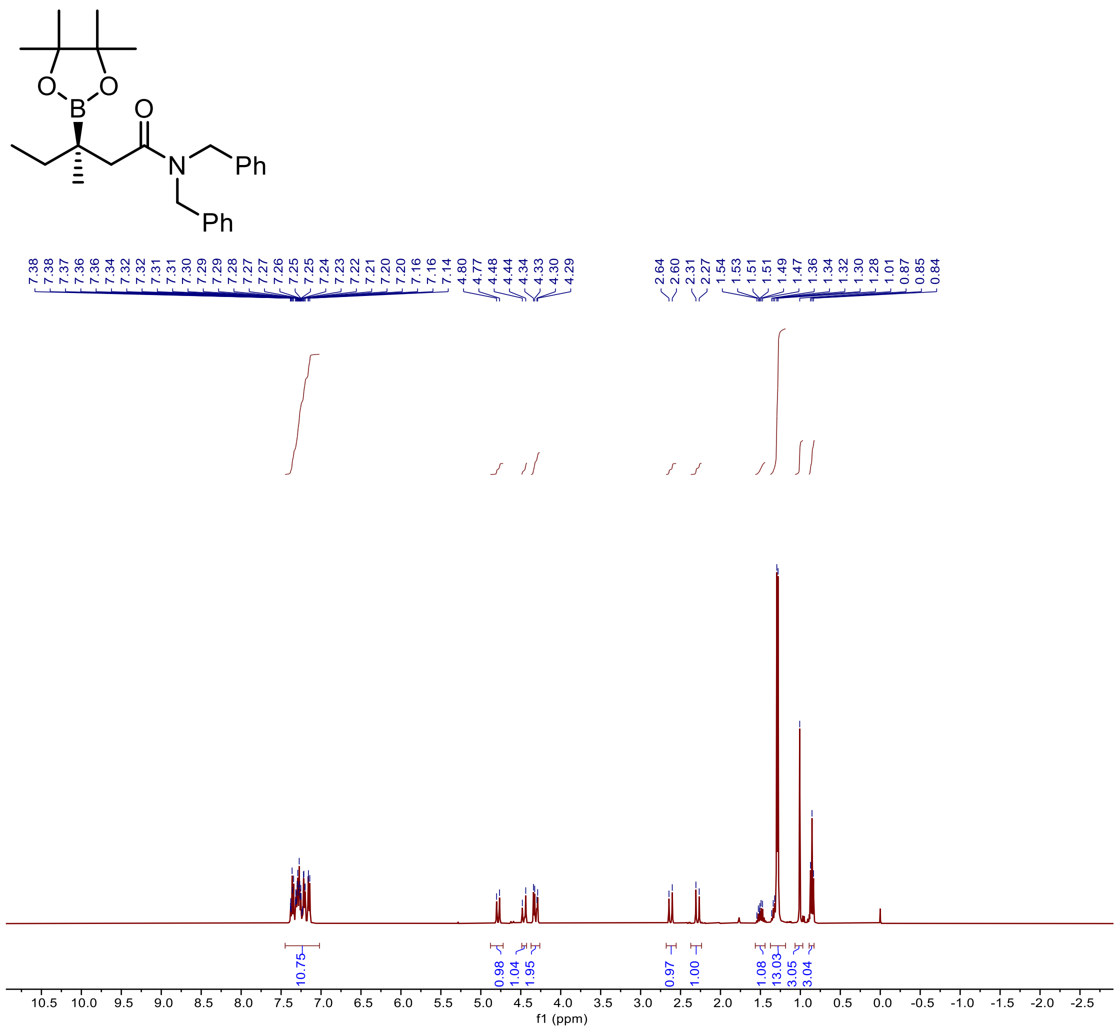

Supplementary Figure 139. ^{1}H NMR (400 MHz, $CDCl_3$) of **3ad**.

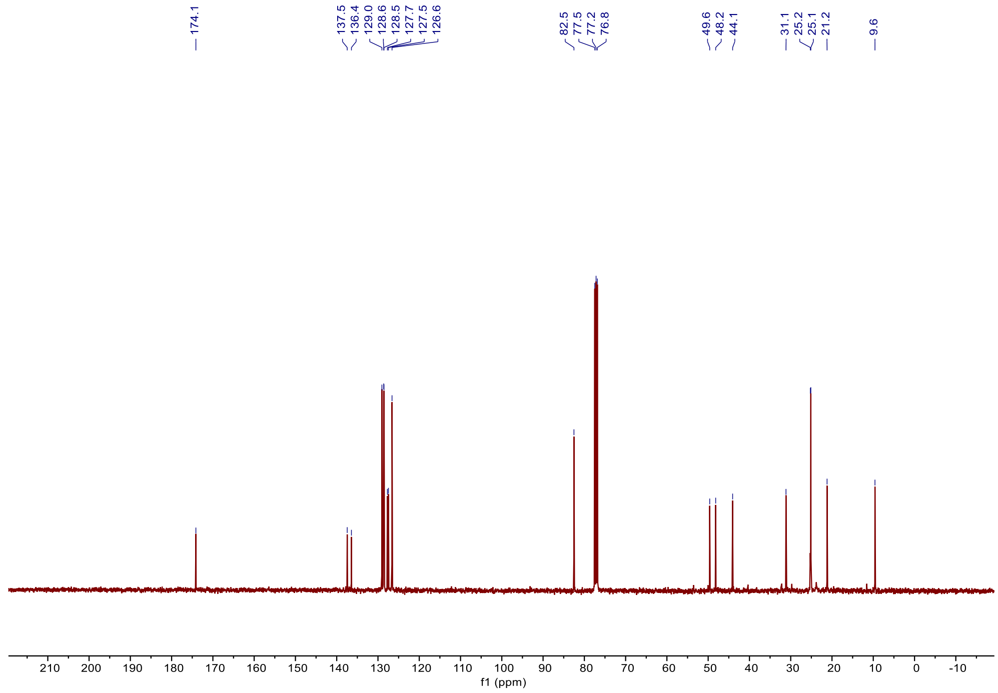

Supplementary Figure 140. ^{13}C NMR (101 MHz, $CDCl_3$) of **3ad**.

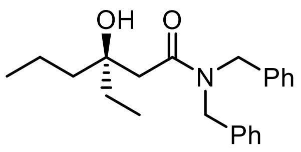

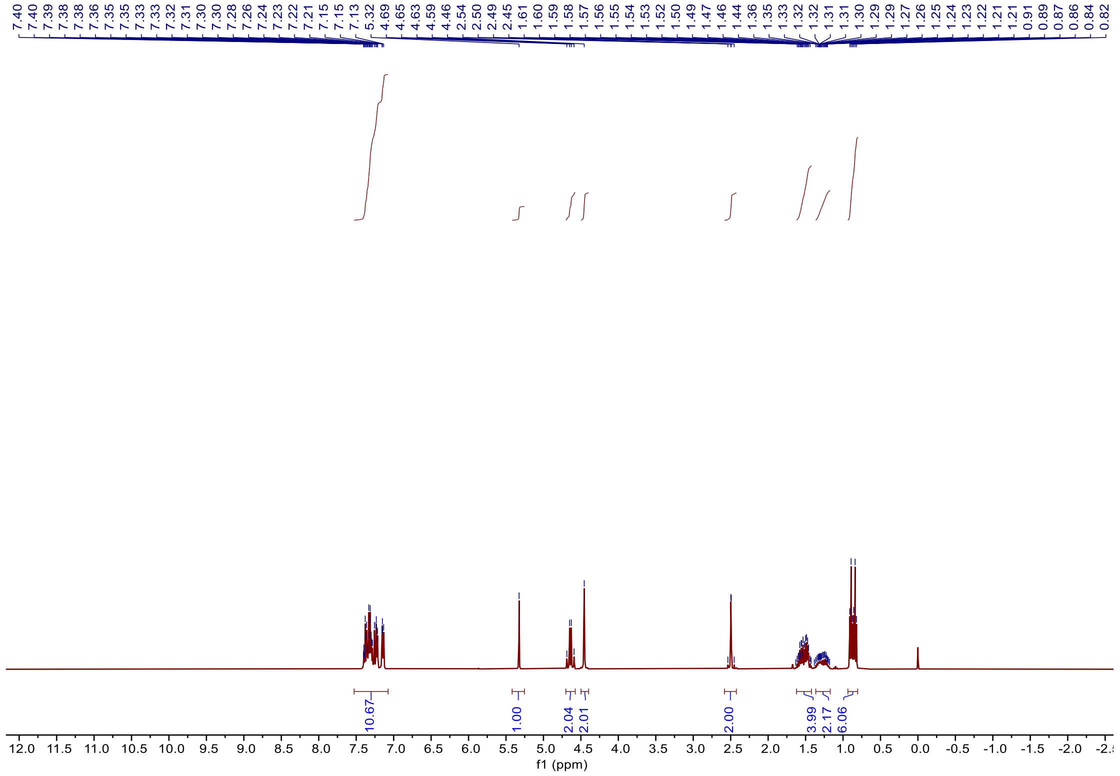

Supplementary Figure 141. ^{1}H NMR (400 MHz, $CDCl_3$) of **3ae-ol**.

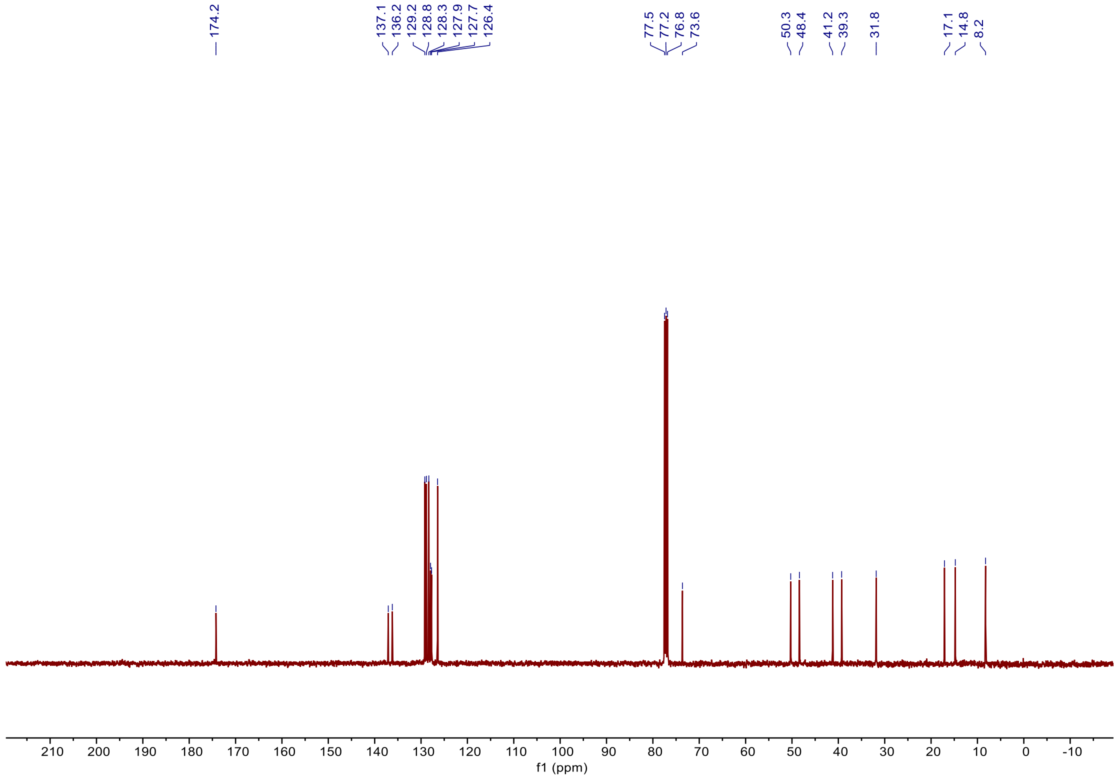

Supplementary Figure 142. ^{13}C NMR (101 MHz, $CDCl_3$) of **3ae-ol**.

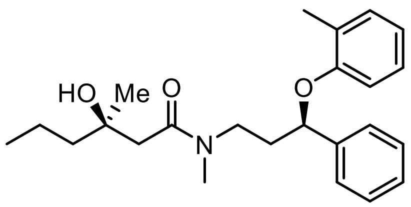

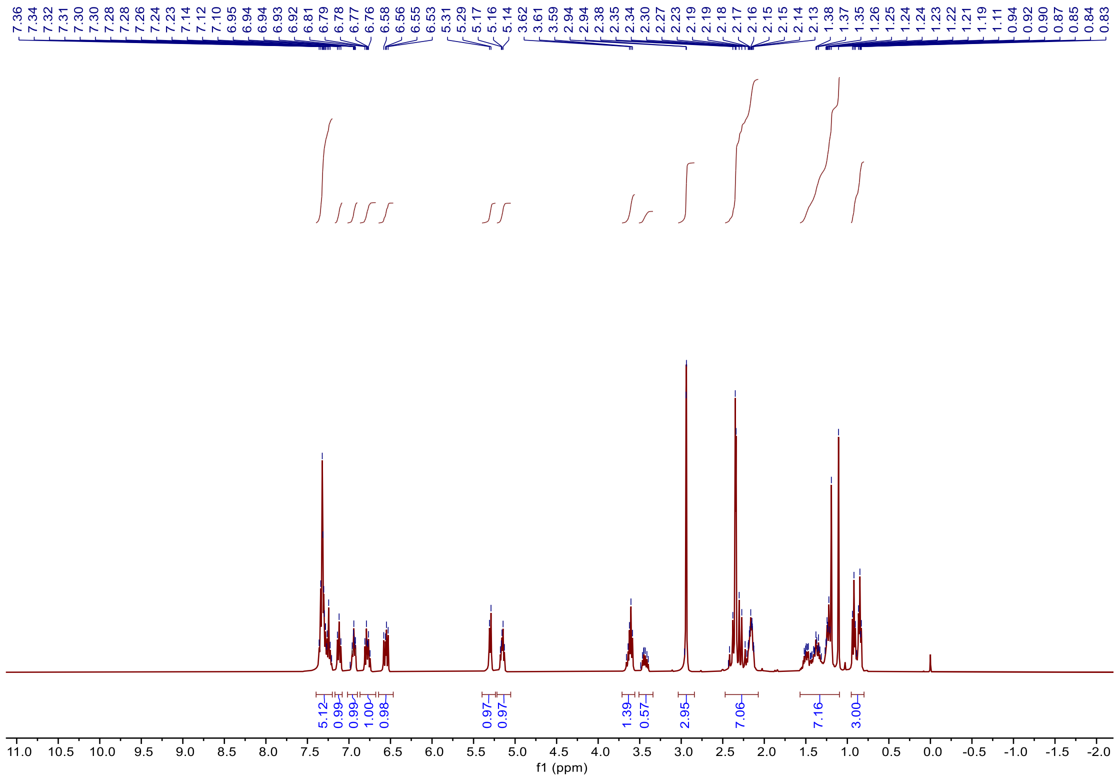

Supplementary Figure 143. ^{1}H NMR (400 MHz, $CDCl_3$) of **3ah-ol.**

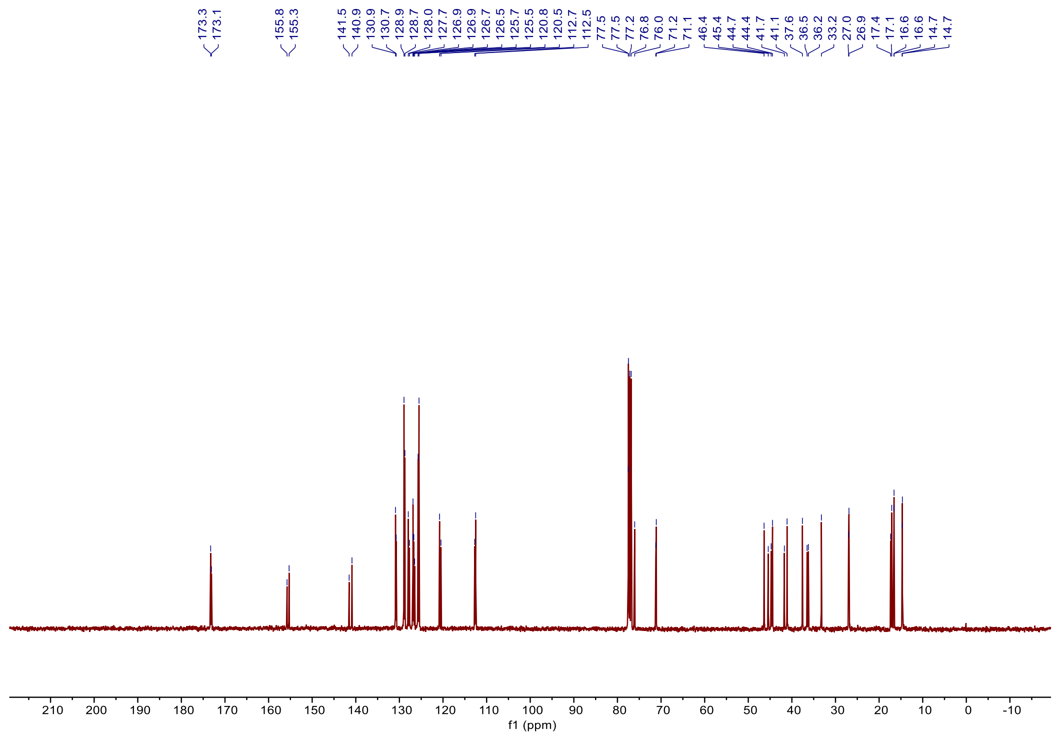

Supplementary Figure 144. ^{13}C NMR (101 MHz, $CDCl_3$) of **3ah-ol**.

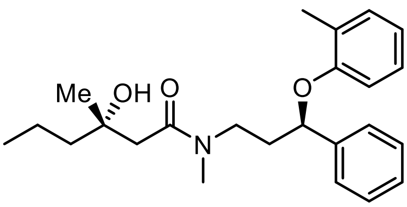

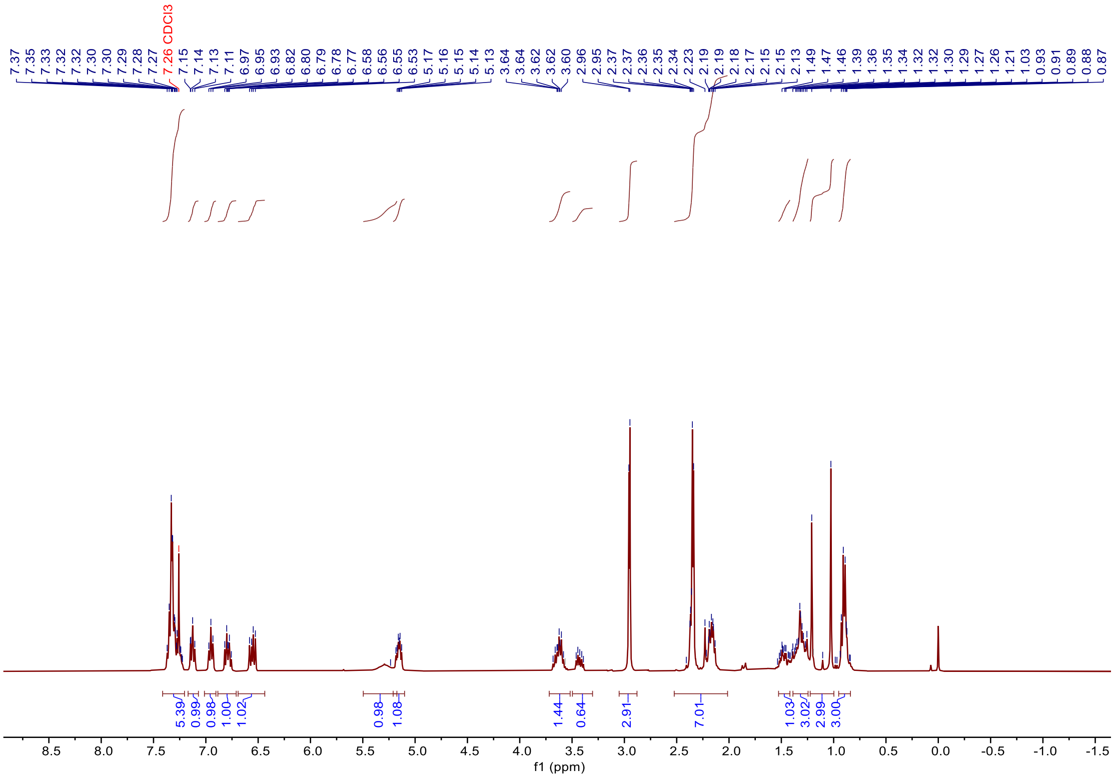

Supplementary Figure 145. ^{1}H NMR (400 MHz, $CDCl_3$) of **3ak-ol.**

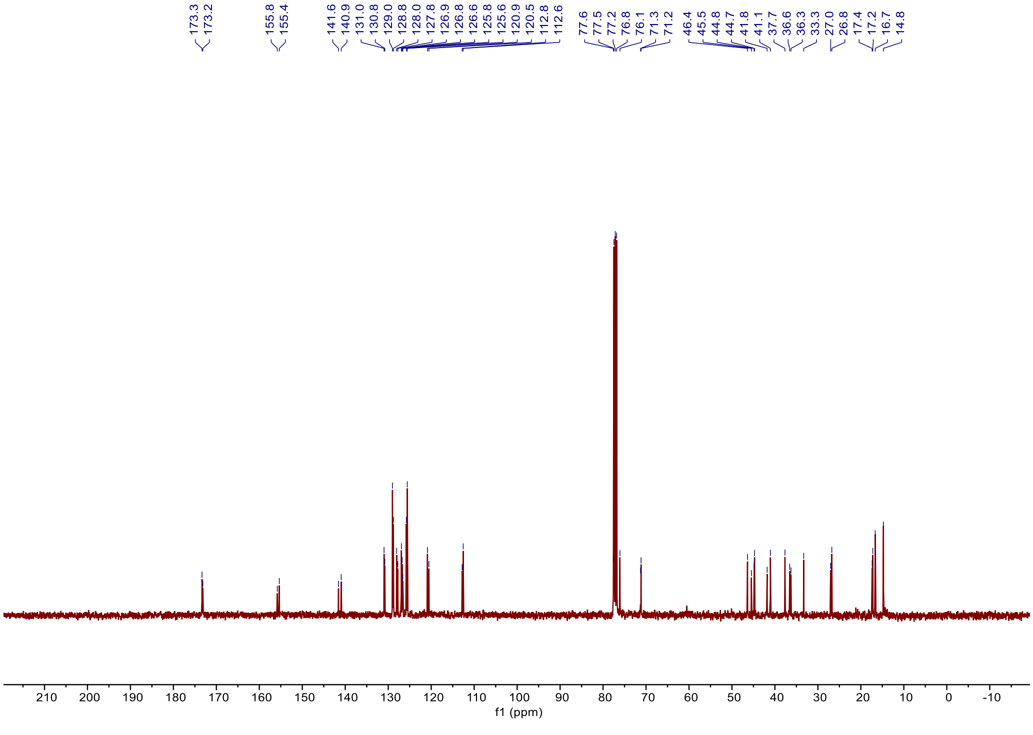

Supplementary Figure 146. ^{13}C NMR (101 MHz, $CDCl_3$) of **3ak-ol**.

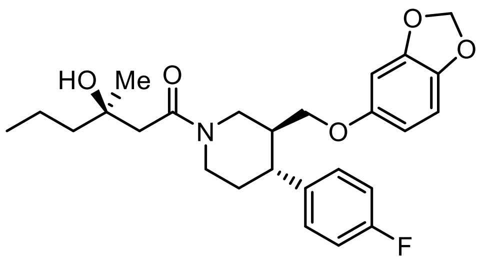

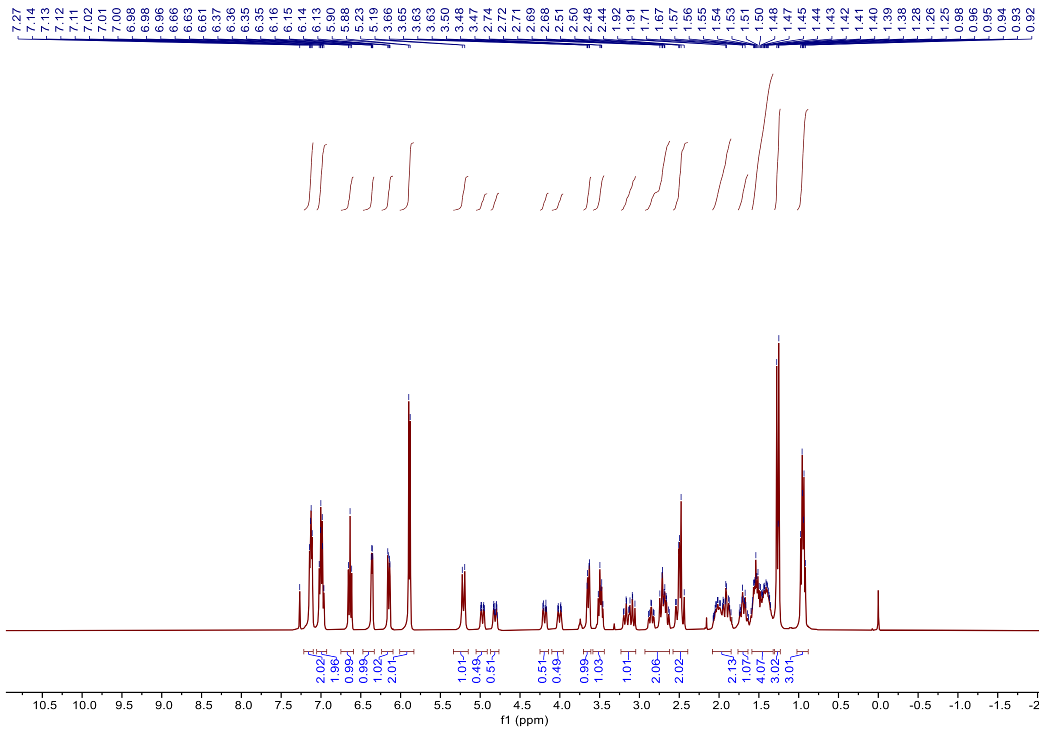

Supplementary Figure 147. ^{1}H NMR (400 MHz, $CDCl_3$) of **3ai-ol**.

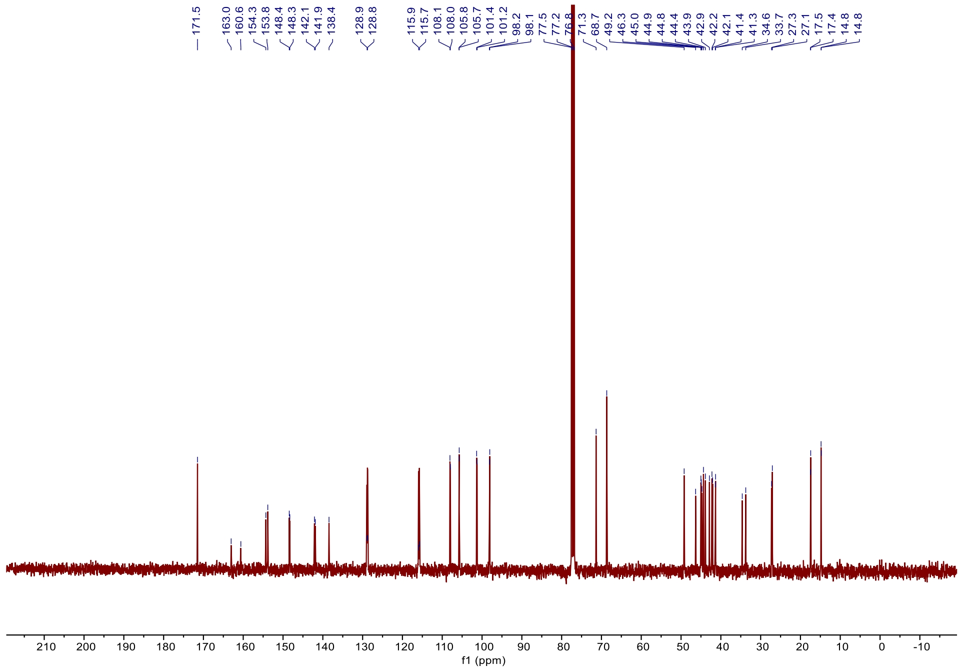

Supplementary Figure 148. ^{13}C NMR (101 MHz, $CDCl_3$) of **3ai-ol**.

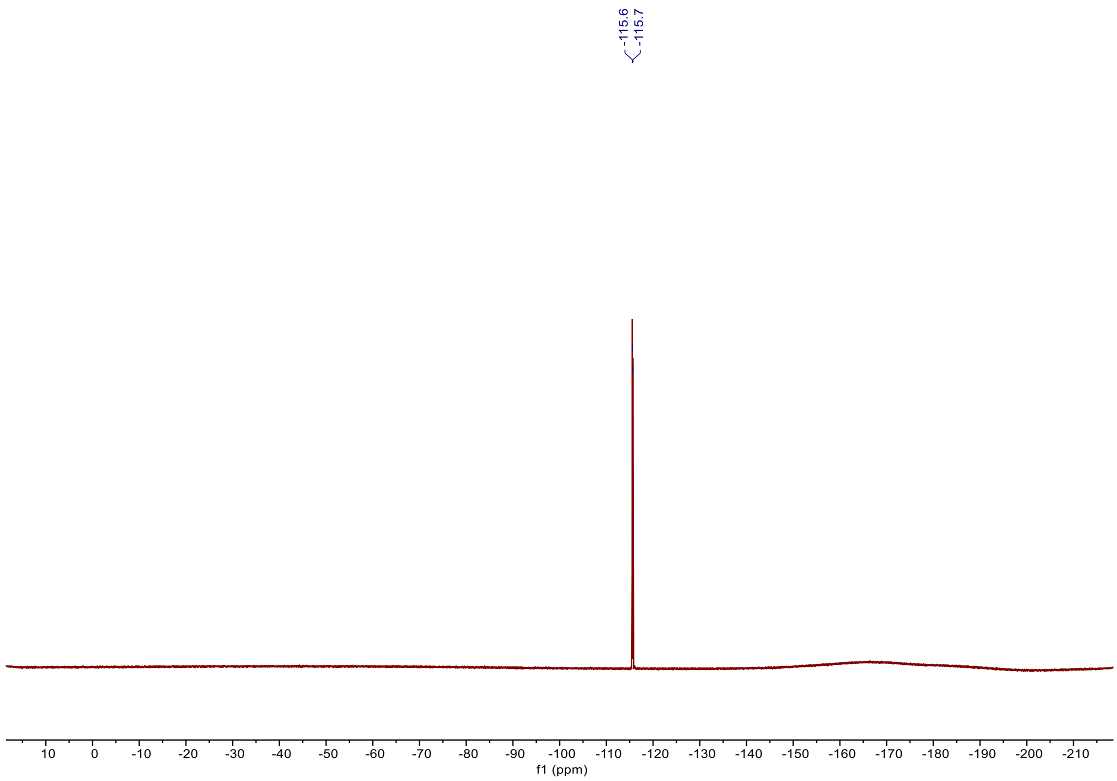

Supplementary Figure 149. ^{19}F NMR (376 MHz, $CDCl_3$) of **3ai-ol**.

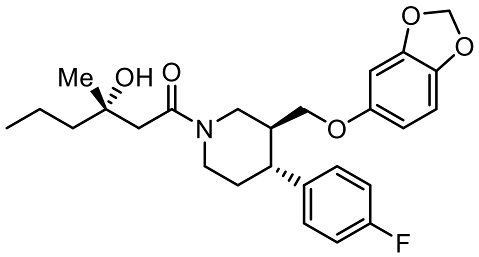

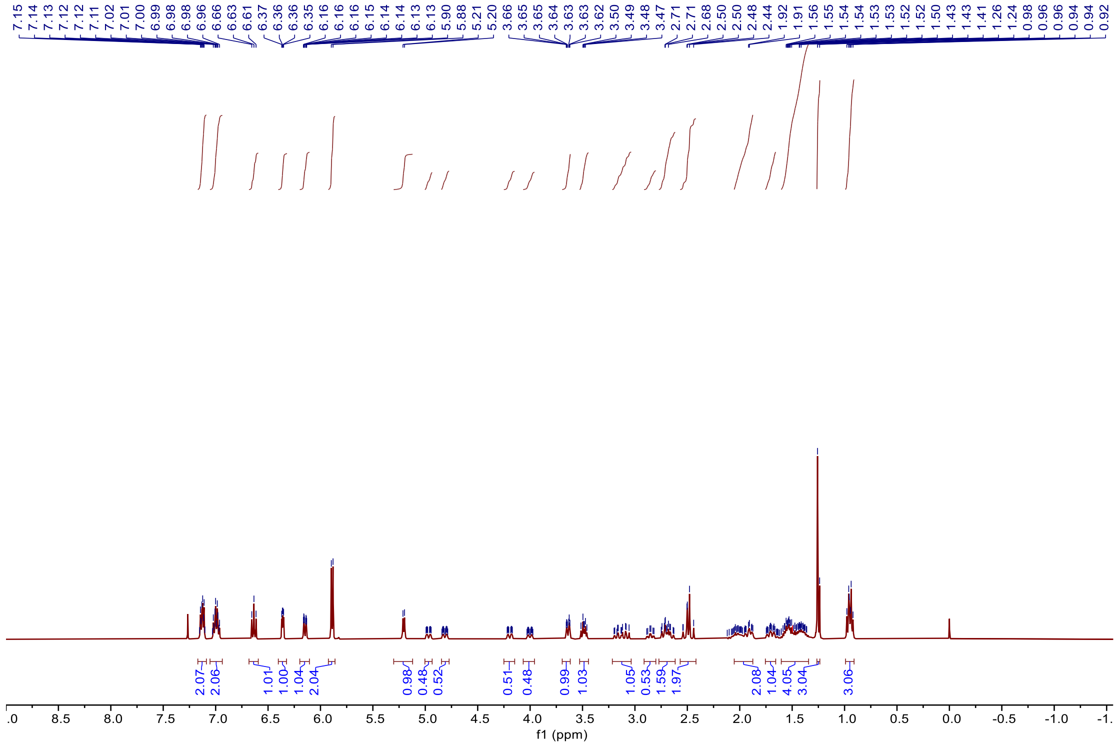

Supplementary Figure 150. ^{1}H NMR (400 MHz, $CDCl_3$) of **3al-ol**.

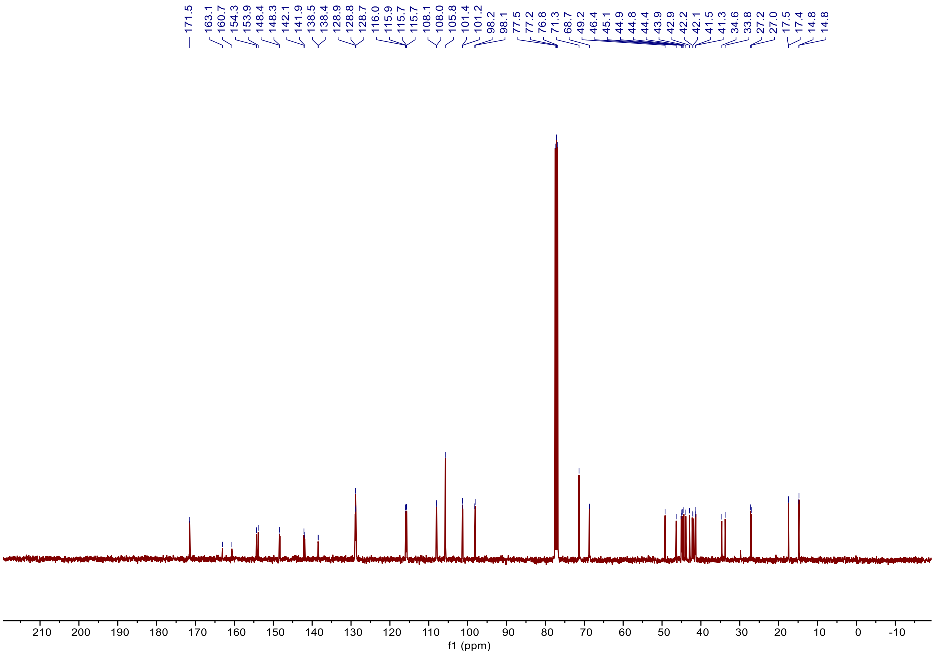

Supplementary Figure 151. ^{13}C NMR (101 MHz, $CDCl_3$) of **3al-ol**.

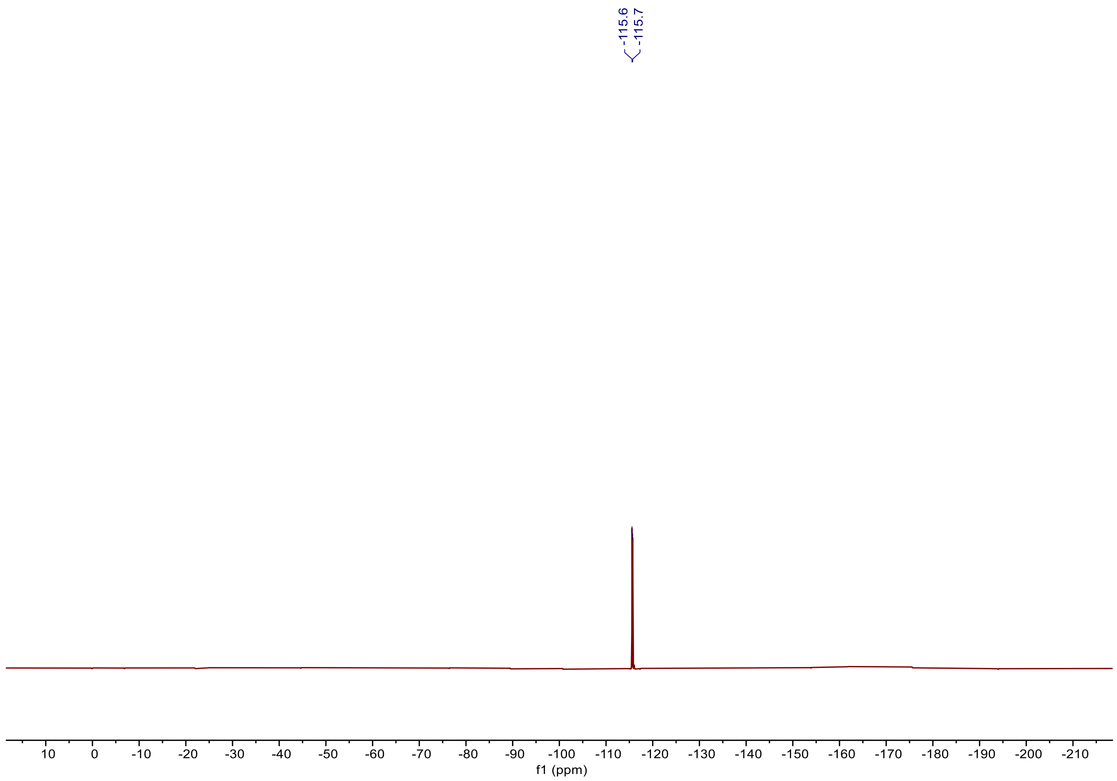

Supplementary Figure 152. ^{19}F NMR (376 MHz, $CDCl_3$) of **3al-ol**.

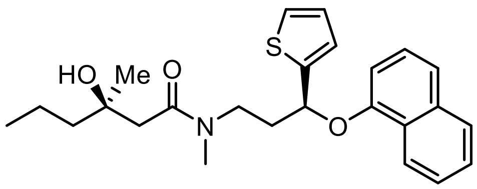

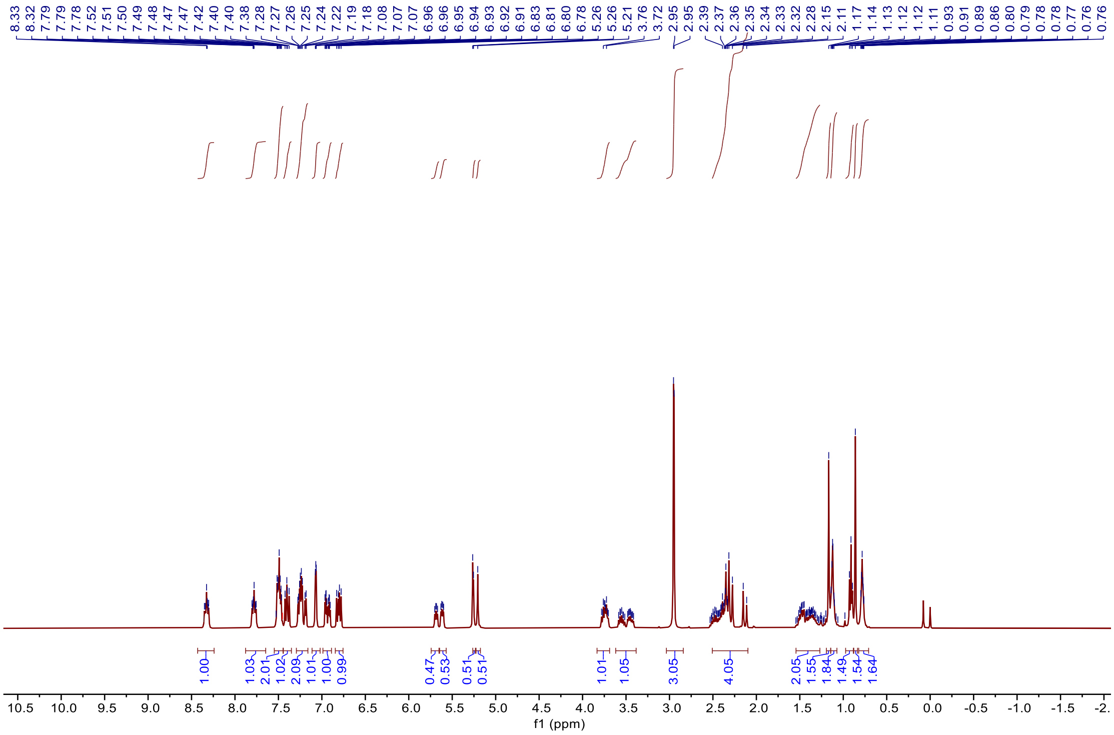

Supplementary Figure 153. ^{1}H NMR (400 MHz, $CDCl_3$) of **3aj-ol**.

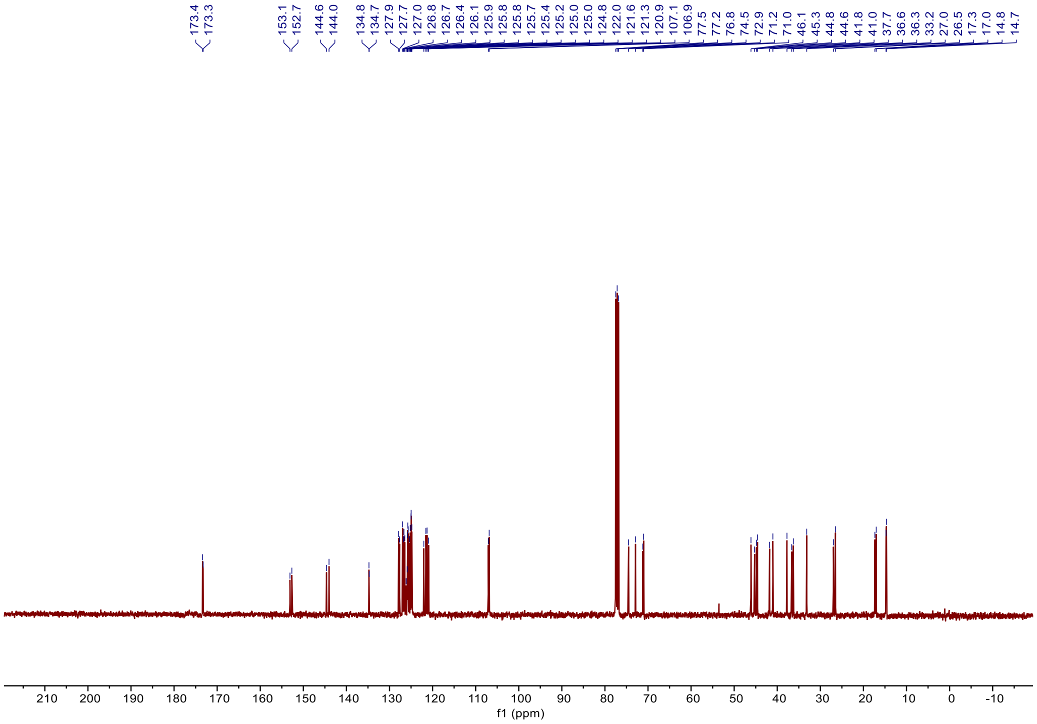

Supplementary Figure 154. ^{13}C NMR (101 MHz, $CDCl_3$) of **3aj-ol**.

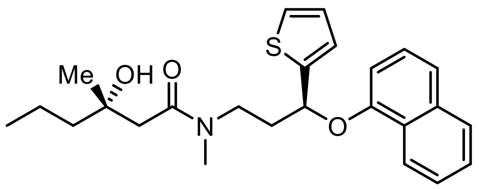

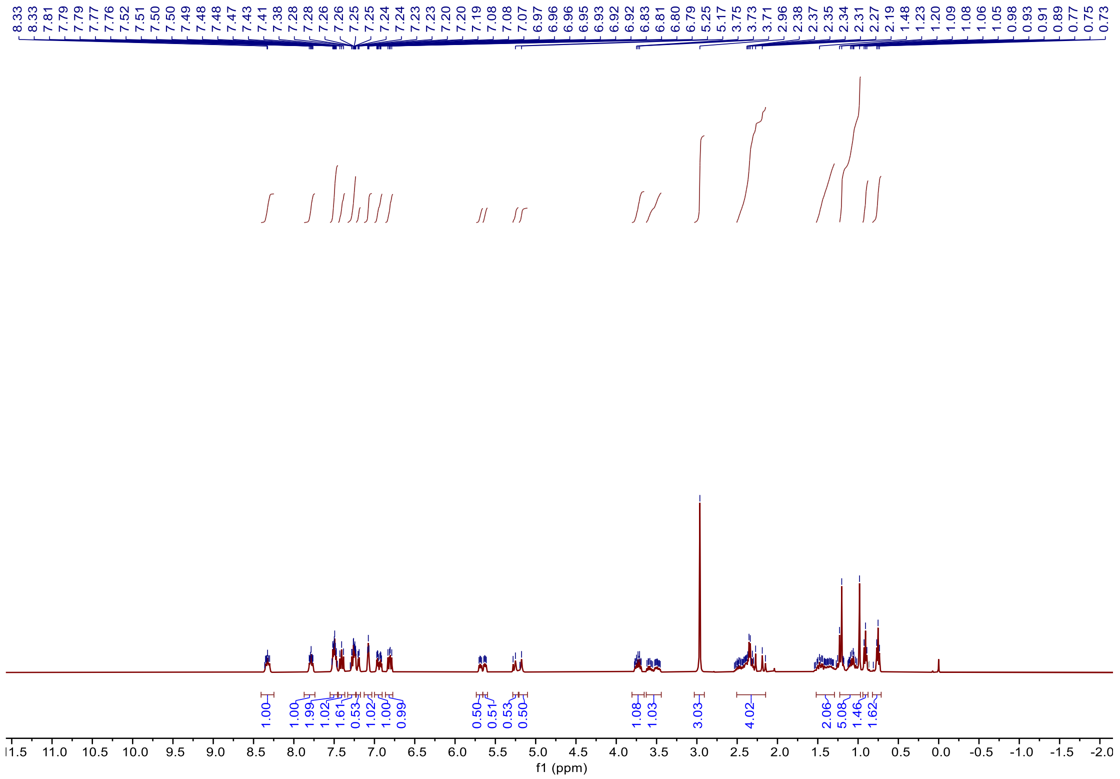

Supplementary Figure 155. ^{1}H NMR (400 MHz, $CDCl_3$) of **3am-ol**.

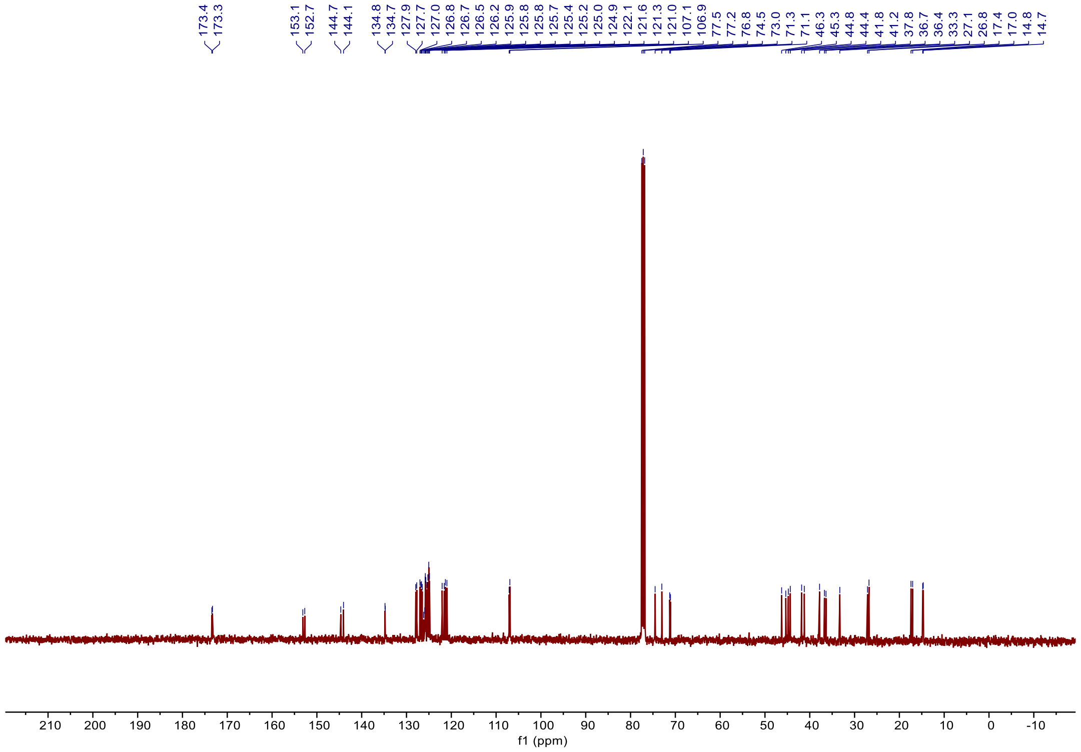

Supplementary Figure 156. ^{13}C NMR (101 MHz, $CDCl_3$) of 3**am-ol**.

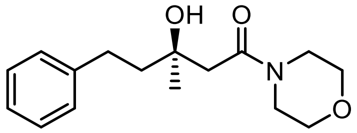

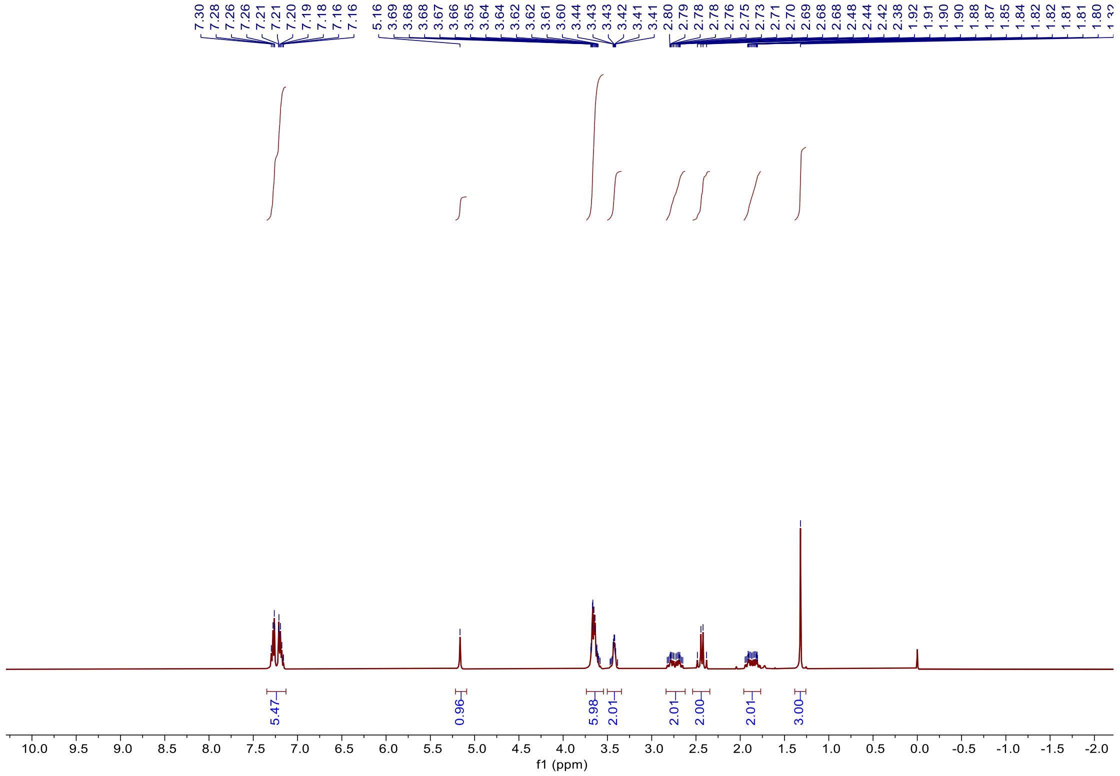

Supplementary Figure 157. ^{1}H NMR (400 MHz, $CDCl_3$) of **4**.

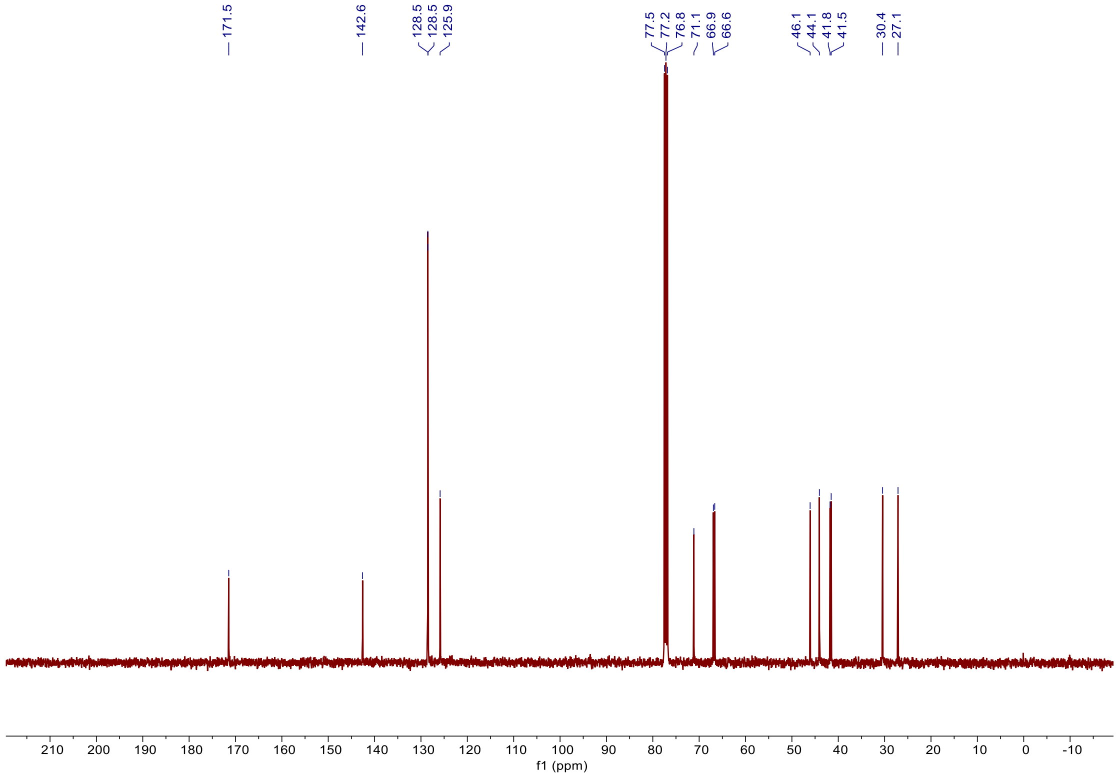

Supplementary Figure 158. ^{13}C NMR (101 MHz, $CDCl_3$) of **4**.

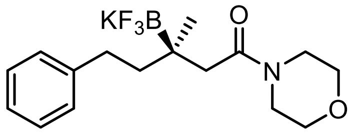

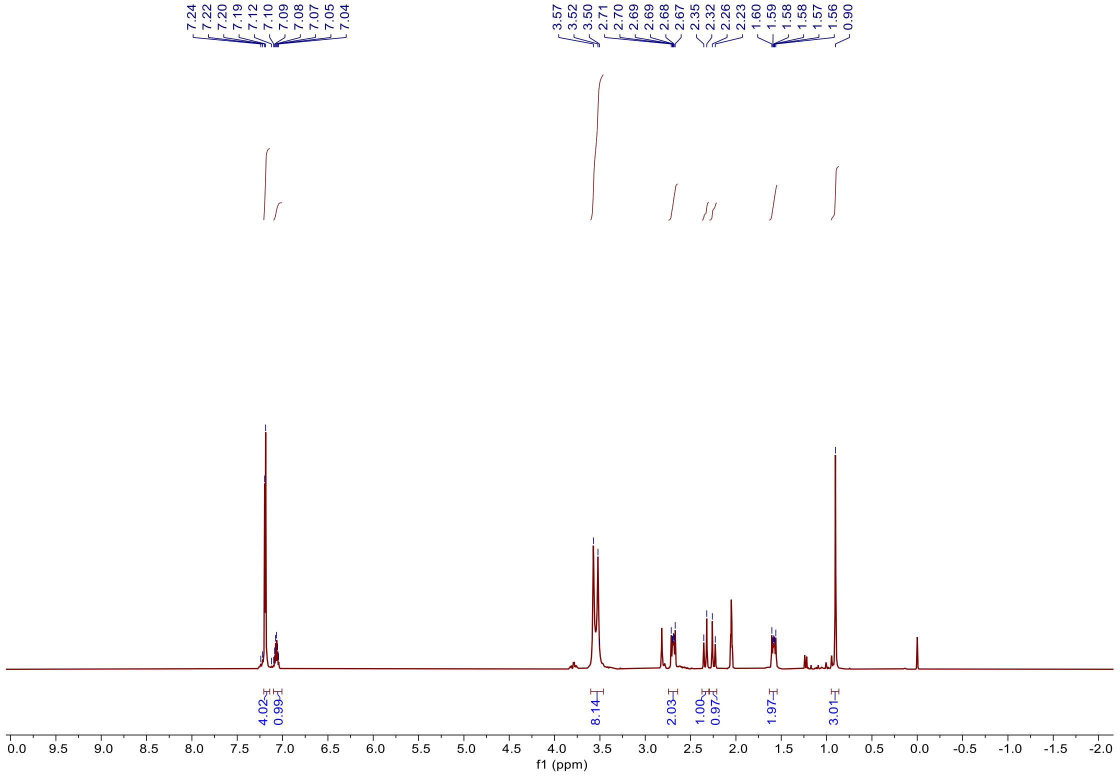

Supplementary Figure 159. ^{1}H NMR (400 MHz, $(CD_3)_2CO$) of **5**.

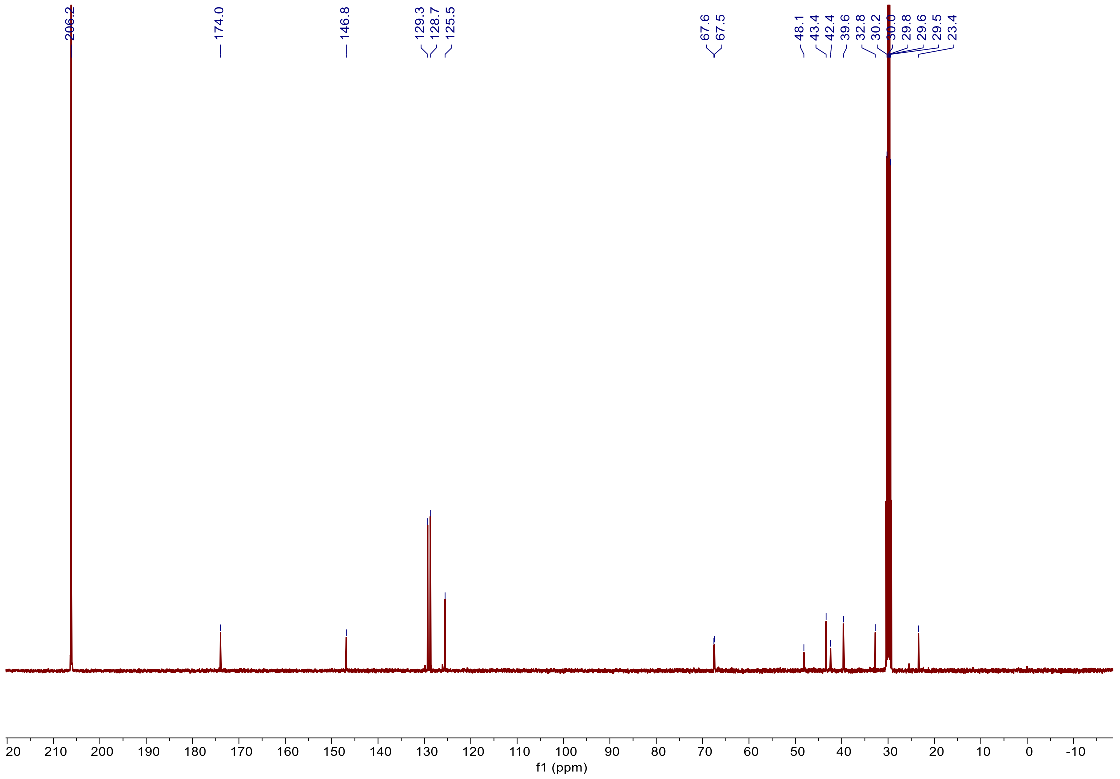

Supplementary Figure 160. ^{13}C NMR (101 MHz, $(CD_3)_2CO$) of **5**.

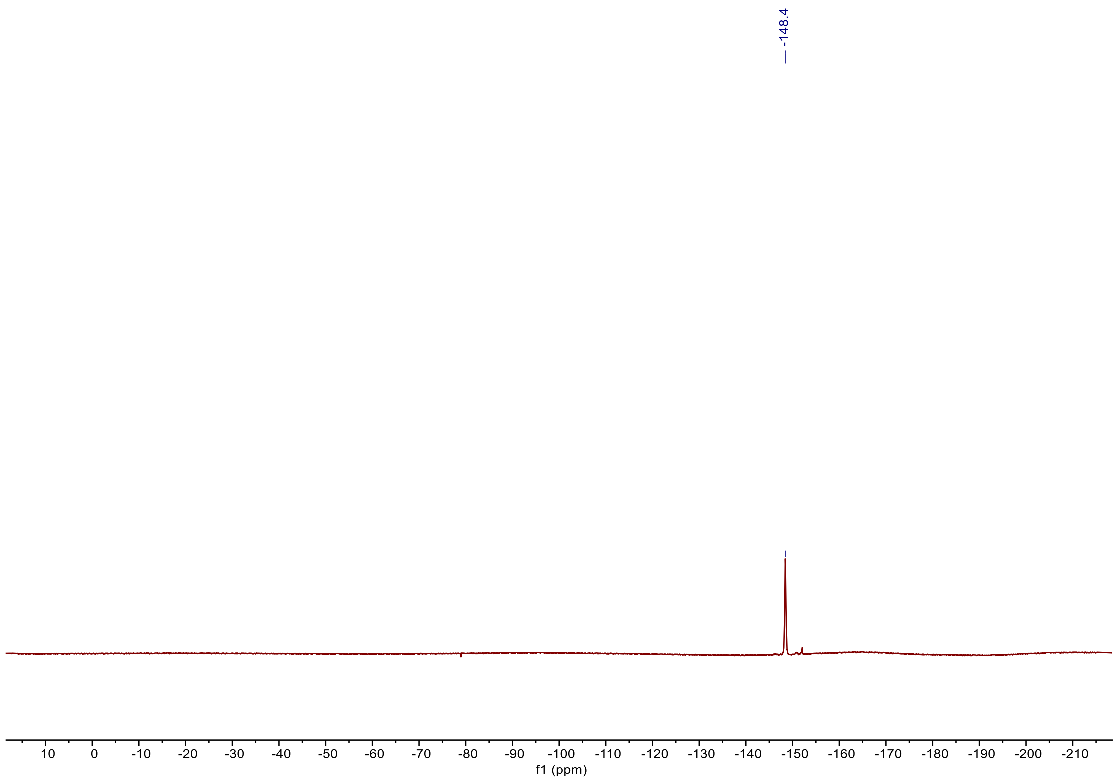

Supplementary Figure 161. ^{19}F NMR (376 MHz, $(CD_3)_2CO$) of **5**.

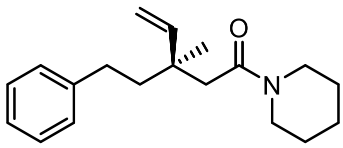

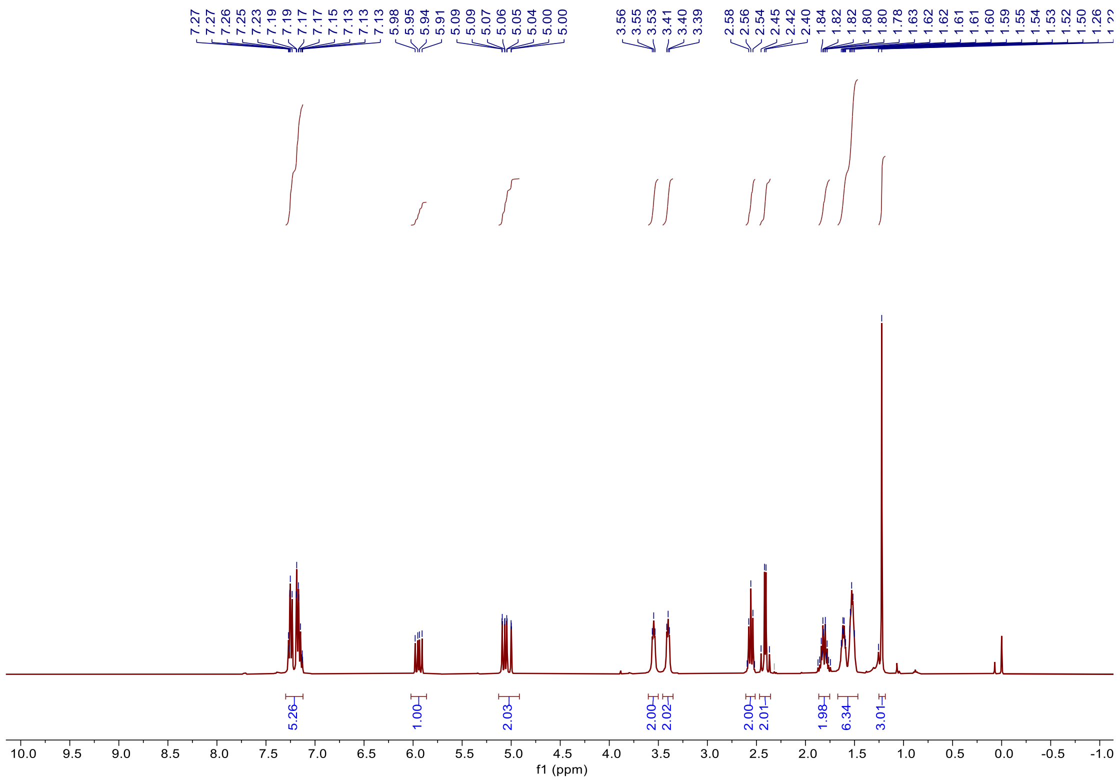

Supplementary Figure 162. ^{1}H NMR (400 MHz, $CDCl_3$) of **6**.

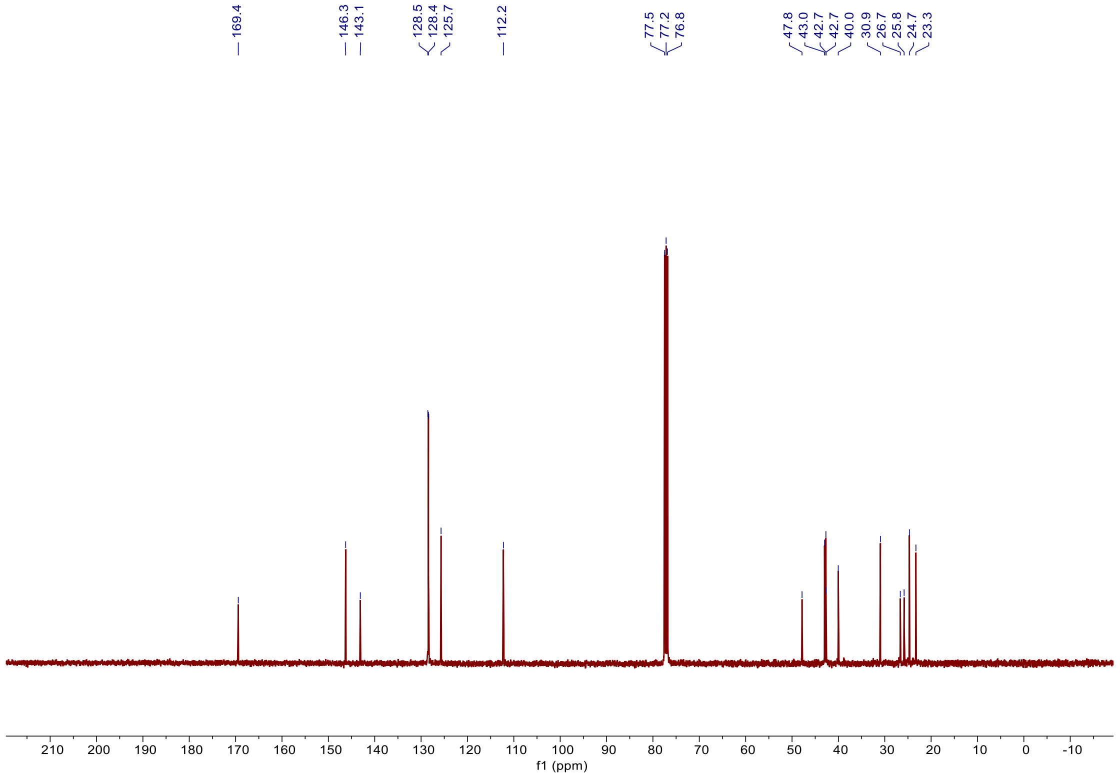

Supplementary Figure 163. ^{13}C NMR (101 MHz, $CDCl_3$) of **6**.

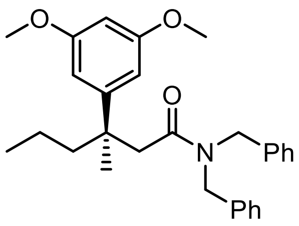

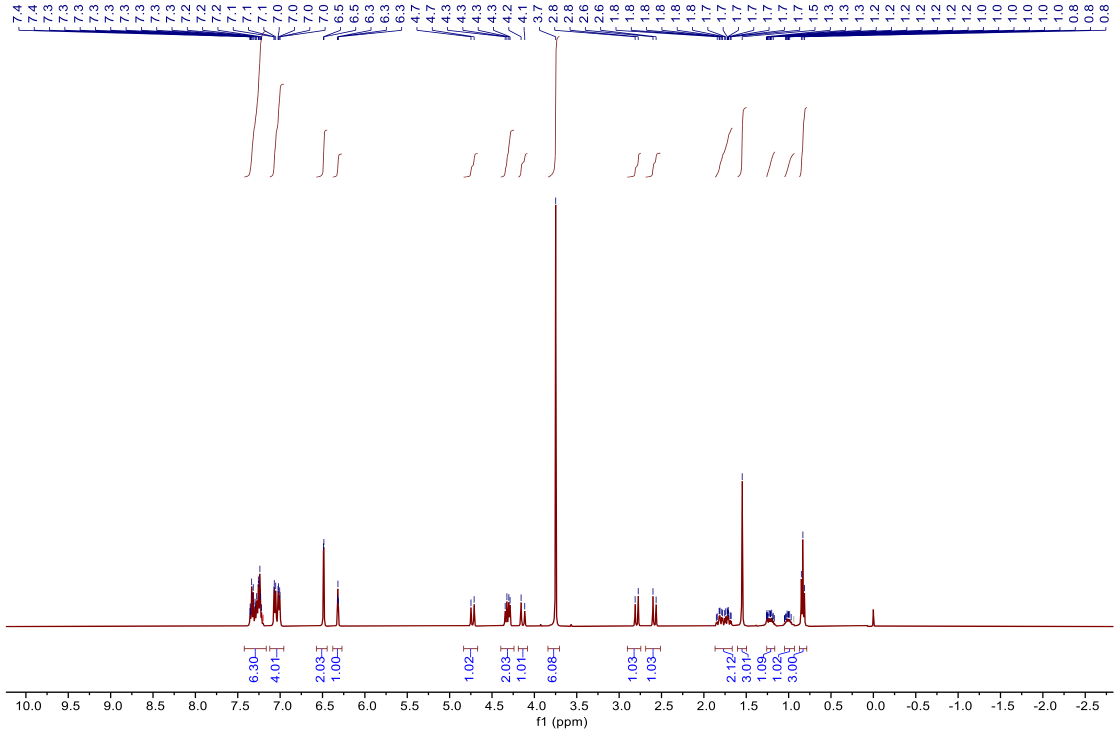

Supplementary Figure 164. ^{1}H NMR (400 MHz, $CDCl_3$) of **7**.

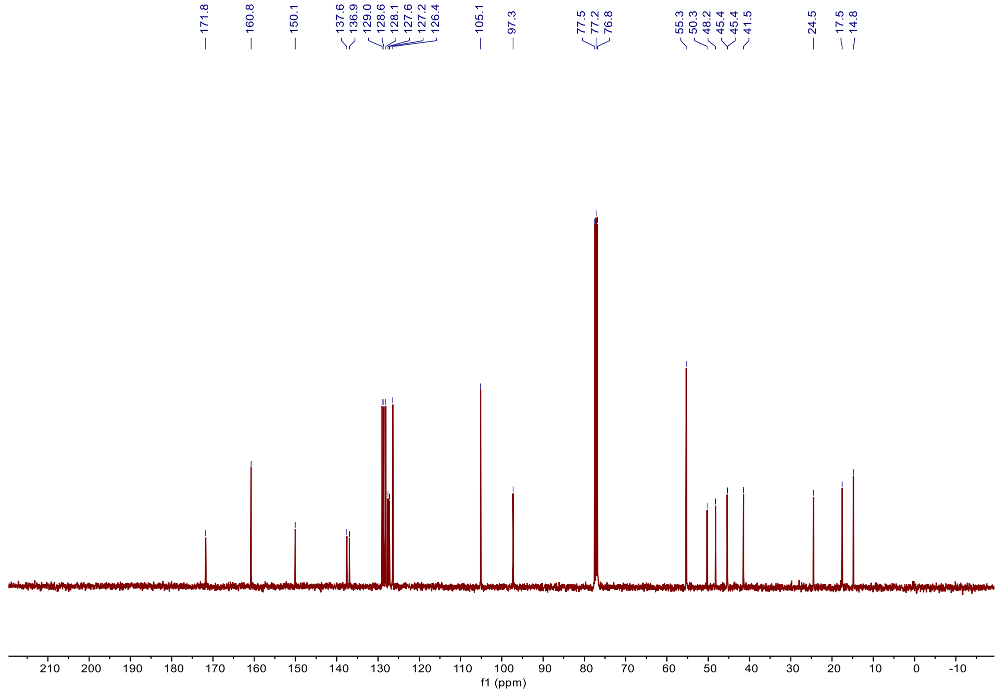

Supplementary Figure 165. ^{13}C NMR (101 MHz, $CDCl_3$) of **7**.

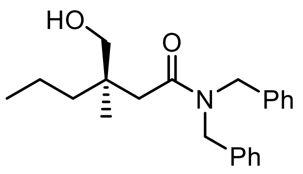

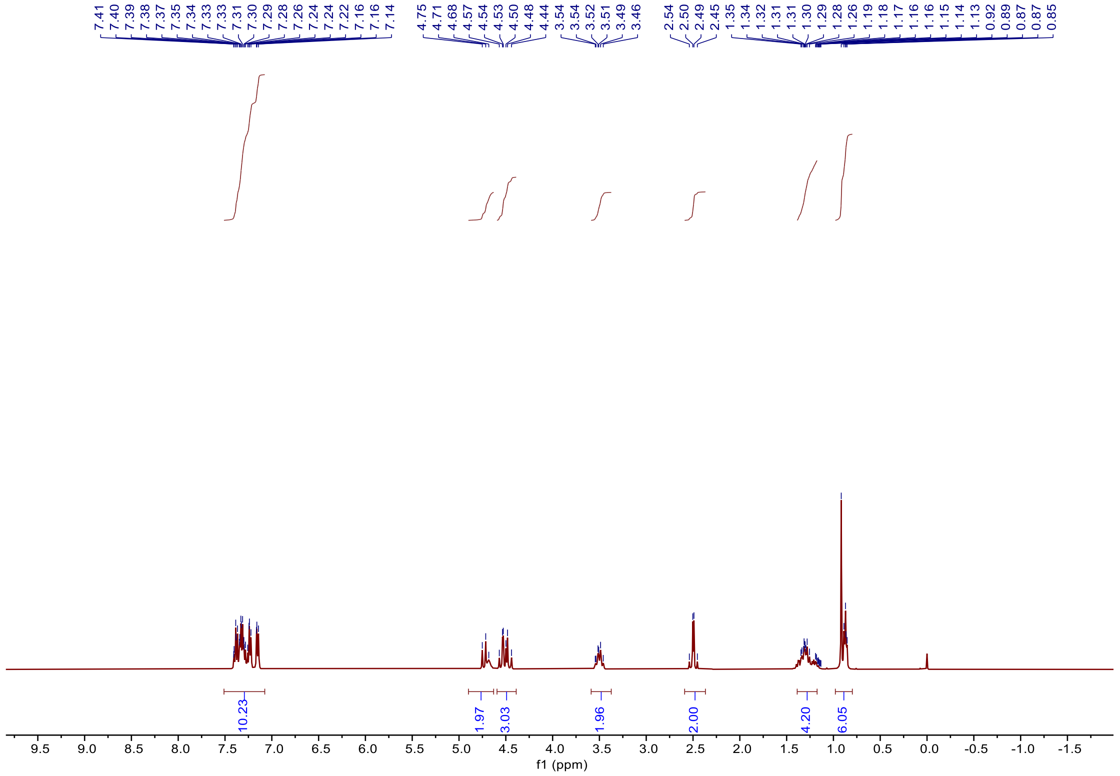

Supplementary Figure 166. ^{1}H NMR (400 MHz, $CDCl_3$) of **8**.

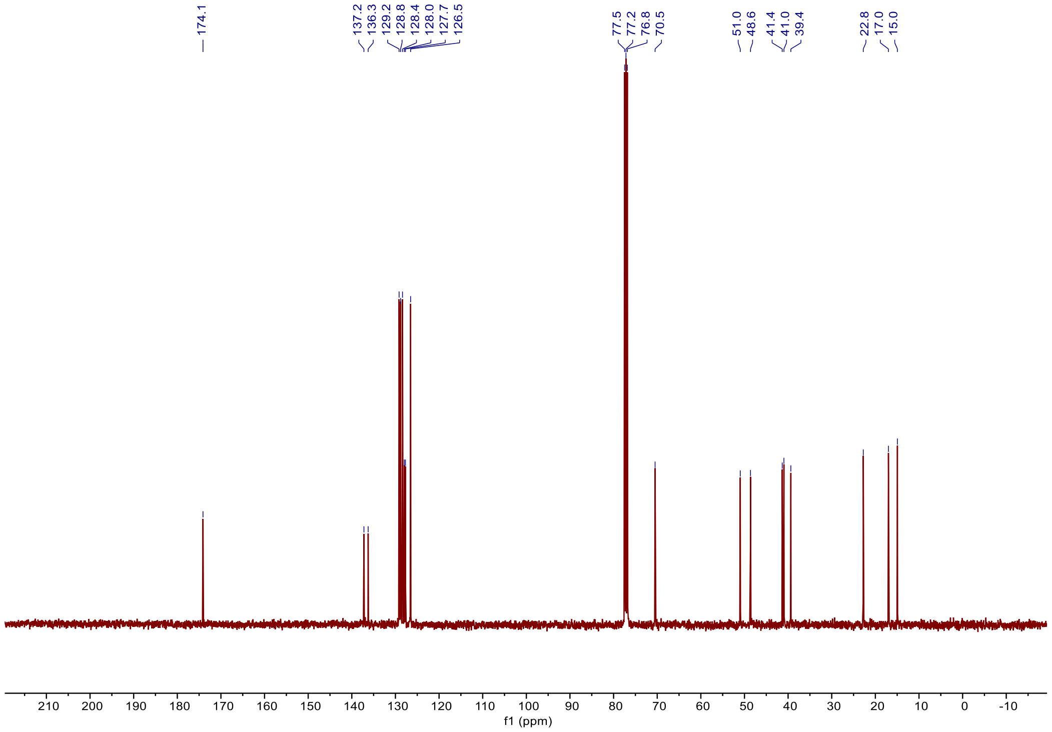

Supplementary Figure 167. ^{13}C NMR (101 MHz, $CDCl_3$) of **8**.

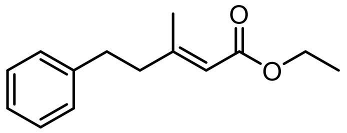

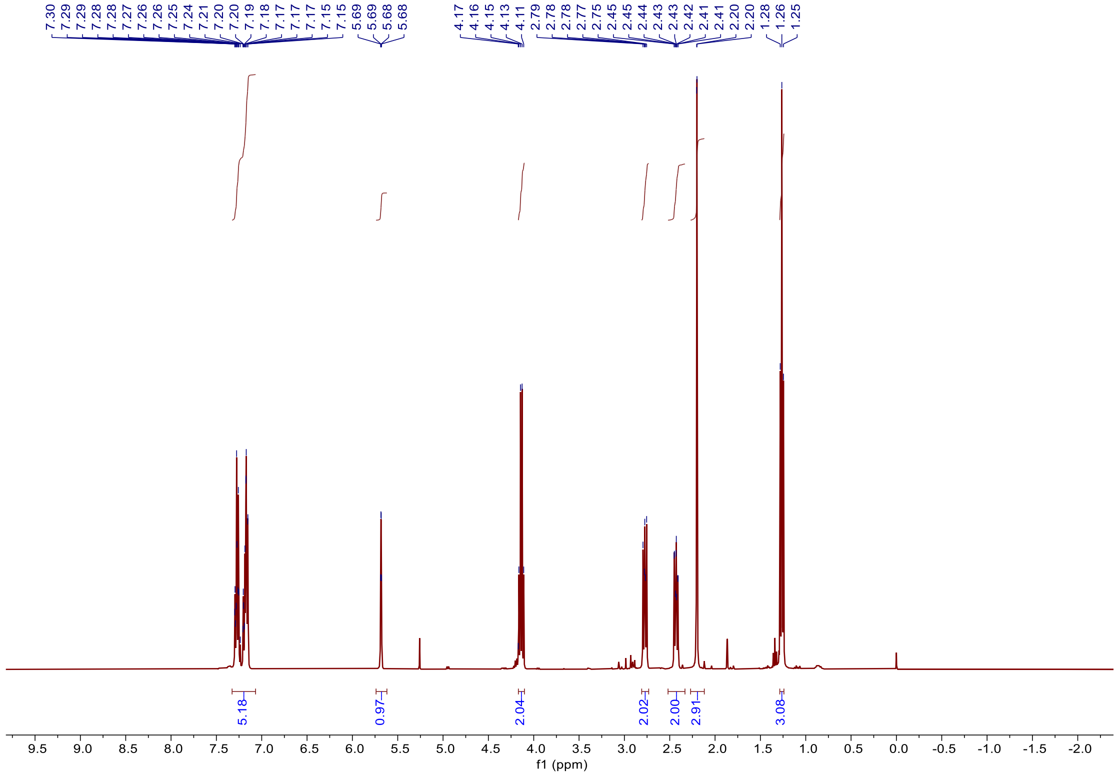

Supplementary Figure 168. ^{1}H NMR (400 MHz, $CDCl_3$) of **9.**

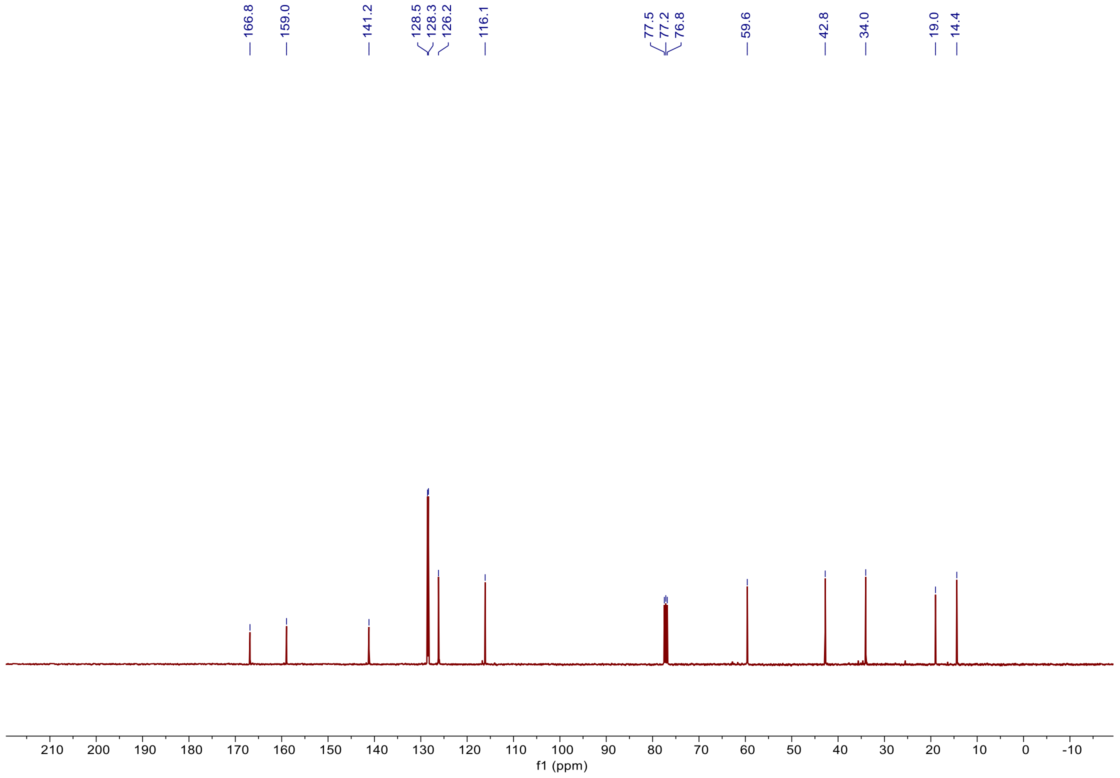

Supplementary Figure 169. ^{13}C NMR (101 MHz, $CDCl_3$) of **9**.

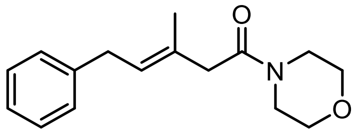

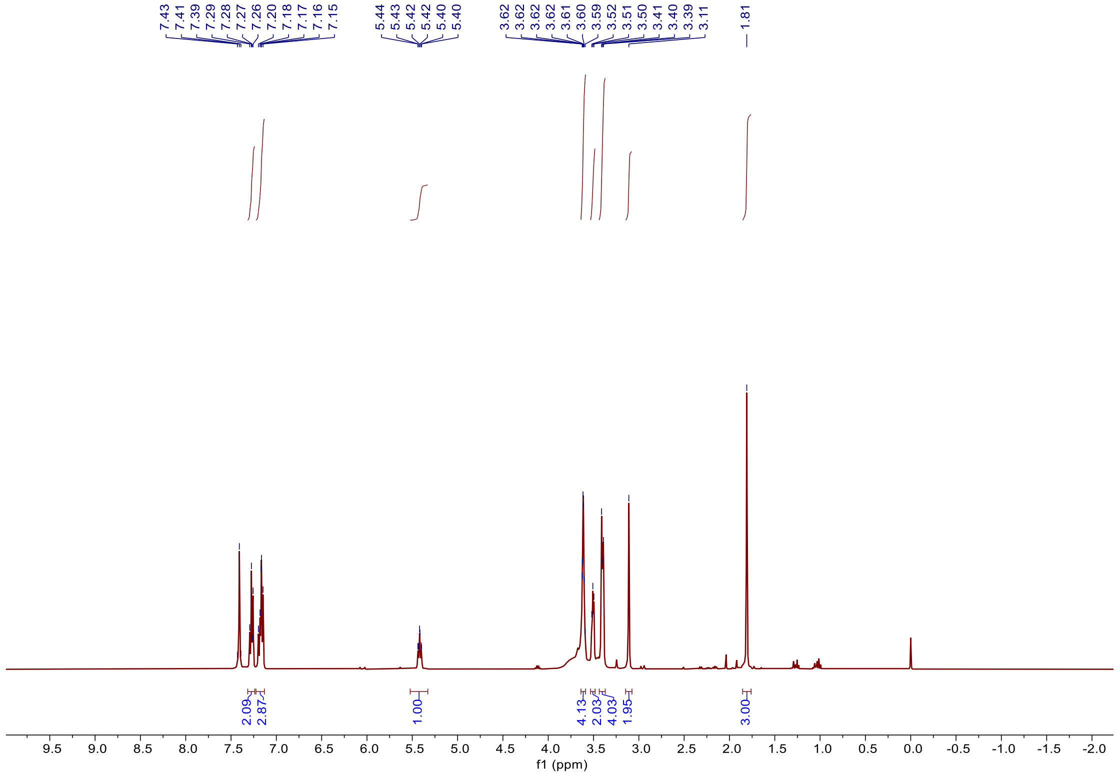

Supplementary Figure 170. ^{1}H NMR (400 MHz, $CDCl_3$) of **10.**

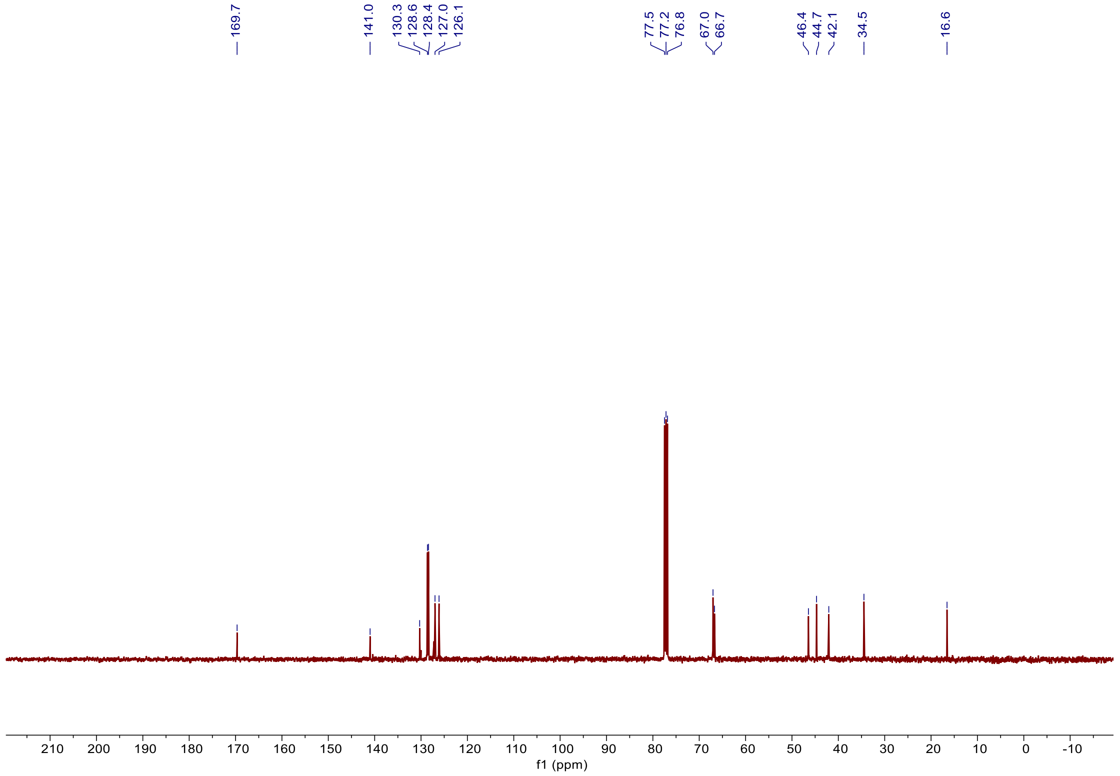

Supplementary Figure 171. ^{13}C NMR (101 MHz, $CDCl_3$) of **10**.

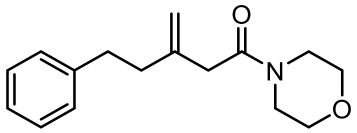

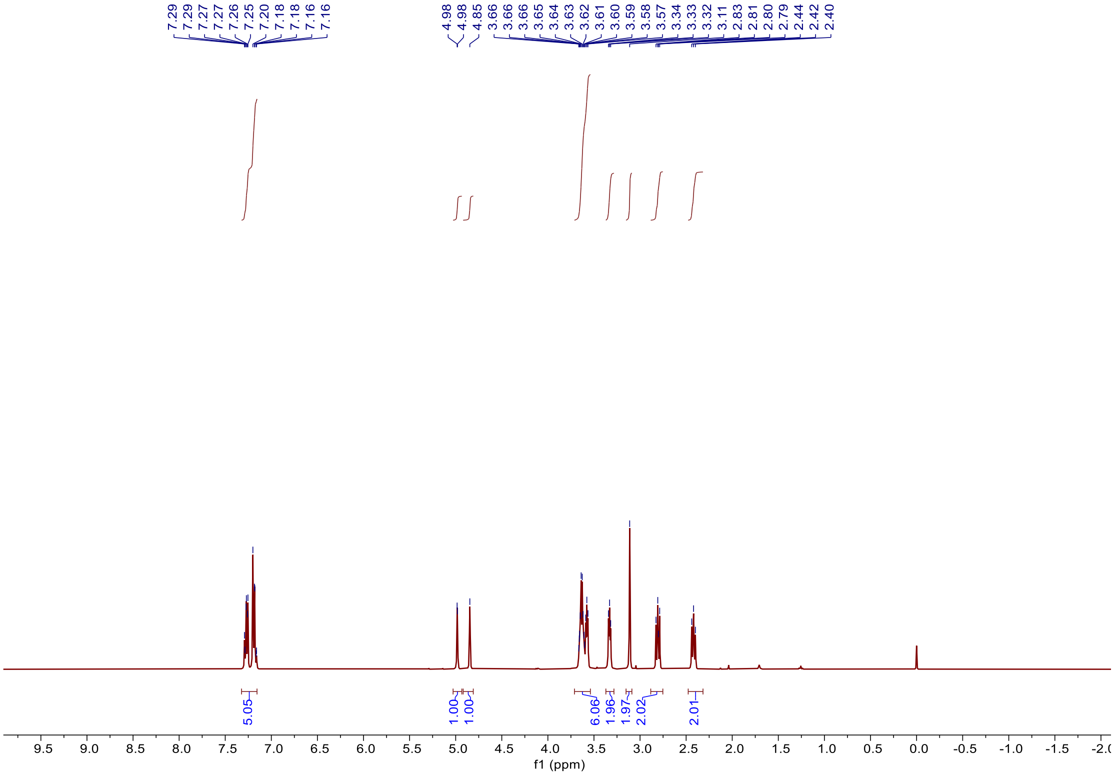

Supplementary Figure 172. ^{1}H NMR (400 MHz, $CDCl_3$) of **11.**

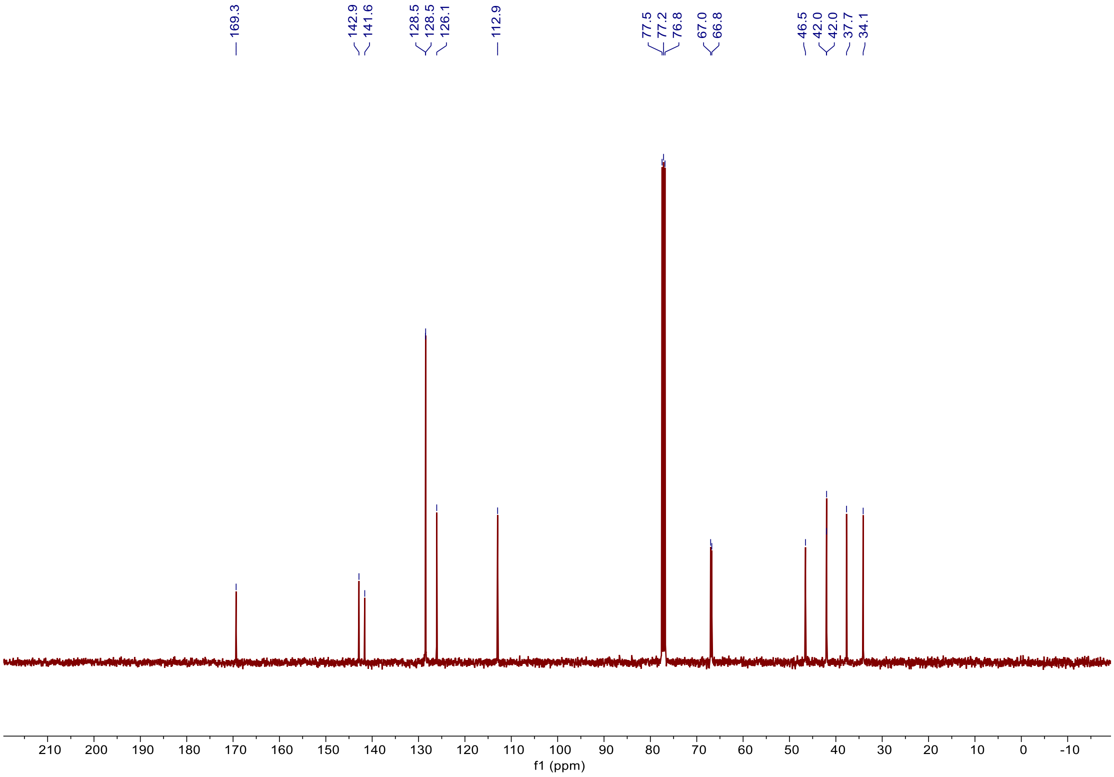

Supplementary Figure 173. ^{13}C NMR (101 MHz, $CDCl_3$) of **11**.

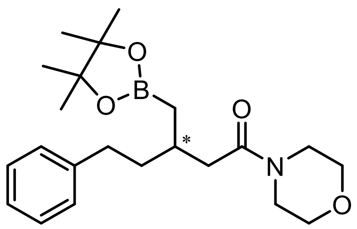

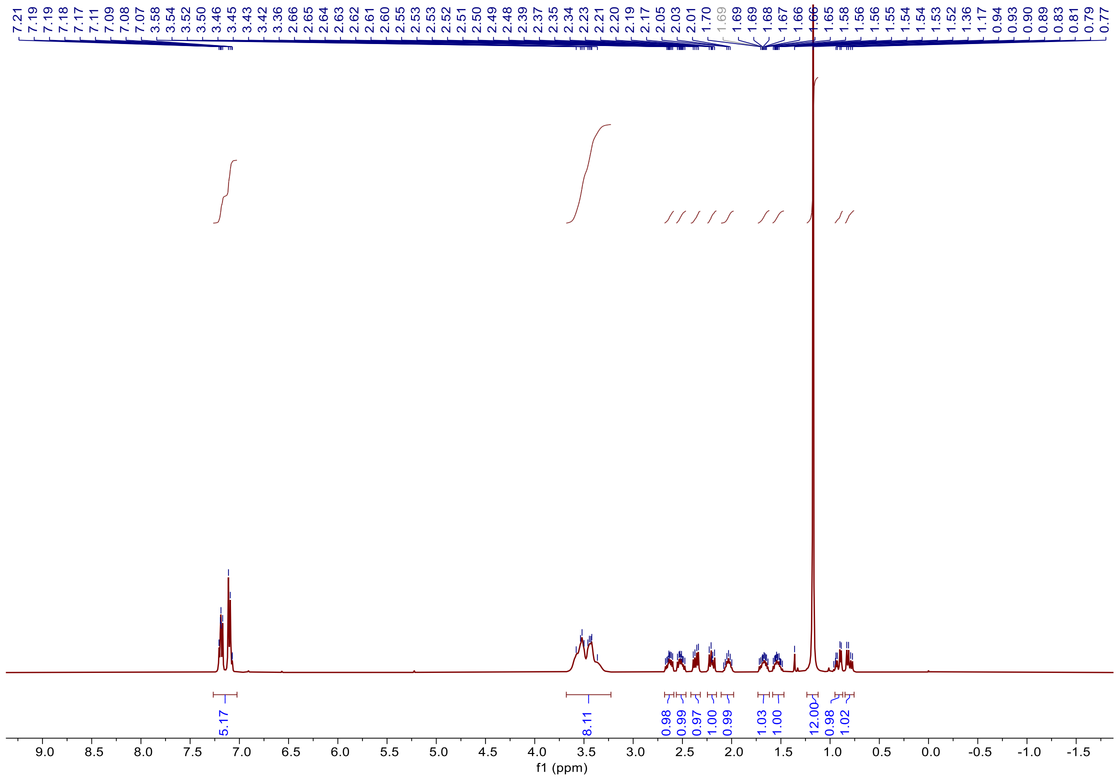

Supplementary Figure 174. ^{1}H NMR (400 MHz, $CDCl_3$) of **11b.**

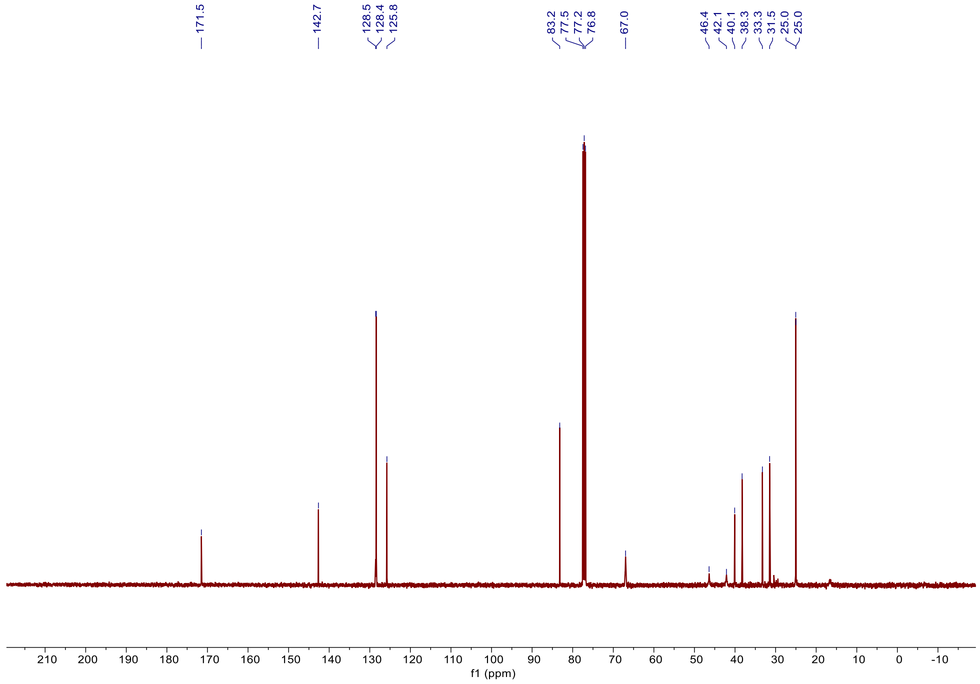

Supplementary Figure 175. ^{13}C NMR (101 MHz, $CDCl_3$) of **11b**.

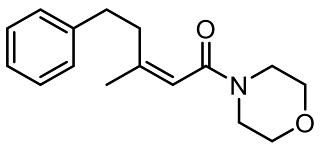

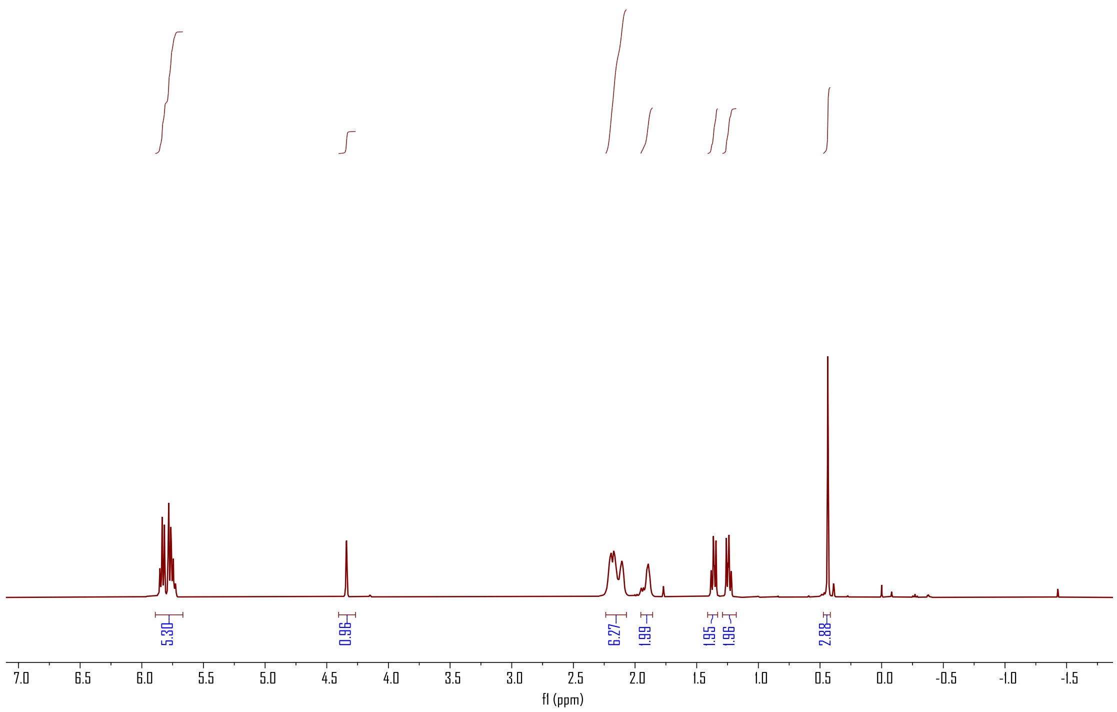

Supplementary Figure 176. ^{1}H NMR (400 MHz, $CDCl_3$) of ***Z*-1i.**

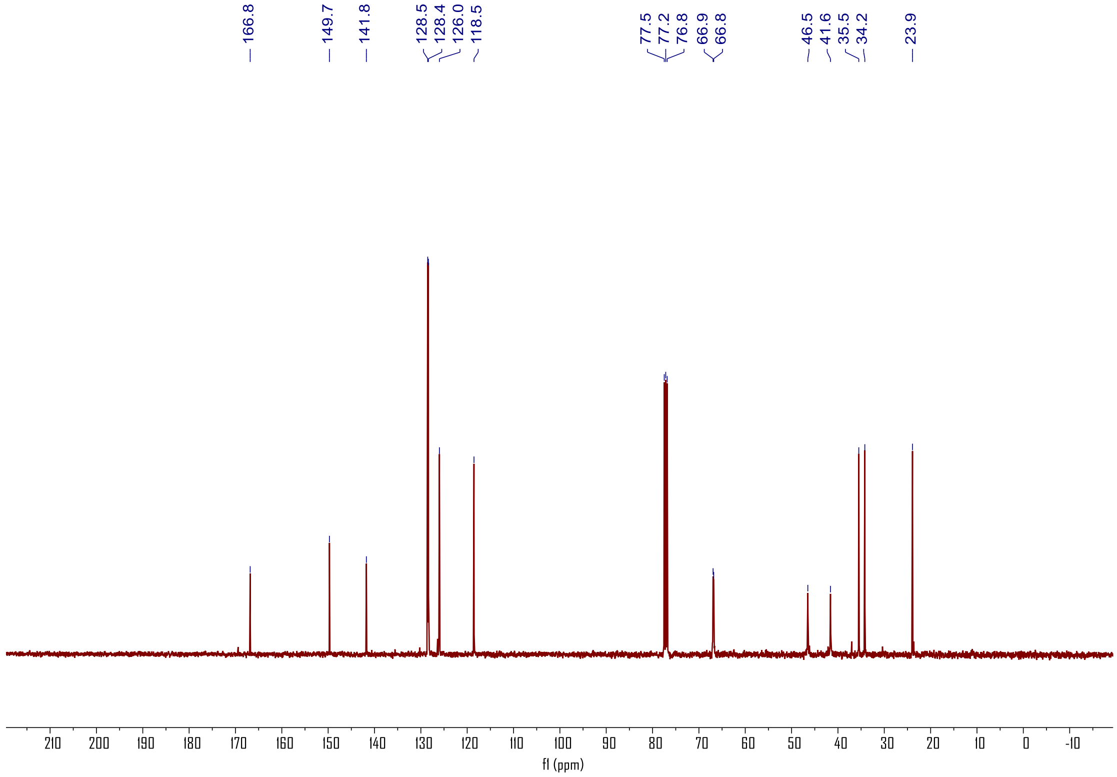

Supplementary Figure 177. ^{13}C NMR (101 MHz, $CDCl_3$) of ***Z*-1i**.

Supplementary References

(1) Tsuchikama, K., Kasagawa, M., Endo, K. & Shibata, T. Cationic Ir(I)-Catalyzed sp3 C−H Bond Alkenylation of Amides with Alkynes. *Org. Lett.* **11**, 1821-1823 (2009).

(2) Lee, J.-E. & Yun, J. Catalytic Asymmetric Boration of Acyclic α,β-Unsaturated Esters and Nitriles. *Angew. Chem., Int. Ed.* **47**, 145-147 (2008).

(3) Liu, Z., Ni, H. Q., Zeng, T. & Engle, K. M. Catalytic Carbo- and Aminoboration of Alkenyl Carbonyl Compounds via Five- and Six-Membered Palladacycles. *J. Am. Chem. Soc.* **140**, 3223-3227 (2018).

(4) Hoang, G. L. & Takacs, J. M. Enantioselective gamma-borylation of unsaturated amides and stereoretentive Suzuki-Miyaura cross-coupling. *Chem. Sci.* **8**, 4511-4516 (2017).

(5) Bonet, A., Odachowski, M., Leonori, D., Essafi, S. & Aggarwal, V. K. Enantiospecific sp2–sp3 coupling of secondary and tertiary boronic esters. *Nat. Chem.* **6**, 584-589 (2014).

(6) Sonawane, R. P. et al. Enantioselective Construction of Quaternary Stereogenic Centers from Tertiary Boronic Esters: Methodology and Applications. *Angew. Chem., Int. Ed.* **50**, 3760-3763 (2011).
